# Understanding metastasis mixed-treatment responses through genomic analyses

**DOI:** 10.1038/s41523-025-00724-z

**Published:** 2025-01-30

**Authors:** Susana Garcia-Recio, Paola Zagami, Brooke M. Felsheim, Amy Wheless, Kerry Thomas, Renato Trimarchi, Lisa A. Carey, Charles M. Perou

**Affiliations:** 1 Department of Genetics, University of North Carolina at Chapel Hill, Chapel Hill, NC USA; 2 Division of Medical Oncology, Department of Medicine, School of Medicine, University of North Carolina at Chapel Hill, Chapel Hill, NC USA; 3 University of Milan, Milan, Italy; 4 Bioinformatics and Computational Biology Curriculum, University of North Carolina at Chapel Hill, Chapel Hill, NC USA; 5 Department of Radiology, School of Medicine, University of North Carolina at Chapel Hill, Chapel Hill, NC USA; 6 Diagnostic and Interventional Radiology Unit, BIOMORF Department, University of Messina, Messina, Italy

**Keywords:** Breast cancer, Cancer genomics

## Abstract

Early-stage and metastatic breast cancers (MBC) can exhibit genomic heterogeneity, even within the same individual. Response to therapy in metastatic breast cancer patients with multiple metastases can also be heterogeneous, with different degrees of responsiveness to the same drug(s) across metastatic sites, termed “mixed response,” within the same patient. Whether this treatment response variability is influenced by factors such as intrinsic tumor characteristics of metastatic lesions and/or the microenvironment is unknown. Through genomic analysis of multiple metastases from the same patient, assayed in 6 different patients who had exhibited mixed response on imaging, we identified that higher regulatory T cells (T reg) and CDKN2A gene expression values correlate with non-response, while the KRAS gene, KRAS amplicon, and CD8T cells were associated with response in individual metastases. These genomic features may explain mixed clinical responses and provide valuable insights into intrapatient variations in treatment sensitivity.

## Introduction

Breast cancer (BC) is the most commonly diagnosed tumor and cause of cancer death among women worldwide, with the vast majority of deaths being caused by metastatic disease^1^. The metastatic process is complex, dynamic, and continuous, representing a critical hallmark of solid tumors^2,3^. In fact, it is the ability of cancer cells to invade distant organs that categorizes breast cancer into its most advanced stage (stage IV/metastatic)^4^. Despite the significant advances towards a more effective therapeutic landscape, de novo and recurrent metastatic breast cancer remain incurable and with poor prognosis^5^. Patients are treated according to clinical subtypes of breast cancer, hormone receptor-positive (HR+), HER2-positive (HER2+), or triple-negative breast cancer (TNBC), based on the expression of hormone receptor (HR) and HER2 protein^6^. In addition, the intrinsic molecular subtypes of BC (Luminal A, Luminal B, HER2-enriched, and Basal-like)^7^ also influence the disease’s response to treatment^8,9^ and the predilection for metastatic sites^10,11^. Other genomic heterogeneity, as observed in DNA and RNA features, plays a role in treatment response^12,13^. This genomic heterogeneity may also contribute to the variable treatment responses seen among metastatic patients (inter-patient variability) and between different metastasis sites within an individual patient (intra-patient variability)^14,15^. It has been described that only about 10% of somatic mutations differ between primary and metastatic tumors^15–17^; however, around 30% of primary tumors undergo a change in gene expression-based molecular subtype during metastasis, resulting in notable changes in the tumor phenotype and microenvironment^14^. For instance, within the same patient, immune cell activation is typically lower in liver metastasis than in other sites like lung metastasis^14^. Not surprisingly, patients with multiple metastases often exhibit different degrees of responsiveness to the same drug(s), a phenomenon known as “mixed response” across metastatic sites. However, it remains unclear whether this variability in treatment response is influenced by inherent tumor cell features, the surrounding microenvironment, or both. There are relatively few studies specifically evaluating mixed responses across metastatic sites within the same patient, and these responses can vary significantly depending on the cancer type and treatment regimen. For example, in metastatic renal clear cell carcinoma, 56% of patients on anti-angiogenic tyrosine kinase inhibitors exhibited mixed responses, associated with poorer overall survival^18^. Similarly, 25.2% of colorectal cancer patients with liver metastases demonstrated radiological heterogeneity in response to chemotherapy, linked to poorer survival outcomes^19^. In breast cancer expressly, dissociated metabolic responses were observed in 48% of cases, primarily affecting bone lesions^20^. In the present study, using our classification criteria, we observed mixed responses in 40% of the patients in our cohort, highlighting the complexity of response patterns in metastatic breast cancer. Given these observations, we used the UNC Rapid Autopsy Program to select patients with documented mixed responses on serial imaging during a line of metastatic therapy, multiple metastases, and available tissue samples from sites of varying response to treatment. We performed genomic analysis of multiple metastases, with matched metastasis-specific imaging response, and sought to identify genomic features that might explain the biology behind the differential response to treatment in different metastatic sites within the same patient.

## Results

### Clinicopathological characteristics of the study population

**Fig. 1:**
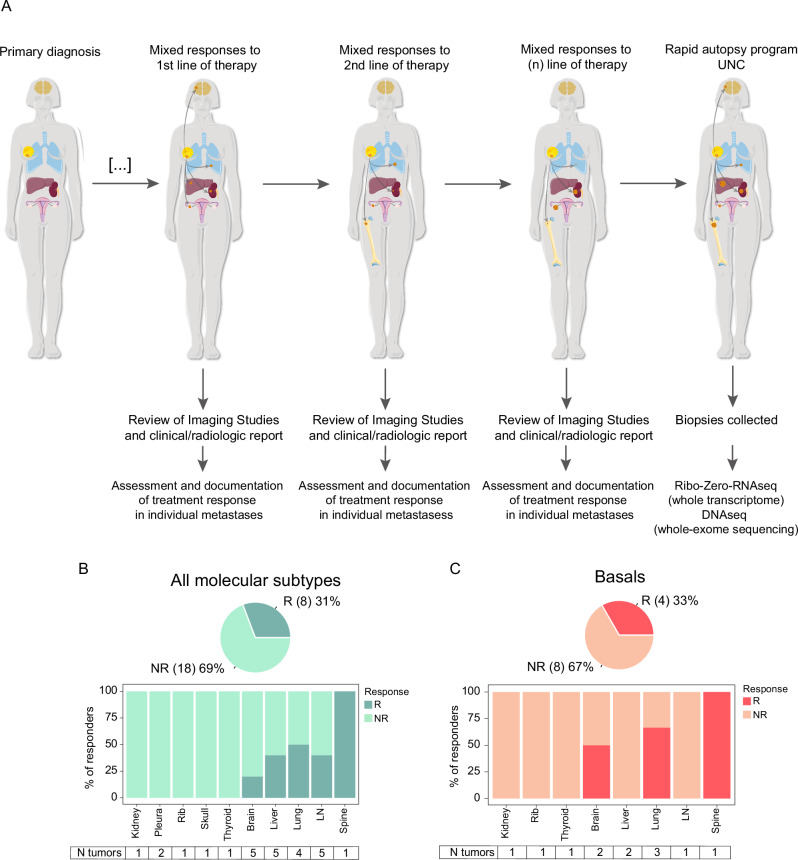
Radiographic responses to therapy in the metastatic setting. **A** Illustrative diagram depicting how the study was conducted. **B** Pie chart and bar plot illustrating the total number of patients and the distribution of organ sites, respectively, for all molecular subtypes divided by Responders (R) and Nonresponders (NR). **C** Pie chart and bar plot illustrating the number of patients and the distribution of organ sites, respectively, for Basal-like only divided by Responders (R) and Nonresponders (NR).

From the UNC Rapid Autopsy Program (RAP) cohort (see Methods), we took tumor imaging response measurements for different patients at various time points across different lines of therapy. We documented the response to treatment for each metastasis in each patient (Fig. 1A and Supplementary Data 1). The response to treatment was categorized for each metastasis, defining “response” as a complete or partial response when at least one of the treatment lines showed a response or as disease stabilization, based on RECIST 1.1 criteria. Patients demonstrating mixed responses to a single line of therapy in metastatic sites where tissue was available were selected for further analysis.

Of the 15 individual RAP patients with multiple metastases who underwent RNA sequencing, 6 patients (40%) met our classification criteria for mixed responses and were included in the analysis. This final study population comprised 6 patients with mixed responses, encompassing 26 metastases analyzed for gene expression (an average of 4 metastases per patient). Among these 6 patients, 1 had stage IV disease at diagnosis, 4 had TNBC, and 2 had hormone receptor-positive HER2-negative disease (HR + /HER2-). The 26 sites of metastases from 6 primary breast tumors were distributed as follows: 5 were brain, 5 liver, 4 lung, 5 lymph nodes, and 7 were found in various other locations named here as “other” (Table 1). These patients underwent a median of 4 lines of systemic therapy; all of them received chemotherapy, and only one also received endocrine therapy (Supplementary Data 1).

**Table 1:** Clinicopathologic characteristics of the metastases analyzed in the study cohort of 6 patients

Variable	*N* = 26^a^	Basal, *N* = 16^a^	Lums-HER2E, *N* = 10^a^	*p*-value^b^
Tissue Type				
Metastasis	26 (100.0%)	16 (100.0%)	10 (100.0%)	
Response				0.664
Responders	8 (30.8%)	4 (25.0%)	4 (40.0%)	
Nonresponders	18 (69.2%)	12 (75.0%)	6 (60.0%)	
Anatomic Site				0.275
Brain	5 (19.2%)	5 (31.3%)	0 (0.0%)	
Liver	5 (19.2%)	2 (12.5%)	3 (30.0%)	
Lung	4 (15.4%)	3 (18.8%)	1 (10.0%)	
Lymph node	5 (19.2%)	2 (12.5%)	3 (30.0%)	
Others	7 (26.9%)	4 (25.0%)	3 (30.0%)	
PAM50 Call				**<0.001**
Basal	16 (61.5%)	16 (100.0%)	0 (0.0%)	
LumA	5 (19.2%)	0 (0.0%)	5 (50.0%)	
LumB	5 (19.2%)	0 (0.0%)	5 (50.0%)	
ER/PR/HER2				**<0.001**
ER + /HER2-	8 (30.8%)	0 (0.0%)	8 (80.0%)	
TNBC	18 (69.2%)	16 (100.0%)	2 (20.0%)	

^a^n (%)

^b^Fisher’s exact test

Bold text indicates statistically significant *p*-values (<0.001) from Fisher’s exact test.

In terms of PAM50 molecular subtypes, 16 of these metastases were Basal-like, 5 Luminal A, and 5 Luminal B. Of 26 sites, 8 metastatic lesions showed a response to an individual line of treatment with 18 nonresponders making the primary comparison groups (Fig. 1B). We further subcategorized patients into two groups based on whether lymph nodes were included within metastasis responders, given the confounding with immune activation analyses. The first group (including those with lymph node metastases) contained all different molecular subtypes, with 8 responding and 18 nonresponding sites. However, after removing those patients with lymph nodes among responders, the remaining patients all had Basal-like tumors (4 responders and 8 non-responders) (Fig. 1C)), so we will refer to this second group as “Basal-like/TNBC”. This also helped limit the contribution of tumor intrinsic subtype switching, which is well-documented in Basal-like tumors but seldom seen in other subtypes.

### Molecular signatures of treatment response and resistance

Ribo-ZERO whole transcriptome sequencing was performed on these tumor specimens, and the gene expression data was transformed into a set of 836 pre-determined signatures as previously described (see Methods)^21^. These signatures covered various biological processes, cellular components, and pathways. They also included features of tumor cell phenotypes and their microenvironment, as well as more than 100 immune cell signatures, some of which have prognostic and predictive value^22–25^. We also included RNA-derived DNA amplification/deletion signatures, indicating regions leading to the overexpression or downregulation of genes in those segments^21^.

**Fig. 2:**
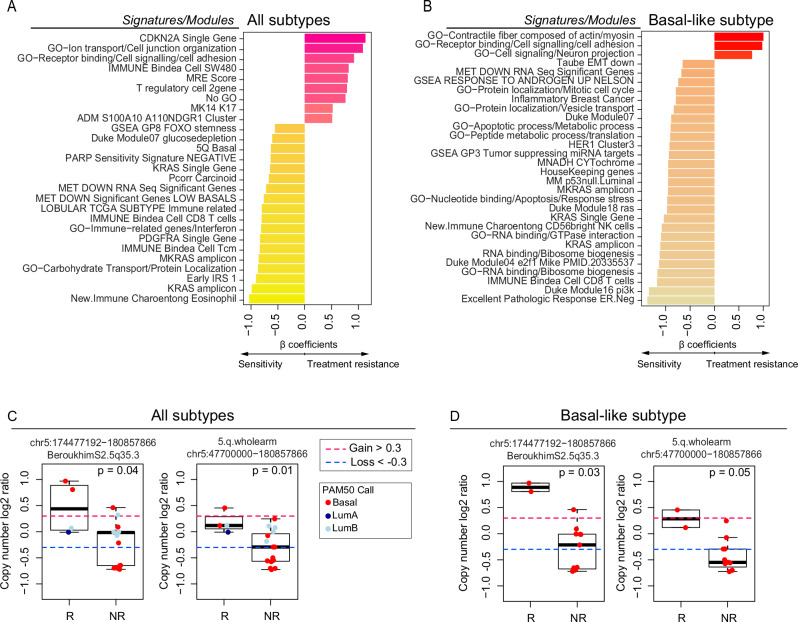
Supervised analysis of gene expression signatures according to response to treatment. **A** Bar plot depicting the differentially expressed (DE) signatures between responders (*n* = 8) and nonresponders (*n* = 18) in the RAP cohort using all metastatic samples. **B** Bar plot depicting the DE signatures between responders (*n* = 4) and nonresponders (*n* = 8) in the RAP cohort using Basal-like metastasis. Significant signatures are ordered from high to low according to β coefficients (regression coefficients). Signature scores were calculated in the UQN, log2 transformed, batch-corrected, and adjusted for normal data (see Methods). **C** CN changes between responders (*n* = 4) and nonresponders (*n* = 15) depicting the list of significant segments from the supervised analysis using all samples. **D** CN changes between responders (*n* = 2) and nonresponders (*n* = 11) depicting the list of significant segments from the supervised analysis using Basal-like samples only. Comparisons between the two groups were performed by a two-sided, unpaired Wilcoxon test. All box and whisker plots display the median value on each bar, showing the lower and upper quartile range of the data (Q1 to Q3). The whiskers represent the lines from the minimum value to Q1 and Q3 to the maximum value. For more information about the background/origin of the signatures and CN segments listed in this figure, see Supplementary Data 3–6. R Responders, NR Nonresponders, GO Gene Ontology.

To find gene expression features associated with a mixed response to treatment, we performed supervised learning with linear regression incorporating “RNA sequencing-based gene expression signatures” as a predictor, non-responder/responder as a fixed effect, and “patient” as a random effect. We performed supervised analyses of all 26 metastases comparing responders versus nonresponders using this library of signatures and identified 27 signatures as being differentially expressed and correlated with response or nonresponse independently of the site of metastasis (*p* < 0.05) (Fig. 2A), including KRAS high expression, KRAS amplicon, CD8/immune-related signatures. We also found elevated expression of RAD50 and RAD17, both of which are included in the 5q11-35 signature and are typically associated with DNA repair^26^. On the other hand, higher T regulatory (T reg) cells, CDKN2A gene expression, and cell junction organization were associated with non-response in metastasis. Subtype-specific differences were observed when supervised analyses were performed within a gene expression subtype that is known to be associated with the likelihood of metastasis to a specific site^10^. Specifically, in Basal-like/TNBC metastasis (*N* = 12), the aptly named “Excellent Pathologic Response” signature^27^ was highly associated with responders. Other associations with response included the RNA-binding signature, CD8 T cells, the PI3K pathway, KRAS gene and amplicon, and some gene ontology related to protein localization and response to stress and apoptosis.

We also calculated 534 recurrent DNA segment-level scores using the DNA Copy Number data (Methods and Supplementary Data 2, 5, 6). We performed supervised analyses of 19 available metastases (all molecular subtypes) or only Basal-like/TNBC metastasis. Our analysis indicated significantly higher CN segments at chromosome 5 bands q31-35 among responders compared with nonresponders (q-value < 0.05). These results were consistent with our prior RNAseq analysis (Fig. 2C, D and Supplementary Data 3–6).

### Gene expression variability in mixed-response patients and corresponding images

One of the objectives of our study was to explore the complexities and variabilities in cancer treatment response across different metastatic sites. We examined our cohort’s responders and their diverse treatments, and no clear pattern or correlation existed between a specific treatment and the response observed (Supplementary Data 1). Consequently, and due to the limited sample size of our dataset, variables such as the patient’s treatment and the site of metastasis were not included in our initial supervised learning model.

**Fig. 3:**
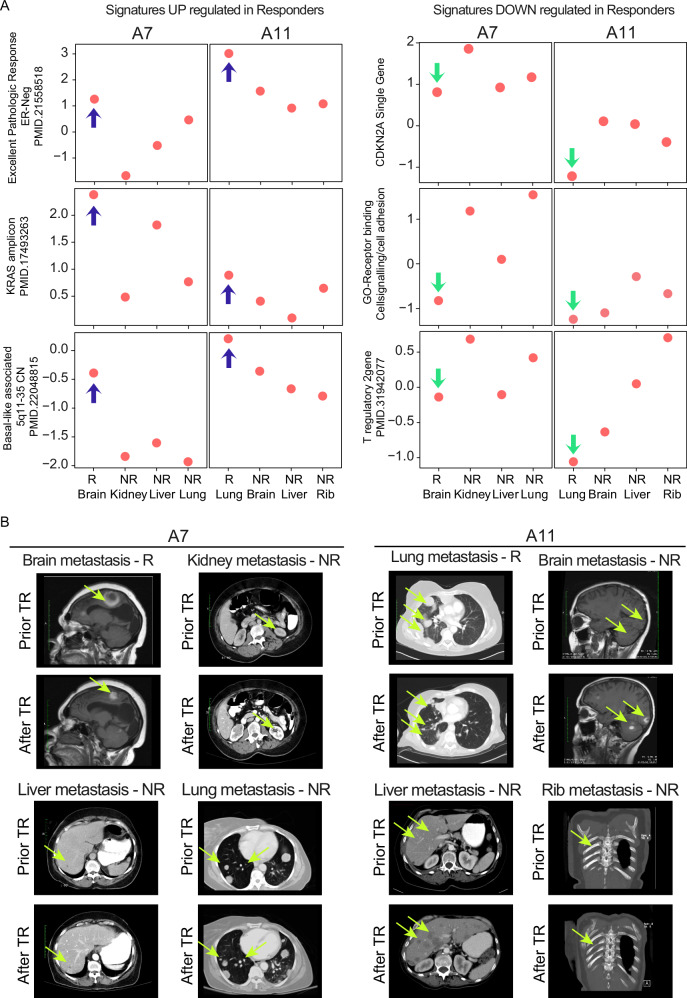
Variability of gene expression signatures in mixed-response patients and their corresponding images. **A** Gene expression signature scores of some representative signatures used in the supervised analysis of Fig. 2, according to the individual sites of metastasis shown in patients A7 and A11. Blue arrows indicate gene expression signatures significantly enriched in responders, while green arrows indicate those depleted. **B** Screenshots of brain-MRI and thoracic/abdomen-CT scans of patients A7 and A11 and the corresponding site of metastasis showing discordant treatment response (TR), with fluorescent yellow arrows marking the tumor areas.; GO Gene Ontology.

As a clear example of mixed response, two patients, A7 (Fig. 3A and Supplementary Fig. 1A) and A11 (Fig. 3A and Supplementary Fig. 1B), exhibited variation in their response to treatment across their four metastases. For patient A7, the brain metastasis, which demonstrated a significant response to capecitabine treatment along with radiotherapy, was characterized by high expression of the following genomic signatures: Excellent Pathologic Response, KRAS Amplicon, and genes associated with chromosome 5q11-35. Patient A11 exhibited a similar pattern, but in this case, the lung metastasis responded better than the brain, liver, and rib metastasis (Fig. 3A, left panel). Interestingly, in the metastases that responded well to radiotherapy and capecitabine (brain in A7, Supplementary Fig. 1A) or the combination of gemcitabine plus carboplatin treatment (lung in A11, Supplementary Fig. 1B), there was a noticeably lower expression of the CDKN2A gene, stem-cell features, and T reg cell signature, unlike the other metastasis (Fig. 3A, right panel). Corresponding images from the CNS MRI and chest/abdominal CT of patients A7 and A11 (Fig. 3B) illustrate this discordant treatment response.

## Discussion

Metastatic breast cancer is uncurable, and its complexity intensifies when considering the varied responses each metastatic site exhibits to current treatments. Using genomic analysis of multiple metastases from individual patients, paired with imaging documentation of site-specific responsiveness, this study aimed to identify features associated with treatment sensitivity and resistance by analyzing metastases with different responses to treatment in the same patients.

Conducting these analyses is challenging due to the inherent difficulties in acquiring multiple metastatic biopsies from a single patient, which we overcame by using metastatic specimens obtained at the time of autopsy. Using this valuable resource, we performed gene expression analysis and found significant differences in gene expression signatures between metastases that responded to treatment and those that did not. This scenario was mainly observed in our cohort of Basal-like subtype patients, and the genomic differences between responders and nonresponders across their metastases were highlighted. Notably, in Basal-like tumors, metastases with higher cell signaling activity and increased cell adhesion were associated with non-responsiveness within an individual patient. Conversely, metastases with activation of the KRAS gene and KRAS amplicon were associated with a greater likelihood of treatment response. Other features identified in responsive tumors included epithelial-mesenchymal (EMT) downregulation, RAS and E2F1 signaling, PI3K, and signatures related to CD8T and NK cells.

When analyzing all tumors, higher T reg cell activity and increased expression of CDKN2A were associated with non-responsiveness within an individual patient, consistent with the known function of T reg cells in suppressing anti-tumor immune responses. Elevated levels of CDKN2A (p16) in Basal-like/TNBC have been previously shown to be associated with resistance to chemotherapy^28–30^. Conversely, the KRAS gene and KRAS amplicon were associated with treatment response, with KRAS gene overexpression being a recurrent characteristic of sensitivity in our two analyzed groups; this was not expected, and additional experiments will be needed to determine the mechanistic role of KRAS in this treatment sensitivity. Other features enriched in sensitive metastases included several immune signatures where CD8 T cells, central memory T cells (Tcm), interferon, and eosinophils seem to be involved.

Basal-like primary tumors frequently exhibit loss of the 5q region^26,31,32^, traditionally linked to more aggressive tumor behavior, increased genomic instability, and, presumably, a less favorable response to treatment. Our analysis indicated a positive association between increased copy number in chromosome 5q (suggesting retention) and treatment response. Specifically, consistent expression of RAD50 and RAD17, genes within the 5q11-35 signature, may improve treatment response by providing more effective DNA repair mechanisms^26,31,32^, thereby contributing to better patient outcomes when analyzing all tumors and Basal-like only. Further research is necessary to fully dissect the relationship between these genes located in chromosome 5q, drug responsiveness, and resistance in metastases.

There are several limitations of our study. First is our small sample size, however, we do note we obtained statistically significant results from our supervised learning analyses. Second, although we identified common features of sensitivity across tumor subtypes and metastatic sites, it is known that drivers of metastasis can vary significantly according to molecular subtype and the site of metastasis, and we are not powered to perform analyses according to the site of metastasis. To validate our observations, a larger cohort encompassing multiple metastases per patient and mixed-treatment responses would be necessary. It should be noted that we performed a sub-analysis focusing only on those patients who did not have lymph nodes among responders and were all Basal-like subtype of breast cancer, representing the most homogeneous group in our study in terms of molecular subtype. We identified features related to mixed response that made biological sense (i.e., stemness and immune features, consistent with previous studies associating with responsive sites). Finally, it is crucial to note that the metastatic samples used for genomic analysis were from autopsy and were separated in time from the treatment and their response/nonresponse to it.

In summary, in a small but unique cohort of patients with mixed response to metastatic therapy, examination of multiple metastases within individual patients identified common genomic features predicting response and resistance. Several confirm previously noted roles in response, such as CD8 T cells, or resistance, such as T regulatory cells, suggesting that variations in these features across involved organs may be contributing to outcome in MBC.

## Methods

### Clinical summary

Tumor tissue was obtained from patients with metastatic breast cancer who consented to participation in the Rapid Autopsy Tumor Donation Program (RAP) at UNC, as previously described^33^. This study followed ethical regulations, including adherence to the Declaration of Helsinki. Eligible patients with MBC were identified from the UNC Rapid Autopsy Program (RAP) based on having primary tumors and multiple metastases in at least two different sites, along with RNA sequencing data available^21,33^. Fifteen patients fulfilled those criteria. The study radiologist (KT) then reviewed all imaging studies to identify patients with mixed radiographic responses to a particular line of therapy (Supplementary Data 1). An independent breast medical oncologist (PZ) reviewed the medical records for clinical/pathologic features, treatment types, and outcomes. Tumor measurements were taken for each patient on different lines of metastatic treatment, and treatment responses for individual metastases were assessed and documented for each patient and site of metastasis. The response to treatment was categorized for each metastasis, with “response” defined as a complete or partial response on each line of treatment based on RECIST v1.1 criteria^34^. Six patients demonstrated intra-patient variability in response across different sites of metastasis based on clinical and/or radiological evaluations. The remaining nine patients, who were not included in this cohort, showed the same response patterns across all sites of metastases (either all sites progressed, exhibited a combination of progression and stability, or lacked clinical/radiological information to assess the response to treatment).

### Radiological image analysis

A single radiologist (KT) reviewed the imaging studies. All available computed tomography (CT) scans of the brain, chest, abdomen, and pelvis, magnetic resonance imaging (MRI) scans of the brain, and/or PET/CT scans were reviewed at the time points obtained during each patient’s metastatic course. The patient cohort had variable numbers and types of examinations available for review, and radiographic and clinical evaluation for response were obtained according to usual clinical criteria. The first available imaging study before starting any line of systemic treatment for MBC was determined as the “baseline” examination. Baseline and response measurements were assessed and were compared before, during, and after each treatment line of therapy using RECIST v1.1 criteria^34^ for complete response, partial response, stable disease, or progressive disease, using the best response during any line of therapy.

### Sample acquisition, biospecimen processing and RNAseq gene expression data values

Samples were acquired and processed as described previously^14^. Gene expression profiles from primary and metastatic tumors for the 6-patient tumor dataset were generated by RNAseq. This dataset was partially previously published in 2018^33^, was resequenced using the rRNA depletion method, and is publicly available (dbGAP phs002429)^14^ (Supplementary Data 1). To conduct our RNAseq analysis, we utilized the entire RAP cohort, which included 101 paired primary and metastatic tumors. Of these, 20 tumors were paraffin-embedded (FFPE) and 81 were fresh frozen (FF) sequenced tumors. We also used the AURORA cohort to batch-correct the data, following the same approach previously used^14^. However, this time, we subtracted only the 101 samples from the RAP study and utilized the resulting upper-fixed quarter normalized (UQN), log2-transformed, and batch-corrected dataset for our RNAseq gene expression analysis. We also removed any normal tissue contamination from this dataset. From the larger cohort, only 6 patients (with 26 metastases) met our analysis criteria and were used for downstream analysis.

### PAM50 subtype classification

PAM50 subtype classification assignments and methodology can be found in Garcia-Recio et al.^14^ and is summarized in Supplementary Data 1.

### Gene expression signatures and differential expression analysis between responders and non-responders

For the 101 batch-corrected and adjusted for normal tissue gene expression dataset we applied a collection of 836 previously published RNA gene expression modules, representing multiple biological pathways and cell types, to all primary and metastatic tumors. Most of these modules are calculated as the median of each gene expression value present in the signature for each sample of the set used, and few of them were used as special models and calculated as it has been previously described using predefined algorithms or a single gene expression value (see methods of refs. ^21,35,36^).

After we calculated the gene signature score per sample, we performed a linear mixed model (LMM) using lmerTest^37^ and lme4^38^ R packages to identify significantly changed modules between metastatic tumor responders and non-responders. In the LMM, we included the term ‘patient’ as a random effect or confounding variable, fit = lmer (module ~ non-responder/responder + (1|patient)), using all metastatic tumors. To avoid the possible confounding factor of lymph nodes in the subsequent analysis, we subgrouped the patients into 2 groups based on whether lymph nodes were included within metastasis responders. The first group contained all different molecular subtypes, including patients with lymph nodes among responders (8 responders and 18 nonresponders), listed as “YES “in column 9 of Supplementary Data 1. After removing those patients with lymph nodes among responders, the remaining patients were Basal-like tumors (4 responders and 8 non-responders), so we refer to this second group as “Basal-like/TNBC” and they are listed as “YES “in column 10 of Supplementary Data 1. We considered differentially expressed modules when *p* value < 0.05.

### DNA copy number analysis (CNA) between responders and non-responders

Paired tumor and normal RAP whole-exome sequencing (WES) DNA fastq files were aligned to the hg38 reference genome with BWA mem and sorting, indexing, and marking of duplicate reads in the aligned BAM files was performed with Biobambam2 bamsormadup^39^. The processed BAM files were used as input for ASCAT (v3.1.2), run with the default workflow for WES data^40^. Log2 ratio copy number scores were calculated from the allele-specific ASCAT output by dividing the total copy number by the tumor ploidy estimate: ${\log }_{2}(\frac{{nAraw}+{nBraw}}{{ploidy}})$. This was input to GISTIC2 (v2.0.23), with the following parameters changed from the default: –genegistic 1 –broad 1 –brlen 0.5 –conf 0.95 –armpeel 1 –savegene 1 –ta 0.3 –td 0.3 –rx 0^41^. The gene-level GISTIC2 copy number output was collapsed to the level of 534 copy number segments, comprised of entire chromosome arm segments and other published chromosomal regions that have shown significance in pan-cancer analyses or breast cancer subtype-specific analyses^26,42–46^. The segment-level copy number scores were calculated by taking the mean GISTIC2 copy number score of all genes in each segment; the full list of 534 segments and the genes used to determine the segment-level score are found in Supplementary Data 2 of Xia et al. and exclude the two Y chromosome segments^42^.

Among the patients with mixed responses, there were 19 metastatic tumors from 4 patients with available DNA samples. These samples were used to fit 534 LMMs that were used to test the association between segment-level copy number and response, using the following equation that accounts for the patient as a random effect: segment_score ~ responder/non-responder + (1|patient). The lme4 R package^38^ was used to fit each model, and the lmerTest R package^37^ was used to calculate corresponding *p*-values using Satterthwaite’s method. All *p*-values were adjusted for multiple testing using the Benjamini-Hochberg method^47^.

### Statistics and reproducibility

The sample size was determined by the entry criteria. For LMM/linear mixed-effects model between primary and metastatic tumors, the lmerTest^37^ R package summary includes a coefficient table with estimates and *P* values for t-statistics using Satterthwaite’s method. Non-parametric, two-sided exact tests were used to make comparisons. A two-sided, unpaired Wilcoxon test was used when the dependent variable was either ordinal or continuous but not normally distributed. Exact *P* values were provided whenever possible. Clinical, RNAseq, and DNA-sequencing analyses were performed using RStudio version 2023.06.1 + 524 and R version 4.3.1 (http://cran.r-project.org). More details about each particular platform analysis are found in each methodology section. No randomization or blinding was done in the data collection or analyses. No data points were excluded from the analyses unless specified otherwise.

### Study approval

Patients gave informed consent before their death for rapid autopsy procedures, in line with the protocols established by the Office for Human Research Ethics at the University of North Carolina (UNC) at Chapel Hill and the mandates of the US Department of Health and Human Services. Paraffin-embedded or fresh-frozen tissue samples of primary breast cancers were obtained before the autopsy as part of study ID LCCC 9819 (ClinicalTrials.gov Identifier: NCT01000883). The Institutional Review Board (IRB) of the University of North Carolina approved this study.

## Supplementary information

Supplementary Figure 1 and Supplementary Data 1-6

## Data Availability

Accession numbers and data sharing are summarized in Supplementary Data 1. Briefly, all data used in this manuscript are in dbGAP (RAP study: phs002429.v1.p1, AURORA study: phs002622.v1.p1; and GEO (RAP study: RNAseq data (GSE193103), AURORA study: RNAseq data (GSE209998), All of the resources used during the studies outlined in this manuscript are summarized in Supplementary Data 1–6 and in the Methods. R packages and scripts used to analyze the data and input data are explained in the Methods. All packages are public and are freely available online. No new code or mathematical algorithms were generated from this manuscript.
