# Supplementary material for: Understanding metastasis mixed-treatment responses through genomic analyses
Source: NPJ Breast Cancer. 2025 Jan 30;11:9. doi: 10.1038/s41523-025-00724-z (PMC11782668; doi:10.1038/s41523-025-00724-z)
Supplement: Supplementary file 1 — Supplementary Figure 1 and Supplementary Data 1-6 [file 41523_2025_724_MOESM1_ESM.pdf]

Supplementary Figure 1

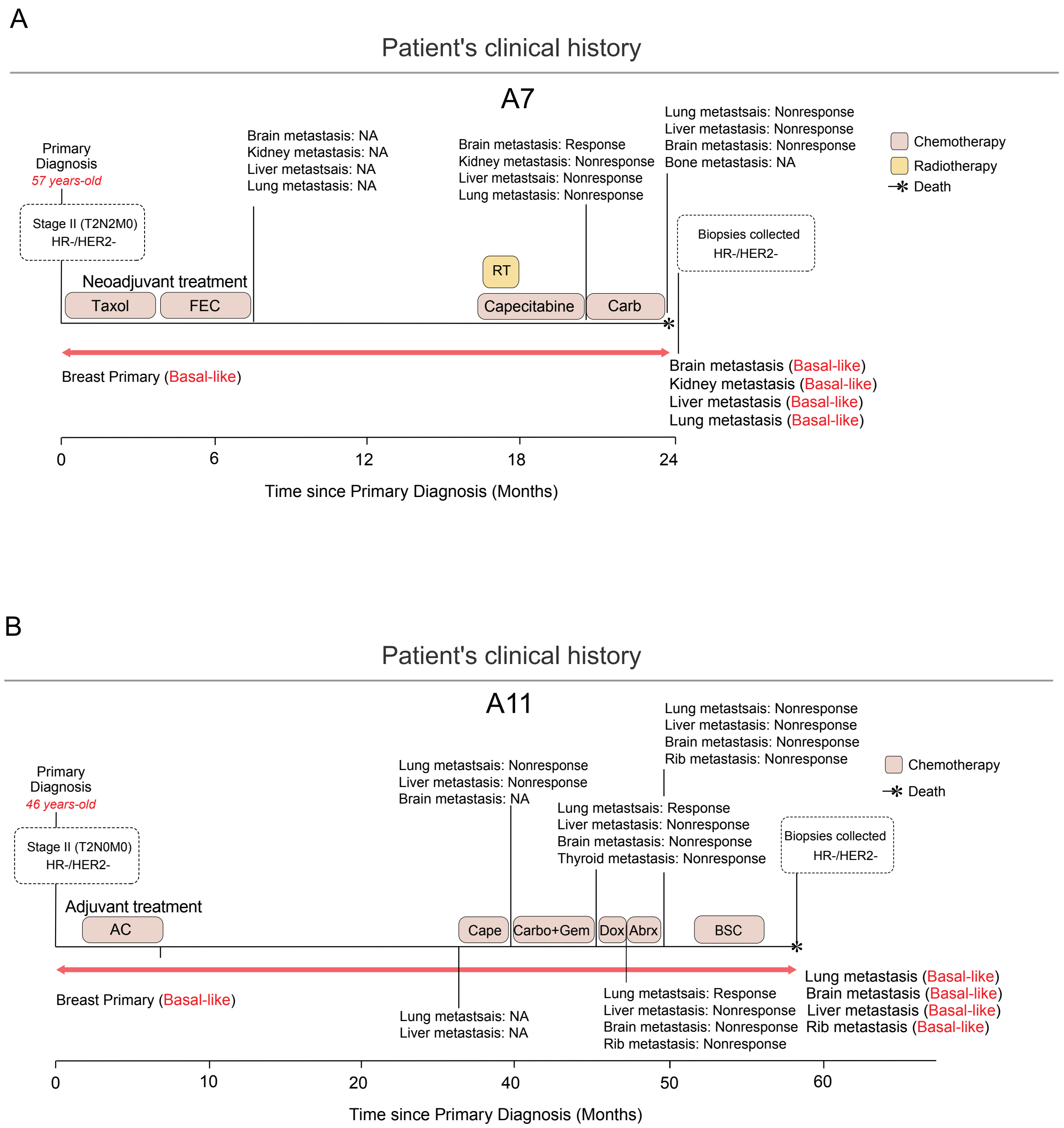

Supplementary Figure 1. Patient clinical history. Patient's clinical history of (A) A7 and (B) A11 as an example of patients exhibiting treatment response variability across their four metastases. R=Responders; NR=Nonresponders; Taxol=Taxane chemotherapy; FEC=5-Fluorouracil, Epidoxorubicin and Cyclophosphamide; Carb=Carboplatin; Cape=Capecitabine; Carbo+Gem=Carboplatin+Gemcitabine; Dox=Doxorubicin ; Abrax=Gemcitabine and nab-paclitaxel (Abraxane); BSC=Best supportive care.

Supplementary Data 1

Clinical data RNA

Patient	Sample name	Short name	Patient	Biosample_ID_dgGap (phs002429.v1.p1	Sample_GEO_accession (GSE193103	Anatomic site	Anatomic site simpl	Group 1 (includes or not Lymph nodes in res	Group 2 (NO lymph nodes in responders)	Sample Type	Tissue type	IHC	Year of Primary diagnosi	Age at Primary Diagnosi	Year of Metastatic Disease Diagnosi	Year of Death	PAM50 call	Stage at diagnosis	Time to metastasis (months)	Response.vs.Nonresponse	Response_class	Chemotherapy**	1st line Taxol+ bone RT (radiotherapy)										
Patient 1	A1.PrimT.ROi	A1.PRIM	A1	SAMN28512965	GSM5773290	Breast	Breast	YES	YES	Primary	FF	HR-/HER2-	2002	64	2002	2002	Basal	T4N2M1	0	Non-Response	Stable disease	Stable disease											
	A1.1.R.LUNG.MET.ROi	A1.MET1	A1	SAMN28512898	GSM5773291	Lung	Lung	YES	YES	Metastasis	FF	HR-/HER2-	NA	NA	NA	NA	Basal	NA	NA	Response	Partial response	Partial response											
	A1.4.SPIN.MET.ROi	A1.MET4	A1	SAMN28512919	GSM5773294	Spine	Other	YES	YES	Metastasis	FF	HR-/HER2-	NA	NA	NA	NA	Basal	NA	NA	Response	Partial response	Partial response											
	A1.AxLN.Met.ROi	A1.MET6	A1	SAMN28512904	GSM5773296	Lymph node	Lymph node	YES	YES	Metastasis	FF	HR-/HER2-	NA	NA	NA	NA	Basal	NA	NA	Non-Response	Stable disease	Stable disease											
Patient	Sample name	Short name	Patient	Biosample_ID_dgGap (phs002429.v1.p1	Sample_GEO_accession (GSE193103	Anatomic site	Anatomic site simpl	Group 1 (includes or not Lymph nodes in res	Group 2 (NO lymph nodes in responders)	Sample_Type	Tissue type	HR_HER2	Year of Primary diagnosi	Age at Primary Diagnosi	Year of Metastatic Disease Diagnosi	Year of Death	PAM50 call	Stage at diagnosis	Time to metastasis (months)	Response.vs.Nonresponse	Response_class	Chemotherapy**	1st line capecitabine + Brain RT	2nd line carboplatinum									
Patient 2	A7.PrimT020552B.ROi	A7.PRIM	A7	SAMN28512858	GSM5773308	Breast	Breast	YES	YES	Primary	FF	HR-/HER2-	2002	57	2003	2004	Basal	T2N2M0	17.03	Non-Response	Stable disease	Stable disease^	Neoadjuvant chemotheraypy Taxol + FEC (FEC=5-Fluorouracil, Epidoxorubicin and Cyclophosphamide)										
	A7.1LiverMet.ROi	A7.MET1	A7	SAMN28512902	GSM5773309	Liver	Liver	YES	YES	Metastasis	FF	HR-/HER2-	NA	NA	NA	NA	Basal	NA	NA	Non-Response	Stable disease	Stable disease											
	A7.BRAIN.MET.ROi	A7.MET2	A7	SAMN28512937	GSM5773310	Brain	Brain	YES	YES	Metastasis	FF	HR-/HER2-	NA	NA	NA	NA	Basal	NA	NA	Response	Partial response	Partial response											
	A7.KIDNEY.MET.ROi	A7.MET3	A7	SAMN28512926	GSM5773311	Kidney	Other	YES	YES	Metastasis	FF	HR-/HER2-	NA	NA	NA	NA	Basal	NA	NA	Non-Response	Progressive disease	Progressive disease											
	A7.LUNG.MET.ROi	A7.MET4	A7	SAMN28512883	GSM5773312	Lung	Lung	YES	YES	Metastasis	FF	HR-/HER2-	NA	NA	NA	NA	Basal	NA	NA	Non-Response	Stable disease	Stable disease											
Patient	Sample name	Short name	Patient	Biosample_ID_dgGap (phs002429.v1.p1	Sample_GEO_accession (GSE193103	Anatomic site	Anatomic site simpl	Group 1 (includes or not Lymph nodes in res	Group 2 (NO lymph nodes in responders)	Sample_Type	Tissue type	HR_HER2	Year of Primary diagnosi	Age at Primary Diagnosi	Year of Metastatic Disease Diagnosi	Year of Death	PAM50 call	Stage at diagnosis	Time to metastasis (months)	Response.vs.Nonresponse	Response_class	Chemotherapy**	1st line capecitabine	2nd line carboplatin+gemcitabine	3rd line liposomial doxorubicin	4th line abraxane							
Patient 3	A11.PrimT.FFPEROi	A11.PRIM	A11	SAMN28512942	GSM5773316	Breast (surgery)	Breast (surgery)	YES	YES	Primary	FFPE	HR-/HER2-	2001	46	2004	2006	Basal	T2N0M0	31.9	NA	NA+	NA+	Adjuvant anthracycline-taxol based chemotherapy										
	A11.CELEBE.MET.ROi	A11.MET2	A11	SAMN28512949	GSM5773318	Brain	Brain	YES	YES	Metastasis	FF	HR-/HER2-	NA	NA	NA	NA	Basal	NA	NA	Non-Response	Progressive disease	Progressive disease											
	A11.LIVER.MET.ROi	A11.MET3	A11	SAMN28512871	GSM5773319	Liver	Liver	YES	YES	Metastasis	FF	HR-/HER2-	NA	NA	NA	NA	Basal	NA	NA	Non-Response	Progressive disease	Progressive disease											
	A11.LUNG.MET.ROi	A11.MET4	A11	SAMN28512940	GSM5773320	Lung	Lung	YES	YES	Metastasis	FF	HR-/HER2-	NA	NA	NA	NA	Basal	NA	NA	Response	Partial response	Partial response											
	A11.RIB.MET.ROi	A11.MET5	A11	SAMN28512950	GSM5773321	Rib	Other	YES	YES	Metastasis	FF	HR-/HER2-	NA	NA	NA	NA	Basal	NA	NA	Non-Response	Progressive disease	Progressive disease											
	A11.THYRO.MET.ROi	A11.MET6	A11	SAMN28512953	GSM5773322	Thyroid	Other	YES	YES	Metastasis	FF	HR-/HER2-	NA	NA	NA	NA	LumA	NA	NA	Non-Response	Stable disease	Stable disease											
Patient	Sample name	Short name	Patient	Biosample_ID_dgGap (phs002429.v1.p1	Sample_GEO_accession (GSE193103	Anatomic site	Anatomic site simpl	Group 1 (includes or not Lymph nodes in res	Group 2 (NO lymph nodes in responders)	Sample_Type	Tissue type	HR_HER2	Year of Primary diagnosi	Age at Primary Diagnosi	Year of Metastatic Disease Diagnosi	Year of Death	PAM50 call	Stage at diagnosis	Time to metastasis (months)	Response.vs.Nonresponse	Response_class	Endocrine therapy#	Chemotherapy**	1st line fulvestrant	2nd line exemestane	3rd line exemestane	4th line capecitabin	5th vinorelbin	6th line gemcitabine	7th carboplati	8th line doxi	9th line irinoteca	10th line Taxol +bevacizumab
Patient 4	A12.PriT020076B.ROi	A12.PRIM	A12	SAMN28512857	GSM5773323	Breast (surgery)	Breast (surgery)	YES	NO	Primary	FF	HR+/HER2-	2001	64	2001	2006	LumA	T3N2M0	8.88	Non-Response	Stable disease	NA+	Stable disease^	NAC: anthracycline-taxol chemotherapy followed by mastectomy									
	A12.LIVER.MET.ROi	A12.MET2	A12	SAMN28512869	GSM5773325	Liver	Liver	YES	NO	Metastasis	FF	HR+/HER2-	NA	NA	NA	NA	LumB	NA	NA	Non-Response	Progressive disease	NA°	Progressive disease										
	A12.LUNG.MET.ROi	A12.MET4	A12	SAMN28512911	GSM5773327	Lung	Lung	YES	NO	Metastasis	FF	HR+/HER2-	NA	NA	NA	NA	LumB	NA	NA	Non-Response	Stable disease	Stable disease	Stable disease										
	A12.PLEURA.MET.ROi	A12.MET6	A12	SAMN28512907	GSM5773329	Pleura	Other	YES	NO	Metastasis	FF	HR+/HER2-	NA	NA	NA	NA	LumB	NA	NA	Non-Response	Partial response	Stable disease	Partial response										
	A12.SKULL.MET.ROi	A12.MET7	A12	SAMN28512952	GSM5773330	Skull	Other	YES	NO	Metastasis	FF	HR+/HER2-	NA	NA	NA	NA	LumB	NA	NA	Non-Response	Progressive disease	Progressive disease	Progressive disease										
	A12.LN.MET.ROi	A12.MET3	A12	SAMN28512935	GSM5773326	Lymph node	Lymph node	YES	NO	Metastasis	FF	HR+/HER2-	NA	NA	NA	NA	LumB	NA	NA	Response	Stable disease	Stable disease	Stable disease										
Patient	Sample name	Short name	Patient	Biosample_ID_dgGap (phs002429.v1.p1	Sample_GEO_accession (GSE193103	Anatomic site	Anatomic site simpl	Group 1 (includes or not Lymph nodes in res	Group 2 (NO lymph nodes in responders)	Sample_Type	Tissue type	HR_HER2	Year of Primary diagnosi	Age at Primary Diagnosi	Year of Metastatic Disease Diagnosi	Year of Death	PAM50 call	Stage at diagnosis	Time to metastasis (months)	Response.vs.Nonresponse	Response_class	Chemotherapy**	1st line Adriamycin + Taxotere	2nd line gemcitabine, carboplatin	3rd line navelbine + carboplatin	4th line telomerase inhibitor drug (GRN163L)+ paclitaxel+ bevacizumab							
Patient 5	A21.PrimT.FFPE.R0i	A21.PRIM	A21	SAMN28512918	GSM5773352	Breast (surgery)	Breast (surgery)	YES	NO	Primary	FFPE	HR+/HER2-	2005	48	2011	2012	LumA	T2N3M0	69.4	NA	NA	NA+	Adjuvant anthracycline-taxane chemotherapy										
	A21.LivMet.2.R0i	A21.MET3	A21	SAMN28512901	GSM5773355	Liver	Liver	YES	NO	Metastasis	FF	HR+/HER2-	NA	NA	NA	NA	LumA	NA	NA	Response	Partial response	Partial response											
	A21.LivMet.3.R0i	A21.MET4	A21	SAMN28512951	GSM5773356	Liver	Liver	YES	NO	Metastasis	FF	HR+/HER2-	NA	NA	NA	NA	LumA	NA	NA	Response	Partial response	Partial response											
	A21.LN.met.FFPE.R0i	A21.MET2	A21	SAMN28512877	GSM5773354	Lymph node	Lymph node	YES	NO	Metastasis	FFPE	HR+/HER2-	NA	NA	NA	NA	LumA	NA	NA	Non-Response	Progressive disease	Progressive disease											
Patient	Sample name	Short name	Patient	Biosample_ID_dgGap (phs002429.v1.p1	Sample_GEO_accession (GSE193103	Anatomic site	Anatomic site simpl	Group 1 (includes or not Lymph nodes in res	Group 2 (NO lymph nodes in responders)	Sample_Type	Tissue type	HR_HER2	Year of Primary diagnosi	Age at Primary Diagnosi	Year of Metastatic Disease Diagnosi	Year of Death	PAM50 call	Stage at diagnosis	Time to metastasis (months)	Response.vs.Nonresponse	Response_class	Chemotherapy**	1st line paclitaxel and bevacizumab	2nd line abraxane + tigatuzumab	3rd line capecitabine	4rd line gemcitabine	5th line doxil						
Patient 6	A23.PTcore2009.FFPER0i	A23.PRIM	A23	SAMN28512847	GSM5773357	Breast (surgery)	Breast (surgery)	YES	NO	Primary	FFPE	HR-/HER2-	2009	47	2011	2012	Basal	T4N2M0	18.65	NA	NA	Stable disease^	Neoadjuvant anthracycline-taxol chemotherapy										
	A23.BrainMet.2.R0i	A23.MET2	A23	SAMN28512928	GSM5773359	Brain	Brain	YES	NO	Metastasis	FF	HR-/HER2-	NA	NA	NA	NA	Basal	NA	NA	Non-Response	Progressive disease	Progressive disease											
	A23.BrainMet.3.R0i	A23.MET3	A23	SAMN28512891	GSM5773360	Brain	Brain	YES	NO	Metastasis	FF	HR-/HER2-	NA	NA	NA	NA	Basal	NA	NA	Non-Response	Progressive disease	Progressive disease											
	A23.BrainMet.R0i	A23.MET4	A23	SAMN28512862	GSM5773361	Brain	Brain	YES	NO	Metastasis	FF	HR-/HER2-	NA	NA	NA	NA	Basal	NA	NA	Non-Response	Progressive disease	Progressive disease											
	A23.LN.Met.2010postT.FFPER0i	A23.MET5	A23	SAMN28512933	GSM5773362	Lymph node	Lymph node	YES	NO	Metastasis	FFPE	HR-/HER2-	NA	NA	NA	NA	Basal	NA	NA	Non-Response	Stable disease	Stable disease											
	A23.LN.Met.R0i	A23.MET6	A23	SAMN28512934	GSM5773363	Lymph node	Lymph node	YES	NO	Metastasis	FF	HR-/HER2-	NA	NA	NA	NA	LumA	NA	NA	Response	Partial response	Partial response											
	A23.PleuraMet.R0i	A23.MET7	A23	SAMN28512900	GSM5773364	Pleura	Other	YES	NO	Metastasis	FF	HR-/HER2-	NA	NA	NA	NA	Basal	NA	NA	Non-Response	Progressive disease	Progressive disease											

* Year of diagnosis of the first metastasis; this does not necessarily correspond to the metastases collected for gene expression analysis described in this table.
** In the columns to the right, we report the specific chemotherapeutic drugs received for each line of metastatic treatment.
# In the columns to the right, we report the specific endorine therapy received for each line of metastatic treatment.
° Not yet present during Endocrine therapy
^ Response to neoadjuvant treatment at surgery

Supplementary Data 2

Clinical data DNA

Sample name	Response.vs.Nonresponse	Sample Type	Patient	Anatomic site	Tissue type	IHC	PAM50 call
A7-PRIMT020552B	Non-Response	Primary	A7	Breast	FF	HR-/HER2-	Basal
A7-BRAIN-Met	Response	Metastasis	A7	Brain	FF	HR-/HER2-	Basal
A7-KIDNYMET	Non-Response	Metastasis	A7	Kidney	FF	HR-/HER2-	Basal
A7-LIV-MET1	Non-Response	Metastasis	A7	Liver	FF	HR-/HER2-	Basal
A7-LUNG-MET1	Non-Response	Metastasis	A7	Lung	FF	HR-/HER2-	Basal
A11-PT-FFPE	NA	Primary	A11	Breast	FFPE	HR-/HER2-	Basal
A11-CELEB-METt	Non-Response	Metastasis	A11	Brain	FF	HR-/HER2-	Basal
A11-LIV-MET	Non-Response	Metastasis	A11	Liver	FF	HR-/HER2-	Basal
A11-LUNG-MET	Response	Metastasis	A11	Lung	FF	HR-/HER2-	Basal
A11-RIB-MET	Non-Response	Metastasis	A11	Rib	FF	HR-/HER2-	Basal
A12-PRIMT020076B	Non-Response	Primary	A12	Breast	FF	HR+/HER2-	LumA
A12-LIV-MET	Non-Response	Metastasis	A12	Liver	FF	HR+/HER2-	LumB
A12-LN-Met-subcarinal	Response	Metastasis	A12	LN	FF	HR+/HER2-	LumB
A12-PleuraMet	Non-Response	Metastasis	A12	Pleura	FF	HR+/HER2-	LumB
A12-RLL-MET	Non-Response	Metastasis	A12	Lung	FF	HR+/HER2-	LumB
A12-SkullMet	Non-Response	Metastasis	A13	Skull	FF	HR+/HER2-	LumB
A23-PTcore-2009-FFPE	NA	Primary	A23	Breast	FFPE	HR-/HER2-	Basal
A23-BrainMet	Non-Response	Metastasis	A23	Brain	FF	HR-/HER2-	Basal
A23-BrainMet-2	Non-Response	Metastasis	A23	Brain	FF	HR-/HER2-	Basal
A23-BrainMet-3	Non-Response	Metastasis	A23	Brain	FF	HR-/HER2-	Basal
A23-LN-Met	Response	Metastasis	A23	LN	FF	HR-/HER2-	LumA
A23-LN-Met-2010postT-FFPE	Non-Response	Metastasis	A23	LN	FFPE	HR-/HER2-	Basal
A23-PleuraMet	Non-Response	Metastasis	A23	Pleura	FF	HR-/HER2-	Basal

Supplementary Data 3

Linear mixed model - RNA - All samples

Signatures	B_value	T.value	dir	P.adj	P.value
KRAS_amplicon_Genome.Biology.2007_PMID.17493263	-1.265236	-4.0287413	Negative	0.87779092	0.00136715
MKRAS_amplicon_BMC.Med.Genomics.2011_PMID.21214954	-1.0848595	-3.2581681	Negative	0.87779092	0.00596289
GSEA_RESPONSE_TO_ANDROGEN_UP_NELSON	-0.80534	-3.2362742	Negative	0.87779092	0.00618154
MUnknown_14_BMC.Med.Genomics.2011_PMID.21214954	-1.0288337	-3.156562	Negative	0.87779092	0.00730089
CDKN2A_Single_Gene	0.83306434	3.03825894	Positive	0.87779092	0.0093472
IMMUNE_Bindea_Cell_CD8_T_cells_Immunity.2013_PMID.24138885	-1.2995224	-2.7894125	Negative	0.87779092	0.01456983
Duke_Module07_glucosedepletion_Mike_PMID.20335537	-0.7908663	-2.7981667	Negative	0.87779092	0.01474868
LOBULAR_TCGA_SUBTYPE_Immune_related_Cell.2015_PMID.26451490	-0.8280595	-2.7232089	Negative	0.87779092	0.01573955
KRAS_Single_Gene	-0.8927484	-2.6798448	Negative	0.87779092	0.01847786
XBP1_Signature_Nature.2014_PMID.24670641	-0.8569227	-2.6346881	Negative	0.87779092	0.02003643
MUnknown_35_BMC.Med.Genomics.2011_PMID.21214954	0.85702218	2.52997873	Positive	0.87779092	0.02482683
MET_DOWN_Significant_Genes_LOW_BASALS_2_Genes_JCI.2018_PMID.29480	-0.7007461	-2.5065942	Negative	0.87779092	0.02575274
MET_DOWN_RNA_Seq_Significant_Genes_JCI.2018_PMID.29480819	-0.7422796	-2.4529438	Negative	0.87779092	0.02829962
Pcorr_Carcinoid_JMD.2013_PMID.23701907	-0.7846137	-2.4245237	Negative	0.87779092	0.03019891
Duke_Module19_src_Mike_PMID.20335537	-0.9703166	-2.4011935	Negative	0.87779092	0.03119451
GSEA_GP8_FOXO_stemness.r.0.931_TTGTTT_V.FOXO4_01	-0.640468	-2.4066216	Negative	0.87779092	0.03135899
Melanoma_Scorr_MITF.low_Cell.2015_PMID.26091043	-0.722013	-2.3962186	Negative	0.87779092	0.03159962
TRIM29_Single_Gene	0.5296036	2.39095155	Positive	0.87779092	0.03213302
ERBB2_Single_Gene	0.29845803	2.35086044	Positive	0.87779092	0.03381334
TCGA_Tfh_cells_Immunity.2018_PMID._29628290	-0.7998017	-2.3593571	Negative	0.87779092	0.03434931
IMMUNE_Bindea_Cell_TFH_Immunity.2013_PMID.24138885	-0.7998017	-2.3593571	Negative	0.87779092	0.03434931
YALE_PIK3CA_Pathway_Ann.Oncol.2017_PMID.28177460	-0.8588229	-2.319156	Negative	0.87779092	0.03525676
MM_Green10_BMC.Med.Genomics.2011_PMID.21214954	-0.8558885	-2.3071986	Negative	0.87779092	0.03739869
MM_Green15_BMC.Med.Genomics.2011_PMID.21214954	-0.7276469	-2.1881297	Negative	0.87779092	0.04384327
IMMUNE_Bindea_Cell_Tcm_Immunity.2013_PMID.24138885	-0.7176624	-2.1597213	Negative	0.87779092	0.04960261
X8p22_Amplicon_BMC.Med.Genomics.2011_PMID.21214954	-0.9744909	-2.0804958	Negative	0.87779092	0.0566101
GSEA_BIOCARTA_RAS_PATHWAY	-0.8270338	-2.0407282	Negative	0.87779092	0.05898928
Melanoma_Scorr_Immune_Cell.2015_PMID.26091043	-0.8138542	-2.0324366	Negative	0.87779092	0.06136353
GSEA_GP3_Tumor_suppressing_miRNA_targets.r.0.940_TGCTTTG.MIR_330	-0.6410947	-2.0178551	Negative	0.87779092	0.06410505
Bcells_Centrocyte_J.Clin.Oncol.2015_PMID.25800755	-1.102247	-1.9850056	Negative	0.87779092	0.06489095
ACTIVATED_LUNG_MSC_SIGNATURE_Nat.Cell.Biol.2019_PMID.31263265	-0.728486	-1.9771729	Negative	0.87779092	0.06765313
New.Immune_Charoentong_Eosinophil_CellRep.2017_PMID.28052254	-1.0860089	-1.9636849	Negative	0.87779092	0.0679634
Duke_Module06_er_Mike_PMID.20335537	-0.3361803	-1.9732461	Negative	0.87779092	0.06953134
Taube_EMT_up_PNAS.2010_PMID.20713713	-0.7222386	-1.9499006	Negative	0.87779092	0.07078005
Shipitsin_CD44_A_Cancer.Cell.2007_PMID.17349583	-0.4053791	-1.9605683	Negative	0.87779092	0.07091039
Duke_Module18_ras_Mike_PMID.20335537	-0.8079991	-1.91936	Negative	0.87779092	0.07336577
Inflammatory_Breast_Cancer_79_IBC_CCR.2013_PMID.23396049	-0.7398223	-1.8992261	Negative	0.87779092	0.07571705
GSEA_MTOR_PATHWAY_BIOCARTA	-0.6996293	-1.910037	Negative	0.87779092	0.077704
Pcorr_Hypoxia_Low_Correlation_PLoS.Med.2006_PMID.16417408	0.81413032	1.88566163	Positive	0.87779092	0.07792464
FOS_JUN_Cluster_BMC.Med.Genomics.2011_PMID.21214954	-0.6636115	-1.8792238	Negative	0.87779092	0.07855437
TCGA.BRCA.1198_immune_FOS_JUN_IL6_JCI.2020_PMID.32573490	-0.6233004	-1.8858572	Negative	0.87779092	0.07914525
aMaSC_Shehata_BCR.2015_PMID.25575446	-0.5361792	-1.8953288	Negative	0.87779092	0.07950484
HGF_up_BCR.2013_PMID.24025166	-0.9656094	-1.8762135	Negative	0.87779092	0.08020253
Pcorr_Hypoxia_High_Correlation_PLoS.Med.2006_PMID.16417408	-0.7700162	-1.8481351	Negative	0.87779092	0.0831525
UNC_Scorr_Her2_Correlation_JCO.2009_PMID.19204204	0.6238312	1.83773582	Positive	0.87779092	0.0853998
New.Immune_Charoentong_Neutrophil_CellRep.2017_PMID.28052254	-0.6108854	-1.8281825	Negative	0.87779092	0.08622795
MM_Green24_BMC.Med.Genomics.2011_PMID.21214954	-0.7681772	-1.8424439	Negative	0.87779092	0.08711042
GRB7_Single_Gene	0.61010877	1.82442192	Positive	0.87779092	0.08899992
EMT_DOWN_Weingberg_PNAS.2010_PMID.20713713	0.68126268	1.81937555	Positive	0.87779092	0.08929823
GSEA_GP3_Tumor_suppressing_miRNA_targets.r.0.952_DACOSTA_UV_RESPON	-0.6674341	-1.7998635	Negative	0.87779092	0.0940515
TCGA.BRCA.1198_UNKNOWN6_JCI.2020_PMID.32573490	-0.4209298	-1.7836144	Negative	0.87779092	0.09457753
Lung.WNT.DiMeo_Cancer.Res.2009_PMID.19549913	-0.7491725	-1.7736739	Negative	0.87779092	0.09514792
Early_IRS_1_PLoS.One.2016_PMID.26991655	-0.809572	-1.7819961	Negative	0.87779092	0.0955858
MM_NeuPyMT_1pFDR_UP_Genome.Biology.2007_PMID.17493263	-0.6013885	-1.791116	Negative	0.87779092	0.09598637
New.Immune_Charoentong_CD56bright_natural_killer_cell_CellRep.2017_PMID	-0.7140508	-1.7653728	Negative	0.87779092	0.09657519
HS_Red2_BMC.Med.Genomics.2011_PMID.21214954	-0.5481669	-1.7792795	Negative	0.87779092	0.09776348
TCGA.BRCA.1198_NORMAL2_JCI.2020_PMID.32573490	-0.4339895	-1.7712839	Negative	0.87779092	0.09789129
MUnknown_6_BMC.Med.Genomics.2011_PMID.21214954	-0.8597727	-1.758899	Negative	0.87779092	0.09996556
Pcorr_Squamous_Cell_Carcinoma_JMD.2013_PMID.23701907	0.63712934	1.76221069	Positive	0.87779092	0.10084176
Inflammatory_Breast_Cancer_491_nIBC_CCR.2013_PMID.23396049	-0.5244705	-1.7464946	Negative	0.87779092	0.10337664
MM_Red2_BMC.Med.Genomics.2011_PMID.21214954	-0.6364865	-1.7439864	Negative	0.87779092	0.10395061
Spike2012_fMaSC_PMID.25575446	0.74287507	1.73435272	Positive	0.87779092	0.10502559
Extensive_Residual_Diesase_ER.Neg_54_JAMA.2011_PMID.21558518	-0.7270588	-1.7197647	Negative	0.87779092	0.10549016
MUnknown_19_BMC.Med.Genomics.2011_PMID.21214954	0.69995486	1.71478158	Positive	0.87779092	0.10625973
EXTENDED_B_CELL_signatures_Garber_Cell.Mol.Gastroenterol.Hepatol.2017_PI	-0.7745029	-1.7157075	Negative	0.87779092	0.10748581
Early_Response_ER.Neg_27_JAMA.2011_PMID.21558518	0.44636615	1.69524141	Positive	0.87779092	0.10939901
New.Immune_Charoentong_Monocyte_CellRep.2017_PMID.28052254	-0.7942689	-1.6975091	Negative	0.87779092	0.10941561
Duke_Module20_stat3_Mike_PMID.20335537	-0.4854947	-1.6993612	Negative	0.87779092	0.10946637
Mature_Luminal_Down_Nat.Med.2009_PMID.19648928	-0.510286	-1.6987236	Negative	0.87779092	0.11035077
MRibosomal_BMC.Med.Genomics.2011_PMID.21214954	-0.7745475	-1.7026135	Negative	0.87779092	0.11051976
XBP1_Single_Gene	-0.4926546	-1.6976884	Negative	0.87779092	0.11155837
MCF7.E2.repressed.genes_JCO.2006_PMID.16505416	-0.3903863	-1.7044146	Negative	0.87779092	0.11180297
MM_Green9_BMC.Med.Genomics.2011_PMID.21214954	0.77505652	1.68403789	Positive	0.87779092	0.1140388
IMMUNE_Bindea_Cell_Macrophages_Immunity.2013_PMID.24138885	-0.794377	-1.6719514	Negative	0.87779092	0.11494789
Claudin_Low_29_Cancer.Res.2009_PMID.19435916	0.79612712	1.66478533	Positive	0.87779092	0.11572681
MUnknown_4_BMC.Med.Genomics.2011_PMID.21214954	0.71211215	1.65520993	Positive	0.87779092	0.11736123
Mouse_Human_ImmuneProfiles_SHAY_M_H_Induced_in_GN_PNAS.2013_PMID	-0.7616897	-1.6590553	Negative	0.87779092	0.11783383
New.Immune_Charoentong_Mast_cell_CellRep.2017_PMID.28052254	-0.8028891	-1.6567854	Negative	0.87779092	0.11784029
RB1_Single_Gene	-0.4995717	-1.6685512	Negative	0.87779092	0.11850012
Ribosomal_Cluster_BMC.Med.Genomics.2011_PMID.21214954	-0.8006159	-1.6586467	Negative	0.87779092	0.11886449
New.Immune_Charoentong_Central_memory_CD8_T_cell_CellRep.2017_PMID.	-0.5354556	-1.6551336	Negative	0.87779092	0.1196011
IMMUNE_Bindea_Cell_Normal_mucosa_Immunity.2013_PMID.24138885	-0.8246246	-1.643819	Negative	0.87779092	0.11971608
New.Immune_ImSig.TranslationPathway_CancerImmunolRes.2018_PMID.3026	-0.7784552	-1.6448973	Negative	0.87779092	0.12162297
IMMUNE_Bindea_Cell_T_helper_cells_Immunity.2013_PMID.24138885	-0.7157348	-1.6425792	Negative	0.87779092	0.12178137
GSEA_MYC_amplified_chr8q24	0.67712268	1.64101608	Positive	0.87779092	0.12215294
Stingl_Down_Basal_High_Nat.Cell.Biol.2014_PMID.25173976	0.38740283	1.64702866	Positive	0.87779092	0.122847
MUnknown_31_BMC.Med.Genomics.2011_PMID.21214954	-0.6557792	-1.6368488	Negative	0.87779092	0.12432451

Signatures_UP-DOWN_p<0.05	Renamed for figure purposes and simplicity	B_value	T.value	dir	P.adj	P.value
CDKN2A_Single_Gene	CDKN2A_Single_Gene	1.13121439	4.4217776	Positive	0.21972077	0.00026282
MUnknown_19_BMC.Med.Genomics.2011_PMID.21214954	GO-Ion_transport/Cell_junction_organization	1.08173907	2.93200878	Positive	0.74634799	0.0074413
MUnknown_35_BMC.Med.Genomics.2011_PMID.21214954	No_GO	0.75827974	2.90428622	Positive	0.74634799	0.00892761
MM_Red3_BMC.Med.Genomics.2011_PMID.21214954	GO-Receptor_binding/Cell_signalling/cell_adhesior	0.91604874	2.30374426	Positive	0.91958868	0.03072985
MRE_Score_Breast.Cancer.Res.Treat_2015_PMID.26109344	MRE_Score	0.80088611	2.19600826	Positive	0.91958868	0.03799519
T_regulatory_cell_2gene_PMID.31942077	T_regulatory_cell_2gene	0.78760687	2.1649779	Positive	0.91958868	0.04190688
IMMUNE_Bindea_Cell_SW480_cancer_cells_Immunity.2013_PMID.24138885	IMMUNE_Bindea_Cell_SW480	0.81889193	2.14779177	Positive	0.91958868	0.04356868
ADM_S100A10_A110NDGR1_Cluster_BMC.Med.Genomics.2011_PMID.2121495	ADM_S100A10_A110NDGR1_Cluster	0.51068034	2.14822105	Positive	0.91958868	0.04377625
MK14_K17_BMC.Med.Genomics.2011_PMID.21214954	MK14_K17	0.52128271	2.09781496	Positive	0.91958868	0.0482273
KRAS_amplicon_Genome.Biology.2007_PMID.17493263	KRAS_amplicon	-0.9865633	-3.4738218	Negative	0.74634799	0.00240243
MUnknown_14_BMC.Med.Genomics.2011_PMID.21214954	GO-Carbohydrate Transport/Protein Localization	-0.8709889	-3.2424221	Negative	0.74634799	0.00408068
IMMUNE_Bindea_Cell_Tcm_Immunity.2013_PMID.24138885	IMMUNE_Bindea_Cell_Tcm	-0.8367162	-3.2452552	Negative	0.74634799	0.00410696
MET_DOWN_Significant_Genes_LOW_BASALS_2_Genes_JCI.2018_PMID.294808	MET_DOWN_Significant_Genes_LOW_BASALS	-0.761594	-3.1239768	Negative	0.74634799	0.00533948
MET_DOWN_RNA_Seq_Significant_Genes_JCI.2018_PMID.29480819	MET_DOWN_RNA_Seq_Significant_Genes	-0.7191607	-3.0536906	Negative	0.74634799	0.00618111
MKRAS_amplicon_BMC.Med.Genomics.2011_PMID.21214954	MKRAS_amplicon	-0.85809	-2.9547192	Negative	0.74634799	0.00778774
LOBULAR_TCGA_SUBTYPE_Immune_related_Cell.2015_PMID.26451490	LOBULAR_TCGA_SUBTYPE_Immune_related	-0.7997569	-2.872227	Negative	0.74634799	0.00871151
New.Immune_Charoentong_Eosinophil_CellRep.2017_PMID.28052254	New.Immune_Charoentong_Eosinophil	-1.0331138	-2.6609397	Negative	0.91958868	0.01386425
MUnknown_28_BMC.Med.Genomics.2011_PMID.21214954	GO-Immune-related genes/Interferon	-0.8127208	-2.4873152	Negative	0.91958868	0.02146904
GSEA_GP8_FOXO_stemness.r.0.931_TTGTTT_V.FOXO4_01	GSEA_GP8_FOXO_stemness	-0.5556793	-2.4421475	Negative	0.91958868	0.02420213
Duke_Module07_glucosedepletion_Mike_PMID.20335537	Duke_Module07_glucosedepletion	-0.6049939	-2.4286116	Negative	0.91958868	0.02468408
Early_IRS_1_PLoS.One.2016_PMID.26991655	Early_IRS_1	-0.9072445	-2.4074287	Negative	0.91958868	0.02487856
Pcorr_Carcinoid_JMD.2013_PMID.23701907	Pcorr_Carcinoid	-0.6693516	-2.3664777	Negative	0.91958868	0.02809044
X5Q_Breast.Cancer.Res.Treat.2012_PMID.22048815	5Q_Basal	-0.6292283	-2.3553308	Negative	0.91958868	0.02870532
KRAS_Single_Gene	KRAS_Single_Gene	-0.6473357	-2.3073754	Negative	0.91958868	0.03173546
PDGFRA_Single_Gene	PDGFRA_Single_Gene	-0.8323754	-2.172584	Negative	0.91958868	0.03991292
PARP_Sensitivity_Signature_NEGATIVE_Sci.Adv.2017_PMID.28439535	PARP_Sensitivity_Signature_NEGATIVE	-0.6347411	-2.1988208	Negative	0.91958868	0.03996414
IMMUNE_Bindea_Cell_CD8_T_cells_Immunity.2013_PMID.24138885	IMMUNE_Bindea_Cell_CD8_T_cells	-0.803392	-2.1087148	Negative	0.91958868	0.04699828

IntClust_Deletion.50.Better.than_Genome.Biol.2014_PMID.25164602	0.55232615	1.62618848	Positive	0.87779092	0.12618423
ACTIVATED_LUNG_NEUTROPHIL_SIGNATURE_Nat.Cell.Biol.2019_PMID.312632	-0.8610957	-1.6192776	Negative	0.87779092	0.12738044
TCGA_TGFB_score_21050467_Immunity.2018_PMID._29628290	-0.6624895	-1.604143	Negative	0.87779092	0.12897429
HS_Red17_BMC.Med.Genomics.2011_PMID.21214954	-0.7102021	-1.6029729	Negative	0.87779092	0.13096182
HS_Green11_BMC.Med.Genomics.2011_PMID.21214954	-0.7135556	-1.5975724	Negative	0.87779092	0.13244548
Excellent_Pathologic_Response_ER.Neg_55_JAMA.2011_PMID.21558518	-0.7761062	-1.5961389	Negative	0.87779092	0.13244765
Her2_Basal_JCI.2020_PMID.32573490	0.24370247	1.598213	Positive	0.87779092	0.13309372
HS_Green1_BMC.Med.Genomics.2011_PMID.21214954	-0.5532754	-1.5937074	Negative	0.87779092	0.13334882
TCGA.BRCA.1198_FGFR4_EGF_JCI.2020_PMID.32573490	0.64962154	1.57675746	Positive	0.87779092	0.13441545
GSEA_GP6_Squamous_differentiation_development.r.0.902_RICKMAN_TUMOR	-0.3966154	-1.5738322	Negative	0.87779092	0.13589302
GENE_PANEL_TRUSEQ_40_ILLUMINA	-0.6578284	-1.5728968	Negative	0.87779092	0.13855972
LUMINAL_Cluster_BMC.Med.Genomics.2011_PMID.21214954	-0.468049	-1.5682558	Negative	0.87779092	0.13925604
Murat_G07_J.Clin.Oncol.2008_PMID.18565887	-0.7147262	-1.5550883	Negative	0.87779092	0.13948115
Duke_Module05_egfr_Mike_PMID.20335537	-0.461553	-1.5668343	Negative	0.87779092	0.13954539
Unknown_10_BMC.Med.Genomics.2011_PMID.21214954	-0.520137	-1.5513754	Negative	0.87779092	0.14147484
IMMUNE_Bindea_Cell_NK_CD56dim_cells_Immunity.2013_PMID.24138885	0.46223014	1.5553013	Positive	0.87779092	0.14299619
X13q14_Amplicon_BMC.Med.Genomics.2011_PMID.21214954	-0.7433892	-1.552299	Negative	0.87779092	0.14332997
Duke_Module16_pi3k_Mike_PMID.20335537	-0.6978073	-1.547306	Negative	0.87779092	0.14414726
Mouse_Human_ImmuneProfiles_SHAY_M_H_Induced_in_MO_PNAS.2013_PMI	-0.7373687	-1.5418504	Negative	0.87779092	0.1442371
MNADH_CYTochrome_BMC.Med.Genomics.2011_PMID.21214954	-0.5830271	-1.5440498	Negative	0.87779092	0.14447646
IMMUNE_Bindea_Cell_Blood_vessels_Immunity.2013_PMID.24138885	-0.8043257	-1.5328738	Negative	0.87779092	0.14532365
PARP_sensitivity_MDACC_NPJ.Syst.Biol.Appl._2017_PMID.28649435	-0.7599418	-1.535923	Negative	0.87779092	0.14715949
New.Immune_Charoentong_Immature_dendritic_cell_CellRep.2017_PMID.2805	-0.5946483	-1.5313718	Negative	0.87779092	0.14722123
BASAL_Cluster_BMC.Med.Genomics.2011_PMID.21214954	0.37423263	1.53582536	Positive	0.87779092	0.14746023
MK14_K17_BMC.Med.Genomics.2011_PMID.21214954	0.51924349	1.52894053	Positive	0.87779092	0.14826167
MM_Red20_BMC.Med.Genomics.2011_PMID.21214954	0.47048428	1.52218009	Positive	0.87779092	0.15076608
IMMUNE_Bindea_Cell_SW480_cancer_cells_Immunity.2013_PMID.24138885	0.77090245	1.50583013	Positive	0.87779092	0.15400886
MUnknown_18_BMC.Med.Genomics.2011_PMID.21214954	0.47321644	1.50144578	Positive	0.87779092	0.15421753
MM_Red23_BMC.Med.Genomics.2011_PMID.21214954	-0.5819622	-1.5033239	Negative	0.87779092	0.15574473
Murat_G18_J.Clin.Oncol.2008_PMID.18565887	0.71576698	1.49258904	Positive	0.87779092	0.15679181
MUnknown_13_BMC.Med.Genomics.2011_PMID.21214954	-0.6937329	-1.489712	Negative	0.87779092	0.15796332
MM_Red3_BMC.Med.Genomics.2011_PMID.21214954	0.75350368	1.48305593	Positive	0.87779092	0.15902598
aStr_Shehata_BCR.2015_PMID.25575446	-0.6118738	-1.475788	Negative	0.87779092	0.15940903
Euclidean_Distance_2_CLOW_Euclidean.Distance_BCR.2010_PMID.20813035	0.64495615	1.47473446	Positive	0.87779092	0.15968893
ESR1_Single_Gene	-0.2785491	-1.4769532	Negative	0.87779092	0.1609859
HS_Red24_BMC.Med.Genomics.2011_PMID.21214954	-0.5914846	-1.4654179	Negative	0.87779092	0.16218194
X11q13_Amplicon_BMC.Med.Genomics.2011_PMID.21214954	-0.4338597	-1.4756203	Negative	0.87779092	0.16319069
Pcorr_secretory_CCR.2010_PMID.20643781	-0.6111563	-1.4608648	Negative	0.87779092	0.16488494
GSEA_GP9_Cell_cell_adhesion.r.960_PerouLab_MClaudin_Cluster_BMC_Med_G	0.70511346	1.45870025	Positive	0.87779092	0.1656962
HS_Red8_BMC.Med.Genomics.2011_PMID.21214954	0.33273958	1.46479847	Positive	0.87779092	0.16597219
TCGA.BRCA.1198_immune_CD34_TIE1_JCI.2020_PMID.32573490	-0.77192	-1.4502233	Negative	0.87779092	0.1663168
Fibroblast_Cluster_BMC.Med.Genomics.2011_PMID.21214954	-0.5959331	-1.4395752	Negative	0.87779092	0.16926587
MUnknown_12_BMC.Med.Genomics.2011_PMID.21214954	-0.6784905	-1.4450068	Negative	0.87779092	0.16983761
GSEA_GP16_Protein_kinase_signailng_MAPKs.r.0.893_INTRACELLULAR_SIGNAL	-0.6410888	-1.4306672	Negative	0.87779092	0.17176591
HS_Red21_BMC.Med.Genomics.2011_PMID.21214954	-0.5822546	-1.424575	Negative	0.87779092	0.17349308
New.Immune_ImmLandscape_IFN_3.PMID.24516633	0.504178	1.43076126	Positive	0.87779092	0.17529182
TCGA_Module3_IFN_score_Immunity.2018_PMID._29628290	0.504178	1.43076126	Positive	0.87779092	0.17529182
TCGA.BRCA.1198_BASAL_JCI.2020_PMID.32573490	0.32046596	1.42833631	Positive	0.87779092	0.17608644
MFGFR2_BMC.Med.Genomics.2011_PMID.21214954	-0.6193276	-1.4215418	Negative	0.87779092	0.17633191
GSEA_GP15_EGF_signailng.r.0.936_NAGASHIMA_EGF_SIGNALING_UP	-0.5247978	-1.4137444	Negative	0.87779092	0.17689757
MDSC_tumor_MO_Schlecker_J.Immunol.2012_PMID.23152559	-0.5361209	-1.4185699	Negative	0.87779092	0.1773561
X16q23_Amplicon_BMC.Med.Genomics.2011_PMID.21214954	-0.5155432	-1.4190946	Negative	0.87779092	0.1775477
GSEA_GP4_MES_ECM.r.0.954_PerouLab_HS_Red7_BMC_Med_Genomics_2011	-0.6255692	-1.4056955	Negative	0.87779092	0.17925872
CIBERSORT_Eosinophils_Nat.Methods.2015_PMID.25822800	-0.7214783	-1.3991696	Negative	0.87779092	0.18167984
MRE_Score_Breast.Cancer.Res.Treat_2015_PMID.26109344	0.55370247	1.39716201	Positive	0.87779092	0.18283659
MM_Green4_BMC.Med.Genomics.2011_PMID.21214954	-0.6094316	-1.3977109	Negative	0.87779092	0.18410216
Shipitsin_CD44_B_Cancer.Cell.2007_PMID.17349583	-0.6128606	-1.3804861	Negative	0.87779092	0.18727539
MM_Red12_BMC.Med.Genomics.2011_PMID.21214954	0.73138341	1.37724207	Positive	0.87779092	0.18780176
Tcell_EXH_Memory_CD8_T_cell_a_vs_Naive_CD8_T_cell_Metagene_2_Science.	-0.6947477	-1.3735854	Negative	0.87779092	0.18851179
HS_Red22_BMC.Med.Genomics.2011_PMID.21214954	-0.6040813	-1.3815231	Negative	0.87779092	0.18862522
New.Immune_Charoentong_Central_memory_CD4_T_cell_CellRep.2017_PMID.	-0.6575008	-1.3729037	Negative	0.87779092	0.1887195
GSEA_BIOCARTA_PTEN_PATHWAY	-0.6979373	-1.3731545	Negative	0.87779092	0.1901022
MDACC.FNA.2_J.Clin.Oncol.2010_PMID.20805453	-0.6426592	-1.3648988	Negative	0.87779092	0.19117238
Neutrophils_MCP_PMID.31942075_PMID.31942077	-0.5642073	-1.3658191	Negative	0.87779092	0.19137042
Stingl_Down_CLOW_High_Nat.Cell.Biol.2014_PMID.25173976	-0.6970886	-1.3593394	Negative	0.87779092	0.19335547
MM_Green5_BMC.Med.Genomics.2011_PMID.21214954	-0.6574931	-1.3560692	Negative	0.87779092	0.19390768
aMaSC_Prat_BCR.2015_PMID.25575446	-0.4898282	-1.3543279	Negative	0.87779092	0.19522387
FGFR4_Single_Gene	0.53338384	1.35336679	Positive	0.87779092	0.19540078
UNC_Differentiation.Score_Model_BCR.2010_PMID.20813035	0.3045209	1.35357539	Positive	0.87779092	0.1968388
Stromal_Down_Nat.Med.2009_PMID.19648928	0.54982952	1.34836891	Positive	0.87779092	0.19742087
Early_Relapse_ER.Pos_33_JAMA.2011_PMID.21558518	0.53583602	1.34865714	Positive	0.87779092	0.19772852
PARP_Sensitivity_Signature_NEGATIVE_Sci.Adv.2017_PMID.28439535	-0.5083237	-1.3489819	Negative	0.87779092	0.19946287
HS_Green9_BMC.Med.Genomics.2011_PMID.21214954	-0.572845	-1.337132	Negative	0.87779092	0.20029271
X15q25_Amplicon_BMC.Med.Genomics.2011_PMID.21214954	-0.5217471	-1.3449917	Negative	0.87779092	0.20087008
MProtocadherin_BMC.Med.Genomics.2011_PMID.21214954	-0.6572146	-1.3330108	Negative	0.87779092	0.20119942
MM_Erbb2.like_Genome.Biol.2013_PMID.24220145	0.40595148	1.33584427	Positive	0.87779092	0.20293392
Lim2010_MatureLum_Adam_PMID.25575446	0.57592708	1.32116844	Positive	0.87779092	0.20502865
X5Q_Breast.Cancer.Res.Treat.2012_PMID.22048815	-0.458965	-1.3280614	Negative	0.87779092	0.20518697
GSEA_GP3_Tumor_suppressing_miRNA_targets.r.0.940_GTTTGTT.MIR_495	-0.5433213	-1.3251445	Negative	0.87779092	0.20710077
MECM_BMC.Med.Genomics.2011_PMID.21214954	-0.6197477	-1.3142791	Negative	0.87779092	0.2072829
aStr_Prat_BCR.2015_PMID.25575446	-0.6014169	-1.3051085	Negative	0.87779092	0.2103141
LumB_Basal_JCI.2020_PMID.32573490	0.20593928	1.31357498	Positive	0.87779092	0.2107523
Scorr_EMAT3_Correlation_BCR.2020_PMID.32641077	0.5956926	1.30083575	Positive	0.87779092	0.21225766
Lim2009_MatureLum_Adam_PMID.25575446	0.21821538	1.29714558	Positive	0.87779092	0.21297454
TCGA.BRCA.1198_UNKNOWN2_JCI.2020_PMID.32573490	-0.4903354	-1.294336	Negative	0.87779092	0.21391956
CIBERSORT_Mast_cells_activated_Nat.Methods.2015_PMID.25822800	-0.585388	-1.294476	Negative	0.87779092	0.21443906
GSEA_GP21_Anti_apoptosis_DNA_stability.r.0.925_MORF_MT4	-0.5831205	-1.2933469	Negative	0.87779092	0.21632211
Pcorr_classical_CCR.2010_PMID.20643781	0.61233785	1.28160951	Positive	0.87779092	0.21824175
Scorr_P53_Mut_Correlation_BMC.Cancer.2006_PMID.17150101	0.53476769	1.27931443	Positive	0.87779092	0.21902848
TCGA.BRCA.1198_UNKNOWN5_JCI.2020_PMID.32573490	0.4646166	1.28177525	Positive	0.87779092	0.22026366
Mouse_Human_ImmuneProfiles_SHAY_M_H_Induced_in_B_PNAS.2013_PMID.2	-0.6893443	-1.2753866	Negative	0.87779092	0.22038006

PDGFRA_Single_Gene	-0.5745308	-1.2723019	Negative	0.87779092	0.22144614
New.Immune_ImSig.Monocytes_CancerImmunolRes.2018_PMID.30266715	-0.554271	-1.2760632	Negative	0.87779092	0.22236065
C_MYB_Signature_PLoS.One.2010_PMID.20949095	-0.4137199	-1.2754219	Negative	0.87779092	0.22363754
MDSC_tumor_Schlecker_J.Immunol.2012_PMID.23152559	-0.505507	-1.2654406	Negative	0.87779092	0.2259023
JANES_Oscillation_JUND_KRT5_Nat.Cell.Biol.2014_PMID.24658685	-0.5941134	-1.2631019	Negative	0.87779092	0.22593108
New.Immune_TCGA.BRCA.1198_immune_HLA_D_Cell.2015_PMID.26451490	-0.509451	-1.2637596	Negative	0.87779092	0.22668347
TCGA.BRCA.1198_immune_HLA_D_JCI.2020_PMID.32573490	-0.509451	-1.2637596	Negative	0.87779092	0.22668347
FGFR4_Induced_JCI.2020_PMID.32573490	-0.4523633	-1.252012	Negative	0.87779092	0.22939024
Secretoglobin_BMC.Med.Genomics.2011_PMID.21214954	0.28040731	1.25321073	Positive	0.87779092	0.23128476
aStr_HsEnriched_Refined2_BCR.2015_PMID.25575446	-0.5942831	-1.2398341	Negative	0.87779092	0.23291355
New.Immune_Charoentong_Natural_killer_cell_CellRep.2017_PMID.28052254	-0.6978493	-1.2410558	Negative	0.87779092	0.23400383
New.Immune_ImSig.Neutrophils_CancerImmunolRes.2018_PMID.30266715	-0.4884461	-1.2302272	Negative	0.87779092	0.23665707
Claudin_Low_Genome.Biol.2007_PMID.17493263	0.54916019	1.23023509	Positive	0.87779092	0.23741242
Early_IRS_2_PLoS.One.2016_PMID.26991655	-0.4478395	-1.2260609	Negative	0.87779092	0.23791552
Fibroblasts_MCP_PMID.31942075_PMID.31942077	-0.5057962	-1.225753	Negative	0.87779092	0.2380283
CIBERSORT_Neutrophils_Nat.Methods.2015_PMID.25822800	-0.4935434	-1.2224565	Negative	0.87779092	0.23994585
IMMUNE_Bindea_Cell_Tem_Immunity.2013_PMID.24138885	-0.4677208	-1.2164193	Negative	0.87779092	0.24146622
Duke_Module02_akt_Mike_PMID.20335537	-0.4859691	-1.2192324	Negative	0.87779092	0.2427723
GSEA_HDAC_TARGETS_DN_HELLER	-0.5813462	-1.2123144	Negative	0.87779092	0.24299027
MM_Green20_BMC.Med.Genomics.2011_PMID.21214954	-0.4420371	-1.2104103	Negative	0.87779092	0.24369976
TCGA.BRCA.1198_Chromogranin_JCI.2020_PMID.32573490	0.71613772	1.20879474	Positive	0.87779092	0.24478631
MUnknown_15_BMC.Med.Genomics.2011_PMID.21214954	-0.4898349	-1.2125654	Negative	0.87779092	0.24554055
MM_Red9_BMC.Med.Genomics.2011_PMID.21214954	-0.5332556	-1.2080297	Negative	0.87779092	0.24567883
EMT_UP_Weinberg_PNAS.2010_PMID.20713713	-0.5175108	-1.2048039	Negative	0.87779092	0.24579791
Stingl_Up_Basal_High_Nat.Cell.Biol.2014_PMID.25173976	0.25082599	1.21234842	Positive	0.87779092	0.24661014
TLS_CXCL13_SingleGene_.31942077	0.50414604	1.20743728	Positive	0.87779092	0.24671749
MM_p53null.Luminal_Genome.Biol.2013_PMID.24220145	-0.4270546	-1.2014993	Negative	0.87779092	0.24704115
TCGA.BRCA.1198_EN1_FDZ9_JCI.2020_PMID.32573490	0.38036738	1.20761944	Positive	0.87779092	0.24704544
MM_p53null_1pFDR_UP_Genome.Biology.2007_PMID.17493263	-0.44676	-1.2068238	Negative	0.87779092	0.24748993
MVEGFC_BMC.Med.Genomics.2011_PMID.21214954	-0.5302413	-1.1995783	Negative	0.87779092	0.24833533
CD34_CD36_Cluster_BMC.Med.Genomics.2011_PMID.21214954	-0.5670877	-1.19334	Negative	0.87779092	0.25013141
Immunosuppression_PMID.31942077	-0.5893346	-1.1962589	Negative	0.87779092	0.25057384
PTEN_Single_Gene	-0.5191502	-1.18627	Negative	0.87779092	0.25283299
GHI_RS_Model_NJEM.2004_PMID.15591335	0.57384769	1.18425246	Positive	0.87779092	0.25360798
ACTIVATED_BLOOD_NEUTROPHIL_SIGNATURE_Nat.Cell.Biol.2019_PMID.31263	-0.5533235	-1.1849926	Negative	0.87779092	0.25404747
MM_Red13_BMC.Med.Genomics.2011_PMID.21214954	0.62692462	1.18269706	Positive	0.87779092	0.2542067
HS_Red11_BMC.Med.Genomics.2011_PMID.21214954	-0.4366606	-1.1892459	Negative	0.87779092	0.25477308
X8p_Amplicon_BMC.Med.Genomics.2011_PMID.21214954	0.66372077	1.1810699	Positive	0.87779092	0.25483419
Cytotoxic_lymphocytes_MCP_PMID.31942075_PMID.31942077	-0.4719437	-1.1813254	Negative	0.87779092	0.25605948
MM_Potluck_1pFDR_UP_Genome.Biology.2007_PMID.17493263	-0.550219	-1.1743675	Negative	0.87779092	0.25821479
Inflammatory_Breast_Cancer_491_IBC_CCR.2013_PMID.23396049	-0.6254155	-1.1732862	Negative	0.87779092	0.25824468
TCGA.BRCA.1198_S100A7_8_9_JCI.2020_PMID.32573490	0.34700091	1.1769731	Positive	0.87779092	0.25870221
Endothelial_cells_MCP_PMID.31942075_PMID.31942077	-0.6260015	-1.1702601	Negative	0.87779092	0.25903283
Fibromatosis_Lab.Invest.2008_PMID.18414401	-0.5461038	-1.168321	Negative	0.87779092	0.25979153
HS_Green7_BMC.Med.Genomics.2011_PMID.21214954	-0.4895468	-1.1733562	Negative	0.87779092	0.26058746
Duke_Module09_hypoxia_Mike_PMID.20335537	-0.4020784	-1.1718472	Negative	0.87779092	0.26101162
GSEA_GP10_Fatty_acid_oxidation.r.0.930_CARBOXYLIC_ACID_METABOLIC_PRC	-0.4264192	-1.1697072	Negative	0.87779092	0.26133075
IntClust_Deletion.50_Genome.Biol.2014_PMID.25164602	0.54805633	1.16677724	Positive	0.87779092	0.26150557
Mouse_Human_ImmuneProfiles_SHAY_M_H_Induced_in_DC_PNAS.2013_PMID	-0.5848015	-1.1609729	Negative	0.87779092	0.26268184
Verhaak_Stromal_Nat.Commun.2013_PMID.24113773	-0.5768829	-1.1619845	Negative	0.87779092	0.26312962
New.Immune_Miller_M_D_Metagene_9gene_Genome_Biol_2013_PMID.23618	-0.4774411	-1.1652644	Negative	0.87779092	0.26321004
Miller_M_D_Metagene_Genome_Biol_2013_PMID.23618380	-0.4774411	-1.1652644	Negative	0.87779092	0.26321004
New.Immune_Charoentong_Macrophage_CellRep.2017_PMID.28052254	-0.5286007	-1.1617821	Negative	0.87779092	0.26387521
Knudsen_Neo_ER_negative_Clin.Cancer.Res.2014_PMID.25047707	-0.5345021	-1.1575699	Negative	0.87779092	0.26526428
HRneg.Tneg_14.Genes_BCR.2010_PMID.20946665	0.60377543	1.15882396	Positive	0.87779092	0.26596011
Unknown_12_BMC.Med.Genomics.2011_PMID.21214954	-0.3659529	-1.1505755	Negative	0.87779092	0.26962749
Tcell_EXH_Memory_CD8_T_cell_a_vs_Naive_CD8_T_cell_Metagene_3_Science.	-0.4626114	-1.1445799	Negative	0.87779092	0.27029217
PGR_Single_Gene	-0.3855846	-1.1414276	Negative	0.87779092	0.27048817
MM_Claudinlow_Genome.Biol.2013_PMID.24220145	-0.5455481	-1.1415927	Negative	0.87779092	0.27122821
Interferon_Breast.Cancer.Research.2008_PMID.19272155	0.36185486	1.14562364	Positive	0.87779092	0.27205898
Tcell_EXH_Exhausted_CD8_T_cell_vs_Naive_CD8_T_cell_Metagene_3_Science.2	-0.5746824	-1.1290147	Negative	0.87779092	0.27623036
NFIB_Single_Gene	-0.3252807	-1.1319109	Negative	0.87779092	0.27754708
HS_Red5_BMC.Med.Genomics.2011_PMID.21214954	0.48915615	1.12084861	Positive	0.87779092	0.2788943
Influenza_11genes_Metasignature_KHATRI_Immunity.2015_PMID.26682989	0.43645218	1.12333181	Positive	0.87779092	0.28082272
MUnknown_10_BMC.Med.Genomics.2011_PMID.21214954	-0.5327475	-1.1207051	Negative	0.87779092	0.28138895
GSEA_GP2_Immune_Tcell_Bcell.r.0.964_KEGG_HEMATOPOIETIC_CELL_LINEAGE	-0.5985331	-1.1116982	Negative	0.87779092	0.28335046
CIBERSORT_Monocytes_Nat.Methods.2015_PMID.25822800	-0.4936538	-1.1073381	Negative	0.87779092	0.2858942
GSEA_GP8_FOXO_stemness.r.0.875_MORF_PTPRB	-0.4724223	-1.1060625	Negative	0.87779092	0.28709741
IMMUNE_Bindea_Cell_Neutrophils_Immunity.2013_PMID.24138885	-0.4671408	-1.1000008	Negative	0.87779092	0.28760724
MM_WapINT3_1pFDR_UP_Genome.Biology.2007_PMID.17493263	-0.4736951	-1.106132	Negative	0.87779092	0.28770849
LOBULAR_TCGA_SUBTYPE_Reactive_like_Cell.2015_PMID.26451490	0.3538839	1.10165951	Positive	0.87779092	0.28905919
X17PP13_Amplicon_BMC.Med.Genomics.2011_PMID.21214954	-0.4568503	-1.1006511	Negative	0.87779092	0.28956548
CD103_Negative_Cancer.Cell.2014_PMID.25446897	-0.4861947	-1.0952102	Negative	0.87779092	0.29083652
MM_Green18_BMC.Med.Genomics.2011_PMID.21214954	-0.4711754	-1.0903705	Negative	0.87779092	0.29169934
HS_Green15_BMC.Med.Genomics.2011_PMID.21214954	-0.4818757	-1.0909431	Negative	0.87779092	0.29260934
MM_Green8_BMC.Med.Genomics.2011_PMID.21214954	-0.4094085	-1.0892207	Negative	0.87779092	0.29274503
Scorr_P53_Wt_Correlation_BMC.Cancer.2006_PMID.17150101	-0.4606554	-1.087685	Negative	0.87779092	0.2928481
GSEA_GP21_Anti_apoptosis_DNA_stability.r.0.898_MORF_STK17A	-0.5255573	-1.0839302	Negative	0.87779092	0.29475899
MS4A1_Single_Gene	-0.5096046	-1.081959	Negative	0.87779092	0.29530855
Wahl_fMasC_Signature_Cell.Stem.Cell.2012_PMID.22305568	0.51724907	1.08629054	Positive	0.87779092	0.29580008
Unknown_3_BMC.Med.Genomics.2011_PMID.21214954	-0.4816571	-1.0832525	Negative	0.87779092	0.29668043
GSEA_BIOCARTA_ALK_PATHWAY	-0.4815342	-1.0792469	Negative	0.87779092	0.29703569
HS_Red14_BMC.Med.Genomics.2011_PMID.21214954	0.52371077	1.07752585	Positive	0.87779092	0.29722384
CD68_cluster_Iglesia_CCR.2014_PMID.24916698	-0.4471579	-1.080277	Negative	0.87779092	0.29795647
TCGA.BRCA.1198_COLLAGEN11A_JCI.2020_PMID.32573490	-0.4231869	-1.0772357	Negative	0.87779092	0.29852923
HS_Red16_BMC.Med.Genomics.2011_PMID.21214954	0.37088481	1.07797041	Positive	0.87779092	0.29999726
MM_Green17_BMC.Med.Genomics.2011_PMID.21214954	-0.4893682	-1.0699932	Negative	0.87779092	0.30286068
Mouse_Human_ImmuneProfiles_SHAY_M_H_Induced_in_NK_PNAS.2013_PMID	-0.5411169	-1.062401	Negative	0.87779092	0.30382682
HS_Green20_BMC.Med.Genomics.2011_PMID.21214954	-0.4172169	-1.0608613	Negative	0.87779092	0.30665854
Scorr_EMAT1_Correlation_BCR.2020_PMID.32641077	-0.4901209	-1.0574646	Negative	0.87779092	0.307237
GSEA_BIOCARTA_RB_PATHWAY	-0.4656446	-1.0503019	Negative	0.87779092	0.30918527

MHC.II_Breast.Cancer.Research.2008_PMID.19272155	-0.4298851	-1.0496307	Negative	0.87779092	0.31102187
Unknown_2_BMC.Med.Genomics.2011_PMID.21214954	-0.4648822	-1.046253	Negative	0.87779092	0.31265614
Scorr_EMAT4_Correlation_BCR.2020_PMID.32641077	0.30596442	1.04621616	Positive	0.87779092	0.31289799
NRAS_Single_Gene	0.35174966	1.04586065	Positive	0.87779092	0.31410939
New.Immune_Charoentong_T_follicular_helper_cell_CellRep.2017_PMID.28052	-0.5001935	-1.0410758	Negative	0.87779092	0.31461784
aStr_HsEnriched_BCR.2015_PMID.25575446	-0.5062601	-1.037911	Negative	0.87779092	0.31474354
MSquamous_BMC.Med.Genomics.2011_PMID.21214954	0.26984822	1.0433691	Positive	0.87779092	0.31501704
MM_Red6_BMC.Med.Genomics.2011_PMID.21214954	-0.4354192	-1.0335016	Negative	0.87779092	0.31673878
Lymphovascular_Invasion_J.Pathol.2017_PMID.27861902	0.34602769	1.03146863	Positive	0.87779092	0.31766174
GSEA_RETINOL_METABOLISM_KEGG	-0.4981208	-1.0303589	Negative	0.87779092	0.31816636
New.Immune_Charoentong_Effector_memeory_CD4_T_cell_CellRep.2017_PMII	-0.5537974	-1.0278965	Negative	0.87779092	0.31982673
FGFR4_Repressed_JCI.2020_PMID.32573490	-0.32541	-1.030733	Negative	0.87779092	0.32084252
Lim.et.al.2010.Conserved.Mature_BCR.2010_PMID.20346151	0.19785663	1.01774051	Positive	0.87779092	0.32489856
MM_Red15_BMC.Med.Genomics.2011_PMID.21214954	-0.4705649	-1.0165646	Negative	0.87779092	0.32522861
Keller2012_CD10_Adam_PMID.25575446	-0.3615499	-1.0126928	Negative	0.87779092	0.32827413
MM_Green1_BMC.Med.Genomics.2011_PMID.21214954	-0.5076658	-1.0090834	Negative	0.87779092	0.32885108
RSS_Score_Clin.Cancer.Res.2018_PMID.29921729	-0.3850416	-1.0104896	Negative	0.87779092	0.32963091
Pcorr_squamoid_PLOS.2012_PMID.22590557	-0.2937075	-1.0089878	Negative	0.87779092	0.3303015
Unknown_15_BMC.Med.Genomics.2011_PMID.21214954	0.43774653	1.0016755	Positive	0.87779092	0.33224701
MHC_Forero_11_Cancer.Immunol.Res.2016_PMID.26980599	-0.4345937	-1.0037879	Negative	0.87779092	0.33248167
Pcorr_Small_Cell_Carcinoma_JMD.2013_PMID.23701907	-0.4657901	-1.0018457	Negative	0.87779092	0.33414115
Stromal_Signature_Nat.Med.2008_PMID.18438415	-0.5311103	-0.9964809	Negative	0.87779092	0.33444538
MITO2_BMC.Med.Genomics.2011_PMID.21214954	-0.4379077	-0.9936622	Negative	0.87779092	0.33742279
MM_Red1_BMC.Med.Genomics.2011_PMID.21214954	-0.5134688	-0.9902181	Negative	0.87779092	0.33770772
aMaSC_HsEnriched_Refined1_BCR.2015_PMID.25575446	-0.5762344	-0.9866992	Negative	0.87779092	0.33879256
CIBERSORT_Macrophages_M2_Nat.Methods.2015_PMID.25822800	-0.4637565	-0.9876278	Negative	0.87779092	0.3394368
HS_Green4_BMC.Med.Genomics.2011_PMID.21214954	-0.4479708	-0.9847722	Negative	0.87779092	0.3400514
GATA3_Single_Gene	0.35975789	0.98526355	Positive	0.87779092	0.34135462
GSEA_GP22_16Q22_24_amplicon.r.0.927_PerouLab_16q24x_BMC_Med_Genor	-0.4498742	-0.976778	Negative	0.87779092	0.34483131
GSEA_GP16_Protein_kinase_signailng_MAPKs.r.0.885_REGULATION_OF_KINASI	-0.4680972	-0.977222	Negative	0.87779092	0.34484864
MUnknown_24_BMC.Med.Genomics.2011_PMID.21214954	-0.3373074	-0.9780748	Negative	0.87779092	0.34497198
MM_Green21_BMC.Med.Genomics.2011_PMID.21214954	0.41271326	0.97679477	Positive	0.87779092	0.34563167
MM_Class3_Genome.Biol.2013_PMID.24220145	0.51838897	0.97224387	Positive	0.87779092	0.34667934
CIBERSORT_Mast_cells_resting_Nat.Methods.2015_PMID.25822800	-0.4219569	-0.9677114	Negative	0.87779092	0.34758983
ID4_Single_Gene	0.27338768	0.97088412	Positive	0.87779092	0.34815005
New.Immune_Tertiary.Lymphoid.Str.melanoma.9gene_Nature.2020_PMID.319	-0.5758803	-0.9677565	Negative	0.87779092	0.34829828
TLS_New_9Gene_Signature_PMID.31942071	-0.5758803	-0.9677565	Negative	0.87779092	0.34829828
Tcell_EXH_Effector_CD8_T_cell_at_day_8_p.i_Armstrong_vs_Naive_CD8_T_cell	-0.5066994	-0.9663063	Negative	0.87779092	0.34891687
HS_Red10_BMC.Med.Genomics.2011_PMID.21214954	-0.4153015	-0.9647168	Negative	0.87779092	0.34904242
Parity_signature_Troester_251_BCR.2014_PMID.25005139	-0.5012532	-0.9639105	Negative	0.87779092	0.35044464
New.Immune_ImSig.PlasmaCells_CancerImmunolRes.2018_PMID.30266715	0.46027058	0.96385021	Positive	0.87779092	0.35092827
Parity_signature_Troester_40_BCR.2014_PMID.25005139	-0.5029793	-0.963103	Negative	0.87779092	0.35104752
Pfefferle2012_MaSC_PMID.25575446	-0.3128815	-0.9604668	Negative	0.87779092	0.35111129
Shehata2012_Stroma_PMID.25575446	-0.4598069	-0.9581523	Negative	0.87779092	0.35248163
Myeloid_dendritic_cells_MCP_PMID.31942075_PMID.31942077	-0.5558477	-0.9564355	Negative	0.87779092	0.35308154
New.Immune_MInterferon_Cluster_BMC.Med.Genomics.2011_PMID.21214954	0.38953459	0.96009631	Positive	0.87779092	0.35376198
GSEA_GP11_Immune_IFN.r.0.965_PerouLab_MInterferon_Cluster_BMC_Med_(	0.38953459	0.96009631	Positive	0.87779092	0.35376198
New.Immune_ImSig.Macrophages_CancerImmunolRes.2018_PMID.30266715	-0.4597236	-0.9556586	Negative	0.87779092	0.35469944
ERBB3_Single_Gene	0.43677626	0.95636093	Positive	0.87779092	0.35475939
GATA3.induced.genes_Oncogene.2004_PMID.15361840	0.39115441	0.95281489	Positive	0.87779092	0.35580222
MUnknown_3_BMC.Med.Genomics.2011_PMID.21214954	-0.400556	-0.9477152	Negative	0.87779092	0.35736963
New.Immune_Charoentong_Type_1_T_helper_cell_CellRep.2017_PMID.280522	-0.5147631	-0.9469247	Negative	0.87779092	0.35871268
MET_DOWN_Significant_Genes_LOW_BASALS_1_Genes_JCI.2018_PMID.29480	-0.3075034	-0.9457624	Negative	0.87779092	0.35944178
MM_WapINT3.2012_Genome.Biol.2013_PMID.24220145	-0.4676944	-0.9459324	Negative	0.87779092	0.3596434
Pfefferle2012_Stroma_PMID.25575446	-0.4414754	-0.941922	Negative	0.87779092	0.36023814
IgG_Breast.Cancer.Research.2008_PMID.19272155	0.53238437	0.94289703	Positive	0.87779092	0.36090477
Spike2012_fStr_PMID.25575446	-0.4083338	-0.9400703	Negative	0.87779092	0.36115835
UNC_ROR_S_Model_JCO.2009_PMID.19204204	0.45118923	0.93925279	Positive	0.87779092	0.36156514
PDGFRB_Single_Gene	-0.4815077	-0.938114	Negative	0.87779092	0.3621323
CD103_Ratio_Cancer.Cell.2014_PMID.25446897	0.43886636	0.93872375	Positive	0.87779092	0.36221729
aStr_Lim09_BCR.2015_PMID.25575446	-0.4492002	-0.9361805	Negative	0.87779092	0.36347158
New.Immune_Miller_B_P_Metagene_16gene_Genome_Biol_2013_PMID.23618	0.54455629	0.93472555	Positive	0.87779092	0.36492929
Miller_B_P_Metagene_Genome_Biol_2013_PMID.23618380	0.54455629	0.93472555	Positive	0.87779092	0.36492929
New.Immune_Charoentong_Gamma_delta_T_cell_CellRep.2017_PMID.280522	-0.3500203	-0.9329701	Negative	0.87779092	0.36502101
MUnknown_26_BMC.Med.Genomics.2011_PMID.21214954	0.38784062	0.93373986	Positive	0.87779092	0.36528943
GSEA_GP18_Vesicle_EPR_membrane_coat.r.0.877_MEMBRANE_COAT	-0.3997452	-0.9343567	Negative	0.87779092	0.36553932
Bcells_Naive_J.Clin.Oncol.2015_PMID.25800755	-0.494513	-0.931812	Negative	0.87779092	0.36667501
HCK_Breast.Cancer.Research.2008_PMID.19272155	-0.438122	-0.9304056	Negative	0.87779092	0.36734751
Shehata2012_ALDHpos_PMID.25575446	0.30024767	0.931044	Positive	0.87779092	0.36788701
Lim.et.al.2010.Conserved.Luminal.Progenitor_BCR.2010_PMID.20346151	0.29765496	0.92575219	Positive	0.87779092	0.36886005
S100A9_A8_BMC.Med.Genomics.2011_PMID.21214954	0.31052548	0.92461391	Positive	0.87779092	0.36921316
Duke_Module13_myc_Mike_PMID.20335537	-0.4471925	-0.9233596	Negative	0.87779092	0.36997556
CIBERSORT_Dendritic_cells_resting_Nat.Methods.2015_PMID.25822800	-0.477206	-0.9216825	Negative	0.87779092	0.37136689
GSEA_GP12_Hypoxia_glycolosis.r.0.939_SEMENZA_HIF1_TARGETS	-0.3678005	-0.9179143	Negative	0.87779092	0.37276489
PR_Isoform_Ratio_Up_in_PRB_H_JNCI.2017_PMID.28376177	0.41564154	0.91622341	Positive	0.87779092	0.3731534
HS_Red3_BMC.Med.Genomics.2011_PMID.21214954	-0.43746	-0.9142851	Negative	0.87779092	0.37414014
Murat_G24_J.Clin.Oncol.2008_PMID.18565887	-0.4357437	-0.9141958	Negative	0.87779092	0.37535455
MHC_Forero_24_Cancer.Immunol.Res.2016_PMID.26980599	-0.4828182	-0.913439	Negative	0.87779092	0.37558269
Lim2009_Stroma_Adam_PMID.25575446	-0.4286605	-0.9104826	Negative	0.87779092	0.37642371
MUnknown_28_BMC.Med.Genomics.2011_PMID.21214954	-0.3634766	-0.9121025	Negative	0.87779092	0.37702857
Pfefferle2012_MatureLum_PMID.25575446	-0.1704388	-0.9067348	Negative	0.87779092	0.3783653
GSEA_BIOCARTA_AKT_PATHWAY	-0.4523747	-0.9061791	Negative	0.87779092	0.37882933
LOBULAR_TCGA_SIGNATURE_Immune_related_Cell.2015_PMID.26451490	-0.4300416	-0.9034643	Negative	0.87779092	0.38122304
MM_Red7_BMC.Med.Genomics.2011_PMID.21214954	-0.3448738	-0.9000593	Negative	0.87779092	0.38143613
New.Immune_Miller_Bcell_Plasma_52gene_PMID.23618380	0.49788736	0.90089274	Positive	0.87779092	0.38209355
HS_Green14_BMC.Med.Genomics.2011_PMID.21214954	-0.4711159	-0.8989417	Negative	0.87779092	0.38219372
IMMUNE_Bindea_Cell_Cytotoxic_cells_Immunity.2013_PMID.24138885	-0.4494802	-0.8992892	Negative	0.87779092	0.38270655
New.Immune_Charoentong_Activated_dendritic_cell_CellRep.2017_PMID.2805	-0.3600445	-0.9002095	Negative	0.87779092	0.38312111
EGFR_Single_Gene	-0.1656592	-0.8938076	Negative	0.87779092	0.38467251
GSEA_GP21_Anti_apoptosis_DNA_stability.r.0.877_MORF_BCL2	-0.2899617	-0.8937439	Negative	0.87779092	0.38682583
HS_Green22_BMC.Med.Genomics.2011_PMID.21214954	-0.4125102	-0.8933052	Negative	0.87779092	0.38691019

MAPK_pathway_activation_Wagle_NPJ.Precis.Oncol.2018_PMID.29872725	-0.4203068	-0.8922122	Negative	0.87779092	0.38746847
GO_DOWN_with_SOX10_OE_Cell.Rep.2015_PMID.26365194	0.29270174	0.88705746	Positive	0.87779092	0.39053362
T_regulatory_cell_2gene_PMID.31942077	0.36754853	0.88311925	Positive	0.87779092	0.39243471
MUnknown_23_BMC.Med.Genomics.2011_PMID.21214954	-0.4226107	-0.8805003	Negative	0.87779092	0.39314374
Bcells_Plasmablast_J.Clin.Oncol.2015_PMID.25800755	-0.4480437	-0.8769774	Negative	0.87779092	0.39347639
Scorr_PTEN_Present_Correlation_PNAS.2007_PMID.17452630	0.42511231	0.87343672	Positive	0.87779092	0.39534541
CIBERSORT_Macrophages_M0_Nat.Methods.2015_PMID.25822800	-0.3859085	-0.8755972	Negative	0.87779092	0.39537489
New.Immune_Miller_Monocyte_DendriticCell_25gene_PMID.23618380	-0.3968391	-0.8758364	Negative	0.87779092	0.39552193
MM_Red21_BMC.Med.Genomics.2011_PMID.21214954	0.4191275	0.8739055	Positive	0.87779092	0.39596837
TCGA.BRCA.1198_IMMUNOGLOBULIN_PMID.26451490	0.46626311	0.87159267	Positive	0.87779092	0.39732007
MS_CD44_DOWN_PNAS.2009_PMID.19666588	0.43668605	0.86925611	Positive	0.87779092	0.3982732
Pcorr_IGS_Correlation_NJEM.2007_PMID.17229949	-0.4027718	-0.8715621	Negative	0.87779092	0.3984796
Lums_HER2E_UP_metastatic.signature_JCI.2020_PMID.32573490	-0.4489938	-0.8681564	Negative	0.87779092	0.39879943
MASC_Up_Nat.Med.2009_PMID.19648928	-0.4316472	-0.8666543	Negative	0.87779092	0.39917626
New.Immune_ImmLandscape_Macro.mono_CSF1_core_response.Clin_Can_Res	-0.4285759	-0.8667214	Negative	0.87779092	0.40005603
TCGA_CSF1_response_Immunity.2018_PMID._29628290	-0.4285759	-0.8667214	Negative	0.87779092	0.40005603
African_and_European_Ancestry_in_TCGA_Negative_JAMA.Oncol.2017_PMID.2	-0.3793111	-0.865497	Negative	0.87779092	0.40094355
Interferon_Cluster_BMC.Med.Genomics.2011_PMID.21214954	0.31811832	0.8667579	Positive	0.87779092	0.40121278
Down_regulated_upon_N_RAS_repression_Cell_Rep.2015_PMID.26166574	-0.4104889	-0.8610131	Negative	0.87779092	0.40299199
LOBULAR_TCGA_SIGNATURE_Reactive_like_Cell.2015_PMID.26451490	0.32264378	0.86181333	Positive	0.87779092	0.40332114
MM_Normal.like_Genome.Biol.2013_PMID.24220145	-0.3342877	-0.8585183	Negative	0.87779092	0.4035983
MUnknown_11_BMC.Med.Genomics.2011_PMID.21214954	0.30588843	0.86011303	Positive	0.87779092	0.40468769
Scorr_PTEN_Absent_Correlation_PNAS.2007_PMID.17452630	-0.4150623	-0.8523139	Negative	0.87779092	0.4066175
MDACC.FNA.1_J.Clin.Oncol.2010_PMID.20805453	-0.4162362	-0.8494619	Negative	0.87779092	0.40977048
New.Immune_Charoentong_Plasmacytoid_dendritic_cell_CellRep.2017_PMID.2	-0.4078525	-0.8470728	Negative	0.87779092	0.4106817
Apocrine_Features_J.Pathol.2017_PMID.27861902	-0.3341369	-0.8434666	Negative	0.87779092	0.41140067
MM_BRCAwnt_1pFDR_UP_Genome.Biology.2007_PMID.17493263	-0.4066926	-0.8430182	Negative	0.87779092	0.41341658
New.Immune_Charoentong_MDSC_CellRep.2017_PMID.28052254	-0.4188258	-0.8419622	Negative	0.87779092	0.41342582
MNB1_BMC.Med.Genomics.2011_PMID.21214954	-0.3544131	-0.8405062	Negative	0.87779092	0.41376513
Stingl_Day7_Downregulated_Nat.Cell.Biol.2014_PMID.25173976	-0.4695855	-0.8397284	Negative	0.87779092	0.41422241
New.Immune_Charoentong_Effector_memeory_CD8_T_cell_CellRep.2017_PMI	-0.4643475	-0.8394706	Negative	0.87779092	0.41439104
UNC_Scorr_LumA_Correlation_JCO.2009_PMID.19204204	-0.3520154	-0.8367661	Negative	0.87779092	0.41504751
IMMUNE_Bindea_Cell_Mast_cells_Immunity.2013_PMID.24138885	-0.3377615	-0.8327928	Negative	0.87779092	0.41721987
Pcorr_Dasatinib_R_Correlation_Cancer.Res.2007_PMID.17332353	-0.3256669	-0.8332543	Negative	0.87779092	0.41751275
Melanoma_Scorr_Keratin_Cell.2015_PMID.26091043	-0.3582848	-0.8343314	Negative	0.87779092	0.4179348
Unknown_14_BMC.Med.Genomics.2011_PMID.21214954	-0.1921563	-0.8342325	Negative	0.87779092	0.41832412
Lim.et.al.2010.Conserved.aMaSC_BCR.2010_PMID.20346151	-0.4353937	-0.8288064	Negative	0.87779092	0.41971858
New.Immune_TCGA.BRCA.1198_immune_INTERFERON_Cell.2015_PMID.26451	0.31828588	0.83206668	Positive	0.87779092	0.4197937
TCGA.BRCA.1198_immune_INTERFERON_JCI.2020_PMID.32573490	0.31828588	0.83206668	Positive	0.87779092	0.4197937
TCGA.BRCA.1198_PDCHA_MANY_JCI.2020_PMID.32573490	0.25336168	0.82944971	Positive	0.87779092	0.4207225
fMaSC_Signature_CellRep.2018_PMID.30089273	-0.40875	-0.8240764	Negative	0.87779092	0.42294946
METAPLASTIC_Up_CanRes.2009_PMID.19435916	-0.3832569	-0.8220419	Negative	0.87779092	0.42313459
MM_Red8_BMC.Med.Genomics.2011_PMID.21214954	-0.3941548	-0.8251117	Negative	0.87779092	0.42314563
Stromal_Up_Nat.Med.2009_PMID.19648928	-0.4022806	-0.8197035	Negative	0.87805713	0.42472936
New.Immune_Charoentong_Regulatory_T_cell_CellRep.2017_PMID.28052254	-0.4063698	-0.8193666	Negative	0.87805713	0.42537456
Wahl_fSTR_Signature_Cell.Stem.Cell.2012_PMID.22305568	-0.3811597	-0.8066971	Negative	0.88940058	0.43193377
MacTh1_cluster_Iglesia_CCR.2014_PMID.24916698	-0.3872114	-0.8046741	Negative	0.89145242	0.43410737
MM_PyMT.2012_Genome.Biol.2013_PMID.24220145	-0.2985634	-0.8014683	Negative	0.89145242	0.43584994
Bcells_Centroblast_J.Clin.Oncol.2015_PMID.25800755	0.38787538	0.79875541	Positive	0.89145242	0.43612924
Claudin_High_Genome.Biol.2007_PMID.17493263	-0.4376604	-0.7965934	Negative	0.89270556	0.43804003
Mmyosin_BMC.Med.Genomics.2011_PMID.21214954	0.28120694	0.79327766	Positive	0.89270556	0.43922229
UNC_Scorr_Basal_Correlation_JCO.2009_PMID.19204204	0.24890359	0.79276172	Positive	0.89270556	0.4399458
Scorr_EMAT2_Correlation_BCR.2020_PMID.32641077	-0.3829115	-0.7913251	Negative	0.89323937	0.44127734
Excellent_Pathologic_Response_ER.Pos_39_JAMA.2011_PMID.21558518	0.28974847	0.79022412	Positive	0.89329247	0.44293461
GO_UP_with_SOX10_OE_Cell.Rep.2015_PMID.26365194	-0.3682385	-0.7867939	Negative	0.89329247	0.44419168
LumProg_Shehata_BCR.2015_PMID.25575446	-0.3706299	-0.7843705	Negative	0.89329247	0.44562674
MM_Red22_BMC.Med.Genomics.2011_PMID.21214954	-0.4063348	-0.7799832	Negative	0.89329247	0.44793413
MUnknown_2_BMC.Med.Genomics.2011_PMID.21214954	-0.3193595	-0.7774038	Negative	0.89329247	0.44826325
Bcells_Memory_J.Clin.Oncol.2015_PMID.25800755	-0.4106546	-0.7770223	Negative	0.89329247	0.44923415
Pcorr_Dasatinib_L_Correlation_Cancer.Res.2007_PMID.17332353	0.29692581	0.77602655	Positive	0.89329247	0.44960981
MM_Green12_BMC.Med.Genomics.2011_PMID.21214954	-0.4122031	-0.7746354	Negative	0.89329247	0.44985183
IMMUNE_Bindea_Cell_NK_CD56bright_cells_Immunity.2013_PMID.24138885	0.42108662	0.772508	Positive	0.89359865	0.45107491
Duke_Module04_e2f1_Mike_PMID.20335537	-0.3649582	-0.7664748	Negative	0.89795257	0.45563497
aStr_HsEnriched_Refined1_BCR.2015_PMID.25575446	-0.3822446	-0.7643714	Negative	0.89795257	0.45577185
AGE_associated_SOOD_Genome.Biol.2015_PMID.26343147	0.33515736	0.76377257	Positive	0.89795257	0.45656354
GSEA_GP5_MYC_targets_TERT.r.0.922_PerouLab_MM_Myc_1pFDR_UP_Genom	-0.3840863	-0.7629888	Negative	0.89795257	0.45756913
MM_Red24_BMC.Med.Genomics.2011_PMID.21214954	-0.1929455	-0.7575884	Negative	0.90047275	0.46041979
New.Immune_IMMUNE_Bindea_Cell_iDC_Median_Immunity.2013_PMID.2413	0.3628885	0.75130009	Positive	0.90047275	0.46384575
New.Immune_TCGA.BRCA.1198_immune_CD4_CD53_CD84_BTK_Cell.2015_PM	-0.3621543	-0.7508862	Negative	0.90047275	0.46487431
TCGA.BRCA.1198_immune_CD4_CD53_CD84_BTK_JCI.2020_PMID.32573490	-0.3621543	-0.7508862	Negative	0.90047275	0.46487431
MM_Red17_BMC.Med.Genomics.2011_PMID.21214954	-0.3980112	-0.7472729	Negative	0.90047275	0.46670394
Mouse_Human_ImmuneProfiles_SHAY_M_H_Induced_in_T_PNAS.2013_PMID.	-0.4909742	-0.7402529	Negative	0.90047275	0.46987064
African_and_European_Ancestry_in_TCGA_Positive_JAMA.Oncol.2017_PMID.2	-0.3039377	-0.7420041	Negative	0.90047275	0.47040981
X16.13_Amplicon_BMC.Med.Genomics.2011_PMID.21214954	-0.2237153	-0.7359565	Negative	0.90047275	0.47376432
MM_Green3_BMC.Med.Genomics.2011_PMID.21214954	-0.3264886	-0.7336042	Negative	0.90047275	0.47560164
TCGA.BRCA.1198_NORMAL_JCI.2020_PMID.32573490	-0.2886185	-0.729443	Negative	0.90047275	0.47627456
IMMUNE_Bindea_Cell_pDC_Immunity.2013_PMID.24138885	-0.3478	-0.7277144	Negative	0.90047275	0.47730346
M2_Macrophage_Blood.2006_PMID.16556895	-0.3522154	-0.7221928	Negative	0.90047275	0.48059892
Verhaak_Immune_Nat.Commun.2013_PMID.24113773	-0.3753515	-0.7229111	Negative	0.90047275	0.4810262
IMMUNE_Bindea_Cell_Th2_cells_Immunity.2013_PMID.24138885	0.37260695	0.72283201	Positive	0.90047275	0.48130383
Vascular.Content_Clin.Exp.Metastasis.2014_PMID.23975155	-0.2268407	-0.7229989	Negative	0.90047275	0.48136343
Immune_Suppression_Kardos_JCI.Insight.2016_PMID.27699256	-0.345271	-0.7215819	Negative	0.90047275	0.48194612
MM_Red19_BMC.Med.Genomics.2011_PMID.21214954	-0.3478482	-0.7193116	Negative	0.90047275	0.48294813
ADM_S100A10_A110NDGR1_Cluster_BMC.Med.Genomics.2011_PMID.21214	0.1982749	0.7198788	Positive	0.90047275	0.48347527
Duke_Module21_tgfb_Mike_PMID.20335537	-0.2937445	-0.7185162	Negative	0.90047275	0.48400765
HER1_Cluster3_BMC.Genomics.2007_PMID.17663798	-0.3719916	-0.7166076	Negative	0.90047275	0.48429041
PDCD1_Single_Gene	-0.349327	-0.7171032	Negative	0.90047275	0.48432624
Late_IRS_2_PLoS.One.2016_PMID.26991655	-0.335662	-0.7160833	Negative	0.90047275	0.48457329
GSEA_IGF1R_PATHWAY_BIOCARTA	-0.3575542	-0.7151897	Negative	0.90047275	0.48479805
MHistone_BMC.Med.Genomics.2011_PMID.21214954	-0.4208138	-0.7116799	Negative	0.90047275	0.48691073
MNB3_BMC.Med.Genomics.2011_PMID.21214954	-0.3237143	-0.7097015	Negative	0.90047275	0.48890946

Inflammatory_Breast_Cancer_79_nIBC_CCR.2013_PMID.23396049	-0.3013734	-0.7066253	Negative	0.90047275	0.49111922
Knudsen_Neo_ER_positive_Clin.Cancer.Res.2014_PMID.25047707	0.39703569	0.70457425	Positive	0.90047275	0.49120458
Unknown_1_BMC.Med.Genomics.2011_PMID.21214954	-0.2928383	-0.7034424	Negative	0.90047275	0.49189059
GSEA_GP20_TAL1_Leukemia_erythropoiesis.r.0.935_GNF2_TAL1	-0.301117	-0.7049223	Negative	0.90047275	0.49201563
New.Immune_TCGA.BRCA.1198_IL8_CCL_Cell.2015_PMID.26451490	-0.2828873	-0.7035507	Negative	0.90047275	0.49224408
TCGA.BRCA.1198_IL8_CCL_JCI.2020_PMID.32573490	-0.2828873	-0.7035507	Negative	0.90047275	0.49224408
Duke_Module14_p53_Mike_PMID.20335537	-0.2853673	-0.7023977	Negative	0.90058614	0.49386649
New.Immune_ImSig.InterferonPathway_CancerImmunolRes.2018_PMID.30266	0.26356998	0.70217658	Positive	0.90058614	0.49446057
MM_Red4_BMC.Med.Genomics.2011_PMID.21214954	-0.3468609	-0.6960081	Negative	0.90089091	0.49733844
GSEA_GP17_Basal_signaling.r.0.958_SMID_BREAST_CANCER_BASAL_UP	0.21670162	0.69556011	Positive	0.90089091	0.49771097
TCGA.BRCA.1198_UNKNOWN1_JCI.2020_PMID.32573490	0.26223648	0.6936311	Positive	0.90089091	0.49786076
STAT3_Basal_Horvath_PNAS.2014_PMID.25139989	-0.2805503	-0.6894886	Negative	0.90332926	0.50193531
Tcell_EXH_Exhausted_CD8_T_cell_vs_Naive_CD8_T_cell_Metagene_1_Science.	-0.3367866	-0.6881594	Negative	0.90332926	0.50195521
Shehata2012_LumProg_PMID.25575446	-0.2713611	-0.6874154	Negative	0.90332926	0.50244989
Unknown_6_BMC.Med.Genomics.2011_PMID.21214954	-0.330015	-0.683694	Negative	0.90511404	0.5052745
GENE_PANEL_UNCSEQ_7.1_Oncologist.2018_PMID.29158372	-0.3907588	-0.6835105	Negative	0.90511404	0.50560796
X12qMDM4_BMC.Med.Genomics.2011_PMID.21214954	-0.3328527	-0.6798812	Negative	0.90514949	0.50671048
MM_Green13_BMC.Med.Genomics.2011_PMID.21214954	0.32047934	0.66899795	Positive	0.91530424	0.51349006
Activate.Endothelium_Clin.Exp.Metastasis.2014_PMID.23975155	-0.2643554	-0.6588868	Negative	0.91772322	0.51933898
fMaSC.refined1_PMID.25575446	0.18859987	0.66035388	Positive	0.91772322	0.5196785
Stingl_Up_Proliferation_Nat.Cell.Biol.2014_PMID.25173976	0.34756231	0.65672815	Positive	0.91772322	0.5206905
PARP_Sensitivity_Signature_POSITIVE_Sci.Adv.2017_PMID.28439535	-0.3752067	-0.6570406	Negative	0.91772322	0.52133913
TCGA.BRCA.1198_UNKNOWN3_JCI.2020_PMID.32573490	-0.3423551	-0.6561365	Negative	0.91772322	0.52138387
MUnknown_22_BMC.Med.Genomics.2011_PMID.21214954	-0.3229542	-0.6566678	Negative	0.91772322	0.52208245
MUnknown_21_BMC.Med.Genomics.2011_PMID.21214954	-0.3151292	-0.6534179	Negative	0.91772322	0.52385889
Lim2010_Stroma_Adam_PMID.25575446	-0.3242115	-0.6516534	Negative	0.91772322	0.52423642
STAT1_Breast.Cancer.Research.2008_PMID.19272155	0.25755603	0.649535	Positive	0.91772322	0.52671703
MM_Red18_BMC.Med.Genomics.2011_PMID.21214954	0.33625615	0.64601487	Positive	0.91772322	0.52742747
TCGA.BRCA.1198_MYBL2_APOBEC3B_JCI.2020_PMID.32573490	0.32982846	0.64390214	Positive	0.91772322	0.52876178
HS_Red25_BMC.Med.Genomics.2011_PMID.21214954	-0.1934554	-0.6386019	Negative	0.91772322	0.53315444
HS_Green2_BMC.Med.Genomics.2011_PMID.21214954	-0.3023845	-0.6351209	Negative	0.91772322	0.53432773
GSEA_GP14_Plasma_membrane_cell_cell_signaling.r.0.925_MORF_CNTN1	0.30468383	0.63578457	Positive	0.91772322	0.53526463
X1p36_Amplicon_BMC.Med.Genomics.2011_PMID.21214954	-0.2713403	-0.633641	Negative	0.91772322	0.53637491
MM_Green11_BMC.Med.Genomics.2011_PMID.21214954	-0.3025037	-0.6286944	Negative	0.91772322	0.53906496
HS_Red4_BMC.Med.Genomics.2011_PMID.21214954	-0.3292296	-0.6285148	Negative	0.91772322	0.53945378
Unknown_13_BMC.Med.Genomics.2011_PMID.21214954	-0.2762775	-0.6285653	Negative	0.91772322	0.5397689
X4p16_Amplicon_BMC.Med.Genomics.2011_PMID.21214954	-0.2628051	-0.6260099	Negative	0.91772322	0.54013682
REPLICATION_STRESS_MODEL_Cell.Rep.2018_PMID.29768207	-0.2454277	-0.6242309	Negative	0.91772322	0.54127513
Pcorr_Adenocarcinoma_JMD.2013_PMID.23701907	0.20793273	0.62513169	Positive	0.91772322	0.54226904
BCL2_Single_Gene	-0.2859123	-0.6189733	Negative	0.91772322	0.54464695
New.Immune_ImSig.NKcells_CancerImmunolRes.2018_PMID.30266715	-0.3208419	-0.6167648	Negative	0.91772322	0.54638862
MM_Red11_BMC.Med.Genomics.2011_PMID.21214954	-0.3220295	-0.6168276	Negative	0.91772322	0.54708704
LumB_Her2_JCI.2020_PMID.32573490	0.11071698	0.61580769	Positive	0.91772322	0.54767466
Extensive_Residual_Diesase_ER.Pos_73_JAMA.2011_PMID.21558518	-0.2634464	-0.6160877	Negative	0.91772322	0.54789886
MBASAL_BMC.Med.Genomics.2011_PMID.21214954	-0.3423314	-0.6130694	Negative	0.91772322	0.54867337
New.Immune_Charoentong_Natural_killer_T_cell_CellRep.2017_PMID.2805225	-0.3109782	-0.6108309	Negative	0.91772322	0.55039572
Chemo_Endocrine_Score_CC_.2.LumA_subtract.by_CC.2.Basal_Clin.Cancer.Res.	0.32745769	0.60584995	Positive	0.91772322	0.55311275
myeloid_cell_chemotaxis_1gene_PMID.31942077	-0.2946023	-0.6071446	Negative	0.91772322	0.55348613
X3p21Amplicon_BMC.Med.Genomics.2011_PMID.21214954	0.25047	0.60407703	Positive	0.91772322	0.55426186
IMMUNE_Bindea_Cell_B_cells_Immunity.2013_PMID.24138885	-0.2630874	-0.6043717	Negative	0.91772322	0.55518212
New.Immune_Tcell_CD8_Memory_vs_naive_1.PMID27789795	-0.3142075	-0.6035249	Negative	0.91772322	0.55522444
Tcell_EXH_Memory_CD8_T_cell_a_vs_Naive_CD8_T_cell_Metagene_1_Science.	-0.3142075	-0.6035249	Negative	0.91772322	0.55522444
MM_Normal_1pFDR_UP_Genome.Biology.2007_PMID.17493263	-0.2742685	-0.6019599	Negative	0.91772322	0.55563574
Pcorr_magnoid_PLOS.2012_PMID.22590557	0.35209094	0.59899786	Positive	0.91772322	0.55774726
Mature_LuminaUp_Nat.Med.2009_PMID.19648928	0.14206623	0.59947532	Positive	0.91772322	0.55780207
TCGA.BRCA.1198_LUMINAL_JCI.2020_PMID.32573490	0.11051077	0.59847608	Positive	0.91772322	0.55790047
CIBERSORT_Dendritic_cells_activated_Nat.Methods.2015_PMID.25822800	-0.2822233	-0.5990662	Negative	0.91772322	0.55870342
Unknown_4_BMC.Med.Genomics.2011_PMID.21214954	-0.2916968	-0.598871	Negative	0.91772322	0.55875732
GSEA_PI3K_CASCADE_REACTOME	-0.1766313	-0.5946357	Negative	0.91918686	0.56195493
MM_Green7_BMC.Med.Genomics.2011_PMID.21214954	0.27744689	0.58932974	Positive	0.91918686	0.56513904
HS_Green6_BMC.Med.Genomics.2011_PMID.21214954	-0.2996327	-0.5823411	Negative	0.91918686	0.56933452
CD4_Single_Gene	-0.269691	-0.5822224	Negative	0.91918686	0.56963249
LumA_Basal_JCI.2020_PMID.32573490	0.1741871	0.57889969	Positive	0.91918686	0.57112028
MM_Class8_Genome.Biol.2013_PMID.24220145	-0.3061782	-0.5785949	Negative	0.91918686	0.57112057
New.Immune_Bcell_Tcell_Cooperation_Hollern_Cell_2019_PMID.31730857	0.24301235	0.57854926	Positive	0.91918686	0.57189672
Bcell_Tcell_Cooperation_Cell.2019_PMID.31730857	0.24301235	0.57854926	Positive	0.91918686	0.57189672
Immune_Hot_using_CD8_vs_Cold_PMID.31942071	-0.2984034	-0.5766614	Negative	0.91918686	0.57303281
PR_Isoform_Ratio_Up_in_PRA_H_JNCI.2017_PMID.28376177	0.23296718	0.57479221	Positive	0.91918686	0.57443539
MatureLum_Shehata_BCR.2015_PMID.25575446	-0.1923677	-0.5698411	Negative	0.91918686	0.57809852
Scorr_IIE_Correlation_JCO.2006_PMID.16505416	0.21818467	0.5652034	Positive	0.91918686	0.58069975
MUnknown_30_BMC.Med.Genomics.2011_PMID.21214954	0.20783662	0.56363233	Positive	0.91918686	0.58081885
New.Immune_TCGA.BRCA.1198_immune_CD19_Cell.2015_PMID.26451490	-0.2389445	-0.5605139	Negative	0.91918686	0.58332213
TCGA.BRCA.1198_immune_CD19_JCI.2020_PMID.32573490	-0.2389445	-0.5605139	Negative	0.91918686	0.58332213
T_RM_Cell_single_cell_signature_Savas_Nat_Med.2018_PMID.29942092	-0.2683868	-0.5568088	Negative	0.91918686	0.58599474
IMMUNE_Bindea_Cell_aDC_Immunity.2013_PMID.24138885	0.27194555	0.55746	Positive	0.91918686	0.58611405
MM_Myoepithelioma.like_Genome.Biol.2013_PMID.24220145	-0.3047469	-0.5555881	Negative	0.91918686	0.58617791
LumProg_HsEnriched_Refined1_BCR.2015_PMID.25575446	0.20934025	0.55414994	Positive	0.91918686	0.58768973
Lim2009_MaSC_Adam_PMID.25575446	0.23417124	0.55275638	Positive	0.91918686	0.588348
HS_Green8_BMC.Med.Genomics.2011_PMID.21214954	-0.0992536	-0.5522837	Negative	0.91918686	0.58919317
HouseKeeping_Genome.Biol.2004_PMID.15287981	-0.2549785	-0.5463465	Negative	0.91918686	0.5923655
CIBERSORT_NK_cells_resting_Nat.Methods.2015_PMID.25822800	-0.3042074	-0.5440478	Negative	0.91918686	0.59416185
Pcorr_NKI70_Good_Correlation_Nature.2002_PMID.11823860	-0.1951477	-0.5423698	Negative	0.91918686	0.59503814
MUnknown_20_BMC.Med.Genomics.2011_PMID.21214954	0.1989493	0.54265158	Positive	0.91918686	0.59579876
GSEA_GP2_Immune_Tcell_Bcell.r.0.949_PerouLab_Immune_cell_Cluster_BMC_	-0.281388	-0.5401896	Negative	0.91918686	0.5971344
Chromogramin_BMC.Med.Genomics.2011_PMID.21214954	0.29528996	0.53915358	Positive	0.91918686	0.59767875
UNC_Scorr_LumB_Correlation_JCO.2009_PMID.19204204	-0.1521952	-0.5358781	Negative	0.91918686	0.60056918
MM_Green25_BMC.Med.Genomics.2011_PMID.21214954	-0.2338354	-0.5332085	Negative	0.91918686	0.60121806
FOXC1_Single_Gene	-0.1456785	-0.5317809	Negative	0.91918686	0.60218391
XRT_inducedgenes_Oh_PMID.24527691	0.13005413	0.53265961	Positive	0.91918686	0.60264413
HS_Green16_BMC.Med.Genomics.2011_PMID.21214954	-0.2544031	-0.5319135	Negative	0.91918686	0.60295029
TLS_tumors_w_TLS_and_CD8_v_CD8alone_PMID.31942071	-0.3274857	-0.5304246	Negative	0.91918686	0.60349815

GSEA_GP19_1Q_amplicon.r.0.967_PerouLab_HS_Green17_BMC_Med_Genomic	0.20222425	0.53108792	Positive	0.91918686	0.60397423
MatureLum_Prat_BCR.2015_PMID.25575446	-0.1491731	-0.5290066	Negative	0.91918686	0.60529937
HS_Red20_BMC.Med.Genomics.2011_PMID.21214954	-0.2916794	-0.5240181	Negative	0.91918686	0.60801985
GSEA_PD1_SIGNALING_REACTOME	-0.2935105	-0.5229491	Negative	0.91918686	0.60863666
FOXA1_Single_Gene	-0.1691341	-0.5227889	Negative	0.91918686	0.60908189
HS_Green19_BMC.Med.Genomics.2011_PMID.21214954	0.2793574	0.52231592	Positive	0.91918686	0.60949795
IMMUNE_Bindea_Cell_Lymph_vessels_Immunity.2013_PMID.24138885	-0.2296836	-0.5154305	Negative	0.91918686	0.61349844
VEGF_13genes_BMC.Med.2009_PMID.19291283	0.16915745	0.51621543	Positive	0.91918686	0.61373376
TNBC_Clinically_Relevant_Good.26_BCR.2011_PMID.21978456	0.27731135	0.51570969	Positive	0.91918686	0.61374389
AMPH_EPIREGULIN_Cluster_BMC.Med.Genomics.2011_PMID.21214954	-0.214082	-0.5150436	Negative	0.91918686	0.61421896
MUnknown_33_BMC.Med.Genomics.2011_PMID.21214954	-0.2128927	-0.5134789	Negative	0.91918686	0.61501618
MM_Green14_BMC.Med.Genomics.2011_PMID.21214954	0.22928927	0.5128027	Positive	0.91918686	0.61546641
IMMUNE_Bindea_Cell_NK_cells_Immunity.2013_PMID.24138885	-0.2947968	-0.5109702	Negative	0.91918686	0.61635001
MASC_Down_Nat.Med.2009_PMID.19648928	-0.1837308	-0.5121182	Negative	0.91918686	0.61652621
MUnknown_5_BMC.Med.Genomics.2011_PMID.21214954	0.15585231	0.50868306	Positive	0.91918686	0.61791662
Unknown_5_BMC.Med.Genomics.2011_PMID.21214954	0.20231775	0.5092814	Positive	0.91918686	0.61871836
MITO1_BMC.Med.Genomics.2011_PMID.21214954	-0.2249203	-0.5062915	Negative	0.91918686	0.61996337
New.Immune_ImSig.Tcells_CancerImmunolRes.2018_PMID.30266715	-0.2972853	-0.5048245	Negative	0.91918686	0.62093196
GSEA_GP1_Proliferation_DNA_repair.r.0.981_PUJANA_CHEK2_PCC_NETWORK	-0.2771494	-0.5040239	Negative	0.91918686	0.62145428
Ductal_Carcinoma_In_Situ_J.Pathol.2017_PMID.27861902	0.27167143	0.50247539	Positive	0.91918686	0.62242861
MUnknown_25_BMC.Med.Genomics.2011_PMID.21214954	-0.2344702	-0.5030619	Negative	0.91918686	0.62244132
GSEA_GP7_Estrogen_signaling.r.0.97_SMID_BREAST_CANCER_BASAL_DN	-0.1185444	-0.5018258	Negative	0.91918686	0.62304591
TCGA.BRCA.1198_TP63_JCI.2020_PMID.32573490	0.17627579	0.49929235	Positive	0.91918686	0.62496466
New.Immune_Charoentong_Activated_CD8_T_cell_CellRep.2017_PMID.280522	-0.3251185	-0.4980898	Negative	0.91918686	0.62544321
New.Immune_Charoentong_Type_2_T_helper_cell_CellRep.2017_PMID.280522	0.12431443	0.49821397	Positive	0.91918686	0.62632838
TCGA_CHANG_CORE_SERUM_RESPONSE_UP_Immunity.2018_PMID._2962829(	-0.2317738	-0.4956047	Negative	0.91918686	0.62691134
Stromal_Central_Fibrotic_Focus_J.Pathol.2017_PMID.27861902	-0.1143554	-0.4926509	Negative	0.91918686	0.63018338
CD8A_Single_Gene	-0.2299596	-0.4911078	Negative	0.91918686	0.63086448
MET_UP_RNA_Seq_Significant_Genes_JCI.2018_PMID.29480819	0.17693483	0.49018803	Positive	0.91918686	0.63170882
MERCK_Immune_signature_Science.2018_PMID.30309915	-0.2319396	-0.4897448	Negative	0.91918686	0.63179623
MatureLum_HsEnriched_BCR.2015_PMID.25575446	0.08839033	0.48666556	Positive	0.91918686	0.63341966
aMaSC_Lim09_BCR.2015_PMID.25575446	-0.1929219	-0.4856665	Negative	0.91918686	0.6343982
MUnknown_17_BMC.Med.Genomics.2011_PMID.21214954	0.26623523	0.48253905	Positive	0.91918686	0.63595844
Pcorr_basal_CCR.2010_PMID.20643781	0.17813475	0.48322647	Positive	0.91918686	0.63602559
New.Immune_Tcell_CD8_Exhausted_vs_antiPDL1_2.PMID27789795	0.23958308	0.48204147	Positive	0.91918686	0.63630417
Monocytic_lineage_MCP_PMID.31942075_PMID.31942077	-0.2251083	-0.4824652	Negative	0.91918686	0.63667544
Duke_Module22_tnfa_Mike_PMID.20335537	-0.2104658	-0.4795869	Negative	0.91918686	0.63870234
Pcorr_primitive_CCR.2010_PMID.20643781	-0.1684627	-0.4798077	Negative	0.91918686	0.63873441
TNBC_Clinically_Relevant_Poor.26_BCR.2011_PMID.21978456	0.21968185	0.47689806	Positive	0.91918686	0.63988305
HS_Green3_BMC.Med.Genomics.2011_PMID.21214954	0.18482615	0.47591416	Positive	0.91918686	0.64056872
MM_C3Tag.2012_Genome.Biol.2013_PMID.24220145	0.22677262	0.46761833	Positive	0.91918686	0.6463633
MM_Red25_BMC.Med.Genomics.2011_PMID.21214954	0.23989077	0.46593269	Positive	0.91918686	0.6475436
YALE_RHOA_pathway_Ann.Oncol.2017_PMID.28177460	-0.2400596	-0.4659384	Negative	0.91918686	0.64798116
RB_LOH_Breast.Cancer.Res.2008_PMID.18782450	0.23540077	0.46468381	Positive	0.91918686	0.64841871
Mouse_Human_ImmuneProfiles_SHAY_M_H_Induced_in_HSC_PNAS.2013_PMI	-0.2378143	-0.4647643	Negative	0.91918686	0.64895131
IMS_Score_Clin.Cancer.Res.2018_PMID.29921729	-0.1555612	-0.4638708	Negative	0.91918686	0.65002328
Pcorr_Breast2Lung_LM2_Correlation_Nature.2005_PMID.16049480	-0.2237386	-0.4555768	Negative	0.91918686	0.65481613
New.Immune_Miller_Tcell_NKcell_51gene_PMID.23618380	-0.2812731	-0.4555053	Negative	0.91918686	0.65512902
Late_IRS_1_PLoS.One.2016_PMID.26991655	0.20951231	0.45448234	Positive	0.91918686	0.65558685
IMMUNE_Bindea_Cell_Eosinophils_Immunity.2013_PMID.24138885	-0.2413455	-0.4530459	Negative	0.91918686	0.65717296
GSEA_BIOCARTA_CTLA4_PATHWAY	-0.2577398	-0.4519145	Negative	0.91918686	0.65763242
MCF7.E2.induced.genes_JCO.2006_PMID.16505416	-0.2105323	-0.4502084	Negative	0.91918686	0.65860041
Prosigna_Proliferation_18_BMC.Med.Genomics.2015_PMID.26297356	0.231788	0.44989437	Positive	0.91918686	0.65882206
New.Immune_TCGA.BRCA.1198_immune_GIMAP_IL16_Cell.2015_PMID.26451	-0.2702156	-0.4496497	Negative	0.91918686	0.65920839
TCGA.BRCA.1198_immune_GIMAP_IL16_JCI.2020_PMID.32573490	-0.2702156	-0.4496497	Negative	0.91918686	0.65920839
LumA_LumB_JCI.2020_PMID.32573490	0.23800846	0.44911556	Positive	0.91918686	0.65937194
Knudsen_Neo_common_Clin.Cancer.Res.2014_PMID.25047707	0.22341462	0.44911464	Positive	0.91918686	0.65937258
IntClust_Amplification.50.Better.than_Genome.Biol.2014_PMID.25164602	-0.2188171	-0.4414412	Negative	0.91918686	0.6656709
MM_Green19_BMC.Med.Genomics.2011_PMID.21214954	0.22167846	0.44021094	Positive	0.91918686	0.66567328
Pcorr_bronchioid_PLOS.2012_PMID.22590557	-0.2227754	-0.4373351	Negative	0.91918686	0.66771394
HS_Green10_BMC.Med.Genomics.2011_PMID.21214954	0.22397169	0.43698411	Positive	0.91918686	0.66796318
Duke_Module17_pr_Mike_PMID.20335537	-0.1419066	-0.4299538	Negative	0.91918686	0.67381994
MM_Red14_BMC.Med.Genomics.2011_PMID.21214954	-0.147793	-0.4287116	Negative	0.91918686	0.67465125
Scorr_S329_R_Correlation_Br.J.Cancer.2008_PMID.18382427	-0.1670464	-0.428706	Negative	0.91918686	0.67473648
MUnknown_34_BMC.Med.Genomics.2011_PMID.21214954	-0.1964317	-0.4258597	Negative	0.91918686	0.67646563
CIBERSORT_NK_cells_activated_Nat.Methods.2015_PMID.25822800	-0.2477979	-0.4236811	Negative	0.91918686	0.67769846
LOBULAR_TCGA_SUBTYPE_Proliferative_Cell.2015_PMID.26451490	0.15988727	0.42315955	Positive	0.91918686	0.67835549
TLS_Known_MARKERS_PMID.31942071	-0.2514945	-0.4201664	Negative	0.91918686	0.6802274
Miller_Proliferation_Metagene_Genome_Biol_2013_PMID.23618380	0.21377938	0.41923043	Positive	0.91918686	0.68062212
MNB2_BMC.Med.Genomics.2011_PMID.21214954	0.13633548	0.41961011	Positive	0.91918686	0.68096756
LCK_Breast.Cancer.Research.2008_PMID.19272155	-0.2386691	-0.4178197	Negative	0.91918686	0.68194777
HS_Red23_BMC.Med.Genomics.2011_PMID.21214954	0.21854	0.41729686	Positive	0.91918686	0.68200689
Stromal_Inflammation_J.Pathol.2017_PMID.27861902	-0.1930896	-0.4163302	Negative	0.91918686	0.68323443
CIBERSORT_T_cells_gamma_delta_Nat.Methods.2015_PMID.25822800	-0.2328538	-0.414415	Negative	0.91918686	0.68429443
IMMUNE_PEREZ_87_JCO.2015_PMID.2560586	-0.2421085	-0.4138454	Negative	0.91918686	0.68487579
CIBERSORT_B_cells_naive_Nat.Methods.2015_PMID.25822800	-0.2128663	-0.4118877	Negative	0.91918686	0.68640629
New.Immune_ImSig.Bcells_CancerImmunolRes.2018_PMID.30266715	-0.1737258	-0.4104796	Negative	0.91918686	0.68750482
CD3E_Single_Gene	-0.2476361	-0.4029255	Negative	0.91918686	0.69246324
MM_Green23_BMC.Med.Genomics.2011_PMID.21214954	0.18263777	0.40308191	Positive	0.91918686	0.69275891
TCGA_Tgd_cells_Immunity.2018_PMID._29628290	0.21366769	0.40197652	Positive	0.91918686	0.69301999
IMMUNE_Bindea_Cell_Tgd_Immunity.2013_PMID.24138885	0.21366769	0.40197652	Positive	0.91918686	0.69301999
HS_Green23_BMC.Med.Genomics.2011_PMID.21214954	-0.2372777	-0.3988891	Negative	0.91918686	0.69524816
Duke_Module12_lacticacidosis_Mike_PMID.20335537	-0.1320368	-0.3999051	Negative	0.91918686	0.69548664
CIBERSORT_T_cells_regulatory_Tregs_Nat.Methods.2015_PMID.25822800	-0.2325214	-0.3983795	Negative	0.91918686	0.69597034
T_cell_activation_PMID.31942077	0.15217339	0.39885064	Positive	0.91918686	0.69616043
JANES_Oscillation_GDF11_TGFBR3_Nat.Cell.Biol.2014_PMID.24658685	0.22272291	0.39469308	Positive	0.91918686	0.69849834
PIK3CA.Hutti_Cancer.Res.2012_PMID.22552288	-0.1678046	-0.3938624	Negative	0.91918686	0.69888201
New.Immune_ImmuneActive_Hollern_Cell_2019_PMID.31730857	-0.2220287	-0.3935087	Negative	0.91918686	0.69952696
DAN_ImmuneActive_Cell.2019_PMID.31730857	-0.2220287	-0.3935087	Negative	0.91918686	0.69952696
CD3D_Single_Gene	-0.2402531	-0.3885938	Negative	0.91918686	0.70269884
MM_DMBAwnt_1pFDR_UP_Genome.Biology.2007_PMID.17493263	-0.2071634	-0.3867965	Negative	0.91918686	0.704119

Duke_Module01_acidosis_Mike_PMID.20335537	-0.2350864	-0.3864196	Negative	0.91918686	0.70443666
Glycolysis_Signature_BMC.Med.2009_PMID.19291283	0.13774619	0.38642887	Positive	0.91918686	0.70498896
LumA_Her2_JCI.2020_PMID.32573490	0.15385615	0.38418786	Positive	0.91918686	0.70589711
Unknown_9_BMC.Med.Genomics.2011_PMID.21214954	0.15793093	0.38470009	Positive	0.91918686	0.70634564
bMYB_Signature_Oncogene.2009_PMID.19043454	0.19769692	0.38300407	Positive	0.91918686	0.70675739
MM_p53null.Basal_Genome.Biol.2013_PMID.24220145	0.1372559	0.38115661	Positive	0.91918686	0.70837334
UNC_Scorr_Norm_Correlation_JCO.2009_PMID.19204204	-0.1621609	-0.3796601	Negative	0.91918686	0.7095615
HS_Green25_BMC.Med.Genomics.2011_PMID.21214954	-0.0515077	-0.378077	Negative	0.91918686	0.71034235
Bcell_IL10_MINUS_LIN_J_Immunol.2014_PMID.25080484	-0.1554607	-0.3750758	Negative	0.91918686	0.7129462
Scorr_IE_Correlation_JCO.2006_PMID.16505416	-0.1587064	-0.373474	Negative	0.91918686	0.71399218
New.Immune_Charoentong_Type_17_T_helper_cell_CellRep.2017_PMID.28052	-0.1264645	-0.3737524	Negative	0.91918686	0.7143003
Wirapati_Proliferation_Breast.Cancer.Res.2008_PMID.18662380	0.19554477	0.37222328	Positive	0.91918686	0.71461073
Bcell_IL10_PLUS_LIN_J_Immunol.2014_PMID.25080484	-0.1938882	-0.3723779	Negative	0.91918686	0.71499076
MS_CD44_UP_PNAS.2009_PMID.19666588	-0.1509497	-0.3711442	Negative	0.91918686	0.71590586
NK_cells_MCP_PMID.31942075_PMID.31942077	-0.1602658	-0.3687521	Negative	0.91918686	0.71757487
Shehata2012_ALDHneg_PMID.25575446	0.12552563	0.36794695	Positive	0.91918686	0.71868682
UNC_Proliferation_11_Mean_JCO.2009_PMID.19204204	0.18763692	0.36590045	Positive	0.91918686	0.71923217
New.Immune_Charoentong_Activated_B_cell_CellRep.2017_PMID.28052254	-0.169306	-0.3659746	Negative	0.91918686	0.71982703
New.Immune_MHCI_coreGenes_PMID29170503	0.14481348	0.36378081	Positive	0.91918686	0.72153042
HS_Green21_BMC.Med.Genomics.2011_PMID.21214954	0.13593745	0.36129564	Positive	0.91918686	0.72309573
HGF_down_BCR.2013_PMID.24025166	-0.1835317	-0.3586226	Negative	0.91918686	0.72508178
New.Immune_Miller_T_NK_Metagene_34gene_Genome_Biol_2013_PMID.2361	-0.2093111	-0.3571959	Negative	0.91918686	0.72591926
Miller_T_NK_Metagene_Genome_Biol_2013_PMID.23618380	-0.2093111	-0.3571959	Negative	0.91918686	0.72591926
Endothelial_Tumor_EC_high_Angiogenesis_2014_PMID.24257808	0.21058215	0.35665782	Positive	0.91918686	0.72600797
TNBC_Clinically_Relevant_Good.230_BCR.2011_PMID.21978456	-0.1181969	-0.3547714	Negative	0.91918686	0.72739382
CIBERSORT_B_cells_memory_Nat.Methods.2015_PMID.25822800	-0.1767315	-0.355098	Negative	0.91918686	0.72769991
New.Immune_Tcell_CD8_Exhausted_vs_naive_2.PMID27789795	0.18712631	0.35358034	Positive	0.91918686	0.72826935
New.Immune_ImSig.ProliferationPathway_CancerImmunolRes.2018_PMID.302(	0.18012138	0.35012679	Positive	0.91918686	0.73081015
MM_Wnt1.Late_Genome.Biol.2013_PMID.24220145	-0.1983062	-0.3483777	Negative	0.91918686	0.73209818
New.Immune_Tcell_CD8_Effector_vs_naive_2.PMID27789795	0.181264	0.34564351	Positive	0.91918686	0.73411336
Shehata2012_NCL_PMID.25575446	0.0595062	0.34662395	Positive	0.91918686	0.73418051
Response_to_PARP_inhibitor_olaparib_Sensitivity_BC_Res_Treat_2012_PMID.2	-0.1592743	-0.3439989	Negative	0.91918686	0.73542754
MET_UP_Significant_Genes_HIGH_BASALS_Genes_JCI.2018_PMID.29480819	-0.0936523	-0.3444024	Negative	0.91918686	0.73593639
IMMUNE_Bindea_Cell_Th1_cells_Immunity.2013_PMID.24138885	0.18728421	0.3434516	Positive	0.91918686	0.73640766
MM_Red16_BMC.Med.Genomics.2011_PMID.21214954	-0.1607038	-0.3414967	Negative	0.91918686	0.737397
MUnknown_9_BMC.Med.Genomics.2011_PMID.21214954	-0.1985485	-0.3393707	Negative	0.91918686	0.73874411
MCD3_CD8_BMC.Med.Genomics.2011_PMID.21214954	-0.1972735	-0.338748	Negative	0.91918686	0.73947016
Proliferation_Cluster_BMC.Med.Genomics.2011_PMID.21214954	0.17254108	0.33744866	Positive	0.91918686	0.74016512
Up_regulated_by_Oncogenic_N_RAS_Cell_Rep.2015_PMID.26166574	0.1865005	0.33725898	Positive	0.91918686	0.7405099
RB_LOSS_J.Clin.Invest.2007_PMID.17160137	0.17649246	0.33633043	Positive	0.91918686	0.74099229
TCGA.BRCA.1198_HISTONES_JCI.2020_PMID.32573490	-0.1686046	-0.3362296	Negative	0.91918686	0.74106692
GSEA_BIOCARTA_VEGF_PATHWAY	-0.1919962	-0.3326875	Negative	0.92020454	0.74405286
New.Immune_Charoentong_Immature__B_cell_CellRep.2017_PMID.28052254	-0.1931896	-0.3298697	Negative	0.92020454	0.74610837
Lobular_Carcinoma_In_Situ_J.Pathol.2017_PMID.27861902	-0.1592692	-0.3286226	Negative	0.92020454	0.74670276
GSEA_GP13_Neural_signailng.r.0.959_MODULE_100	0.20483234	0.32849591	Positive	0.92020454	0.74691148
MM_Red10_BMC.Med.Genomics.2011_PMID.21214954	0.15658129	0.32650094	Positive	0.92020454	0.74835251
TLS_Hallmark_Gene_Signature_PMID.31942071	0.2076754	0.32664856	Positive	0.92020454	0.74849173
MProliferation_BMC.Med.Genomics.2011_PMID.21214954	0.17093046	0.32455988	Positive	0.92035977	0.7497189
FOXC1_Hair_Follicles_Wang_SCIENCE.2016_P30C.LO.vs.WT_Negative_PMID.26	0.18827255	0.32030192	Positive	0.92301825	0.75298858
CTLA4_Single_Gene	-0.1440498	-0.3184331	Negative	0.92352007	0.75450264
C3_TAG_UNTREATED_Usary_Clin.Cancer.Res.2013_PMID.23780888	0.16724946	0.31415843	Positive	0.9250744	0.75745986
MatureLum_HsEnriched_Refined1_BCR.2015_PMID.25575446	-0.0368271	-0.3127803	Negative	0.9250744	0.75878228
HS_Green18_BMC.Med.Genomics.2011_PMID.21214954	0.07527315	0.31124036	Positive	0.9250744	0.75988976
Unknown_7_BMC.Med.Genomics.2011_PMID.21214954	0.1340733	0.31085586	Positive	0.9250744	0.7605094
IMMUNE_Bindea_Cell_TReg_Immunity.2013_PMID.24138885	0.12924749	0.3093389	Positive	0.9250744	0.76130524
REPLICATION_STRESS_POS_Cell.Rep.2018_PMID.29768207	-0.1134394	-0.3037579	Negative	0.92788156	0.76580063
IMMUNE_Bindea_Cell_Th17_cells_Immunity.2013_PMID.24138885	-0.1200153	-0.3036167	Negative	0.92788156	0.76583526
STAT3_Basal_Horvath_SHORT_PNAS.2014_PMID.25139989	-0.1336988	-0.3012595	Negative	0.9288053	0.76770869
MPYMT_NEU_Cluster_BMC.Med.Genomics.2011_PMID.21214954	0.11379041	0.29698473	Positive	0.93071625	0.77040149
GATA3.induced.genes_JCO.2006_PMID.16505416	0.08669769	0.29430312	Positive	0.93167576	0.77231017
HS_Red9_BMC.Med.Genomics.2011_PMID.21214954	-0.0948644	-0.2902496	Negative	0.93321968	0.77573223
Luminal_Progenitor_Up_Nat.Med.2009_PMID.19648928	0.11181147	0.28984903	Positive	0.93321968	0.77582258
CIBERSORT_T_cells_follicular_helper_Nat.Methods.2015_PMID.25822800	-0.1854052	-0.2865589	Negative	0.93473184	0.77833905
HS_Red13_BMC.Med.Genomics.2011_PMID.21214954	0.11540289	0.28515113	Positive	0.93473184	0.7793159
Spike2012_aMaSC_PMID.25575446	0.10403756	0.28220127	Positive	0.93537904	0.7819426
T_Cell_cluster_Iglesia_CCR.2014_PMID.24916698	-0.1624771	-0.2800359	Negative	0.93537904	0.78319721
Fibrinogen_Cluster_BMC.Med.Genomics.2011_PMID.21214954	0.07504565	0.28075296	Positive	0.93537904	0.78321211
Tumour_hypoxia_causes_DNA_hypermethylation_by_reducing_TET_activity_Na	-0.0947152	-0.2776268	Negative	0.93600291	0.78485412
HS_Green13_BMC.Med.Genomics.2011_PMID.21214954	0.10794983	0.27309666	Positive	0.93945823	0.78887521
HISTONE_BMC.Med.Genomics.2011_PMID.21214954	-0.1496685	-0.2707125	Negative	0.93954666	0.79007333
MM_WAPTag_1pFDR_UP_Genome.Biology.2007_PMID.17493263	0.13404077	0.26759871	Positive	0.93978063	0.79242714
IMMUNE_PEREZ_14_JCO.2015_PMID.2560586	-0.1503341	-0.2668321	Negative	0.93978063	0.79318733
CIBERSORT_T_cells_CD4_naive_Nat.Methods.2015_PMID.25822800	-0.1595708	-0.2633461	Negative	0.93978063	0.79579551
New.Immune_ImmLandscape_IFNG_5.PMID.24516633	-0.1364391	-0.2630348	Negative	0.93978063	0.79621009
Endothelial_Normal_EC_high_Angiogenesis_2014_PMID.24257808	0.15550923	0.26065501	Positive	0.93978063	0.79768353
New.Immune_Charoentong_CD56dim_natural_killer_cell_CellRep.2017_PMID.2	-0.0830699	-0.2611175	Negative	0.93978063	0.79795093
IMMUNE_Bindea_Cell_DC_Immunity.2013_PMID.24138885	-0.1485692	-0.2600539	Negative	0.93978063	0.79813905
MM_Squamous.like_Genome.Biol.2013_PMID.24220145	-0.0915019	-0.2562941	Negative	0.94005956	0.80142859
MM_Myc.2012_Genome.Biol.2013_PMID.24220145	-0.1119693	-0.2548412	Negative	0.94005956	0.80233333
ACTIVATED_CANCER_CELL_SIGNATURE_Nat.Cell.Biol.2019_PMID.31263265	-0.1275046	-0.2523159	Negative	0.94005956	0.80418416
MM_Neu.2012_Genome.Biol.2013_PMID.24220145	0.09223662	0.25128884	Positive	0.94005956	0.80478966
New.Immune_TCGA.BRCA.1198_immune_CD8_GZMK_Cell.2015_PMID.264514	-0.1486235	-0.25101	Negative	0.94005956	0.80512278
TCGA.BRCA.1198_immune_CD8_GZMK_JCI.2020_PMID.32573490	-0.1486235	-0.25101	Negative	0.94005956	0.80512278
IMMUNE_Bindea_Cell_T_cells_Immunity.2013_PMID.24138885	-0.1552403	-0.2489947	Negative	0.94045914	0.80658996
Troester_Wound_Signature_Clin.Cancer.Res.2009_PMID.19887484	-0.1192911	-0.2421199	Negative	0.94106443	0.81203253
MUnknown_16_BMC.Med.Genomics.2011_PMID.21214954	-0.107648	-0.2407021	Negative	0.94106443	0.8131982
CIBERSORT_Plasma_cells_Nat.Methods.2015_PMID.25822800	-0.101572	-0.2394346	Negative	0.94106443	0.81431878
CORE_B_CELL_signatures_Garber_Cell.Mol.Gastroenterol.Hepatol.2017_PMID.2	-0.1099526	-0.2390159	Negative	0.94106443	0.81454692
aMaSC_HsEnriched_BCR.2015_PMID.25575446	-0.1034515	-0.235124	Negative	0.94106443	0.81709526
Lim2010_LumProg_Adam_PMID.25575446	0.13010359	0.23176634	Positive	0.94106443	0.81975523
Duke_Module15_p63_Mike_PMID.20335537	0.11374425	0.23156741	Positive	0.94106443	0.82011598

Unknown_11_BMC.Med.Genomics.2011_PMID.21214954	0.11521247	0.23019895	Positive	0.94106443	0.82097854
MIR_200c_Induced_ONCO.2015_PMID.25746005	0.11965071	0.2291217	Positive	0.94106443	0.82176852
LumProg_HsEnriched_BCR.2015_PMID.25575446	-0.0913123	-0.2274935	Negative	0.94106443	0.82292153
CD8_cluster_Iglesia_CCR.2014_PMID.24916698	-0.137271	-0.2275731	Negative	0.94106443	0.82294931
fMaSC_Metab8_CellRep.2018_PMID.30089273	-0.0740496	-0.2273734	Negative	0.94106443	0.82301326
Shehata2012_Erbb3neg_PMID.25575446	0.11946478	0.22683358	Positive	0.94106443	0.82379847
Duke_Module08_her2_Mike_PMID.20335537	-0.0924883	-0.2262835	Negative	0.94106443	0.82390203
CIBERSORT_T_cells_CD8_Nat.Methods.2015_PMID.25822800	-0.1322714	-0.2237019	Negative	0.94106443	0.8259539
High_In_No_Response_MCP_TLS_Melanoma_PMID.31942075	0.14849719	0.22261526	Positive	0.94106443	0.82673735
GSEA_BIOCARTA_BRCA_ATR_PATHWAY_ATRBRCA	0.12669321	0.22187738	Positive	0.94106443	0.82730397
Pcorr_CSR_Activated_Correlation_PNAS.2005_PMID.15701700	0.10947554	0.22087602	Positive	0.94106443	0.82798293
Pcorr_Breast2Lung_Parental_Correlation_Nature.2005_PMID.16049480	-0.101385	-0.2205408	Negative	0.94106443	0.82849691
MHC.I_Breast.Cancer.Research.2008_PMID.19272155	-0.0980251	-0.2178928	Negative	0.94142765	0.83068347
MUnknown_32_BMC.Med.Genomics.2011_PMID.21214954	0.1021186	0.21706306	Positive	0.94142765	0.83106891
MUnknown_27_BMC.Med.Genomics.2011_PMID.21214954	-0.1013278	-0.2140735	Negative	0.94170951	0.83328906
REPLICATION_STRESS_UP_SET_Cell.Rep.2018_PMID.29768207	-0.0788082	-0.2133525	Negative	0.94170951	0.83413863
Lums_HER2E_DOWN_metastatic.signature_JCI.2020_PMID.32573490	-0.1117169	-0.2121135	Negative	0.94170951	0.83469707
CIBERSORT_T_cells_CD4_memory_resting_Nat.Methods.2015_PMID.25822800	-0.1296409	-0.208134	Negative	0.9437987	0.83784642
HS_Red15_BMC.Med.Genomics.2011_PMID.21214954	0.0772093	0.20736956	Positive	0.9437987	0.83880674
Duke_Module03_betacatenin_Mike_PMID.20335537	-0.09896	-0.2045906	Negative	0.94440121	0.84047189
Durvalumab_signature_Higgs_Clin.Cancer.Res.2018_PMID.29716923	-0.0558791	-0.1964955	Negative	0.94875648	0.84721151
Stingl_Day7_Upregulated_Nat.Cell.Biol.2014_PMID.25173976	0.09295	0.1897942	Positive	0.94875648	0.85185734
MNOtch4_BMC.Med.Genomics.2011_PMID.21214954	0.07560011	0.18895403	Positive	0.94875648	0.8526451
Duke_Module10_ifna_Mike_PMID.20335537	0.07827162	0.18797504	Positive	0.94875648	0.85364731
MM_Green2_BMC.Med.Genomics.2011_PMID.21214954	0.09362198	0.186825	Positive	0.94875648	0.85432296
New.Immune_Charoentong_Activated_CD4_T_cell_CellRep.2017_PMID.280522	0.09486069	0.18618026	Positive	0.94875648	0.85475144
Taube_EMT_down_PNAS.2010_PMID.20713713	-0.075628	-0.1860151	Negative	0.94875648	0.85480339
FOXC1_Hair_Follicles_Wang_SCIENCE.2016_P30C.LO.vs.WT_Positive_PMID.269	0.09107709	0.18610013	Positive	0.94875648	0.85498667
HER2_AMPLIFIED_PerouLab_HER2_Amplicon_BMC_Med_Genomics_2011_PMI	0.03945029	0.18504307	Positive	0.94875648	0.85563314
MDACC_P53_ER.Pos_CCR.2011_PMID.21248301	0.09492031	0.18481477	Positive	0.94875648	0.85569664
HS_Green24_BMC.Med.Genomics.2011_PMID.21214954	0.06442158	0.18355641	Positive	0.94893838	0.85699579
MUnknown_29_BMC.Med.Genomics.2011_PMID.21214954	-0.0818646	-0.1789753	Negative	0.95123067	0.86020381
Shehata2012_Basal_PMID.25575446	-0.0803615	-0.1769819	Negative	0.95167459	0.86174362
LumProg_Lim09_BCR.2015_PMID.25575446	-0.0704933	-0.1755772	Negative	0.95172698	0.86292949
HS_Red18_BMC.Med.Genomics.2011_PMID.21214954	0.06224203	0.17437672	Positive	0.95176259	0.86412874
MM_Green16_BMC.Med.Genomics.2011_PMID.21214954	0.07151923	0.17275245	Positive	0.95176259	0.86523872
CIBERSORT_Macrophages_M1_Nat.Methods.2015_PMID.25822800	-0.0812933	-0.1706888	Negative	0.95237517	0.86693482
CIN70_Nat.Genet.2006_PMID.16921376	0.084604	0.16746133	Positive	0.9533439	0.86910536
Lim2009_LumProg_Adam_PMID.25575446	0.06006308	0.16617968	Positive	0.9533439	0.87009737
Histological_Grade_J.Pathol.2017_PMID.27861902	0.08301862	0.16157931	Positive	0.95376534	0.87365991
New.Immune_TCGA.BRCA.1198_immune_CTLA4_CXCL_FOXP3_Cell.2015_PMID	0.06473531	0.16167763	Positive	0.95376534	0.8739046
TCGA.BRCA.1198_immune_CTLA4_CXCL_FOXP3_JCI.2020_PMID.32573490	0.06473531	0.16167763	Positive	0.95376534	0.8739046
MatureLum_Lim09_BCR.2015_PMID.25575446	0.03278409	0.15808102	Positive	0.95464147	0.87664959
CIBERSORT_T_cells_CD4_memory_activated_Nat.Methods.2015_PMID.258228	-0.0871573	-0.1574664	Negative	0.95464147	0.87699121
MM_Red5_BMC.Med.Genomics.2011_PMID.21214954	-0.0716806	-0.1502008	Negative	0.95942337	0.88253178
HS_Red19_BMC.Med.Genomics.2011_PMID.21214954	-0.0439352	-0.143332	Negative	0.96391625	0.8878176
MDSC_Neutrophil_Youn_J_Leukoc_Biol.2012_PMID.21954284	-0.0490204	-0.140173	Negative	0.96556437	0.89049058
MUnknown_1_BMC.Med.Genomics.2011_PMID.21214954	0.05610512	0.13474139	Positive	0.96883888	0.8946694
X19p13_Amplicon_BMC.Med.Genomics.2011_PMID.21214954	-0.0677337	-0.1310422	Negative	0.96900533	0.89749434
CD8_T_cells_MCP_.31942075_PMID.31942077	0.04838371	0.13036216	Positive	0.96900533	0.89819855
MM_Stat1_Genome.Biol.2013_PMID.24220145	-0.0542826	-0.1280668	Negative	0.96900533	0.89969199
B_Cell_cluster_Iglesia_CCR.2014_PMID.24916698	0.05295751	0.12753587	Positive	0.96900533	0.90025533
Duke_Module11_ifng_Mike_PMID.20335537	0.05564188	0.12579392	Positive	0.96900533	0.90170696
GSEA_GP13_Neural_signailng.r.0.894_NERVOUS_SYSTEM_DEVELOPMENT	-0.0633877	-0.1228717	Negative	0.96900533	0.90373886
IntClust_Amplification.50_Genome.Biol.2014_PMID.25164602	-0.0592759	-0.1185922	Negative	0.96900533	0.9072442
Necrosis_J.Pathol.2017_PMID.27861902	0.06338187	0.11707764	Positive	0.96900533	0.90843481
MICRORNA_138_Signature_Cancer.Res.2014_PMID.25339353	-0.0498369	-0.1166751	Negative	0.96900533	0.90862837
New.Immune_ImmLandscape_Wound_Healing.PMID.14737219	-0.0558492	-0.1158201	Negative	0.96900533	0.90923632
X17q25x_BMC.Med.Genomics.2011_PMID.21214954	-0.0427817	-0.1158672	Negative	0.96900533	0.90948225
BPMS_7.Genes_PLoS.One.2013_PMID.24349199	0.05159562	0.11546047	Positive	0.96900533	0.90956848
IGG_Cluster_BMC.Med.Genomics.2011_PMID.21214954	0.0539702	0.11227097	Positive	0.96900533	0.91214604
T.cells_MCP_PMID.31942075_PMID.31942077	-0.0673955	-0.1120841	Negative	0.96900533	0.91220814
fMaSC_Metab_CellRep.2018_PMID.30089273	0.05165954	0.11200886	Positive	0.96900533	0.91220956
HS_Red1_BMC.Med.Genomics.2011_PMID.21214954	-0.0451185	-0.1066691	Negative	0.97092666	0.91640342
HS_Green5_BMC.Med.Genomics.2011_PMID.21214954	0.05085936	0.10560011	Positive	0.97092666	0.91724631
C3_TAG_RESPONDING_Usary_Clin.Cancer.Res.2013_PMID.23780888	0.04234308	0.10410159	Positive	0.97092666	0.9183825
Unknown_16_BMC.Med.Genomics.2011_PMID.21214954	-0.0571863	-0.1038508	Negative	0.97092666	0.91875653
MM_Wnt1.Early_Genome.Biol.2013_PMID.24220145	0.05141038	0.10180383	Positive	0.97092666	0.92030979
Nuclear_Pleomorphism_J.Pathol.2017_PMID.27861902	0.04642402	0.10064016	Positive	0.97092666	0.92108643
Luminal_Progenitor_Down_Nat.Med.2009_PMID.19648928	-0.0451934	-0.0993177	Negative	0.97092666	0.92214805
MYC_Single_Gene	0.03600399	0.09715764	Positive	0.97159781	0.92394768
MM_Green6_BMC.Med.Genomics.2011_PMID.21214954	0.04401846	0.09108241	Positive	0.97319356	0.92855768
New.Immune_TCGA.BRCA.1198_immune_HLA_A_F_Cell.2015_PMID.26451490	-0.0381665	-0.0907949	Negative	0.97319356	0.92895749
TCGA.BRCA.1198_immune_HLA_A_F_JCI.2020_PMID.32573490	-0.0381665	-0.0907949	Negative	0.97319356	0.92895749
New.Immune_ImmLandscape_lymphocyte_Infil_T.B.PMID.18592372	-0.0470938	-0.086944	Negative	0.97381459	0.93187999
TCGA_LIexpression_score_Immunity.2018_PMID._29628290	-0.0470938	-0.086944	Negative	0.97381459	0.93187999
MUnknown_8_BMC.Med.Genomics.2011_PMID.21214954	-0.0516689	-0.0849997	Negative	0.97415182	0.93336796
HS_Red6_BMC.Med.Genomics.2011_PMID.21214954	-0.0395285	-0.0797576	Negative	0.97500085	0.93741909
HS_Green12_BMC.Med.Genomics.2011_PMID.21214954	-0.0282663	-0.0790242	Negative	0.97500085	0.93813128
CD19_Single_Gene	0.03576007	0.07880231	Positive	0.97500085	0.93824889
Up_regulated_upon_N_RAS_repression_Cell_Rep.2015_PMID.26166574	0.03441728	0.07793445	Positive	0.97500085	0.93884651
HER1_Cluster1_BMC.Genomics.2007_PMID.17663798	0.02489385	0.06434561	Positive	0.98483301	0.94949211
GSEA_GP1_Proliferation_DNA_repair.r.0.972_REACTOME_CELL_CYCLE_MITOTI	0.03322248	0.0620044	Positive	0.98533321	0.95134581
New.Immune_Tertiary.Lymphoid.Structure.12chemokine_FrontImmunol.2017.P	-0.0321263	-0.060838	Negative	0.98533321	0.95233162
HS_Red12_BMC.Med.Genomics.2011_PMID.21214954	0.02239663	0.05461176	Positive	0.9891015	0.95715683
HRAS_Single_Gene	0.01734044	0.04631056	Positive	0.99461744	0.96368436
Epithelial_Tubule_Formation_J.Pathol.2017_PMID.27861902	-0.0142388	-0.0407613	Negative	0.99514136	0.96805389
MM_Green22_BMC.Med.Genomics.2011_PMID.21214954	0.0152165	0.03997766	Positive	0.99514136	0.96865212
TCGA.BRCA.1198_HOXC11_HOTAIR_SIX1_JCI.2020_PMID.32573490	0.02120738	0.0396717	Positive	0.99514136	0.96884567
REPLICATION_STRESS_DOWN_SET_Cell.Rep.2018_PMID.29768207	0.01351062	0.0395344	Positive	0.99514136	0.96895343
GSEA_CASPASE_CASCADE_APOPTOSIS_SA	-0.0196384	-0.0374595	Negative	0.99559645	0.97061032

MM_C3Tag_1pFDR_UP_Genome.Biology.2007_PMID.17493263	-0.0143651	-0.0331434	Negative	0.99559645	0.97399004
New.Immune_TCGA.BRCA.1198_IMMUNE1_Cell.2015_PMID.26451490	-0.0158551	-0.0284115	Negative	0.99559645	0.97771513
TCGA.BRCA.1198_IMMUNE1_JCI.2020_PMID.32573490	-0.0158551	-0.0284115	Negative	0.99559645	0.97771513
CD103_Positive_Cancer.Cell.2014_PMID.25446897	-0.0180402	-0.026699	Negative	0.99559645	0.97904974
New.Immune_Charoentong_Memory_B_cell_CellRep.2017_PMID.28052254	-0.0181677	-0.0259439	Negative	0.99559645	0.97962292
Response_to_PARP_inhibitor_olaparib_Resistance_BC_Res_Treat_2012_PMID.2	-0.0127982	-0.0246593	Negative	0.99559645	0.98067124
Scorr_S329_L_Correlation_Br.J.Cancer.2008_PMID.18382427	0.01093067	0.02417162	Positive	0.99559645	0.98104336
LumProg_Prat_BCR.2015_PMID.25575446	-0.0096508	-0.0240439	Negative	0.99559645	0.9811149
Wahl_aMaSC_Signature_Cell.Stem.Cell.2012_PMID.22305568	-0.0080978	-0.0238722	Negative	0.99559645	0.98130559
Lim2010_MaSC_Adam_PMID.25575446	0.00901937	0.02150192	Positive	0.99586328	0.9831424
MDSC_Granulocytic_Youn_J_Leukoc_Biol.2012_PMID.21954284	-0.0100016	-0.020465	Negative	0.99586328	0.98395104
Mitotic_Count_J.Pathol.2017_PMID.27861902	0.00985054	0.01748627	Positive	0.99689901	0.98626489
B_lineage_MCP_PMID.31942075_PMID.31942077	0.00672451	0.015847	Positive	0.99689901	0.98756845
Unknown_8_BMC.Med.Genomics.2011_PMID.21214954	0.00497176	0.01414298	Positive	0.99689901	0.98891459
REPLICATION_STRESS_NEG_Cell.Rep.2018_PMID.29768207	-0.0043666	-0.0120146	Negative	0.99689901	0.99056254
Pfefferle2012_LumProg_PMID.25575446	-0.0039146	-0.0109601	Negative	0.99689901	0.99139075
MIR_200c_Repressed_ONCO.2015_PMID.25746005	0.0041205	0.00806789	Positive	0.99689901	0.99366905
Tcell_EXH_Anti_PDL1_vs_control_treated_exhausted_CD8_T_cell_Metagene_1_	-0.0030584	-0.007814	Negative	0.99689901	0.99387916
MUnknown_7_BMC.Med.Genomics.2011_PMID.21214954	-0.0030386	-0.0069891	Negative	0.99689901	0.99451409
HER1_Cluster2_BMC.Genomics.2007_PMID.17663798	-0.0003988	-0.0008904	Negative	0.99953308	0.9993008
Immune_High_In_Response_MCP_TLS_Melanoma_PMID.31942075	0.00028276	0.00059551	Positive	0.99953308	0.99953308

Supplementary Data 4

Linear mixed model - RNA - Basal-like samples

Signatures	B_value	T.value	dir	P.adj	P.value
KRAS_amplicon_Genome.Biology.2007_PMID.17493263	-1.265236	-4.0287413	Negative	0.87779092	0.00136715
MKRAS_amplicon_BMC.Med.Genomics.2011_PMID.21214954	-1.0848595	-3.2581681	Negative	0.87779092	0.00596289
GSEA_RESPONSE_TO_ANDROGEN_UP_NELSON	-0.80534	-3.2362742	Negative	0.87779092	0.00618154
MUnknown_14_BMC.Med.Genomics.2011_PMID.21214954	-1.0288337	-3.156562	Negative	0.87779092	0.00730089
CDKN2A_Single_Gene	0.83306434	3.03825894	Positive	0.87779092	0.0093472
IMMUNE_Bindea_Cell_CD8_T_cells_Immunity.2013_PMID.24138885	-1.2995224	-2.7894125	Negative	0.87779092	0.01456983
Duke_Module07_glucosedepletion_Mike_PMID.20335537	-0.7908663	-2.7981667	Negative	0.87779092	0.01474868
LOBULAR_TCGA_SUBTYPE_Immune_related_Cell.2015_PMID.26451490	-0.8280595	-2.7232089	Negative	0.87779092	0.01573955
KRAS_Single_Gene	-0.8927484	-2.6798448	Negative	0.87779092	0.01847786
XBP1_Signature_Nature.2014_PMID.24670641	-0.8569227	-2.6346881	Negative	0.87779092	0.02003643
MUnknown_35_BMC.Med.Genomics.2011_PMID.21214954	0.85702218	2.52997873	Positive	0.87779092	0.02482683
MET_DOWN_Significant_Genes_LOW_BASALS_2_Genes_JCI.2018_PMID.29480	-0.7007461	-2.5065942	Negative	0.87779092	0.02575274
MET_DOWN_RNA_Seq_Significant_Genes_JCI.2018_PMID.29480819	-0.7422796	-2.4529438	Negative	0.87779092	0.02829962
Pcorr_Carcinoid_JMD.2013_PMID.23701907	-0.7846137	-2.4245237	Negative	0.87779092	0.03019891
Duke_Module19_src_Mike_PMID.20335537	-0.9703166	-2.4011935	Negative	0.87779092	0.03119451
GSEA_GP8_FOXO_stemness.r.0.931_TTGTTT_V.FOXO4_01	-0.640468	-2.4066216	Negative	0.87779092	0.03135899
Melanoma_Scorr_MITF.low_Cell.2015_PMID.26091043	-0.722013	-2.3962186	Negative	0.87779092	0.03159962
TRIM29_Single_Gene	0.5296036	2.39095155	Positive	0.87779092	0.03213302
ERBB2_Single_Gene	0.29845803	2.35086044	Positive	0.87779092	0.03381334
TCGA_Tfh_cells_Immunity.2018_PMID._29628290	-0.7998017	-2.3593571	Negative	0.87779092	0.03434931
IMMUNE_Bindea_Cell_TFH_Immunity.2013_PMID.24138885	-0.7998017	-2.3593571	Negative	0.87779092	0.03434931
YALE_PIK3CA_Pathway_Ann.Oncol.2017_PMID.28177460	-0.8588229	-2.319156	Negative	0.87779092	0.03525676
MM_Green10_BMC.Med.Genomics.2011_PMID.21214954	-0.8558885	-2.3071986	Negative	0.87779092	0.03739869
MM_Green15_BMC.Med.Genomics.2011_PMID.21214954	-0.7276469	-2.1881297	Negative	0.87779092	0.04384327
IMMUNE_Bindea_Cell_Tcm_Immunity.2013_PMID.24138885	-0.7176624	-2.1597213	Negative	0.87779092	0.04960261
X8p22_Amplicon_BMC.Med.Genomics.2011_PMID.21214954	-0.9744909	-2.0804958	Negative	0.87779092	0.0566101
GSEA_BIOCARTA_RAS_PATHWAY	-0.8270338	-2.0407282	Negative	0.87779092	0.05898928
Melanoma_Scorr_Immune_Cell.2015_PMID.26091043	-0.8138542	-2.0324366	Negative	0.87779092	0.06136353
GSEA_GP3_Tumor_suppressing_miRNA_targets.r.0.940_TGCTTTG.MIR_330	-0.6410947	-2.0178551	Negative	0.87779092	0.06410505
Bcells_Centrocyte_J.Clin.Oncol.2015_PMID.25800755	-1.102247	-1.9850056	Negative	0.87779092	0.06489095
ACTIVATED_LUNG_MSC_SIGNATURE_Nat.Cell.Biol.2019_PMID.31263265	-0.728486	-1.9771729	Negative	0.87779092	0.06765313
New.Immune_Charoentong_Eosinophil_CellRep.2017_PMID.28052254	-1.0860089	-1.9636849	Negative	0.87779092	0.0679634
Duke_Module06_er_Mike_PMID.20335537	-0.3361803	-1.9732461	Negative	0.87779092	0.06953134
Taube_EMT_up_PNAS.2010_PMID.20713713	-0.7222386	-1.9499006	Negative	0.87779092	0.07078005
Shipitsin_CD44_A_Cancer.Cell.2007_PMID.17349583	-0.4053791	-1.9605683	Negative	0.87779092	0.07091039
Duke_Module18_ras_Mike_PMID.20335537	-0.8079991	-1.91936	Negative	0.87779092	0.07336577
Inflammatory_Breast_Cancer_79_IBC_CCR.2013_PMID.23396049	-0.7398223	-1.8992261	Negative	0.87779092	0.07571705
GSEA_MTOR_PATHWAY_BIOCARTA	-0.6996293	-1.910037	Negative	0.87779092	0.077704
Pcorr_Hypoxia_Low_Correlation_PLoS.Med.2006_PMID.16417408	0.81413032	1.88566163	Positive	0.87779092	0.07792464
FOS_JUN_Cluster_BMC.Med.Genomics.2011_PMID.21214954	-0.6636115	-1.8792238	Negative	0.87779092	0.07855437
TCGA.BRCA.1198_immune_FOS_JUN_IL6_JCI.2020_PMID.32573490	-0.6233004	-1.8858572	Negative	0.87779092	0.07914525
aMaSC_Shehata_BCR.2015_PMID.25575446	-0.5361792	-1.8953288	Negative	0.87779092	0.07950484
HGF_up_BCR.2013_PMID.24025166	-0.9656094	-1.8762135	Negative	0.87779092	0.08020253
Pcorr_Hypoxia_High_Correlation_PLoS.Med.2006_PMID.16417408	-0.7700162	-1.8481351	Negative	0.87779092	0.0831525
UNC_Scorr_Her2_Correlation_JCO.2009_PMID.19204204	0.6238312	1.83773582	Positive	0.87779092	0.0853998
New.Immune_Charoentong_Neutrophil_CellRep.2017_PMID.28052254	-0.6108854	-1.8281825	Negative	0.87779092	0.08622795
MM_Green24_BMC.Med.Genomics.2011_PMID.21214954	-0.7681772	-1.8424439	Negative	0.87779092	0.08711042
GRB7_Single_Gene	0.61010877	1.82442192	Positive	0.87779092	0.08899992
EMT_DOWN_Weingberg_PNAS.2010_PMID.20713713	0.68126268	1.81937555	Positive	0.87779092	0.08929823
GSEA_GP3_Tumor_suppressing_miRNA_targets.r.0.952_DACOSTA_UV_RESPON	-0.6674341	-1.7998635	Negative	0.87779092	0.0940515
TCGA.BRCA.1198_UNKNOWN6_JCI.2020_PMID.32573490	-0.4209298	-1.7836144	Negative	0.87779092	0.09457753
Lung.WNT.DiMeo_Cancer.Res.2009_PMID.19549913	-0.7491725	-1.7736739	Negative	0.87779092	0.09514792
Early_IRS_1_PLoS.One.2016_PMID.26991655	-0.809572	-1.7819961	Negative	0.87779092	0.0955858
MM_NeuPyMT_1pFDR_UP_Genome.Biology.2007_PMID.17493263	-0.6013885	-1.791116	Negative	0.87779092	0.09598637
New.Immune_Charoentong_CD56bright_natural_killer_cell_CellRep.2017_PMII	-0.7140508	-1.7653728	Negative	0.87779092	0.09657519
HS_Red2_BMC.Med.Genomics.2011_PMID.21214954	-0.5481669	-1.7792795	Negative	0.87779092	0.09776348
TCGA.BRCA.1198_NORMAL2_JCI.2020_PMID.32573490	-0.4339895	-1.7712839	Negative	0.87779092	0.09789129
MUnknown_6_BMC.Med.Genomics.2011_PMID.21214954	-0.8597727	-1.758899	Negative	0.87779092	0.09996556
Pcorr_Squamous_Cell_Carcinoma_JMD.2013_PMID.23701907	0.63712934	1.76221069	Positive	0.87779092	0.10084176
Inflammatory_Breast_Cancer_491_nIBC_CCR.2013_PMID.23396049	-0.5244705	-1.7464946	Negative	0.87779092	0.10337664
MM_Red2_BMC.Med.Genomics.2011_PMID.21214954	-0.6364865	-1.7439864	Negative	0.87779092	0.10395061
Spike2012_fMaSC_PMID.25575446	0.74287507	1.73435272	Positive	0.87779092	0.10502559
Extensive_Residual_Diesase_ER.Neg_54_JAMA.2011_PMID.21558518	-0.7270588	-1.7197647	Negative	0.87779092	0.10549016
MUnknown_19_BMC.Med.Genomics.2011_PMID.21214954	0.69995486	1.71478158	Positive	0.87779092	0.10625973
EXTENDED_B_CELL_signatures_Garber_Cell.Mol.Gastroenterol.Hepatol.2017_P	-0.7745029	-1.7157075	Negative	0.87779092	0.10748581
Early_Response_ER.Neg_27_JAMA.2011_PMID.21558518	0.44636615	1.69524141	Positive	0.87779092	0.10939901
New.Immune_Charoentong_Monocyte_CellRep.2017_PMID.28052254	-0.7942689	-1.6975091	Negative	0.87779092	0.10941561
Duke_Module20_stat3_Mike_PMID.20335537	-0.4854947	-1.6993612	Negative	0.87779092	0.10946637
Mature_Luminal_Down_Nat.Med.2009_PMID.19648928	-0.510286	-1.6987236	Negative	0.87779092	0.11035077
MRibosomal_BMC.Med.Genomics.2011_PMID.21214954	-0.7745475	-1.7026135	Negative	0.87779092	0.11051976
XBP1_Single_Gene	-0.4926546	-1.6976884	Negative	0.87779092	0.11155837
MCF7.E2.repressed.genes_JCO.2006_PMID.16505416	-0.3903863	-1.7044146	Negative	0.87779092	0.11180297
MM_Green9_BMC.Med.Genomics.2011_PMID.21214954	0.77505652	1.68403789	Positive	0.87779092	0.1140388
IMMUNE_Bindea_Cell_Macrophages_Immunity.2013_PMID.24138885	-0.794377	-1.6719514	Negative	0.87779092	0.11494789
Claudin_Low_29_Cancer.Res.2009_PMID.19435916	0.79612712	1.66478533	Positive	0.87779092	0.11572681
MUnknown_4_BMC.Med.Genomics.2011_PMID.21214954	0.71211215	1.65520993	Positive	0.87779092	0.11736123
Mouse_Human_ImmuneProfiles_SHAY_M_H_Induced_in_GN_PNAS.2013_PMII	-0.7616897	-1.6590553	Negative	0.87779092	0.11783383
New.Immune_Charoentong_Mast_cell_CellRep.2017_PMID.28052254	-0.8028891	-1.6567854	Negative	0.87779092	0.11784029
RB1_Single_Gene	-0.4995717	-1.6685512	Negative	0.87779092	0.11850012
Ribosomal_Cluster_BMC.Med.Genomics.2011_PMID.21214954	-0.8006159	-1.6586467	Negative	0.87779092	0.11886449
New.Immune_Charoentong_Central_memory_CD8_T_cell_CellRep.2017_PMID.	-0.5354556	-1.6551336	Negative	0.87779092	0.1196011
IMMUNE_Bindea_Cell_Normal_mucosa_Immunity.2013_PMID.24138885	-0.8246246	-1.643819	Negative	0.87779092	0.11971608
New.Immune_ImSig.TranslationPathway_CancerImmunolRes.2018_PMID.3026	-0.7784552	-1.6448973	Negative	0.87779092	0.12162297
IMMUNE_Bindea_Cell_T_helper_cells_Immunity.2013_PMID.24138885	-0.7157348	-1.6425792	Negative	0.87779092	0.12178137
GSEA_MYC_amplified_chr8q24	0.67712268	1.64101608	Positive	0.87779092	0.12215294
Stingl_Down_Basal_High_Nat.Cell.Biol.2014_PMID.25173976	0.38740283	1.64702866	Positive	0.87779092	0.122847
MUnknown_31_BMC.Med.Genomics.2011_PMID.21214954	-0.6557792	-1.6368488	Negative	0.87779092	0.12432451

Signatures_UP-DOWN_p<0.05	Renamed for figure purposes and simplicity	B_value	T.value	dir	P.adj	P.value
Unknown_15_BMC.Med.Genomics.2011_PMID.21214954	GO-Cell_signaling/Neuron_projection	0.7710384	3.6543941	Positive	0.99631	0.0061421
MM_Green13_BMC.Med.Genomics.2011_PMID.21214954	GO-Contractile_fiber_composed_of_actin/myosin	1.0027089	2.8253019	Positive	0.99631	0.0200631
MM_Red3_BMC.Med.Genomics.2011_PMID.21214954	GO-Receptor_binding/Cell_signalling/cell_adhesion	0.9810561	2.6369825	Positive	0.99631	0.0285578
Duke_Module16_pi3k_Mike_PMID.20335537	Duke_Module16_pi3k	-1.3474418	-5.365483	Negative	0.54466	0.0006515
Duke_Module18_ras_Mike_PMID.20335537	Duke_Module18_ras	-0.9733013	-4.0525551	Negative	0.96728	0.0023141
IMMUNE_Bindea_Cell_CD8_T_cells_Immunity.2013_PMID.24138885	IMMUNE_Bindea_Cell_CD8_T_cells	-1.1787055	-3.7554605	Negative	0.99631	0.0053572
MM_p53null.Luminal_Genome.Biol.2013_PMID.24220145	MM_p53null.Luminal	-0.9505822	-3.5389593	Negative	0.99631	0.0064607
Inflammatory_Breast_Cancer_79_IBC_CCR.2013_PMID.23396049	Inflammatory_Breast_Cancer	-0.79145	-3.1883145	Negative	0.99631	0.0096818
KRAS_amplicon_Genome.Biology.2007_PMID.17493263	KRAS_amplicon	-1.1114397	-3.157797	Negative	0.99631	0.0117803
HS_Green2_BMC.Med.Genomics.2011_PMID.21214954	GO-Protein localization/Mitotic_cell_cycle	-0.7906082	-2.956679	Negative	0.99631	0.0173249
HS_Red17_BMC.Med.Genomics.2011_PMID.21214954	GO-Nucleotide_binding/Apoptosis/Response_stress	-0.9702376	-2.9619011	Negative	0.99631	0.017515
Duke_Module07_glucosedepletion_Mike_PMID.20335537	Duke_Module07	-0.8872726	-2.9469965	Negative	0.99631	0.0181057
Taube_EMT_down_PNAS.2010_PMID.20713713	Taube_EMT_down	-0.6532608	-2.7898558	Negative	0.99631	0.0212823
MNADH_CYTochrome_BMC.Med.Genomics.2011_PMID.21214954	MNADH_CYTochrome	-0.9499026	-2.8173595	Negative	0.99631	0.0217366
MET_DOWN_RNA_Seq_Significant_Genes_JCI.2018_PMID.29480819	MET_DOWN_RNA_Seq_Significant_Genes	-0.6795672	-2.7700436	Negative	0.99631	0.0237745
Excellent_Pathologic_Response_ER.Neg_55_JAMA.2011_PMID.21558518	Excellent_Pathologic_Response_ER.Neg	-1.378017	-2.7403081	Negative	0.99631	0.0238027
HS_Green11_BMC.Med.Genomics.2011_PMID.21214954	GO-Peptide_metabolic_process/translation	-0.908802	-2.7596795	Negative	0.99631	0.0240759
MUnknown_6_BMC.Med.Genomics.2011_PMID.21214954	GO-RNA binding/Bibosome biogenesis	-1.1719368	-2.7360769	Negative	0.99631	0.0242044
MUnknown_12_BMC.Med.Genomics.2011_PMID.21214954	GO-RNA binding/GTPase interaction	-1.0965031	-2.6045226	Negative	0.99631	0.0301313
HouseKeeping_Genome.Biol.2004_PMID.15287981	HouseKeeping_genes	-0.9501851	-2.5184608	Negative	0.99631	0.0333387
MKRAS_amplicon_BMC.Med.Genomics.2011_PMID.21214954	MKRAS_amplicon	-0.95425	-2.4455145	Negative	0.99631	0.0345217
Duke_Module04_e2f1_Mike_PMID.20335537	Duke_Module04_e2f1_Mike_PMID.20335537	-1.1385606	-2.5146431	Negative	0.99631	0.0348431
New.Immune_Charoentong_CD56bright_natural_killer_cell_CellRep.2017_PMID.2805225	New.Immune_Charoentong_CD56bright_NK_cells	-1.0838125	-2.4232777	Negative	0.99631	0.0358601
MM_Green4_BMC.Med.Genomics.2011_PMID.21214954	GO-Apoptotic_process/Metabolic process	-0.9011217	-2.4441346	Negative	0.99631	0.0394585
HS_Red22_BMC.Med.Genomics.2011_PMID.21214954	GO-Protein localization/Vesicle transport	-0.8348042	-2.4246622	Negative	0.99631	0.0406106
KRAS_Single_Gene	KRAS_Single_Gene	-1.037734	-2.382985	Negative	0.99631	0.0417934
HER1_Cluster3_BMC.Genomics.2007_PMID.17663798	HER1_Cluster3	-0.9242736	-2.3855769	Negative	0.99631	0.0419924
GSEA_RESPONSE_TO_ANDROGEN_UP_NELSON	GSEA_RESPONSE_TO_ANDROGEN_UP_NELSON	-0.7433566	-2.358427	Negative	0.99631	0.0447941
GSEA_GP3_Tumor_suppressing_miRNA_targets.r.0.940_TGCTTTG.MIR_330	GSEA_GP3_Tumor_suppressing_miRNA_targets.	-0.9347022	-2.2966622	Negative	0.99631	0.0499537

IntClust_Deletion.50.Better.than_Genome.Biol.2014_PMID.25164602	0.55232615	1.62618848	Positive	0.87779092	0.12618423
ACTIVATED_LUNG_NEUTROPHIL_SIGNATURE_Nat.Cell.Biol.2019_PMID.312632	-0.8610957	-1.6192776	Negative	0.87779092	0.12738044
TCGA_TGFB_score_21050467_Immunity.2018_PMID._29628290	-0.6624895	-1.604143	Negative	0.87779092	0.12897429
HS_Red17_BMC.Med.Genomics.2011_PMID.21214954	-0.7102021	-1.6029729	Negative	0.87779092	0.13096182
HS_Green11_BMC.Med.Genomics.2011_PMID.21214954	-0.7135556	-1.5975724	Negative	0.87779092	0.13244548
Excellent_Pathologic_Response_ER.Neg_55_JAMA.2011_PMID.21558518	-0.7761062	-1.5961389	Negative	0.87779092	0.13244765
Her2_Basal_JCI.2020_PMID.32573490	0.24370247	1.598213	Positive	0.87779092	0.13309372
HS_Green1_BMC.Med.Genomics.2011_PMID.21214954	-0.5532754	-1.5937074	Negative	0.87779092	0.13334882
TCGA.BRCA.1198_FGFR4_EGF_JCI.2020_PMID.32573490	0.64962154	1.57675746	Positive	0.87779092	0.13441545
GSEA_GP6_Squamous_differentiation_development.r.0.902_RICKMAN_TUMOR	-0.3966154	-1.5738322	Negative	0.87779092	0.13589302
GENE_PANEL_TRUSEQ_40_ILLUMINA	-0.6578284	-1.5728968	Negative	0.87779092	0.13855972
LUMINAL_Cluster_BMC.Med.Genomics.2011_PMID.21214954	-0.468049	-1.5682558	Negative	0.87779092	0.13925604
Murat_G07_J.Clin.Oncol.2008_PMID.18565887	-0.7147262	-1.5550883	Negative	0.87779092	0.13948115
Duke_Module05_egfr_Mike_PMID.20335537	-0.461553	-1.5668343	Negative	0.87779092	0.13954539
Unknown_10_BMC.Med.Genomics.2011_PMID.21214954	-0.520137	-1.5513754	Negative	0.87779092	0.14147484
IMMUNE_Bindea_Cell_NK_CD56dim_cells_Immunity.2013_PMID.24138885	0.46223014	1.5553013	Positive	0.87779092	0.14299619
X13q14_Amplicon_BMC.Med.Genomics.2011_PMID.21214954	-0.7433892	-1.552299	Negative	0.87779092	0.14332997
Duke_Module16_pi3k_Mike_PMID.20335537	-0.6978073	-1.547306	Negative	0.87779092	0.14414726
Mouse_Human_ImmuneProfiles_SHAY_M_H_Induced_in_MO_PNAS.2013_PMI	-0.7373687	-1.5418504	Negative	0.87779092	0.1442371
MNADH_CYTochrome_BMC.Med.Genomics.2011_PMID.21214954	-0.5830271	-1.5440498	Negative	0.87779092	0.14447646
IMMUNE_Bindea_Cell_Blood_vessels_Immunity.2013_PMID.24138885	-0.8043257	-1.5328738	Negative	0.87779092	0.14532365
PARP_sensitivity_MDACC_NPJ.Syst.Biol.Appl._2017_PMID.28649435	-0.7599418	-1.535923	Negative	0.87779092	0.14715949
New.Immune_Charoentong_Immature_dendritic_cell_CellRep.2017_PMID.280!	-0.5946483	-1.5313718	Negative	0.87779092	0.14722123
BASAL_Cluster_BMC.Med.Genomics.2011_PMID.21214954	0.37423263	1.53582536	Positive	0.87779092	0.14746023
MK14_K17_BMC.Med.Genomics.2011_PMID.21214954	0.51924349	1.52894053	Positive	0.87779092	0.14826167
MM_Red20_BMC.Med.Genomics.2011_PMID.21214954	0.47048428	1.52218009	Positive	0.87779092	0.15076608
IMMUNE_Bindea_Cell_SW480_cancer_cells_Immunity.2013_PMID.24138885	0.77090245	1.50583013	Positive	0.87779092	0.15400886
MUnknown_18_BMC.Med.Genomics.2011_PMID.21214954	0.47321644	1.50144578	Positive	0.87779092	0.15421753
MM_Red23_BMC.Med.Genomics.2011_PMID.21214954	-0.5819622	-1.5033239	Negative	0.87779092	0.15574473
Murat_G18_J.Clin.Oncol.2008_PMID.18565887	0.71576698	1.49258904	Positive	0.87779092	0.15679181
MUnknown_13_BMC.Med.Genomics.2011_PMID.21214954	-0.6937329	-1.489712	Negative	0.87779092	0.15796332
MM_Red3_BMC.Med.Genomics.2011_PMID.21214954	0.75350368	1.48305593	Positive	0.87779092	0.15902598
aStr_Shehata_BCR.2015_PMID.25575446	-0.6118738	-1.475788	Negative	0.87779092	0.15940903
Euclidean_Distance_2_CLOW_Euclidean.Distance_BCR.2010_PMID.20813035	0.64495615	1.47473446	Positive	0.87779092	0.15968893
ESR1_Single_Gene	-0.2785491	-1.4769532	Negative	0.87779092	0.1609859
HS_Red24_BMC.Med.Genomics.2011_PMID.21214954	-0.5914846	-1.4654179	Negative	0.87779092	0.16218194
X11q13_Amplicon_BMC.Med.Genomics.2011_PMID.21214954	-0.4338597	-1.4756203	Negative	0.87779092	0.16319069
Pcorr_secretory_CCR.2010_PMID.20643781	-0.6111563	-1.4608648	Negative	0.87779092	0.16488494
GSEA_GP9_Cell_cell_adhesion.r.960_PerouLab_MClaudin_Cluster_BMC_Med_(	0.70511346	1.45870025	Positive	0.87779092	0.1656962
HS_Red8_BMC.Med.Genomics.2011_PMID.21214954	0.33273958	1.46479847	Positive	0.87779092	0.16597219
TCGA.BRCA.1198_immune_CD34_TIE1_JCI.2020_PMID.32573490	-0.77192	-1.4502233	Negative	0.87779092	0.1663168
Fibroblast_Cluster_BMC.Med.Genomics.2011_PMID.21214954	-0.5959331	-1.4395752	Negative	0.87779092	0.16926587
MUnknown_12_BMC.Med.Genomics.2011_PMID.21214954	-0.6784905	-1.4450068	Negative	0.87779092	0.16983761
GSEA_GP16_Protein_kinase_signailng_MAPKs.r.0.893_INTRACELLULAR_SIGNAL	-0.6410888	-1.4306672	Negative	0.87779092	0.17176591
HS_Red21_BMC.Med.Genomics.2011_PMID.21214954	-0.5822546	-1.424575	Negative	0.87779092	0.17349308
New.Immune_ImmLandscape_IFN_3.PMID.24516633	0.504178	1.43076126	Positive	0.87779092	0.17529182
TCGA_Module3_IFN_score_Immunity.2018_PMID._29628290	0.504178	1.43076126	Positive	0.87779092	0.17529182
TCGA.BRCA.1198_BASAL_JCI.2020_PMID.32573490	0.32046596	1.42833631	Positive	0.87779092	0.17608644
MFGFR2_BMC.Med.Genomics.2011_PMID.21214954	-0.6193276	-1.4215418	Negative	0.87779092	0.17633191
GSEA_GP15_EGF_signailng.r.0.936_NAGASHIMA_EGF_SIGNALING_UP	-0.5247978	-1.4137444	Negative	0.87779092	0.17689757
MDSC_tumor_MO_Schlecker_J.Immunol.2012_PMID.23152559	-0.5361209	-1.4185699	Negative	0.87779092	0.1773561
X16q23_Amplicon_BMC.Med.Genomics.2011_PMID.21214954	-0.5155432	-1.4190946	Negative	0.87779092	0.1775477
GSEA_GP4_MES_ECM.r.0.954_PerouLab_HS_Red7_BMC_Med_Genomics_2011	-0.6255692	-1.4056955	Negative	0.87779092	0.17925872
CIBERSORT_Eosinophils_Nat.Methods.2015_PMID.25822800	-0.7214783	-1.3991696	Negative	0.87779092	0.18167984
MRE_Score_Breast.Cancer.Res.Treat_2015_PMID.26109344	0.55370247	1.39716201	Positive	0.87779092	0.18283659
MM_Green4_BMC.Med.Genomics.2011_PMID.21214954	-0.6094316	-1.3977109	Negative	0.87779092	0.18410216
Shipitsin_CD44_B_Cancer.Cell.2007_PMID.17349583	-0.6128606	-1.3804861	Negative	0.87779092	0.18727539
MM_Red12_BMC.Med.Genomics.2011_PMID.21214954	0.73138341	1.37724207	Positive	0.87779092	0.18780176
Tcell_EXH_Memory_CD8_T_cell_a_vs_Naive_CD8_T_cell_Metagene_2_Science.	-0.6947477	-1.3735854	Negative	0.87779092	0.18851179
HS_Red22_BMC.Med.Genomics.2011_PMID.21214954	-0.6040813	-1.3815231	Negative	0.87779092	0.18862522
New.Immune_Charoentong_Central_memory_CD4_T_cell_CellRep.2017_PMID.	-0.6575008	-1.3729037	Negative	0.87779092	0.1887195
GSEA_BIOCARTA_PTEN_PATHWAY	-0.6979373	-1.3731545	Negative	0.87779092	0.1901022
MDACC.FNA.2_J.Clin.Oncol.2010_PMID.20805453	-0.6426592	-1.3648988	Negative	0.87779092	0.19117238
Neutrophils_MCP_PMID.31942075_PMID.31942077	-0.5642073	-1.3658191	Negative	0.87779092	0.19137042
Stingl_Down_CLOW_High_Nat.Cell.Biol.2014_PMID.25173976	-0.6970886	-1.3593394	Negative	0.87779092	0.19335547
MM_Green5_BMC.Med.Genomics.2011_PMID.21214954	-0.6574931	-1.3560692	Negative	0.87779092	0.19390768
aMaSC_Prat_BCR.2015_PMID.25575446	-0.4898282	-1.3543279	Negative	0.87779092	0.19522387
FGFR4_Single_Gene	0.53338384	1.35336679	Positive	0.87779092	0.19540078
UNC_Differentiation.Score_Model_BCR.2010_PMID.20813035	0.3045209	1.35357539	Positive	0.87779092	0.1968388
Stromal_Down_Nat.Med.2009_PMID.19648928	0.54982952	1.34836891	Positive	0.87779092	0.19742087
Early_Relapse_ER.Pos_33_JAMA.2011_PMID.21558518	0.53583602	1.34865714	Positive	0.87779092	0.19772852
PARP_Sensitivity_Signature_NEGATIVE_Sci.Adv.2017_PMID.28439535	-0.5083237	-1.3489819	Negative	0.87779092	0.19946287
HS_Green9_BMC.Med.Genomics.2011_PMID.21214954	-0.572845	-1.337132	Negative	0.87779092	0.20029271
X15q25_Amplicon_BMC.Med.Genomics.2011_PMID.21214954	-0.5217471	-1.3449917	Negative	0.87779092	0.20087008
MProtocadherin_BMC.Med.Genomics.2011_PMID.21214954	-0.6572146	-1.3330108	Negative	0.87779092	0.20119942
MM_Erbb2.like_Genome.Biol.2013_PMID.24220145	0.40595148	1.33584427	Positive	0.87779092	0.20293392
Lim2010_MatureLum_Adam_PMID.25575446	0.57592708	1.32116844	Positive	0.87779092	0.20502865
X5Q_Breast.Cancer.Res.Treat.2012_PMID.22048815	-0.458965	-1.3280614	Negative	0.87779092	0.20518697
GSEA_GP3_Tumor_suppressing_miRNA_targets.r.0.940_GTTTGTT.MIR_495	-0.5433213	-1.3251445	Negative	0.87779092	0.20710077
MECM_BMC.Med.Genomics.2011_PMID.21214954	-0.6197477	-1.3142791	Negative	0.87779092	0.2072829
aStr_Prat_BCR.2015_PMID.25575446	-0.6014169	-1.3051085	Negative	0.87779092	0.2103141
LumB_Basal_JCI.2020_PMID.32573490	0.20593928	1.31357498	Positive	0.87779092	0.2107523
Scorr_EMAT3_Correlation_BCR.2020_PMID.32641077	0.5956926	1.30083575	Positive	0.87779092	0.21225766
Lim2009_MatureLum_Adam_PMID.25575446	0.21821538	1.29714558	Positive	0.87779092	0.21297454
TCGA.BRCA.1198_UNKNOWN2_JCI.2020_PMID.32573490	-0.4903354	-1.294336	Negative	0.87779092	0.21391956
CIBERSORT_Mast_cells_activated_Nat.Methods.2015_PMID.25822800	-0.585388	-1.294476	Negative	0.87779092	0.21443906
GSEA_GP21_Anti_apoptosis_DNA_stability.r.0.925_MORF_MT4	-0.5831205	-1.2933469	Negative	0.87779092	0.21632211
Pcorr_classical_CCR.2010_PMID.20643781	0.61233785	1.28160951	Positive	0.87779092	0.21824175
Scorr_P53_Mut_Correlation_BMC.Cancer.2006_PMID.17150101	0.53476769	1.27931443	Positive	0.87779092	0.21902848
TCGA.BRCA.1198_UNKNOWN5_JCI.2020_PMID.32573490	0.4646166	1.28177525	Positive	0.87779092	0.22026366
Mouse_Human_ImmuneProfiles_SHAY_M_H_Induced_in_B_PNAS.2013_PMID.:	-0.6893443	-1.2753866	Negative	0.87779092	0.22038006

PDGFRA_Single_Gene	-0.5745308	-1.2723019	Negative	0.87779092	0.22144614
New.Immune_ImSig.Monocytes_CancerImmunolRes.2018_PMID.30266715	-0.554271	-1.2760632	Negative	0.87779092	0.22236065
C_MYB_Signature_PLoS.One.2010_PMID.20949095	-0.4137199	-1.2754219	Negative	0.87779092	0.22363754
MDSC_tumor_Schlecker_J.Immunol.2012_PMID.23152559	-0.505507	-1.2654406	Negative	0.87779092	0.2259023
JANES_Oscillation_JUND_KRT5_Nat.Cell.Biol.2014_PMID.24658685	-0.5941134	-1.2631019	Negative	0.87779092	0.22593108
New.Immune_TCGA.BRCA.1198_immune_HLA_D_Cell.2015_PMID.26451490	-0.509451	-1.2637596	Negative	0.87779092	0.22668347
TCGA.BRCA.1198_immune_HLA_D_JCI.2020_PMID.32573490	-0.509451	-1.2637596	Negative	0.87779092	0.22668347
FGFR4_Induced_JCI.2020_PMID.32573490	-0.4523633	-1.252012	Negative	0.87779092	0.22939024
Secretoglobin_BMC.Med.Genomics.2011_PMID.21214954	0.28040731	1.25321073	Positive	0.87779092	0.23128476
aStr_HsEnriched_Refined2_BCR.2015_PMID.25575446	-0.5942831	-1.2398341	Negative	0.87779092	0.23291355
New.Immune_Charoentong_Natural_killer_cell_CellRep.2017_PMID.28052254	-0.6978493	-1.2410558	Negative	0.87779092	0.23400383
New.Immune_ImSig.Neutrophils_CancerImmunolRes.2018_PMID.30266715	-0.4884461	-1.2302272	Negative	0.87779092	0.23665707
Claudin_Low_Genome.Biol.2007_PMID.17493263	0.54916019	1.23023509	Positive	0.87779092	0.23741242
Early_IRS_2_PLoS.One.2016_PMID.26991655	-0.4478395	-1.2260609	Negative	0.87779092	0.23791552
Fibroblasts_MCP_PMID.31942075_PMID.31942077	-0.5057962	-1.225753	Negative	0.87779092	0.2380283
CIBERSORT_Neutrophils_Nat.Methods.2015_PMID.25822800	-0.4935434	-1.2224565	Negative	0.87779092	0.23994585
IMMUNE_Bindea_Cell_Tem_Immunity.2013_PMID.24138885	-0.4677208	-1.2164193	Negative	0.87779092	0.24146622
Duke_Module02_akt_Mike_PMID.20335537	-0.4859691	-1.2192324	Negative	0.87779092	0.2427723
GSEA_HDAC_TARGETS_DN_HELLER	-0.5813462	-1.2123144	Negative	0.87779092	0.24299027
MM_Green20_BMC.Med.Genomics.2011_PMID.21214954	-0.4420371	-1.2104103	Negative	0.87779092	0.24369976
TCGA.BRCA.1198_Chromogranin_JCI.2020_PMID.32573490	0.71613772	1.20879474	Positive	0.87779092	0.24478631
MUnknown_15_BMC.Med.Genomics.2011_PMID.21214954	-0.4898349	-1.2125654	Negative	0.87779092	0.24554055
MM_Red9_BMC.Med.Genomics.2011_PMID.21214954	-0.5332556	-1.2080297	Negative	0.87779092	0.24567883
EMT_UP_Weinberg_PNAS.2010_PMID.20713713	-0.5175108	-1.2048039	Negative	0.87779092	0.24579791
Stingl_Up_Basal_High_Nat.Cell.Biol.2014_PMID.25173976	0.25082599	1.21234842	Positive	0.87779092	0.24661014
TLS_CXCL13_SingleGene_.31942077	0.50414604	1.20743728	Positive	0.87779092	0.24671749
MM_p53null.Luminal_Genome.Biol.2013_PMID.24220145	-0.4270546	-1.2014993	Negative	0.87779092	0.24704115
TCGA.BRCA.1198_EN1_FDZ9_JCI.2020_PMID.32573490	0.38036738	1.20761944	Positive	0.87779092	0.24704544
MM_p53null_1pFDR_UP_Genome.Biology.2007_PMID.17493263	-0.44676	-1.2068238	Negative	0.87779092	0.24748993
MVEGFC_BMC.Med.Genomics.2011_PMID.21214954	-0.5302413	-1.1995783	Negative	0.87779092	0.24833533
CD34_CD36_Cluster_BMC.Med.Genomics.2011_PMID.21214954	-0.5670877	-1.19334	Negative	0.87779092	0.25013141
Immunosuppression_PMID.31942077	-0.5893346	-1.1962589	Negative	0.87779092	0.25057384
PTEN_Single_Gene	-0.5191502	-1.18627	Negative	0.87779092	0.25283299
GHI_RS_Model_NJEM.2004_PMID.15591335	0.57384769	1.18425246	Positive	0.87779092	0.25360798
ACTIVATED_BLOOD_NEUTROPHIL_SIGNATURE_Nat.Cell.Biol.2019_PMID.31263	-0.5533235	-1.1849926	Negative	0.87779092	0.25404747
MM_Red13_BMC.Med.Genomics.2011_PMID.21214954	0.62692462	1.18269706	Positive	0.87779092	0.2542067
HS_Red11_BMC.Med.Genomics.2011_PMID.21214954	-0.4366606	-1.1892459	Negative	0.87779092	0.25477308
X8p_Amplicon_BMC.Med.Genomics.2011_PMID.21214954	0.66372077	1.1810699	Positive	0.87779092	0.25483419
Cytotoxic_lymphocytes_MCP_PMID.31942075_PMID.31942077	-0.4719437	-1.1813254	Negative	0.87779092	0.25605948
MM_Potluck_1pFDR_UP_Genome.Biology.2007_PMID.17493263	-0.550219	-1.1743675	Negative	0.87779092	0.25821479
Inflammatory_Breast_Cancer_491_IBC_CCR.2013_PMID.23396049	-0.6254155	-1.1732862	Negative	0.87779092	0.25824468
TCGA.BRCA.1198_S100A7_8_9_JCI.2020_PMID.32573490	0.34700091	1.1769731	Positive	0.87779092	0.25870221
Endothelial_cells_MCP_PMID.31942075_PMID.31942077	-0.6260015	-1.1702601	Negative	0.87779092	0.25903283
Fibromatosis_Lab.Invest.2008_PMID.18414401	-0.5461038	-1.168321	Negative	0.87779092	0.25979153
HS_Green7_BMC.Med.Genomics.2011_PMID.21214954	-0.4895468	-1.1733562	Negative	0.87779092	0.26058746
Duke_Module09_hypoxia_Mike_PMID.20335537	-0.4020784	-1.1718472	Negative	0.87779092	0.26101162
GSEA_GP10_Fatty_acid_oxidation.r.0.930_CARBOXYLIC_ACID_METABOLIC_PRC	-0.4264192	-1.1697072	Negative	0.87779092	0.26133075
IntClust_Deletion.50_Genome.Biol.2014_PMID.25164602	0.54805633	1.16677724	Positive	0.87779092	0.26150557
Mouse_Human_ImmuneProfiles_SHAY_M_H_Induced_in_DC_PNAS.2013_PMIC	-0.5848015	-1.1609729	Negative	0.87779092	0.26268184
Verhaak_Stromal_Nat.Commun.2013_PMID.24113773	-0.5768829	-1.1619845	Negative	0.87779092	0.26312962
New.Immune_Miller_M_D_Metagene_9gene_Genome_Biol_2013_PMID.23618	-0.4774411	-1.1652644	Negative	0.87779092	0.26321004
Miller_M_D_Metagene_Genome_Biol_2013_PMID.23618380	-0.4774411	-1.1652644	Negative	0.87779092	0.26321004
New.Immune_Charoentong_Macrophage_CellRep.2017_PMID.28052254	-0.5286007	-1.1617821	Negative	0.87779092	0.26387521
Knudsen_Neo_ER_negative_Clin.Cancer.Res.2014_PMID.25047707	-0.5345021	-1.1575699	Negative	0.87779092	0.26526428
HRneg.Tneg_14.Genes_BCR.2010_PMID.20946665	0.60377543	1.15882396	Positive	0.87779092	0.26596011
Unknown_12_BMC.Med.Genomics.2011_PMID.21214954	-0.3659529	-1.1505755	Negative	0.87779092	0.26962749
Tcell_EXH_Memory_CD8_T_cell_a_vs_Naive_CD8_T_cell_Metagene_3_Science.	-0.4626114	-1.1445799	Negative	0.87779092	0.27029217
PGR_Single_Gene	-0.3855846	-1.1414276	Negative	0.87779092	0.27048817
MM_Claudinlow_Genome.Biol.2013_PMID.24220145	-0.5455481	-1.1415927	Negative	0.87779092	0.27122821
Interferon_Breast.Cancer.Research.2008_PMID.19272155	0.36185486	1.14562364	Positive	0.87779092	0.27205898
Tcell_EXH_Exhausted_CD8_T_cell_vs_Naive_CD8_T_cell_Metagene_3_Science.	-0.5746824	-1.1290147	Negative	0.87779092	0.27623036
NFIB_Single_Gene	-0.3252807	-1.1319109	Negative	0.87779092	0.27754708
HS_Red5_BMC.Med.Genomics.2011_PMID.21214954	0.48915615	1.12084861	Positive	0.87779092	0.2788943
Influenza_11genes_Metasignature_KHATRI_Immunity.2015_PMID.26682989	0.43645218	1.12333181	Positive	0.87779092	0.28082272
MUnknown_10_BMC.Med.Genomics.2011_PMID.21214954	-0.5327475	-1.1207051	Negative	0.87779092	0.28138895
GSEA_GP2_Immune_Tcell_Bcell.r.0.964_KEGG_HEMATOPOIETIC_CELL_LINEAGI	-0.5985331	-1.1116982	Negative	0.87779092	0.28335046
CIBERSORT_Monocytes_Nat.Methods.2015_PMID.25822800	-0.4936538	-1.1073381	Negative	0.87779092	0.2858942
GSEA_GP8_FOXO_stemness.r.0.875_MORF_PTPRB	-0.4724223	-1.1060625	Negative	0.87779092	0.28709741
IMMUNE_Bindea_Cell_Neutrophils_Immunity.2013_PMID.24138885	-0.4671408	-1.1000008	Negative	0.87779092	0.28760724
MM_WapINT3_1pFDR_UP_Genome.Biology.2007_PMID.17493263	-0.4736951	-1.106132	Negative	0.87779092	0.28770849
LOBULAR_TCGA_SUBTYPE_Reactive_like_Cell.2015_PMID.26451490	0.3538839	1.10165951	Positive	0.87779092	0.28905919
X17PP13_Amplicon_BMC.Med.Genomics.2011_PMID.21214954	-0.4568503	-1.1006511	Negative	0.87779092	0.28956548
CD103_Negative_Cancer.Cell.2014_PMID.25446897	-0.4861947	-1.0952102	Negative	0.87779092	0.29083652
MM_Green18_BMC.Med.Genomics.2011_PMID.21214954	-0.4711754	-1.0903705	Negative	0.87779092	0.29169934
HS_Green15_BMC.Med.Genomics.2011_PMID.21214954	-0.4818757	-1.0909431	Negative	0.87779092	0.29260934
MM_Green8_BMC.Med.Genomics.2011_PMID.21214954	-0.4094085	-1.0892207	Negative	0.87779092	0.29274503
Scorr_P53_Wt_Correlation_BMC.Cancer.2006_PMID.17150101	-0.4606554	-1.087685	Negative	0.87779092	0.2928481
GSEA_GP21_Anti_apoptosis_DNA_stability.r.0.898_MORF_STK17A	-0.5255573	-1.0839302	Negative	0.87779092	0.29475899
MS4A1_Single_Gene	-0.5096046	-1.081959	Negative	0.87779092	0.29530855
Wahl_fMasC_Signature_Cell.Stem.Cell.2012_PMID.22305568	0.51724907	1.08629054	Positive	0.87779092	0.29580008
Unknown_3_BMC.Med.Genomics.2011_PMID.21214954	-0.4816571	-1.0832525	Negative	0.87779092	0.29668043
GSEA_BIOCARTA_ALK_PATHWAY	-0.4815342	-1.0792469	Negative	0.87779092	0.29703569
HS_Red14_BMC.Med.Genomics.2011_PMID.21214954	0.52371077	1.07752585	Positive	0.87779092	0.29722384
CD68_cluster_Iglesia_CCR.2014_PMID.24916698	-0.4471579	-1.080277	Negative	0.87779092	0.29795647
TCGA.BRCA.1198_COLLAGEN11A_JCI.2020_PMID.32573490	-0.4231869	-1.0772357	Negative	0.87779092	0.29852923
HS_Red16_BMC.Med.Genomics.2011_PMID.21214954	0.37088481	1.07797041	Positive	0.87779092	0.29999726
MM_Green17_BMC.Med.Genomics.2011_PMID.21214954	-0.4893682	-1.0699932	Negative	0.87779092	0.30286068
Mouse_Human_ImmuneProfiles_SHAY_M_H_Induced_in_NK_PNAS.2013_PMIC	-0.5411169	-1.062401	Negative	0.87779092	0.30382682
HS_Green20_BMC.Med.Genomics.2011_PMID.21214954	-0.4172169	-1.0608613	Negative	0.87779092	0.30665854
Scorr_EMAT1_Correlation_BCR.2020_PMID.32641077	-0.4901209	-1.0574646	Negative	0.87779092	0.307237
GSEA_BIOCARTA_RB_PATHWAY	-0.4656446	-1.0503019	Negative	0.87779092	0.30918527

MHC.II_Breast.Cancer.Research.2008_PMID.19272155	-0.4298851	-1.0496307	Negative	0.87779092	0.31102187
Unknown_2_BMC.Med.Genomics.2011_PMID.21214954	-0.4648822	-1.046253	Negative	0.87779092	0.31265614
Scorr_EMAT4_Correlation_BCR.2020_PMID.32641077	0.30596442	1.04621616	Positive	0.87779092	0.31289799
NRAS_Single_Gene	0.35174966	1.04586065	Positive	0.87779092	0.31410939
New.Immune_Charoentong_T_follicular_helper_cell_CellRep.2017_PMID.28052	-0.5001935	-1.0410758	Negative	0.87779092	0.31461784
aStr_HsEnriched_BCR.2015_PMID.25575446	-0.5062601	-1.037911	Negative	0.87779092	0.31474354
MSquamous_BMC.Med.Genomics.2011_PMID.21214954	0.26984822	1.0433691	Positive	0.87779092	0.31501704
MM_Red6_BMC.Med.Genomics.2011_PMID.21214954	-0.4354192	-1.0335016	Negative	0.87779092	0.31673878
Lymphovascular_Invasion_J.Pathol.2017_PMID.27861902	0.34602769	1.03146863	Positive	0.87779092	0.31766174
GSEA_RETINOL_METABOLISM_KEGG	-0.4981208	-1.0303589	Negative	0.87779092	0.31816636
New.Immune_Charoentong_Effector_memeory_CD4_T_cell_CellRep.2017_PMI	-0.5537974	-1.0278965	Negative	0.87779092	0.31982673
FGFR4_Repressed_JCI.2020_PMID.32573490	-0.32541	-1.030733	Negative	0.87779092	0.32084252
Lim.et.al.2010.Conserved.Mature_BCR.2010_PMID.20346151	0.19785663	1.01774051	Positive	0.87779092	0.32489856
MM_Red15_BMC.Med.Genomics.2011_PMID.21214954	-0.4705649	-1.0165646	Negative	0.87779092	0.32522861
Keller2012_CD10_Adam_PMID.25575446	-0.3615499	-1.0126928	Negative	0.87779092	0.32827413
MM_Green1_BMC.Med.Genomics.2011_PMID.21214954	-0.5076658	-1.0090834	Negative	0.87779092	0.32885108
RSS_Score_Clin.Cancer.Res.2018_PMID.29921729	-0.3850416	-1.0104896	Negative	0.87779092	0.32963091
Pcorr_squamoid_PLOS.2012_PMID.22590557	-0.2937075	-1.0089878	Negative	0.87779092	0.3303015
Unknown_15_BMC.Med.Genomics.2011_PMID.21214954	0.43774653	1.0016755	Positive	0.87779092	0.33224701
MHC_Forero_11_Cancer.Immunol.Res.2016_PMID.26980599	-0.4345937	-1.0037879	Negative	0.87779092	0.33248167
Pcorr_Small_Cell_Carcinoma_JMD.2013_PMID.23701907	-0.4657901	-1.0018457	Negative	0.87779092	0.33414115
Stromal_Signature_Nat.Med.2008_PMID.18438415	-0.5311103	-0.9964809	Negative	0.87779092	0.33444538
MITO2_BMC.Med.Genomics.2011_PMID.21214954	-0.4379077	-0.9936622	Negative	0.87779092	0.33742279
MM_Red1_BMC.Med.Genomics.2011_PMID.21214954	-0.5134688	-0.9902181	Negative	0.87779092	0.33770772
aMaSC_HsEnriched_Refined1_BCR.2015_PMID.25575446	-0.5762344	-0.9866992	Negative	0.87779092	0.33879256
CIBERSORT_Macrophages_M2_Nat.Methods.2015_PMID.25822800	-0.4637565	-0.9876278	Negative	0.87779092	0.3394368
HS_Green4_BMC.Med.Genomics.2011_PMID.21214954	-0.4479708	-0.9847722	Negative	0.87779092	0.3400514
GATA3_Single_Gene	0.35975789	0.98526355	Positive	0.87779092	0.34135462
GSEA_GP22_16Q22_24_amplicon.r.0.927_PerouLab_16q24x_BMC_Med_Genoı	-0.4498742	-0.976778	Negative	0.87779092	0.34483131
GSEA_GP16_Protein_kinase_signailng_MAPKs.r.0.885_REGULATION_OF_KINAS	-0.4680972	-0.977222	Negative	0.87779092	0.34484864
MUnknown_24_BMC.Med.Genomics.2011_PMID.21214954	-0.3373074	-0.9780748	Negative	0.87779092	0.34497198
MM_Green21_BMC.Med.Genomics.2011_PMID.21214954	0.41271326	0.97679477	Positive	0.87779092	0.34563167
MM_Class3_Genome.Biol.2013_PMID.24220145	0.51838897	0.97224387	Positive	0.87779092	0.34667934
CIBERSORT_Mast_cells_resting_Nat.Methods.2015_PMID.25822800	-0.4219569	-0.9677114	Negative	0.87779092	0.34758983
ID4_Single_Gene	0.27338768	0.97088412	Positive	0.87779092	0.34815005
New.Immune_Tertiary.Lymphoid.Str.melanoma.9gene_Nature.2020_PMID.319	-0.5758803	-0.9677565	Negative	0.87779092	0.34829828
TLS_New_9Gene_Signature_PMID.31942071	-0.5758803	-0.9677565	Negative	0.87779092	0.34829828
Tcell_EXH_Effector_CD8_T_cell_at_day_8_p.i_Armstrong_vs_Naive_CD8_T_cell	-0.5066994	-0.9663063	Negative	0.87779092	0.34891687
HS_Red10_BMC.Med.Genomics.2011_PMID.21214954	-0.4153015	-0.9647168	Negative	0.87779092	0.34904242
Parity_signature_Troester_251_BCR.2014_PMID.25005139	-0.5012532	-0.9639105	Negative	0.87779092	0.35044464
New.Immune_ImSig.PlasmaCells_CancerImmunolRes.2018_PMID.30266715	0.46027058	0.96385021	Positive	0.87779092	0.35092827
Parity_signature_Troester_40_BCR.2014_PMID.25005139	-0.5029793	-0.963103	Negative	0.87779092	0.35104752
Pfefferle2012_MaSC_PMID.25575446	-0.3128815	-0.9604668	Negative	0.87779092	0.35111129
Shehata2012_Stroma_PMID.25575446	-0.4598069	-0.9581523	Negative	0.87779092	0.35248163
Myeloid_dendritic_cells_MCP_PMID.31942075_PMID.31942077	-0.5558477	-0.9564355	Negative	0.87779092	0.35308154
New.Immune_MInterferon_Cluster_BMC.Med.Genomics.2011_PMID.21214954	0.38953459	0.96009631	Positive	0.87779092	0.35376198
GSEA_GP11_Immune_IFN.r.0.965_PerouLab_MInterferon_Cluster_BMC_Med_(	0.38953459	0.96009631	Positive	0.87779092	0.35376198
New.Immune_ImSig.Macrophages_CancerImmunolRes.2018_PMID.30266715	-0.4597236	-0.9556586	Negative	0.87779092	0.35469944
ERBB3_Single_Gene	0.43677626	0.95636093	Positive	0.87779092	0.35475939
GATA3.induced.genes_Oncogene.2004_PMID.15361840	0.39115441	0.95281489	Positive	0.87779092	0.35580222
MUnknown_3_BMC.Med.Genomics.2011_PMID.21214954	-0.400556	-0.9477152	Negative	0.87779092	0.35736963
New.Immune_Charoentong_Type_1_T_helper_cell_CellRep.2017_PMID.280522	-0.5147631	-0.9469247	Negative	0.87779092	0.35871268
MET_DOWN_Significant_Genes_LOW_BASALS_1_Genes_JCI.2018_PMID.29480	-0.3075034	-0.9457624	Negative	0.87779092	0.35944178
MM_WapINT3.2012_Genome.Biol.2013_PMID.24220145	-0.4676944	-0.9459324	Negative	0.87779092	0.3596434
Pfefferle2012_Stroma_PMID.25575446	-0.4414754	-0.941922	Negative	0.87779092	0.36023814
IgG_Breast.Cancer.Research.2008_PMID.19272155	0.53238437	0.94289703	Positive	0.87779092	0.36090477
Spike2012_fStr_PMID.25575446	-0.4083338	-0.9400703	Negative	0.87779092	0.36115835
UNC_ROR_S_Model_JCO.2009_PMID.19204204	0.45118923	0.93925279	Positive	0.87779092	0.36156514
PDGFRB_Single_Gene	-0.4815077	-0.938114	Negative	0.87779092	0.3621323
CD103_Ratio_Cancer.Cell.2014_PMID.25446897	0.43886636	0.93872375	Positive	0.87779092	0.36221729
aStr_Lim09_BCR.2015_PMID.25575446	-0.4492002	-0.9361805	Negative	0.87779092	0.36347158
New.Immune_Miller_B_P_Metagene_16gene_Genome_Biol_2013_PMID.23618	0.54455629	0.93472555	Positive	0.87779092	0.36492929
Miller_B_P_Metagene_Genome_Biol_2013_PMID.23618380	0.54455629	0.93472555	Positive	0.87779092	0.36492929
New.Immune_Charoentong_Gamma_delta_T_cell_CellRep.2017_PMID.280522	-0.3500203	-0.9329701	Negative	0.87779092	0.36502101
MUnknown_26_BMC.Med.Genomics.2011_PMID.21214954	0.38784062	0.93373986	Positive	0.87779092	0.36528943
GSEA_GP18_Vesicle_EPR_membrane_coat.r.0.877_MEMBRANE_COAT	-0.3997452	-0.9343567	Negative	0.87779092	0.36553932
Bcells_Naive_J.Clin.Oncol.2015_PMID.25800755	-0.494513	-0.931812	Negative	0.87779092	0.36667501
HCK_Breast.Cancer.Research.2008_PMID.19272155	-0.438122	-0.9304056	Negative	0.87779092	0.36734751
Shehata2012_ALDHpos_PMID.25575446	0.30024767	0.931044	Positive	0.87779092	0.36788701
Lim.et.al.2010.Conserved.Luminal.Progenitor_BCR.2010_PMID.20346151	0.29765496	0.92575219	Positive	0.87779092	0.36886005
S100A9_A8_BMC.Med.Genomics.2011_PMID.21214954	0.31052548	0.92461391	Positive	0.87779092	0.36921316
Duke_Module13_myc_Mike_PMID.20335537	-0.4471925	-0.9233596	Negative	0.87779092	0.36997556
CIBERSORT_Dendritic_cells_resting_Nat.Methods.2015_PMID.25822800	-0.477206	-0.9216825	Negative	0.87779092	0.37136689
GSEA_GP12_Hypoxia_glycolosis.r.0.939_SEMENZA_HIF1_TARGETS	-0.3678005	-0.9179143	Negative	0.87779092	0.37276489
PR_Isoform_Ratio_Up_in_PRB_H_JNCI.2017_PMID.28376177	0.41564154	0.91622341	Positive	0.87779092	0.3731534
HS_Red3_BMC.Med.Genomics.2011_PMID.21214954	-0.43746	-0.9142851	Negative	0.87779092	0.37414014
Murat_G24_J.Clin.Oncol.2008_PMID.18565887	-0.4357437	-0.9141958	Negative	0.87779092	0.37535455
MHC_Forero_24_Cancer.Immunol.Res.2016_PMID.26980599	-0.4828182	-0.913439	Negative	0.87779092	0.37558269
Lim2009_Stroma_Adam_PMID.25575446	-0.4286605	-0.9104826	Negative	0.87779092	0.37642371
MUnknown_28_BMC.Med.Genomics.2011_PMID.21214954	-0.3634766	-0.9121025	Negative	0.87779092	0.37702857
Pfefferle2012_MatureLum_PMID.25575446	-0.1704388	-0.9067348	Negative	0.87779092	0.3783653
GSEA_BIOCARTA_AKT_PATHWAY	-0.4523747	-0.9061791	Negative	0.87779092	0.37882933
LOBULAR_TCGA_SIGNATURE_Immune_related_Cell.2015_PMID.26451490	-0.4300416	-0.9034643	Negative	0.87779092	0.38122304
MM_Red7_BMC.Med.Genomics.2011_PMID.21214954	-0.3448738	-0.9000593	Negative	0.87779092	0.38143613
New.Immune_Miller_Bcell_Plasma_52gene_PMID.23618380	0.49788736	0.90089274	Positive	0.87779092	0.38209355
HS_Green14_BMC.Med.Genomics.2011_PMID.21214954	-0.4711159	-0.8989417	Negative	0.87779092	0.38219372
IMMUNE_Bindea_Cell_Cytotoxic_cells_Immunity.2013_PMID.24138885	-0.4494802	-0.8992892	Negative	0.87779092	0.38270655
New.Immune_Charoentong_Activated_dendritic_cell_CellRep.2017_PMID.2805	-0.3600445	-0.9002095	Negative	0.87779092	0.38312111
EGFR_Single_Gene	-0.1656592	-0.8938076	Negative	0.87779092	0.38467251
GSEA_GP21_Anti_apoptosis_DNA_stability.r.0.877_MORF_BCL2	-0.2899617	-0.8937439	Negative	0.87779092	0.38682583
HS_Green22_BMC.Med.Genomics.2011_PMID.21214954	-0.4125102	-0.8933052	Negative	0.87779092	0.38691019

MAPK_pathway_activation_Wagle_NPJ.Precis.Oncol.2018_PMID.29872725	-0.4203068	-0.8922122	Negative	0.87779092	0.38746847
GO_DOWN_with_SOX10_OE_Cell.Rep.2015_PMID.26365194	0.29270174	0.88705746	Positive	0.87779092	0.39053362
T_regulatory_cell_2gene_PMID.31942077	0.36754853	0.88311925	Positive	0.87779092	0.39243471
MUnknown_23_BMC.Med.Genomics.2011_PMID.21214954	-0.4226107	-0.8805003	Negative	0.87779092	0.39314374
Bcells_Plasmablast_J.Clin.Oncol.2015_PMID.25800755	-0.4480437	-0.8769774	Negative	0.87779092	0.39347639
Scorr_PTEN_Present_Correlation_PNAS.2007_PMID.17452630	0.42511231	0.87343672	Positive	0.87779092	0.39534541
CIBERSORT_Macrophages_M0_Nat.Methods.2015_PMID.25822800	-0.3859085	-0.8755972	Negative	0.87779092	0.39537489
New.Immune_Miller_Monocyte_DendriticCell_25gene_PMID.23618380	-0.3968391	-0.8758364	Negative	0.87779092	0.39552193
MM_Red21_BMC.Med.Genomics.2011_PMID.21214954	0.4191275	0.8739055	Positive	0.87779092	0.39596837
TCGA.BRCA.1198_IMMUNOGLOBULIN_PMID.26451490	0.46626311	0.87159267	Positive	0.87779092	0.39732007
MS_CD44_DOWN_PNAS.2009_PMID.19666588	0.43668605	0.86925611	Positive	0.87779092	0.3982732
Pcorr_IGS_Correlation_NJEM.2007_PMID.17229949	-0.4027718	-0.8715621	Negative	0.87779092	0.3984796
Lums_HER2E_UP_metastatic.signature_JCI.2020_PMID.32573490	-0.4489938	-0.8681564	Negative	0.87779092	0.39879943
MASC_Up_Nat.Med.2009_PMID.19648928	-0.4316472	-0.8666543	Negative	0.87779092	0.39917626
New.Immune_ImmLandscape_Macro.mono_CSF1_core_response.Clin_Can_Res	-0.4285759	-0.8667214	Negative	0.87779092	0.40005603
TCGA_CSF1_response_Immunity.2018_PMID._29628290	-0.4285759	-0.8667214	Negative	0.87779092	0.40005603
African_and_European_Ancestry_in_TCGA_Negative_JAMA.Oncol.2017_PMID.2	-0.3793111	-0.865497	Negative	0.87779092	0.40094355
Interferon_Cluster_BMC.Med.Genomics.2011_PMID.21214954	0.31811832	0.8667579	Positive	0.87779092	0.40121278
Down_regulated_upon_N_RAS_repression_Cell_Rep.2015_PMID.26166574	-0.4104889	-0.8610131	Negative	0.87779092	0.40299199
LOBULAR_TCGA_SIGNATURE_Reactive_like_Cell.2015_PMID.26451490	0.32264378	0.86181333	Positive	0.87779092	0.40332114
MM_Normal.like_Genome.Biol.2013_PMID.24220145	-0.3342877	-0.8585183	Negative	0.87779092	0.4035983
MUnknown_11_BMC.Med.Genomics.2011_PMID.21214954	0.30588843	0.86011303	Positive	0.87779092	0.40468769
Scorr_PTEN_Absent_Correlation_PNAS.2007_PMID.17452630	-0.4150623	-0.8523139	Negative	0.87779092	0.4066175
MDACC.FNA.1_J.Clin.Oncol.2010_PMID.20805453	-0.4162362	-0.8494619	Negative	0.87779092	0.40977048
New.Immune_Charoentong_Plasmacytoid_dendritic_cell_CellRep.2017_PMID.2	-0.4078525	-0.8470728	Negative	0.87779092	0.4106817
Apocrine_Features_J.Pathol.2017_PMID.27861902	-0.3341369	-0.8434666	Negative	0.87779092	0.41140067
MM_BRCAwnt_1pFDR_UP_Genome.Biology.2007_PMID.17493263	-0.4066926	-0.8430182	Negative	0.87779092	0.41341658
New.Immune_Charoentong_MDSC_CellRep.2017_PMID.28052254	-0.4188258	-0.8419622	Negative	0.87779092	0.41342582
MNB1_BMC.Med.Genomics.2011_PMID.21214954	-0.3544131	-0.8405062	Negative	0.87779092	0.41376513
Stingl_Day7_Downregulated_Nat.Cell.Biol.2014_PMID.25173976	-0.4695855	-0.8397284	Negative	0.87779092	0.41422241
New.Immune_Charoentong_Effector_memeory_CD8_T_cell_CellRep.2017_PMI	-0.4643475	-0.8394706	Negative	0.87779092	0.41439104
UNC_Scorr_LumA_Correlation_JCO.2009_PMID.19204204	-0.3520154	-0.8367661	Negative	0.87779092	0.41504751
IMMUNE_Bindea_Cell_Mast_cells_Immunity.2013_PMID.24138885	-0.3377615	-0.8327928	Negative	0.87779092	0.41721987
Pcorr_Dasatinib_R_Correlation_Cancer.Res.2007_PMID.17332353	-0.3256669	-0.8332543	Negative	0.87779092	0.41751275
Melanoma_Scorr_Keratin_Cell.2015_PMID.26091043	-0.3582848	-0.8343314	Negative	0.87779092	0.4179348
Unknown_14_BMC.Med.Genomics.2011_PMID.21214954	-0.1921563	-0.8342325	Negative	0.87779092	0.41832412
Lim.et.al.2010.Conserved.aMaSC_BCR.2010_PMID.20346151	-0.4353937	-0.8288064	Negative	0.87779092	0.41971858
New.Immune_TCGA.BRCA.1198_immune_INTERFERON_Cell.2015_PMID.26451	0.31828588	0.83206668	Positive	0.87779092	0.4197937
TCGA.BRCA.1198_immune_INTERFERON_JCI.2020_PMID.32573490	0.31828588	0.83206668	Positive	0.87779092	0.4197937
TCGA.BRCA.1198_PDCHA_MANY_JCI.2020_PMID.32573490	0.25336168	0.82944971	Positive	0.87779092	0.4207225
fMaSC_Signature_CellRep.2018_PMID.30089273	-0.40875	-0.8240764	Negative	0.87779092	0.42294946
METAPLASTIC_Up_CanRes.2009_PMID.19435916	-0.3832569	-0.8220419	Negative	0.87779092	0.42313459
MM_Red8_BMC.Med.Genomics.2011_PMID.21214954	-0.3941548	-0.8251117	Negative	0.87779092	0.42314563
Stromal_Up_Nat.Med.2009_PMID.19648928	-0.4022806	-0.8197035	Negative	0.87805713	0.42472936
New.Immune_Charoentong_Regulatory_T_cell_CellRep.2017_PMID.28052254	-0.4063698	-0.8193666	Negative	0.87805713	0.42537456
Wahl_fSTR_Signature_Cell.Stem.Cell.2012_PMID.22305568	-0.3811597	-0.8066971	Negative	0.88940058	0.43193377
MacTh1_cluster_Iglesia_CCR.2014_PMID.24916698	-0.3872114	-0.8046741	Negative	0.89145242	0.43410737
MM_PyMT.2012_Genome.Biol.2013_PMID.24220145	-0.2985634	-0.8014683	Negative	0.89145242	0.43584994
Bcells_Centroblast_J.Clin.Oncol.2015_PMID.25800755	0.38787538	0.79875541	Positive	0.89145242	0.43612924
Claudin_High_Genome.Biol.2007_PMID.17493263	-0.4376604	-0.7965934	Negative	0.89270556	0.43804003
Mmyosin_BMC.Med.Genomics.2011_PMID.21214954	0.28120694	0.79327766	Positive	0.89270556	0.43922229
UNC_Scorr_Basal_Correlation_JCO.2009_PMID.19204204	0.24890359	0.79276172	Positive	0.89270556	0.4399458
Scorr_EMAT2_Correlation_BCR.2020_PMID.32641077	-0.3829115	-0.7913251	Negative	0.89323937	0.44127734
Excellent_Pathologic_Response_ER.Pos_39_JAMA.2011_PMID.21558518	0.28974847	0.79022412	Positive	0.89329247	0.44293461
GO_UP_with_SOX10_OE_Cell.Rep.2015_PMID.26365194	-0.3682385	-0.7867939	Negative	0.89329247	0.44419168
LumProg_Shehata_BCR.2015_PMID.25575446	-0.3706299	-0.7843705	Negative	0.89329247	0.44562674
MM_Red22_BMC.Med.Genomics.2011_PMID.21214954	-0.4063348	-0.7799832	Negative	0.89329247	0.44793413
MUnknown_2_BMC.Med.Genomics.2011_PMID.21214954	-0.3193595	-0.7774038	Negative	0.89329247	0.44826325
Bcells_Memory_J.Clin.Oncol.2015_PMID.25800755	-0.4106546	-0.7770223	Negative	0.89329247	0.44923415
Pcorr_Dasatinib_L_Correlation_Cancer.Res.2007_PMID.17332353	0.29692581	0.77602655	Positive	0.89329247	0.44960981
MM_Green12_BMC.Med.Genomics.2011_PMID.21214954	-0.4122031	-0.7746354	Negative	0.89329247	0.44985183
IMMUNE_Bindea_Cell_NK_CD56bright_cells_Immunity.2013_PMID.24138885	0.42108662	0.772508	Positive	0.89359865	0.45107491
Duke_Module04_e2f1_Mike_PMID.20335537	-0.3649582	-0.7664748	Negative	0.89795257	0.45563497
aStr_HsEnriched_Refined1_BCR.2015_PMID.25575446	-0.3822446	-0.7643714	Negative	0.89795257	0.45577185
AGE_associated_SOOD_Genome.Biol.2015_PMID.26343147	0.33515736	0.76377257	Positive	0.89795257	0.45656354
GSEA_GP5_MYC_targets_TERT.r.0.922_PerouLab_MM_Myc_1pFDR_UP_Genon	-0.3840863	-0.7629888	Negative	0.89795257	0.45756913
MM_Red24_BMC.Med.Genomics.2011_PMID.21214954	-0.1929455	-0.7575884	Negative	0.90047275	0.46041979
New.Immune_IMMUNE_Bindea_Cell_iDC_Median_Immunity.2013_PMID.2413	0.3628885	0.75130009	Positive	0.90047275	0.46384575
New.Immune_TCGA.BRCA.1198_immune_CD4_CD53_CD84_BTK_Cell.2015_PM	-0.3621543	-0.7508862	Negative	0.90047275	0.46487431
TCGA.BRCA.1198_immune_CD4_CD53_CD84_BTK_JCI.2020_PMID.32573490	-0.3621543	-0.7508862	Negative	0.90047275	0.46487431
MM_Red17_BMC.Med.Genomics.2011_PMID.21214954	-0.3980112	-0.7472729	Negative	0.90047275	0.46670394
Mouse_Human_ImmuneProfiles_SHAY_M_H_Induced_in_T_PNAS.2013_PMID.	-0.4909742	-0.7402529	Negative	0.90047275	0.46987064
African_and_European_Ancestry_in_TCGA_Positive_JAMA.Oncol.2017_PMID.2	-0.3039377	-0.7420041	Negative	0.90047275	0.47040981
X16.13_Amplicon_BMC.Med.Genomics.2011_PMID.21214954	-0.2237153	-0.7359565	Negative	0.90047275	0.47376432
MM_Green3_BMC.Med.Genomics.2011_PMID.21214954	-0.3264886	-0.7336042	Negative	0.90047275	0.47560164
TCGA.BRCA.1198_NORMAL_JCI.2020_PMID.32573490	-0.2886185	-0.729443	Negative	0.90047275	0.47627456
IMMUNE_Bindea_Cell_pDC_Immunity.2013_PMID.24138885	-0.3478	-0.7277144	Negative	0.90047275	0.47730346
M2_Macrophage_Blood.2006_PMID.16556895	-0.3522154	-0.7221928	Negative	0.90047275	0.48059892
Verhaak_Immune_Nat.Commun.2013_PMID.24113773	-0.3753515	-0.7229111	Negative	0.90047275	0.4810262
IMMUNE_Bindea_Cell_Th2_cells_Immunity.2013_PMID.24138885	0.37260695	0.72283201	Positive	0.90047275	0.48130383
Vascular.Content_Clin.Exp.Metastasis.2014_PMID.23975155	-0.2268407	-0.7229989	Negative	0.90047275	0.48136343
Immune_Suppression_Kardos_JCI.Insight.2016_PMID.27699256	-0.345271	-0.7215819	Negative	0.90047275	0.48194612
MM_Red19_BMC.Med.Genomics.2011_PMID.21214954	-0.3478482	-0.7193116	Negative	0.90047275	0.48294813
ADM_S100A10_A110NDGR1_Cluster_BMC.Med.Genomics.2011_PMID.212149	0.1982749	0.7198788	Positive	0.90047275	0.48347527
Duke_Module21_tgfb_Mike_PMID.20335537	-0.2937445	-0.7185162	Negative	0.90047275	0.48400765
HER1_Cluster3_BMC.Genomics.2007_PMID.17663798	-0.3719916	-0.7166076	Negative	0.90047275	0.48429041
PDCD1_Single_Gene	-0.349327	-0.7171032	Negative	0.90047275	0.48432624
Late_IRS_2_PLoS.One.2016_PMID.26991655	-0.335662	-0.7160833	Negative	0.90047275	0.48457329
GSEA_IGF1R_PATHWAY_BIOCARTA	-0.3575542	-0.7151897	Negative	0.90047275	0.48479805
MHistone_BMC.Med.Genomics.2011_PMID.21214954	-0.4208138	-0.7116799	Negative	0.90047275	0.48691073
MNB3_BMC.Med.Genomics.2011_PMID.21214954	-0.3237143	-0.7097015	Negative	0.90047275	0.48890946

Inflammatory_Breast_Cancer_79_nIBC_CCR.2013_PMID.23396049	-0.3013734	-0.7066253	Negative	0.90047275	0.49111922
Knudsen_Neo_ER_positive_Clin.Cancer.Res.2014_PMID.25047707	0.39703569	0.70457425	Positive	0.90047275	0.49120458
Unknown_1_BMC.Med.Genomics.2011_PMID.21214954	-0.2928383	-0.7034424	Negative	0.90047275	0.49189059
GSEA_GP20_TAL1_Leukemia_erythropoiesis.r.0.935_GNF2_TAL1	-0.301117	-0.7049223	Negative	0.90047275	0.49201563
New.Immune_TCGA.BRCA.1198_IL8_CCL_Cell.2015_PMID.26451490	-0.2828873	-0.7035507	Negative	0.90047275	0.49224408
TCGA.BRCA.1198_IL8_CCL_JCI.2020_PMID.32573490	-0.2828873	-0.7035507	Negative	0.90047275	0.49224408
Duke_Module14_p53_Mike_PMID.20335537	-0.2853673	-0.7023977	Negative	0.90058614	0.49386649
New.Immune_ImSig.InterferonPathway_CancerImmunolRes.2018_PMID.30266	0.26356998	0.70217658	Positive	0.90058614	0.49446057
MM_Red4_BMC.Med.Genomics.2011_PMID.21214954	-0.3468609	-0.6960081	Negative	0.90089091	0.49733844
GSEA_GP17_Basal_signaling.r.0.958_SMID_BREAST_CANCER_BASAL_UP	0.21670162	0.69556011	Positive	0.90089091	0.49771097
TCGA.BRCA.1198_UNKNOWN1_JCI.2020_PMID.32573490	0.26223648	0.6936311	Positive	0.90089091	0.49786076
STAT3_Basal_Horvath_PNAS.2014_PMID.25139989	-0.2805503	-0.6894886	Negative	0.90332926	0.50193531
Tcell_EXH_Exhausted_CD8_T_cell_vs_Naive_CD8_T_cell_Metagene_1_Science.	-0.3367866	-0.6881594	Negative	0.90332926	0.50195521
Shehata2012_LumProg_PMID.25575446	-0.2713611	-0.6874154	Negative	0.90332926	0.50244989
Unknown_6_BMC.Med.Genomics.2011_PMID.21214954	-0.330015	-0.683694	Negative	0.90511404	0.5052745
GENE_PANEL_UNCSEQ_7.1_Oncologist.2018_PMID.29158372	-0.3907588	-0.6835105	Negative	0.90511404	0.50560796
X12qMDM4_BMC.Med.Genomics.2011_PMID.21214954	-0.3328527	-0.6798812	Negative	0.90514949	0.50671048
MM_Green13_BMC.Med.Genomics.2011_PMID.21214954	0.32047934	0.66899795	Positive	0.91530424	0.51349006
Activate.Endothelium_Clin.Exp.Metastasis.2014_PMID.23975155	-0.2643554	-0.6588868	Negative	0.91772322	0.51933898
fMaSC.refined1_PMID.25575446	0.18859987	0.66035388	Positive	0.91772322	0.5196785
Stingl_Up_Proliferation_Nat.Cell.Biol.2014_PMID.25173976	0.34756231	0.65672815	Positive	0.91772322	0.5206905
PARP_Sensitivity_Signature_POSITIVE_Sci.Adv.2017_PMID.28439535	-0.3752067	-0.6570406	Negative	0.91772322	0.52133913
TCGA.BRCA.1198_UNKNOWN3_JCI.2020_PMID.32573490	-0.3423551	-0.6561365	Negative	0.91772322	0.52138387
MUnknown_22_BMC.Med.Genomics.2011_PMID.21214954	-0.3229542	-0.6566678	Negative	0.91772322	0.52208245
MUnknown_21_BMC.Med.Genomics.2011_PMID.21214954	-0.3151292	-0.6534179	Negative	0.91772322	0.52385889
Lim2010_Stroma_Adam_PMID.25575446	-0.3242115	-0.6516534	Negative	0.91772322	0.52423642
STAT1_Breast.Cancer.Research.2008_PMID.19272155	0.25755603	0.649535	Positive	0.91772322	0.52671703
MM_Red18_BMC.Med.Genomics.2011_PMID.21214954	0.33625615	0.64601487	Positive	0.91772322	0.52742747
TCGA.BRCA.1198_MYBL2_APOBEC3B_JCI.2020_PMID.32573490	0.32982846	0.64390214	Positive	0.91772322	0.52876178
HS_Red25_BMC.Med.Genomics.2011_PMID.21214954	-0.1934554	-0.6386019	Negative	0.91772322	0.53315444
HS_Green2_BMC.Med.Genomics.2011_PMID.21214954	-0.3023845	-0.6351209	Negative	0.91772322	0.53432773
GSEA_GP14_Plasma_membrane_cell_cell_signaling.r.0.925_MORF_CNTN1	0.30468383	0.63578457	Positive	0.91772322	0.53526463
X1p36_Amplicon_BMC.Med.Genomics.2011_PMID.21214954	-0.2713403	-0.633641	Negative	0.91772322	0.53637491
MM_Green11_BMC.Med.Genomics.2011_PMID.21214954	-0.3025037	-0.6286944	Negative	0.91772322	0.53906496
HS_Red4_BMC.Med.Genomics.2011_PMID.21214954	-0.3292296	-0.6285148	Negative	0.91772322	0.53945378
Unknown_13_BMC.Med.Genomics.2011_PMID.21214954	-0.2762775	-0.6285653	Negative	0.91772322	0.5397689
X4p16_Amplicon_BMC.Med.Genomics.2011_PMID.21214954	-0.2628051	-0.6260099	Negative	0.91772322	0.54013682
REPLICATION_STRESS_MODEL_Cell.Rep.2018_PMID.29768207	-0.2454277	-0.6242309	Negative	0.91772322	0.54127513
Pcorr_Adenocarcinoma_JMD.2013_PMID.23701907	0.20793273	0.62513169	Positive	0.91772322	0.54226904
BCL2_Single_Gene	-0.2859123	-0.6189733	Negative	0.91772322	0.54464695
New.Immune_ImSig.NKcells_CancerImmunolRes.2018_PMID.30266715	-0.3208419	-0.6167648	Negative	0.91772322	0.54638862
MM_Red11_BMC.Med.Genomics.2011_PMID.21214954	-0.3220295	-0.6168276	Negative	0.91772322	0.54708704
LumB_Her2_JCI.2020_PMID.32573490	0.11071698	0.61580769	Positive	0.91772322	0.54767466
Extensive_Residual_Diesase_ER.Pos_73_JAMA.2011_PMID.21558518	-0.2634464	-0.6160877	Negative	0.91772322	0.54789886
MBASAL_BMC.Med.Genomics.2011_PMID.21214954	-0.3423314	-0.6130694	Negative	0.91772322	0.54867337
New.Immune_Charoentong_Natural_killer_T_cell_CellRep.2017_PMID.2805225	-0.3109782	-0.6108309	Negative	0.91772322	0.55039572
Chemo_Endocrine_Score_CC_.2.LumA_subtract.by_CC.2.Basal_Clin.Cancer.Res.	0.32745769	0.60584995	Positive	0.91772322	0.55311275
myeloid_cell_chemotaxis_1gene_PMID.31942077	-0.2946023	-0.6071446	Negative	0.91772322	0.55348613
X3p21Amplicon_BMC.Med.Genomics.2011_PMID.21214954	0.25047	0.60407703	Positive	0.91772322	0.55426186
IMMUNE_Bindea_Cell_B_cells_Immunity.2013_PMID.24138885	-0.2630874	-0.6043717	Negative	0.91772322	0.55518212
New.Immune_Tcell_CD8_Memory_vs_naive_1.PMID27789795	-0.3142075	-0.6035249	Negative	0.91772322	0.55522444
Tcell_EXH_Memory_CD8_T_cell_a_vs_Naive_CD8_T_cell_Metagene_1_Science.	-0.3142075	-0.6035249	Negative	0.91772322	0.55522444
MM_Normal_1pFDR_UP_Genome.Biology.2007_PMID.17493263	-0.2742685	-0.6019599	Negative	0.91772322	0.55563574
Pcorr_magnoid_PLOS.2012_PMID.22590557	0.35209094	0.59899786	Positive	0.91772322	0.55774726
Mature_LuminaUp_Nat.Med.2009_PMID.19648928	0.14206623	0.59947532	Positive	0.91772322	0.55780207
TCGA.BRCA.1198_LUMINAL_JCI.2020_PMID.32573490	0.11051077	0.59847608	Positive	0.91772322	0.55790047
CIBERSORT_Dendritic_cells_activated_Nat.Methods.2015_PMID.25822800	-0.2822233	-0.5990662	Negative	0.91772322	0.55870342
Unknown_4_BMC.Med.Genomics.2011_PMID.21214954	-0.2916968	-0.598871	Negative	0.91772322	0.55875732
GSEA_PI3K_CASCADE_REACTOME	-0.1766313	-0.5946357	Negative	0.91918686	0.56195493
MM_Green7_BMC.Med.Genomics.2011_PMID.21214954	0.27744689	0.58932974	Positive	0.91918686	0.56513904
HS_Green6_BMC.Med.Genomics.2011_PMID.21214954	-0.2996327	-0.5823411	Negative	0.91918686	0.56933452
CD4_Single_Gene	-0.269691	-0.5822224	Negative	0.91918686	0.56963249
LumA_Basal_JCI.2020_PMID.32573490	0.1741871	0.57889969	Positive	0.91918686	0.57112028
MM_Class8_Genome.Biol.2013_PMID.24220145	-0.3061782	-0.5785949	Negative	0.91918686	0.57112057
New.Immune_Bcell_Tcell_Cooperation_Hollern_Cell_2019_PMID.31730857	0.24301235	0.57854926	Positive	0.91918686	0.57189672
Bcell_Tcell_Cooperation_Cell.2019_PMID.31730857	0.24301235	0.57854926	Positive	0.91918686	0.57189672
Immune_Hot_using_CD8_vs_Cold_PMID.31942071	-0.2984034	-0.5766614	Negative	0.91918686	0.57303281
PR_Isoform_Ratio_Up_in_PRA_H_JNCI.2017_PMID.28376177	0.23296718	0.57479221	Positive	0.91918686	0.57443539
MatureLum_Shehata_BCR.2015_PMID.25575446	-0.1923677	-0.5698411	Negative	0.91918686	0.57809852
Scorr_IIE_Correlation_JCO.2006_PMID.16505416	0.21818467	0.5652034	Positive	0.91918686	0.58069975
MUnknown_30_BMC.Med.Genomics.2011_PMID.21214954	0.20783662	0.56363233	Positive	0.91918686	0.58081885
New.Immune_TCGA.BRCA.1198_immune_CD19_Cell.2015_PMID.26451490	-0.2389445	-0.5605139	Negative	0.91918686	0.58332213
TCGA.BRCA.1198_immune_CD19_JCI.2020_PMID.32573490	-0.2389445	-0.5605139	Negative	0.91918686	0.58332213
T_RM_Cell_single_cell_signature_Savas_Nat_Med.2018_PMID.29942092	-0.2683868	-0.5568088	Negative	0.91918686	0.58599474
IMMUNE_Bindea_Cell_aDC_Immunity.2013_PMID.24138885	0.27194555	0.55746	Positive	0.91918686	0.58611405
MM_Myoepithelioma.like_Genome.Biol.2013_PMID.24220145	-0.3047469	-0.5555881	Negative	0.91918686	0.58617791
LumProg_HsEnriched_Refined1_BCR.2015_PMID.25575446	0.20934025	0.55414994	Positive	0.91918686	0.58768973
Lim2009_MaSC_Adam_PMID.25575446	0.23417124	0.55275638	Positive	0.91918686	0.588348
HS_Green8_BMC.Med.Genomics.2011_PMID.21214954	-0.0992536	-0.5522837	Negative	0.91918686	0.58919317
HouseKeeping_Genome.Biol.2004_PMID.15287981	-0.2549785	-0.5463465	Negative	0.91918686	0.5923655
CIBERSORT_NK_cells_resting_Nat.Methods.2015_PMID.25822800	-0.3042074	-0.5440478	Negative	0.91918686	0.59416185
Pcorr_NKI70_Good_Correlation_Nature.2002_PMID.11823860	-0.1951477	-0.5423698	Negative	0.91918686	0.59503814
MUnknown_20_BMC.Med.Genomics.2011_PMID.21214954	0.1989493	0.54265158	Positive	0.91918686	0.59579876
GSEA_GP2_Immune_Tcell_Bcell.r.0.949_PerouLab_Immune_cell_Cluster_BMC_	-0.281388	-0.5401896	Negative	0.91918686	0.5971344
Chromogramin_BMC.Med.Genomics.2011_PMID.21214954	0.29528996	0.53915358	Positive	0.91918686	0.59767875
UNC_Scorr_LumB_Correlation_JCO.2009_PMID.19204204	-0.1521952	-0.5358781	Negative	0.91918686	0.60056918
MM_Green25_BMC.Med.Genomics.2011_PMID.21214954	-0.2338354	-0.5332085	Negative	0.91918686	0.60121806
FOXC1_Single_Gene	-0.1456785	-0.5317809	Negative	0.91918686	0.60218391
XRT_inducedgenes_Oh_PMID.24527691	0.13005413	0.53265961	Positive	0.91918686	0.60264413
HS_Green16_BMC.Med.Genomics.2011_PMID.21214954	-0.2544031	-0.5319135	Negative	0.91918686	0.60295029
TLS_tumors_w_TLS_and_CD8_v_CD8alone_PMID.31942071	-0.3274857	-0.5304246	Negative	0.91918686	0.60349815

GSEA_GP19_1Q_amplicon.r.0.967_PerouLab_HS_Green17_BMC_Med_Genomi	0.20222425	0.53108792	Positive	0.91918686	0.60397423
MatureLum_Prat_BCR.2015_PMID.25575446	-0.1491731	-0.5290066	Negative	0.91918686	0.60529937
HS_Red20_BMC.Med.Genomics.2011_PMID.21214954	-0.2916794	-0.5240181	Negative	0.91918686	0.60801985
GSEA_PD1_SIGNALING_REACTOME	-0.2935105	-0.5229491	Negative	0.91918686	0.60863666
FOXA1_Single_Gene	-0.1691341	-0.5227889	Negative	0.91918686	0.60908189
HS_Green19_BMC.Med.Genomics.2011_PMID.21214954	0.2793574	0.52231592	Positive	0.91918686	0.60949795
IMMUNE_Bindea_Cell_Lymph_vessels_Immunity.2013_PMID.24138885	-0.2296836	-0.5154305	Negative	0.91918686	0.61349844
VEGF_13genes_BMC.Med.2009_PMID.19291283	0.16915745	0.51621543	Positive	0.91918686	0.61373376
TNBC_Clinically_Relevant_Good.26_BCR.2011_PMID.21978456	0.27731135	0.51570969	Positive	0.91918686	0.61374389
AMPH_EPIREGULIN_Cluster_BMC.Med.Genomics.2011_PMID.21214954	-0.214082	-0.5150436	Negative	0.91918686	0.61421896
MUnknown_33_BMC.Med.Genomics.2011_PMID.21214954	-0.2128927	-0.5134789	Negative	0.91918686	0.61501618
MM_Green14_BMC.Med.Genomics.2011_PMID.21214954	0.22928927	0.5128027	Positive	0.91918686	0.61546641
IMMUNE_Bindea_Cell_NK_cells_Immunity.2013_PMID.24138885	-0.2947968	-0.5109702	Negative	0.91918686	0.61635001
MASC_Down_Nat.Med.2009_PMID.19648928	-0.1837308	-0.5121182	Negative	0.91918686	0.61652621
MUnknown_5_BMC.Med.Genomics.2011_PMID.21214954	0.15585231	0.50868306	Positive	0.91918686	0.61791662
Unknown_5_BMC.Med.Genomics.2011_PMID.21214954	0.20231775	0.5092814	Positive	0.91918686	0.61871836
MITO1_BMC.Med.Genomics.2011_PMID.21214954	-0.2249203	-0.5062915	Negative	0.91918686	0.61996337
New.Immune_ImSig.Tcells_CancerImmunolRes.2018_PMID.30266715	-0.2972853	-0.5048245	Negative	0.91918686	0.62093196
GSEA_GP1_Proliferation_DNA_repair.r.0.981_PUJANA_CHEK2_PCC_NETWORK	-0.2771494	-0.5040239	Negative	0.91918686	0.62145428
Ductal_Carcinoma_In_Situ_J.Pathol.2017_PMID.27861902	0.27167143	0.50247539	Positive	0.91918686	0.62242861
MUnknown_25_BMC.Med.Genomics.2011_PMID.21214954	-0.2344702	-0.5030619	Negative	0.91918686	0.62244132
GSEA_GP7_Estrogen_signaling.r.0.97_SMID_BREAST_CANCER_BASAL_DN	-0.1185444	-0.5018258	Negative	0.91918686	0.62304591
TCGA.BRCA.1198_TP63_JCI.2020_PMID.32573490	0.17627579	0.49929235	Positive	0.91918686	0.62496466
New.Immune_Charoentong_Activated_CD8_T_cell_CellRep.2017_PMID.280522	-0.3251185	-0.4980898	Negative	0.91918686	0.62544321
New.Immune_Charoentong_Type_2_T_helper_cell_CellRep.2017_PMID.280522	0.12431443	0.49821397	Positive	0.91918686	0.62632838
TCGA_CHANG_CORE_SERUM_RESPONSE_UP_Immunity.2018_PMID._2962829	-0.2317738	-0.4956047	Negative	0.91918686	0.62691134
Stromal_Central_Fibrotic_Focus_J.Pathol.2017_PMID.27861902	-0.1143554	-0.4926509	Negative	0.91918686	0.63018338
CD8A_Single_Gene	-0.2299596	-0.4911078	Negative	0.91918686	0.63086448
MET_UP_RNA_Seq_Significant_Genes_JCI.2018_PMID.29480819	0.17693483	0.49018803	Positive	0.91918686	0.63170882
MERCK_Immune_signature_Science.2018_PMID.30309915	-0.2319396	-0.4897448	Negative	0.91918686	0.63179623
MatureLum_HsEnriched_BCR.2015_PMID.25575446	0.08839033	0.48666556	Positive	0.91918686	0.63341966
aMaSC_Lim09_BCR.2015_PMID.25575446	-0.1929219	-0.4856665	Negative	0.91918686	0.6343982
MUnknown_17_BMC.Med.Genomics.2011_PMID.21214954	0.26623523	0.48253905	Positive	0.91918686	0.63595844
Pcorr_basal_CCR.2010_PMID.20643781	0.17813475	0.48322647	Positive	0.91918686	0.63602559
New.Immune_Tcell_CD8_Exhausted_vs_antiPDL1_2.PMID27789795	0.23958308	0.48204147	Positive	0.91918686	0.63630417
Monocytic_lineage_MCP_PMID.31942075_PMID.31942077	-0.2251083	-0.4824652	Negative	0.91918686	0.63667544
Duke_Module22_tnfa_Mike_PMID.20335537	-0.2104658	-0.4795869	Negative	0.91918686	0.63870234
Pcorr_primitive_CCR.2010_PMID.20643781	-0.1684627	-0.4798077	Negative	0.91918686	0.63873441
TNBC_Clinically_Relevant_Poor.26_BCR.2011_PMID.21978456	0.21968185	0.47689806	Positive	0.91918686	0.63988305
HS_Green3_BMC.Med.Genomics.2011_PMID.21214954	0.18482615	0.47591416	Positive	0.91918686	0.64056872
MM_C3Tag.2012_Genome.Biol.2013_PMID.24220145	0.22677262	0.46761833	Positive	0.91918686	0.6463633
MM_Red25_BMC.Med.Genomics.2011_PMID.21214954	0.23989077	0.46593269	Positive	0.91918686	0.6475436
YALE_RHOA_pathway_Ann.Oncol.2017_PMID.28177460	-0.2400596	-0.4659384	Negative	0.91918686	0.64798116
RB_LOH_Breast.Cancer.Res.2008_PMID.18782450	0.23540077	0.46468381	Positive	0.91918686	0.64841871
Mouse_Human_ImmuneProfiles_SHAY_M_H_Induced_in_HSC_PNAS.2013_PMI	-0.2378143	-0.4647643	Negative	0.91918686	0.64895131
IMS_Score_Clin.Cancer.Res.2018_PMID.29921729	-0.1555612	-0.4638708	Negative	0.91918686	0.65002328
Pcorr_Breast2Lung_LM2_Correlation_Nature.2005_PMID.16049480	-0.2237386	-0.4555768	Negative	0.91918686	0.65481613
New.Immune_Miller_Tcell_NKcell_51gene_PMID.23618380	-0.2812731	-0.4555053	Negative	0.91918686	0.65512902
Late_IRS_1_PLoS.One.2016_PMID.26991655	0.20951231	0.45448234	Positive	0.91918686	0.65558685
IMMUNE_Bindea_Cell_Eosinophils_Immunity.2013_PMID.24138885	-0.2413455	-0.4530459	Negative	0.91918686	0.65717296
GSEA_BIOCARTA_CTLA4_PATHWAY	-0.2577398	-0.4519145	Negative	0.91918686	0.65763242
MCF7.E2.induced.genes_JCO.2006_PMID.16505416	-0.2105323	-0.4502084	Negative	0.91918686	0.65860041
Prosigna_Proliferation_18_BMC.Med.Genomics.2015_PMID.26297356	0.231788	0.44989437	Positive	0.91918686	0.65882206
New.Immune_TCGA.BRCA.1198_immune_GIMAP_IL16_Cell.2015_PMID.26451	-0.2702156	-0.4496497	Negative	0.91918686	0.65920839
TCGA.BRCA.1198_immune_GIMAP_IL16_JCI.2020_PMID.32573490	-0.2702156	-0.4496497	Negative	0.91918686	0.65920839
LumA_LumB_JCI.2020_PMID.32573490	0.23800846	0.44911556	Positive	0.91918686	0.65937194
Knudsen_Neo_common_Clin.Cancer.Res.2014_PMID.25047707	0.22341462	0.44911464	Positive	0.91918686	0.65937258
IntClust_Amplification.50.Better.than_Genome.Biol.2014_PMID.25164602	-0.2188171	-0.4414412	Negative	0.91918686	0.6656709
MM_Green19_BMC.Med.Genomics.2011_PMID.21214954	0.22167846	0.44021094	Positive	0.91918686	0.66567328
Pcorr_bronchioid_PLOS.2012_PMID.22590557	-0.2227754	-0.4373351	Negative	0.91918686	0.66771394
HS_Green10_BMC.Med.Genomics.2011_PMID.21214954	0.22397169	0.43698411	Positive	0.91918686	0.66796318
Duke_Module17_pr_Mike_PMID.20335537	-0.1419066	-0.4299538	Negative	0.91918686	0.67381994
MM_Red14_BMC.Med.Genomics.2011_PMID.21214954	-0.147793	-0.4287116	Negative	0.91918686	0.67465125
Scorr_S329_R_Correlation_Br.J.Cancer.2008_PMID.18382427	-0.1670464	-0.428706	Negative	0.91918686	0.67473648
MUnknown_34_BMC.Med.Genomics.2011_PMID.21214954	-0.1964317	-0.4258597	Negative	0.91918686	0.67646563
CIBERSORT_NK_cells_activated_Nat.Methods.2015_PMID.25822800	-0.2477979	-0.4236811	Negative	0.91918686	0.67769846
LOBULAR_TCGA_SUBTYPE_Proliferative_Cell.2015_PMID.26451490	0.15988727	0.42315955	Positive	0.91918686	0.67835549
TLS_Known_MARKERS_PMID.31942071	-0.2514945	-0.4201664	Negative	0.91918686	0.6802274
Miller_Proliferation_Metagene_Genome_Biol_2013_PMID.23618380	0.21377938	0.41923043	Positive	0.91918686	0.68062212
MNB2_BMC.Med.Genomics.2011_PMID.21214954	0.13633548	0.41961011	Positive	0.91918686	0.68096756
LCK_Breast.Cancer.Research.2008_PMID.19272155	-0.2386691	-0.4178197	Negative	0.91918686	0.68194777
HS_Red23_BMC.Med.Genomics.2011_PMID.21214954	0.21854	0.41729686	Positive	0.91918686	0.68200689
Stromal_Inflammation_J.Pathol.2017_PMID.27861902	-0.1930896	-0.4163302	Negative	0.91918686	0.68323443
CIBERSORT_T_cells_gamma_delta_Nat.Methods.2015_PMID.25822800	-0.2328538	-0.414415	Negative	0.91918686	0.68429443
IMMUNE_PEREZ_87_JCO.2015_PMID.2560586	-0.2421085	-0.4138454	Negative	0.91918686	0.68487579
CIBERSORT_B_cells_naive_Nat.Methods.2015_PMID.25822800	-0.2128663	-0.4118877	Negative	0.91918686	0.68640629
New.Immune_ImSig.Bcells_CancerImmunolRes.2018_PMID.30266715	-0.1737258	-0.4104796	Negative	0.91918686	0.68750482
CD3E_Single_Gene	-0.2476361	-0.4029255	Negative	0.91918686	0.69246324
MM_Green23_BMC.Med.Genomics.2011_PMID.21214954	0.18263777	0.40308191	Positive	0.91918686	0.69275891
TCGA_Tgd_cells_Immunity.2018_PMID._29628290	0.21366769	0.40197652	Positive	0.91918686	0.69301999
IMMUNE_Bindea_Cell_Tgd_Immunity.2013_PMID.24138885	0.21366769	0.40197652	Positive	0.91918686	0.69301999
HS_Green23_BMC.Med.Genomics.2011_PMID.21214954	-0.2372777	-0.3988891	Negative	0.91918686	0.69524816
Duke_Module12_lacticacidosis_Mike_PMID.20335537	-0.1320368	-0.3999051	Negative	0.91918686	0.69548664
CIBERSORT_T_cells_regulatory_Tregs_Nat.Methods.2015_PMID.25822800	-0.2325214	-0.3983795	Negative	0.91918686	0.69597034
T_cell_activation_PMID.31942077	0.15217339	0.39885064	Positive	0.91918686	0.69616043
JANES_Oscillation_GDF11_TGFBR3_Nat.Cell.Biol.2014_PMID.24658685	0.22272291	0.39469308	Positive	0.91918686	0.69849834
PIK3CA.Hutti_Cancer.Res.2012_PMID.22552288	-0.1678046	-0.3938624	Negative	0.91918686	0.69888201
New.Immune_ImmuneActive_Hollern_Cell_2019_PMID.31730857	-0.2220287	-0.3935087	Negative	0.91918686	0.69952696
DAN_ImmuneActive_Cell.2019_PMID.31730857	-0.2220287	-0.3935087	Negative	0.91918686	0.69952696
CD3D_Single_Gene	-0.2402531	-0.3885938	Negative	0.91918686	0.70269884
MM_DMBAwnt_1pFDR_UP_Genome.Biology.2007_PMID.17493263	-0.2071634	-0.3867965	Negative	0.91918686	0.704119

Duke_Module01_acidosis_Mike_PMID.20335537	-0.2350864	-0.3864196	Negative	0.91918686	0.70443666
Glycolysis_Signature_BMC.Med.2009_PMID.19291283	0.13774619	0.38642887	Positive	0.91918686	0.70498896
LumA_Her2_JCI.2020_PMID.32573490	0.15385615	0.38418786	Positive	0.91918686	0.70589711
Unknown_9_BMC.Med.Genomics.2011_PMID.21214954	0.15793093	0.38470009	Positive	0.91918686	0.70634564
bMYB_Signature_Oncogene.2009_PMID.19043454	0.19769692	0.38300407	Positive	0.91918686	0.70675739
MM_p53null.Basal_Genome.Biol.2013_PMID.24220145	0.1372559	0.38115661	Positive	0.91918686	0.70837334
UNC_Scorr_Norm_Correlation_JCO.2009_PMID.19204204	-0.1621609	-0.3796601	Negative	0.91918686	0.7095615
HS_Green25_BMC.Med.Genomics.2011_PMID.21214954	-0.0515077	-0.378077	Negative	0.91918686	0.71034235
Bcell_IL10_MINUS_LIN_J_Immunol.2014_PMID.25080484	-0.1554607	-0.3750758	Negative	0.91918686	0.7129462
Scorr_IE_Correlation_JCO.2006_PMID.16505416	-0.1587064	-0.373474	Negative	0.91918686	0.71399218
New.Immune_Charoentong_Type_17_T_helper_cell_CellRep.2017_PMID.28052	-0.1264645	-0.3737524	Negative	0.91918686	0.7143003
Wirapati_Proliferation_Breast.Cancer.Res.2008_PMID.18662380	0.19554477	0.37222328	Positive	0.91918686	0.71461073
Bcell_IL10_PLUS_LIN_J_Immunol.2014_PMID.25080484	-0.1938882	-0.3723779	Negative	0.91918686	0.71499076
MS_CD44_UP_PNAS.2009_PMID.19666588	-0.1509497	-0.3711442	Negative	0.91918686	0.71590586
NK_cells_MCP_PMID.31942075_PMID.31942077	-0.1602658	-0.3687521	Negative	0.91918686	0.71757487
Shehata2012_ALDHneg_PMID.25575446	0.12552563	0.36794695	Positive	0.91918686	0.71868682
UNC_Proliferation_11_Mean_JCO.2009_PMID.19204204	0.18763692	0.36590045	Positive	0.91918686	0.71923217
New.Immune_Charoentong_Activated_B_cell_CellRep.2017_PMID.28052254	-0.169306	-0.3659746	Negative	0.91918686	0.71982703
New.Immune_MHCI_coreGenes_PMID29170503	0.14481348	0.36378081	Positive	0.91918686	0.72153042
HS_Green21_BMC.Med.Genomics.2011_PMID.21214954	0.13593745	0.36129564	Positive	0.91918686	0.72309573
HGF_down_BCR.2013_PMID.24025166	-0.1835317	-0.3586226	Negative	0.91918686	0.72508178
New.Immune_Miller_T_NK_Metagene_34gene_Genome_Biol_2013_PMID.236	-0.2093111	-0.3571959	Negative	0.91918686	0.72591926
Miller_T_NK_Metagene_Genome_Biol_2013_PMID.23618380	-0.2093111	-0.3571959	Negative	0.91918686	0.72591926
Endothelial_Tumor_EC_high_Angiogenesis_2014_PMID.24257808	0.21058215	0.35665782	Positive	0.91918686	0.72600797
TNBC_Clinically_Relevant_Good.230_BCR.2011_PMID.21978456	-0.1181969	-0.3547714	Negative	0.91918686	0.72739382
CIBERSORT_B_cells_memory_Nat.Methods.2015_PMID.25822800	-0.1767315	-0.355098	Negative	0.91918686	0.72769991
New.Immune_Tcell_CD8_Exhausted_vs_naive_2.PMID27789795	0.18712631	0.35358034	Positive	0.91918686	0.72826935
New.Immune_ImSig.ProliferationPathway_CancerImmunolRes.2018_PMID.302	0.18012138	0.35012679	Positive	0.91918686	0.73081015
MM_Wnt1.Late_Genome.Biol.2013_PMID.24220145	-0.1983062	-0.3483777	Negative	0.91918686	0.73209818
New.Immune_Tcell_CD8_Effector_vs_naive_2.PMID27789795	0.181264	0.34564351	Positive	0.91918686	0.73411336
Shehata2012_NCL_PMID.25575446	0.0595062	0.34662395	Positive	0.91918686	0.73418051
Response_to_PARP_inhibitor_olaparib_Sensitivity_BC_Res_Treat_2012_PMID.2	-0.1592743	-0.3439989	Negative	0.91918686	0.73542754
MET_UP_Significant_Genes_HIGH_BASALS_Genes_JCI.2018_PMID.29480819	-0.0936523	-0.3444024	Negative	0.91918686	0.73593639
IMMUNE_Bindea_Cell_Th1_cells_Immunity.2013_PMID.24138885	0.18728421	0.3434516	Positive	0.91918686	0.73640766
MM_Red16_BMC.Med.Genomics.2011_PMID.21214954	-0.1607038	-0.3414967	Negative	0.91918686	0.737397
MUnknown_9_BMC.Med.Genomics.2011_PMID.21214954	-0.1985485	-0.3393707	Negative	0.91918686	0.73874411
MCD3_CD8_BMC.Med.Genomics.2011_PMID.21214954	-0.1972735	-0.338748	Negative	0.91918686	0.73947016
Proliferation_Cluster_BMC.Med.Genomics.2011_PMID.21214954	0.17254108	0.33744866	Positive	0.91918686	0.74016512
Up_regulated_by_Oncogenic_N_RAS_Cell_Rep.2015_PMID.26166574	0.1865005	0.33725898	Positive	0.91918686	0.7405099
RB_LOSS_J.Clin.Invest.2007_PMID.17160137	0.17649246	0.33633043	Positive	0.91918686	0.74099229
TCGA.BRCA.1198_HISTONES_JCI.2020_PMID.32573490	-0.1686046	-0.3362296	Negative	0.91918686	0.74106692
GSEA_BIOCARTA_VEGF_PATHWAY	-0.1919962	-0.3326875	Negative	0.92020454	0.74405286
New.Immune_Charoentong_Immature__B_cell_CellRep.2017_PMID.28052254	-0.1931896	-0.3298697	Negative	0.92020454	0.74610837
Lobular_Carcinoma_In_Situ_J.Pathol.2017_PMID.27861902	-0.1592692	-0.3286226	Negative	0.92020454	0.74670276
GSEA_GP13_Neural_signailng.r.0.959_MODULE_100	0.20483234	0.32849591	Positive	0.92020454	0.74691148
MM_Red10_BMC.Med.Genomics.2011_PMID.21214954	0.15658129	0.32650094	Positive	0.92020454	0.74835251
TLS_Hallmark_Gene_Signature_PMID.31942071	0.2076754	0.32664856	Positive	0.92020454	0.74849173
MProliferation_BMC.Med.Genomics.2011_PMID.21214954	0.17093046	0.32455988	Positive	0.92035977	0.7497189
FOXC1_Hair_Follicles_Wang_SCIENCE.2016_P30C.LO.vs.WT_Negative_PMID.26	0.18827255	0.32030192	Positive	0.92301825	0.75298858
CTLA4_Single_Gene	-0.1440498	-0.3184331	Negative	0.92352007	0.75450264
C3_TAG_UNTREATED_Usary_Clin.Cancer.Res.2013_PMID.23780888	0.16724946	0.31415843	Positive	0.9250744	0.75745986
MatureLum_HsEnriched_Refined1_BCR.2015_PMID.25575446	-0.0368271	-0.3127803	Negative	0.9250744	0.75878228
HS_Green18_BMC.Med.Genomics.2011_PMID.21214954	0.07527315	0.31124036	Positive	0.9250744	0.75988976
Unknown_7_BMC.Med.Genomics.2011_PMID.21214954	0.1340733	0.31085586	Positive	0.9250744	0.7605094
IMMUNE_Bindea_Cell_TReg_Immunity.2013_PMID.24138885	0.12924749	0.3093389	Positive	0.9250744	0.76130524
REPLICATION_STRESS_POS_Cell.Rep.2018_PMID.29768207	-0.1134394	-0.3037579	Negative	0.92788156	0.76580063
IMMUNE_Bindea_Cell_Th17_cells_Immunity.2013_PMID.24138885	-0.1200153	-0.3036167	Negative	0.92788156	0.76583526
STAT3_Basal_Horvath_SHORT_PNAS.2014_PMID.25139989	-0.1336988	-0.3012595	Negative	0.9288053	0.76770869
MPYMT_NEU_Cluster_BMC.Med.Genomics.2011_PMID.21214954	0.11379041	0.29698473	Positive	0.93071625	0.77040149
GATA3.induced.genes_JCO.2006_PMID.16505416	0.08669769	0.29430312	Positive	0.93167576	0.77231017
HS_Red9_BMC.Med.Genomics.2011_PMID.21214954	-0.0948644	-0.2902496	Negative	0.93321968	0.77573223
Luminal_Progenitor_Up_Nat.Med.2009_PMID.19648928	0.11181147	0.28984903	Positive	0.93321968	0.77582258
CIBERSORT_T_cells_follicular_helper_Nat.Methods.2015_PMID.25822800	-0.1854052	-0.2865589	Negative	0.93473184	0.77833905
HS_Red13_BMC.Med.Genomics.2011_PMID.21214954	0.11540289	0.28515113	Positive	0.93473184	0.7793159
Spike2012_aMaSC_PMID.25575446	0.10403756	0.28220127	Positive	0.93537904	0.7819426
T_Cell_cluster_Iglesia_CCR.2014_PMID.24916698	-0.1624771	-0.2800359	Negative	0.93537904	0.78319721
Fibrinogen_Cluster_BMC.Med.Genomics.2011_PMID.21214954	0.07504565	0.28075296	Positive	0.93537904	0.78321211
Tumour_hypoxia_causes_DNA_hypermethylation_by_reducing_TET_activity_Na	-0.0947152	-0.2776268	Negative	0.93600291	0.78485412
HS_Green13_BMC.Med.Genomics.2011_PMID.21214954	0.10794983	0.27309666	Positive	0.93945823	0.78887521
HISTONE_BMC.Med.Genomics.2011_PMID.21214954	-0.1496685	-0.2707125	Negative	0.93954666	0.79007333
MM_WAPTag_1pFDR_UP_Genome.Biology.2007_PMID.17493263	0.13404077	0.26759871	Positive	0.93978063	0.79242714
IMMUNE_PEREZ_14_JCO.2015_PMID.2560586	-0.1503341	-0.2668321	Negative	0.93978063	0.79318733
CIBERSORT_T_cells_CD4_naive_Nat.Methods.2015_PMID.25822800	-0.1595708	-0.2633461	Negative	0.93978063	0.79579551
New.Immune_ImmLandscape_IFNG_5.PMID.24516633	-0.1364391	-0.2630348	Negative	0.93978063	0.79621009
Endothelial_Normal_EC_high_Angiogenesis_2014_PMID.24257808	0.15550923	0.26065501	Positive	0.93978063	0.79768353
New.Immune_Charoentong_CD56dim_natural_killer_cell_CellRep.2017_PMID.2	-0.0830699	-0.2611175	Negative	0.93978063	0.79795093
IMMUNE_Bindea_Cell_DC_Immunity.2013_PMID.24138885	-0.1485692	-0.2600539	Negative	0.93978063	0.79813905
MM_Squamous.like_Genome.Biol.2013_PMID.24220145	-0.0915019	-0.2562941	Negative	0.94005956	0.80142859
MM_Myc.2012_Genome.Biol.2013_PMID.24220145	-0.1119693	-0.2548412	Negative	0.94005956	0.80233333
ACTIVATED_CANCER_CELL_SIGNATURE_Nat.Cell.Biol.2019_PMID.31263265	-0.1275046	-0.2523159	Negative	0.94005956	0.80418416
MM_Neu.2012_Genome.Biol.2013_PMID.24220145	0.09223662	0.25128884	Positive	0.94005956	0.80478966
New.Immune_TCGA.BRCA.1198_immune_CD8_GZMK_Cell.2015_PMID.264514	-0.1486235	-0.25101	Negative	0.94005956	0.80512278
TCGA.BRCA.1198_immune_CD8_GZMK_JCI.2020_PMID.32573490	-0.1486235	-0.25101	Negative	0.94005956	0.80512278
IMMUNE_Bindea_Cell_T_cells_Immunity.2013_PMID.24138885	-0.1552403	-0.2489947	Negative	0.94045914	0.80658996
Troester_Wound_Signature_Clin.Cancer.Res.2009_PMID.19887484	-0.1192911	-0.2421199	Negative	0.94106443	0.81203253
MUnknown_16_BMC.Med.Genomics.2011_PMID.21214954	-0.107648	-0.2407021	Negative	0.94106443	0.8131982
CIBERSORT_Plasma_cells_Nat.Methods.2015_PMID.25822800	-0.101572	-0.2394346	Negative	0.94106443	0.81431878
CORE_B_CELL_signatures_Garber_Cell.Mol.Gastroenterol.Hepatol.2017_PMID.2	-0.1099526	-0.2390159	Negative	0.94106443	0.81454692
aMaSC_HsEnriched_BCR.2015_PMID.25575446	-0.1034515	-0.235124	Negative	0.94106443	0.81709526
Lim2010_LumProg_Adam_PMID.25575446	0.13010359	0.23176634	Positive	0.94106443	0.81975523
Duke_Module15_p63_Mike_PMID.20335537	0.11374425	0.23156741	Positive	0.94106443	0.82011598

Unknown_11_BMC.Med.Genomics.2011_PMID.21214954	0.11521247	0.23019895	Positive	0.94106443	0.82097854
MIR_200c_Induced_ONCO.2015_PMID.25746005	0.11965071	0.2291217	Positive	0.94106443	0.82176852
LumProg_HsEnriched_BCR.2015_PMID.25575446	-0.0913123	-0.2274935	Negative	0.94106443	0.82292153
CD8_cluster_Iglesia_CCR.2014_PMID.24916698	-0.137271	-0.2275731	Negative	0.94106443	0.82294931
fMaSC_Metab8_CellRep.2018_PMID.30089273	-0.0740496	-0.2273734	Negative	0.94106443	0.82301326
Shehata2012_Erbb3neg_PMID.25575446	0.11946478	0.22683358	Positive	0.94106443	0.82379847
Duke_Module08_her2_Mike_PMID.20335537	-0.0924883	-0.2262835	Negative	0.94106443	0.82390203
CIBERSORT_T_cells_CD8_Nat.Methods.2015_PMID.25822800	-0.1322714	-0.2237019	Negative	0.94106443	0.8259539
High_In_No_Response_MCP_TLS_Melanoma_PMID.31942075	0.14849719	0.22261526	Positive	0.94106443	0.82673735
GSEA_BIOCARTA_BRCA_ATR_PATHWAY_ATRBRCA	0.12669321	0.22187738	Positive	0.94106443	0.82730397
Pcorr_CSR_Activated_Correlation_PNAS.2005_PMID.15701700	0.10947554	0.22087602	Positive	0.94106443	0.82798293
Pcorr_Breast2Lung_Parental_Correlation_Nature.2005_PMID.16049480	-0.101385	-0.2205408	Negative	0.94106443	0.82849691
MHC.I_Breast.Cancer.Research.2008_PMID.19272155	-0.0980251	-0.2178928	Negative	0.94142765	0.83068347
MUnknown_32_BMC.Med.Genomics.2011_PMID.21214954	0.1021186	0.21706306	Positive	0.94142765	0.83106891
MUnknown_27_BMC.Med.Genomics.2011_PMID.21214954	-0.1013278	-0.2140735	Negative	0.94170951	0.83328906
REPLICATION_STRESS_UP_SET_Cell.Rep.2018_PMID.29768207	-0.0788082	-0.2133525	Negative	0.94170951	0.83413863
Lums_HER2E_DOWN_metastatic.signature_JCI.2020_PMID.32573490	-0.1117169	-0.2121135	Negative	0.94170951	0.83469707
CIBERSORT_T_cells_CD4_memory_resting_Nat.Methods.2015_PMID.25822800	-0.1296409	-0.208134	Negative	0.9437987	0.83784642
HS_Red15_BMC.Med.Genomics.2011_PMID.21214954	0.0772093	0.20736956	Positive	0.9437987	0.83880674
Duke_Module03_betacatenin_Mike_PMID.20335537	-0.09896	-0.2045906	Negative	0.94440121	0.84047189
Durvalumab_signature_Higgs_Clin.Cancer.Res.2018_PMID.29716923	-0.0558791	-0.1964955	Negative	0.94875648	0.84721151
Stingl_Day7_Upregulated_Nat.Cell.Biol.2014_PMID.25173976	0.09295	0.1897942	Positive	0.94875648	0.85185734
MNOtch4_BMC.Med.Genomics.2011_PMID.21214954	0.07560011	0.18895403	Positive	0.94875648	0.8526451
Duke_Module10_ifna_Mike_PMID.20335537	0.07827162	0.18797504	Positive	0.94875648	0.85364731
MM_Green2_BMC.Med.Genomics.2011_PMID.21214954	0.09362198	0.186825	Positive	0.94875648	0.85432296
New.Immune_Charoentong_Activated_CD4_T_cell_CellRep.2017_PMID.280522	0.09486069	0.18618026	Positive	0.94875648	0.85475144
Taube_EMT_down_PNAS.2010_PMID.20713713	-0.075628	-0.1860151	Negative	0.94875648	0.85480339
FOXC1_Hair_Follicles_Wang_SCIENCE.2016_P30C.LO.vs.WT_Positive_PMID.269	0.09107709	0.18610013	Positive	0.94875648	0.85498667
HER2_AMPLIFIED_PerouLab_HER2_Amplicon_BMC_Med_Genomics_2011_PMI	0.03945029	0.18504307	Positive	0.94875648	0.85563314
MDACC_P53_ER.Pos_CCR.2011_PMID.21248301	0.09492031	0.18481477	Positive	0.94875648	0.85569664
HS_Green24_BMC.Med.Genomics.2011_PMID.21214954	0.06442158	0.18355641	Positive	0.94893838	0.85699579
MUnknown_29_BMC.Med.Genomics.2011_PMID.21214954	-0.0818646	-0.1789753	Negative	0.95123067	0.86020381
Shehata2012_Basal_PMID.25575446	-0.0803615	-0.1769819	Negative	0.95167459	0.86174362
LumProg_Lim09_BCR.2015_PMID.25575446	-0.0704933	-0.1755772	Negative	0.95172698	0.86292949
HS_Red18_BMC.Med.Genomics.2011_PMID.21214954	0.06224203	0.17437672	Positive	0.95176259	0.86412874
MM_Green16_BMC.Med.Genomics.2011_PMID.21214954	0.07151923	0.17275245	Positive	0.95176259	0.86523872
CIBERSORT_Macrophages_M1_Nat.Methods.2015_PMID.25822800	-0.0812933	-0.1706888	Negative	0.95237517	0.86693482
CIN70_Nat.Genet.2006_PMID.16921376	0.084604	0.16746133	Positive	0.9533439	0.86910536
Lim2009_LumProg_Adam_PMID.25575446	0.06006308	0.16617968	Positive	0.9533439	0.87009737
Histological_Grade_J.Pathol.2017_PMID.27861902	0.08301862	0.16157931	Positive	0.95376534	0.87365991
New.Immune_TCGA.BRCA.1198_immune_CTLA4_CXCL_FOXP3_Cell.2015_PMIL	0.06473531	0.16167763	Positive	0.95376534	0.8739046
TCGA.BRCA.1198_immune_CTLA4_CXCL_FOXP3_JCI.2020_PMID.32573490	0.06473531	0.16167763	Positive	0.95376534	0.8739046
MatureLum_Lim09_BCR.2015_PMID.25575446	0.03278409	0.15808102	Positive	0.95464147	0.87664959
CIBERSORT_T_cells_CD4_memory_activated_Nat.Methods.2015_PMID.258228	-0.0871573	-0.1574664	Negative	0.95464147	0.87699121
MM_Red5_BMC.Med.Genomics.2011_PMID.21214954	-0.0716806	-0.1502008	Negative	0.95942337	0.88253178
HS_Red19_BMC.Med.Genomics.2011_PMID.21214954	-0.0439352	-0.143332	Negative	0.96391625	0.8878176
MDSC_Neutrophil_Youn_J_Leukoc_Biol.2012_PMID.21954284	-0.0490204	-0.140173	Negative	0.96556437	0.89049058
MUnknown_1_BMC.Med.Genomics.2011_PMID.21214954	0.05610512	0.13474139	Positive	0.96883888	0.8946694
X19p13_Amplicon_BMC.Med.Genomics.2011_PMID.21214954	-0.0677337	-0.1310422	Negative	0.96900533	0.89749434
CD8_T_cells_MCP_.31942075_PMID.31942077	0.04838371	0.13036216	Positive	0.96900533	0.89819855
MM_Stat1_Genome.Biol.2013_PMID.24220145	-0.0542826	-0.1280668	Negative	0.96900533	0.89969199
B_Cell_cluster_Iglesia_CCR.2014_PMID.24916698	0.05295751	0.12753587	Positive	0.96900533	0.90025533
Duke_Module11_ifng_Mike_PMID.20335537	0.05564188	0.12579392	Positive	0.96900533	0.90170696
GSEA_GP13_Neural_signailng.r.0.894_NERVOUS_SYSTEM_DEVELOPMENT	-0.0633877	-0.1228717	Negative	0.96900533	0.90373886
IntClust_Amplification.50_Genome.Biol.2014_PMID.25164602	-0.0592759	-0.1185922	Negative	0.96900533	0.9072442
Necrosis_J.Pathol.2017_PMID.27861902	0.06338187	0.11707764	Positive	0.96900533	0.90843481
MICRORNA_138_Signature_Cancer.Res.2014_PMID.25339353	-0.0498369	-0.1166751	Negative	0.96900533	0.90862837
New.Immune_ImmLandscape_Wound_Healing.PMID.14737219	-0.0558492	-0.1158201	Negative	0.96900533	0.90923632
X17q25x_BMC.Med.Genomics.2011_PMID.21214954	-0.0427817	-0.1158672	Negative	0.96900533	0.90948225
BPMS_7.Genes_PLoS.One.2013_PMID.24349199	0.05159562	0.11546047	Positive	0.96900533	0.90956848
IGG_Cluster_BMC.Med.Genomics.2011_PMID.21214954	0.0539702	0.11227097	Positive	0.96900533	0.91214604
T.cells_MCP_PMID.31942075_PMID.31942077	-0.0673955	-0.1120841	Negative	0.96900533	0.91220814
fMaSC_Metab_CellRep.2018_PMID.30089273	0.05165954	0.11200886	Positive	0.96900533	0.91220956
HS_Red1_BMC.Med.Genomics.2011_PMID.21214954	-0.0451185	-0.1066691	Negative	0.97092666	0.91640342
HS_Green5_BMC.Med.Genomics.2011_PMID.21214954	0.05085936	0.10560011	Positive	0.97092666	0.91724631
C3_TAG_RESPONDING_Usary_Clin.Cancer.Res.2013_PMID.23780888	0.04234308	0.10410159	Positive	0.97092666	0.9183825
Unknown_16_BMC.Med.Genomics.2011_PMID.21214954	-0.0571863	-0.1038508	Negative	0.97092666	0.91875653
MM_Wnt1.Early_Genome.Biol.2013_PMID.24220145	0.05141038	0.10180383	Positive	0.97092666	0.92030979
Nuclear_Pleomorphism_J.Pathol.2017_PMID.27861902	0.04642402	0.10064016	Positive	0.97092666	0.92108643
Luminal_Progenitor_Down_Nat.Med.2009_PMID.19648928	-0.0451934	-0.0993177	Negative	0.97092666	0.92214805
MYC_Single_Gene	0.03600399	0.09715764	Positive	0.97159781	0.92394768
MM_Green6_BMC.Med.Genomics.2011_PMID.21214954	0.04401846	0.09108241	Positive	0.97319356	0.92855768
New.Immune_TCGA.BRCA.1198_immune_HLA_A_F_Cell.2015_PMID.26451490	-0.0381665	-0.0907949	Negative	0.97319356	0.92895749
TCGA.BRCA.1198_immune_HLA_A_F_JCI.2020_PMID.32573490	-0.0381665	-0.0907949	Negative	0.97319356	0.92895749
New.Immune_ImmLandscape_lymphocyte_Infil_T.B.PMID.18592372	-0.0470938	-0.086944	Negative	0.97381459	0.93187999
TCGA_LIexpression_score_Immunity.2018_PMID._29628290	-0.0470938	-0.086944	Negative	0.97381459	0.93187999
MUnknown_8_BMC.Med.Genomics.2011_PMID.21214954	-0.0516689	-0.0849997	Negative	0.97415182	0.93336796
HS_Red6_BMC.Med.Genomics.2011_PMID.21214954	-0.0395285	-0.0797576	Negative	0.97500085	0.93741909
HS_Green12_BMC.Med.Genomics.2011_PMID.21214954	-0.0282663	-0.0790242	Negative	0.97500085	0.93813128
CD19_Single_Gene	0.03576007	0.07880231	Positive	0.97500085	0.93824889
Up_regulated_upon_N_RAS_repression_Cell_Rep.2015_PMID.26166574	0.03441728	0.07793445	Positive	0.97500085	0.93884651
HER1_Cluster1_BMC.Genomics.2007_PMID.17663798	0.02489385	0.06434561	Positive	0.98483301	0.94949211
GSEA_GP1_Proliferation_DNA_repair.r.0.972_REACTOME_CELL_CYCLE_MITOTI	0.03322248	0.0620044	Positive	0.98533321	0.95134581
New.Immune_Tertiary.Lymphoid.Structure.12chemokine_FrontImmunol.2017.F	-0.0321263	-0.060838	Negative	0.98533321	0.95233162
HS_Red12_BMC.Med.Genomics.2011_PMID.21214954	0.02239663	0.05461176	Positive	0.9891015	0.95715683
HRAS_Single_Gene	0.01734044	0.04631056	Positive	0.99461744	0.96368436
Epithelial_Tubule_Formation_J.Pathol.2017_PMID.27861902	-0.0142388	-0.0407613	Negative	0.99514136	0.96805389
MM_Green22_BMC.Med.Genomics.2011_PMID.21214954	0.0152165	0.03997766	Positive	0.99514136	0.96865212
TCGA.BRCA.1198_HOXC11_HOTAIR_SIX1_JCI.2020_PMID.32573490	0.02120738	0.0396717	Positive	0.99514136	0.96884567
REPLICATION_STRESS_DOWN_SET_Cell.Rep.2018_PMID.29768207	0.01351062	0.0395344	Positive	0.99514136	0.96895343
GSEA_CASPASE_CASCADE_APOPTOSIS_SA	-0.0196384	-0.0374595	Negative	0.99559645	0.97061032

MM_C3Tag_1pFDR_UP_Genome.Biology.2007_PMID.17493263	-0.0143651	-0.0331434	Negative	0.99559645	0.97399004
New.Immune_TCGA.BRCA.1198_IMMUNE1_Cell.2015_PMID.26451490	-0.0158551	-0.0284115	Negative	0.99559645	0.97771513
TCGA.BRCA.1198_IMMUNE1_JCI.2020_PMID.32573490	-0.0158551	-0.0284115	Negative	0.99559645	0.97771513
CD103_Positive_Cancer.Cell.2014_PMID.25446897	-0.0180402	-0.026699	Negative	0.99559645	0.97904974
New.Immune_Charoentong_Memory_B_cell_CellRep.2017_PMID.28052254	-0.0181677	-0.0259439	Negative	0.99559645	0.97962292
Response_to_PARP_inhibitor_olaparib_Resistance_BC_Res_Treat_2012_PMID.2	-0.0127982	-0.0246593	Negative	0.99559645	0.98067124
Scorr_S329_L_Correlation_Br.J.Cancer.2008_PMID.18382427	0.01093067	0.02417162	Positive	0.99559645	0.98104336
LumProg_Prat_BCR.2015_PMID.25575446	-0.0096508	-0.0240439	Negative	0.99559645	0.9811149
Wahl_aMaSC_Signature_Cell.Stem.Cell.2012_PMID.22305568	-0.0080978	-0.0238722	Negative	0.99559645	0.98130559
Lim2010_MaSC_Adam_PMID.25575446	0.00901937	0.02150192	Positive	0.99586328	0.9831424
MDSC_Granulocytic_Youn_J_Leukoc_Biol.2012_PMID.21954284	-0.0100016	-0.020465	Negative	0.99586328	0.98395104
Mitotic_Count_J.Pathol.2017_PMID.27861902	0.00985054	0.01748627	Positive	0.99689901	0.98626489
B_lineage_MCP_PMID.31942075_PMID.31942077	0.00672451	0.015847	Positive	0.99689901	0.98756845
Unknown_8_BMC.Med.Genomics.2011_PMID.21214954	0.00497176	0.01414298	Positive	0.99689901	0.98891459
REPLICATION_STRESS_NEG_Cell.Rep.2018_PMID.29768207	-0.0043666	-0.0120146	Negative	0.99689901	0.99056254
Pfefferle2012_LumProg_PMID.25575446	-0.0039146	-0.0109601	Negative	0.99689901	0.99139075
MIR_200c_Repressed_ONCO.2015_PMID.25746005	0.0041205	0.00806789	Positive	0.99689901	0.99366905
Tcell_EXH_Anti_PDL1_vs_control_treated_exhausted_CD8_T_cell_Metagene_1	-0.0030584	-0.007814	Negative	0.99689901	0.99387916
MUnknown_7_BMC.Med.Genomics.2011_PMID.21214954	-0.0030386	-0.0069891	Negative	0.99689901	0.99451409
HER1_Cluster2_BMC.Genomics.2007_PMID.17663798	-0.0003988	-0.0008904	Negative	0.99953308	0.9993008
Immune_High_In_Response_MCP_TLS_Melanoma_PMID.31942075	0.00028276	0.00059551	Positive	0.99953308	0.99953308

Supplementary Data 5

Linear mixed model - DNA - All samples

Segment	Intercept	Coef	Std.err	df	T.value	P.value	P.adj
chr5:139008130-142795270.Basal.5q31.3-50.del	0.48868684	-0.80472307	0.10779302	14.0089281	-7.46544668	3.02E-06	0.00161397
chr5:143171919-147191453.Basal.5q32-51.del	0.25175	-0.57231963	0.09029249	14.0014212	-6.33850737	1.83E-05	0.00371006
chr5:147238467-151765017.Basal.5q33.1-52.del	0.28866228	-0.57914351	0.09251874	14.0116831	-6.25974307	2.08E-05	0.00371006
chr5:176660805-180009206.Basal.5q35.3-58.del	0.48575	-0.60599745	0.11842017	14.0581919	-5.11735007	1.55E-04	0.020638
chr5:135493104-138988200.Basal.5q31.2-49.del	0.425	-0.68635721	0.13741794	13.9881131	-4.99466971	1.97E-04	0.02103389
chr5:174477192-180857866.BeroukhimS2.5q35.3.amp	0.45913043	-0.58729011	0.12818781	14.0722087	-4.58148158	4.22E-04	0.03754105
5.q.wholearm.chr5:47700000-180857866	0.17090141	-0.46574901	0.10442789	14.0310889	-4.46000602	5.36E-04	0.04089957
chr5:152850499-154326721.Basal.5q33.2-53.del	0.48575	-0.57098948	0.13420056	14.0767079	-4.25474732	7.91E-04	0.05269814
chr5:177541057-180857866.BeroukhimS2.5q35.3.del	0.43009091	-0.53669458	0.12788529	14.061855	-4.19668749	8.88E-04	0.05269814
chr4:37121738-38804810.Basal.4p14-12.del	0.4655	-0.89490497	0.21856506	14.143194	-4.09445576	0.00107238	0.05726513
4.p.wholearm.chr4:1-50700000	0.22413732	-0.60133952	0.1503568	14.2795707	-3.99941692	0.00127023	0.06166391
chr4:5577784-10295484.Basal.4p16.1-6.del	0.07761765	-0.62196969	0.1590124	14.1452521	-3.91145396	0.00153786	0.06380501
chr4:41057559-42353879.Basal.4p13-13.del	0.4655	-0.86678562	0.2254697	14.1456404	-3.84435529	0.00175632	0.06380501
chr4:13238819-37565122.BeroukhimS5.del	0.16960417	-0.67525289	0.17568843	14.0600369	-3.84346812	0.00177754	0.06380501
chr4:19864333-22430289.Basal.4p15.31-9.del	0.12891667	-0.66449494	0.17700848	14.035473	-3.7540288	0.00212766	0.06380501
chr4:10979549-15266111.Basal.4p15.33-7.del	0.2465	-0.66825219	0.17918834	14.1024419	-3.72932865	0.00221763	0.06380501
chr4:15313739-17632477.Basal.4p15.32-8.del	0.2465	-0.66825219	0.17918834	14.1024419	-3.72932865	0.00221763	0.06380501
chr4:3220565-5553626.Basal.4p16.2-5.del	0.0485	-0.62255517	0.16760834	14.1598053	-3.71434486	0.00227022	0.06380501
chr4:4349500-4427489.BeroukhimS5.amp	0.0485	-0.62255517	0.16760834	14.1598053	-3.71434486	0.00227022	0.06380501
chr4:30331135-30753569.Basal.4p15.1-11.del	0.029	-0.65234209	0.17724131	13.9966759	-3.68053077	0.00247167	0.06599354
chr4:23402742-26636101.Basal.4p15.2-10.del	0.01875	-0.64862206	0.17798777	13.9949084	-3.64419455	0.00265668	0.06755566
chr1:174611641-175371076.BeroukhimS5.amp	0.909	0.43829025	0.14043958	13.9983893	3.12084563	0.00751554	0.18242272
1.q.wholearm.chr1:124300000-247249719	0.8579105	0.48324211	0.15839695	13.9963276	3.05082964	0.00863775	0.20054602
chr1:162199047-163197517.BeroukhimS5.amp	0.909	0.39978978	0.13960032	13.998276	2.86381719	0.01250562	0.27825006
chr5:167651670-171366482.Basal.5q35.1-56.del	0.48575	-0.65877376	0.23545785	14.161167	-2.79784162	0.01411636	0.28992835
chr5:172591744-174888201.Basal.5q35.2-57.del	0.48575	-0.65877376	0.23545785	14.161167	-2.79784162	0.01411636	0.28992835
chr1:163619952-164752515.BeroukhimS5.amp	0.909	0.39487962	0.14379908	13.998177	2.74605109	0.01576825	0.29402502
chr1:144078052-144943150.BeroukhimS5.amp	0.73154167	0.52470953	0.19307556	13.9960823	2.71763822	0.01667381	0.29402502
chr1:158317017-159953843.BeroukhimS2.1q23.3.amp	0.89472	0.46946596	0.17288936	13.9971867	2.71541274	0.01674575	0.29402502
chr1:148661965-149063439.BeroukhimS2.1q21.2.amp.MCL1	0.888	0.50717029	0.18759531	13.9966982	2.70353398	0.01714046	0.29402502
chr14:23609961-31698685.Basal.14q12-88.del	0.46258962	-0.72509403	0.27488866	17	-2.63777347	0.01727376	0.29402502
chr14:28763785-29367842.BeroukhimS5.amp	0.51225	-0.77438333	0.29464144	17	-2.62822272	0.01761948	0.29402502
chr1:151026302-152973244.BeroukhimS5.amp	0.88825	0.49574925	0.1867924	13.9967279	2.65401184	0.01888358	0.30471896
chr4:91089383-93486891.BeroukhimS2.4q22.1.del	0.48325	-0.52593889	0.20718572	17	-2.53849009	0.0212059	0.30471896
chr5:85837489-133480433.BeroukhimS2.5q21.1.del.APC	-0.02046875	-0.39372617	0.15196028	13.9581908	-2.59098082	0.02138878	0.30471896
chr14:92773649-95250286.Basal.14q32.13-99.del	0.00976429	-0.41407621	0.1604616	13.9901416	-2.58053152	0.02179639	0.30471896
chr20:1-325978.BeroukhimS2.2p13.del	0.46075	-0.52539262	0.20383922	13.947322	-2.57748539	0.0219674	0.30471896
chr5:159922707-167623739.Basal.5q34-55.del	0.48575	-0.53145763	0.20666752	14.1743909	-2.57155852	0.02200275	0.30471896
chr5:130522776-135427403.Basal.5q31.1-48.del	0.16739655	-0.53926791	0.20965343	13.8860287	-2.57218744	0.02225476	0.30471896
chr11:42387077-47453403.BeroukhimS5.amp	-0.08025	-0.26698862	0.10447012	14.0223765	-2.55564576	0.02284357	0.30496165
chr14:1-29140968.BeroukhimS2.14q11.2.del	0.47541797	-0.68233672	0.27865341	17	-2.4486932	0.02548082	0.31045624
chr5:155686345-159845035.Basal.5q33.3-54.del	0.48575	-0.50722781	0.2033102	14.1612794	-2.49484689	0.02555576	0.31045624
chr14:21961377-23608756.Basal.14q11.2-87.del	0.37923125	-0.64853792	0.265259	17	-2.4449233	0.02567698	0.31045624
chr5:121675719-127022221.Basal.5q23.2-46.del	0.08201389	-0.47029338	0.19060511	13.9307427	-2.46737025	0.02719555	0.31045624
chr14:92049878-92765306.Basal.14q32.12-98.del	0.26575	-0.70310621	0.28536855	13.9864646	-2.46385317	0.0273203	0.31045624
chr5:127447382-129549383.Basal.5q23.3-47.del	0.148	-0.550842	0.22334187	13.8600918	-2.46636243	0.0273248	0.31045624
chr5:128458341-128477620.LumB.5q23.3-138.amp	0.148	-0.550842	0.22334187	13.8600918	-2.46636243	0.0273248	0.31045624
chr19:44889764-44995371.BeroukhimS5.del	0.85075	1.00008677	0.41381544	13.9982283	2.41674589	0.02989581	0.32465333
chr12:20728852-21227330.BeroukhimS5.del	1.453	-0.60139018	0.25082451	14.0015602	-2.39765321	0.03100622	0.32465333
chr12:25189655-25352305.BeroukhimS2.12p12.1.amp.KRAS	1.453	-0.60139018	0.25082451	14.0015602	-2.39765321	0.03100622	0.32465333
chr12:27342867-29425476.BeroukhimS5.amp	1.453	-0.60139018	0.25082451	14.0015602	-2.39765321	0.03100622	0.32465333
chr4:76623381-79684447.Basal.4q21.1-16.del	0.38900926	-0.45370062	0.19848712	17	-2.2857937	0.03537518	0.3621094
chr14:1-23145193.BeroukhimS2.14q11.2.amp	0.43536875	-0.62013612	0.2673826	14.0573659	-2.3192838	0.0359397	0.3621094
chr5:98132900-102642260.Basal.5q21.1-39.del	-0.09535	-0.3362318	0.1467886	13.9732249	-2.29058521	0.03805537	0.36456398
chr22:37740208-38258806.Basal.22q13.1-113.amp	-0.19375	0.64630563	0.28262727	14.0802043	2.28677731	0.03820046	0.36456398
chr6:63255006-65243766.BeroukhimS2.6q12.amp	-0.0455	0.67565671	0.29574838	14.1360616	2.28456606	0.03829371	0.36456398
chr4:104889590-106814504.BeroukhimS5.del	0.32655	-0.37740333	0.16922684	17	-2.23016237	0.03950397	0.36456398
chr3:116900556-120107320.BeroukhimS2.3q13.31.del	-0.01025	0.34157831	0.15055937	13.9397789	2.26872826	0.03970276	0.36456398
chr5:82803339-90714877.Basal.5q14.3-37.del	-0.10092647	-0.30722192	0.13638378	13.9834541	-2.25262797	0.04087166	0.36456398
chr5:125787000-126200608.LumB.5q23.2-137.amp	0.148	-0.52878075	0.2349755	13.8727219	-2.25036546	0.04118656	0.36456398
chr5:9224578-10673735.BeroukhimS5.amp	-0.222	0.61973528	0.27644483	14.0382091	2.24180454	0.04164495	0.36456398
chr5:115193714-121517156.Basal.5q23.1-45.del	-0.034	-0.38332727	0.17398098	13.9685973	-2.20327118	0.0448652	0.38507376
chr14:31868274-34078694.Basal.14q13.1-89.del	0.04225	-0.33671667	0.15676255	17	-2.14794073	0.04642647	0.38507376
chr4:71492582-75938920.Basal.4q13.3-15.del	0.39175	-0.42841667	0.20047786	17	-2.13697746	0.04742934	0.38507376
chr4:73526461-75252649.BeroukhimS5.amp	0.39175	-0.42841667	0.20047786	17	-2.13697746	0.04742934	0.38507376
chr19:43177306-45393020.BeroukhimS2.19q13.2.amp	0.87692917	0.90884698	0.42429873	13.9997605	2.14199788	0.05026049	0.38507376
chr22:36048950-38681384.BeroukhimS5.amp	-0.02762917	0.61309647	0.28811986	14.0710778	2.12792156	0.05149714	0.38507376
chr14:34100051-35410919.Basal.14q13.2-90.del	0.04225	-0.30606111	0.14747483	17	-2.07534468	0.0534454	0.38507376
chr14:80741860-106368585.BeroukhimS2.14q32.12.del	0.11899548	-0.30363014	0.14474058	14.0652371	-2.09775406	0.05447019	0.38507376
chr1:119996566-120303234.BeroukhimS2.1p12.amp	0.299	1.09633718	0.52333771	14.0297278	2.09489429	0.05480782	0.38507376
chr5:101597589-102642260.LumB.5q21.1-135.amp	-0.034	-0.3636647	0.17571782	13.9663066	-2.06959489	0.05751719	0.38507376
chr12:29425476-31105002.BeroukhimS5.del	1.453	-0.56761875	0.27627616	14.0013364	-2.05453393	0.05908323	0.38507376
chr5:102912456-102926389.Basal.5q21.2-40.del	-0.034	-0.36272581	0.17658403	13.9660603	-2.05412577	0.05917722	0.38507376
chr5:102912456-102926389.LumB.5q21.2-136.amp	-0.034	-0.36272581	0.17658403	13.9660603	-2.05412577	0.05917722	0.38507376
chr5:106744250-109231328.Basal.5q21.3-41.del	-0.034	-0.36272581	0.17658403	13.9660603	-2.05412577	0.05917722	0.38507376
chr5:109877429-111137815.Basal.5q22.1-42.del	-0.034	-0.36272581	0.17658403	13.9660603	-2.05412577	0.05917722	0.38507376
chr5:111507433-112958878.Basal.5q22.2-43.del	-0.034	-0.36272581	0.17658403	13.9660603	-2.05412577	0.05917722	0.38507376
chr5:113725565-115180304.Basal.5q22.3-44.del	-0.034	-0.36272581	0.17658403	13.9660603	-2.05412577	0.05917722	0.38507376
chr14:35708407-36097605.BeroukhimS2.14q13.3.amp.NKX2-	0.04225	-0.29998333	0.14853261	17	-2.01964623	0.05947085	0.38507376
2.q.wholearm.chr2:93300000-242951149	0.05770408	-0.16703453	0.08150048	13.9788453	-2.04949127	0.05966491	0.38507376
chr4:79691766-80079606.Basal.4q21.21-17.del	0.2605	-0.44141988	0.21516167	13.6721458	-2.05157304	0.05987966	0.38507376
chr4:83634873-83961360.BeroukhimS5.del	0.2605	-0.44141988	0.21516167	13.6721458	-2.05157304	0.05987966	0.38507376
chr1:223876038-247249719.BeroukhimS2.1q43.del.RYR2	0.68501698	0.47877026	0.23449416	14.0015608	2.04171511	0.06049024	0.38507376
chr19:39677306-41155075.BeroukhimS5.amp	0.9365	0.86142915	0.42205679	13.9968142	2.04102661	0.0605734	0.38507376
chr2:1-15244284.BeroukhimS2.2p25.3.del	0.26963208	0.33842276	0.17075792	13.9939909	1.98188616	0.06748534	0.42396675
14.q.wholearm.chr14:15600000-106368585	0.08652133	-0.28439217	0.14528385	14.4952625	-1.95749329	0.06982866	0.4262936
chr1:169549478-170484405.BeroukhimS2.1q24.3.amp	0.909	0.36953904	0.18888895	13.9969766	1.95638251	0.07067613	0.4262936
chr12:10732932-10778896.BeroukhimS5.amp	1.1015	-0.48591722	0.2487464	13.9979519	-1.95346435	0.07104893	0.4262936
chr12:11410696-12118386.BeroukhimS2.12p13.2.del.ETV6	1.1015	-0.48591722	0.2487464	13.9979519	-1.95346435	0.07104893	0.4262936
chr10:20145174-20609292.Basal.10p12.32-71.amp	0.58575	0.84386681	0.44225907	14.0134078	1.90808254	0.07708194	0.45553056
chr14:35837522-37090214.Basal.14q13.3-91.del	0.02032143	-0.27805476	0.14850118	17	-1.87240775	0.07845474	0.45553056
chr1:166729757-167532460.BeroukhimS5.amp	0.909	0.36184413	0.19134542	13.9968583	1.89105197	0.07949723	0.45553056
chr1:168438419-168880930.BeroukhimS5.amp	0.909	0.36184413	0.19134542	13.9968583	1.89105197	0.07949723	0.45553056
chr10:7244255-12332593.Basal.10p14-68.amp	0.72225	0.79951867	0.42392948	14.0210014	1.8859709	0.08018703	0.45553056
chr7:137042370-158819393.BeroukhimS5.amp	-0.12645701	0.24703043	0.13231596	14.0298723	1.86697374	0.08294126	0.45618364
chr19:37265855-38702029.BeroukhimS5.amp	0.9365	0.81449416	0.43631841	13.9958526	1.86674259	0.08302685	0.45618364
chr4:54471680-55980061.BeroukhimS2.4q12.amp.PDGFRA.K	0.52825	-0.39773491	0.21315759	13.9682823	-1.86591958	0.08319057	0.45618364
chr19:39664720-39962225.Basal.19q13.11-112.amp	0.9365	0.812711	0.43645294	13.9959099	1.86208163	0.08371909	0.45618364
chr19:34975531-35098303.BeroukhimS2.19q12.amp.CCNE1	0.9365	0.78547617	0.42313	13.9948902	1.85634715	0.08457954	0.4562169
chr10:21110098-22746531.Basal.10p12.31-72.amp	0.58575	0.80631801	0.44095645	14.0107659	1.82856609	0.08882909	0.46965085
chr10:22863772-24876778.Basal.10p12.2-73.amp	0.58575	0.80631801	0.44095645	14.0107659	1.82856609	0.08882909	0.46965085
chr2:53680282-53845245.BeroukhimS5.amp	0.27975	0.27412716	0.150669	13.9947193	1.81939986	0.09030555	0.47277611
chr5:76408288-81082828.Basal.5q14.1-35.del	-0.13370588	-0.25464619	0.14203863	13.9772963	-1.79279537	0.09466715	0.49062133
chr5:1473801-6790134.BeroukhimS5.amp	-0.0768375	0.53749038	0.30077548	14.0299621	1.78701528	0.09555172	0.49062133
chr21:38584860-42033506.BeroukhimS2.21q22.2.del.DSCAM	-0.1487	0.2017659	0.11497665	13.9968546	1.75484245	0.10114037	0.50766865
12.p.wholearm.chr12:1-35400000	1.17253507	-0.40192651	0.22945469	13.9978515	-1.7516596	0.10170177	0.50766865
chr10:101955-12088176.BeroukhimS5.amp	0.67898529	0.7017031	0.40150933	14.0076729	1.74766327	0.10239725	0.50766865
chr21:35022364-36008600.BeroukhimS5.del	-0.16275	0.19568255	0.11206348	14.0001714	1.74617595	0.10267456	0.50766865
chr10:3811260-6662244.Basal.10p15.1-67.amp	0.72225	0.68199497	0.39261041	14.0091745	1.73707817	0.10429869	0.51096788
chr12:30999223-32594050.BeroukhimS2.12p11.21.amp	1.36527273	-0.48364382	0.2817436	14.0009048	-1.7166098	0.1080815	0.52468656
chr10:12431738-17283687.Basal.10p13-69.amp	0.73031897	0.76131508	0.45145536	14.0221257	1.68635737	0.11383914	0.54765855
chr22:31596415-33790650.BeroukhimS5.del	-0.0665	0.5265941	0.31338028	14.0880435	1.68036767	0.11491461	0.54789642
chr3:1-2121282.BeroukhimS2.3p26.3.del	0.41025	0.42622902	0.25855103	13.9590337	1.64852961	0.1215591	0.55166712
chr3:41866-18694914.BeroukhimS5.amp	0.41025	0.4230852	0.25716696	13.9596546	1.64517711	0.12225441	0.55166712
7.q.wholearm.chr7:59100000-158821424	-0.18229966	0.21520853	0.13088544	14.0806045	1.64425118	0.12225755	0.55166712
chr10:17311250-19896508.Basal.10p12.33-70.amp	0.73825	0.74560049	0.45387425	14.0226747	1.64274682	0.12266203	0.55166712
chr7:144118814-148066271.BeroukhimS2.7q35.del.CNTNAP2	-0.078	0.24246682	0.14789723	14.0078678	1.63942768	0.12338052	0.55166712
chr7:83246850-83689170.BeroukhimS5.amp	-0.339	0.27128844	0.16658349	14.1253832	1.62854339	0.12550111	0.55166712
21.p.wholearm.chr21:1-12300000	-0.0525	0.30225289	0.18602097	13.9772528	1.62483236	0.12652659	0.55166712
10.p.wholearm.chr10:1-40300000	0.71562199	0.70395364	0.43449287	14.0123508	1.62017305	0.12747426	0.55166712
chr11:32027116-37799354.BeroukhimS2.11p13.amp	-0.02172857	-0.21823046	0.13579388	14.091656	-1.60707134	0.13020746	0.55166712
21.q.wholearm.chr21:12300000-46944323	-0.06008778	0.17457235	0.10868233	13.9997041	1.60626257	0.13052947	0.55166712
chr12:18393150-19784941.BeroukhimS5.amp	1.1015	-0.43138066	0.26869402	13.9981221	-1.60547175	0.13070638	0.55166712
chrX:139424793-139908341.BeroukhimS5.del	-0.10325	-0.43792032	0.27280005	13.9758732	-1.60527945	0.13078391	0.55166712
chrX:148267454-148753845.BeroukhimS5.amp	-0.10325	-0.43792032	0.27280005	13.9758732	-1.60527945	0.13078391	0.55166712
chr15:31945721-37862331.Basal.15q14-103.del	-0.35745	-0.1580907	0.09862992	14.0034039	-1.60286761	0.13127381	0.55166712
chr2:61834240-63151654.BeroukhimS5.amp	0.167875	0.20484471	0.12820566	13.9926305	1.59778213	0.13242143	0.55166712
chr16:51646446-54466783.LumA.16q12.2-121.del	0.17975	-0.28534825	0.17902598	14.0077771	-1.59389294	0.1332679	0.55166712
chr16:54782803-56638303.LumA.16q13-122.del	0.17975	-0.28534825	0.17902598	14.0077771	-1.59389294	0.1332679	0.55166712
chr10:24604620-28074753.Basal.10p12.1-74.amp	0.68148864	0.72041514	0.45358598	14.009912	1.58826587	0.13453245	0.5526179
chr10:3099740-3205003.Basal.10p15.2-66.amp	0.54525	0.67503235	0.42739941	13.9977087	1.57939467	0.13657152	0.55378519
chr7:129281946-131188950.BeroukhimS5.amp	-0.19125	0.2004871	0.12709662	14.0805869	1.57743855	0.13689072	0.55378519
chrX:152729030-154913754.BeroukhimS2.Xq28.amp	0.07975	-0.42209549	0.26939591	13.9381428	-1.56682216	0.13957264	0.55669247
chr5:92250850-96544700.Basal.5q15-38.del	-0.2715	-0.21400007	0.13664884	13.9738903	-1.56605846	0.13969436	0.55669247
11.p.wholearm.chr11:1-52900000	-0.2190359	-0.20580776	0.13200972	14.1412714	-1.55903488	0.14108212	0.55805816
chr7:87797038-89779004.BeroukhimS5.del	-0.339	0.25561134	0.16761655	14.134437	1.52497673	0.14932499	0.58632019
chr7:92829306-132566935.BeroukhimS5.del	-0.1749973	0.19760462	0.13118481	14.0728444	1.50630717	0.15410258	0.60062916
chr7:86091574-88424037.BeroukhimS5.amp	-0.339	0.31993524	0.21355036	14.164449	1.49817234	0.15604063	0.60062916
chr11:57344931-58658339.BeroukhimS5.del	-0.2875	0.23311044	0.15655322	14.0719074	1.48901725	0.15855019	0.60062916
chr19:7321723-7480918.BeroukhimS5.del	0.0465	-0.21303333	0.1449819	17	-1.46937884	0.15999329	0.60062916
chr7:115981465-116676953.BeroukhimS2.7q31.2.amp.MET	0.328	0.19214136	0.12969131	13.9969696	1.48152837	0.16062522	0.60062916
chr16:11352227-11658331.BeroukhimS5.amp	0.353	-0.26979895	0.18275219	14.1000531	-1.47631029	0.16184284	0.60062916
chr16:56705000-65205296.LumA.16q21-123.del	0.17975	-0.23754463	0.16110284	14.0051075	-1.47449066	0.16247182	0.60062916
chr7:89924533-98997268.BeroukhimS2.7q21.3.amp.CDK6	-0.17139231	0.22838406	0.15518796	14.0502085	1.47166093	0.16315466	0.60062916
2.p.wholearm.chr2:1-93300000	0.05107883	0.17962188	0.12206486	13.9961086	1.47152816	0.16327381	0.60062916
chr7:141592807-142264966.BeroukhimS2.7q34.del.TRB	-0.19875	0.2603158	0.1777889	14.0265572	1.4641848	0.16519374	0.60062916
chr11:57861150-58074632.BeroukhimS5.amp	-0.2875	0.22786716	0.1557114	14.0687287	1.46339421	0.16534174	0.60062916
chr1:241364021-247249719.BeroukhimS2.1q44.amp	0.92186404	0.39899859	0.27409252	13.9975905	1.45570769	0.16753396	0.60448063
chr16:1-359092.BeroukhimS2.16p13.3.del	0.15525	-0.3653188	0.25411899	13.9660719	-1.43758952	0.17257769	0.61849991
chr10:19022250-42889659.BeroukhimS5.del	0.70285667	0.62753874	0.43966563	14.0107984	1.42730908	0.17539743	0.62441486
chr7:156893473-158821424.BeroukhimS2.7q36.3.del.PTPRN2	-0.26175	0.21489488	0.15129746	14.087134	1.42034692	0.17726007	0.62686674
chr10:3811260-5139878.LumB.10p15.1-143.del	0.72225	0.59061983	0.41713694	14.0044545	1.41588952	0.17866289	0.62767093
chr17:18837023-19933105.BeroukhimS2.17p11.2.amp	0.33626667	-0.28392147	0.20145268	14.0314478	-1.40937053	0.18050249	0.6299891
chr10:170643-1769670.Basal.10p15.3-65.amp	0.537	0.62723331	0.44818551	13.9956004	1.39949482	0.18343755	0.63234822
chr10:1-1042949.BeroukhimS2.1p15.3.del	0.537	0.62713879	0.44823711	13.9955968	1.39912288	0.18354677	0.63234822
chr19:10895846-11164554.BeroukhimS5.del	-0.06175	-0.21416862	0.15389951	13.9607263	-1.3916134	0.18581647	0.63606405
chr12:33156845-33480710.BeroukhimS5.amp	1.30375	-0.38612571	0.28103512	13.9998004	-1.37394113	0.19105953	0.64984578
3.q.wholearm.chr3:91700000-199501827	0.32975893	0.19075833	0.14118799	17	1.35109462	0.19437405	0.65693508
chr5:36750649-37111693.BeroukhimS5.amp	0.807	-0.37236349	0.27635427	14.0901034	-1.34741358	0.19911609	0.65990328
chr10:35175415-37212810.BeroukhimS5.amp	0.83575	0.62971822	0.47053715	14.0155241	1.3382965	0.2021073	0.65990328
chr10:31648148-32903498.Basal.10p11.22-76.amp	0.83575	0.62944803	0.47049772	14.0154945	1.3378344	0.20225422	0.65990328
4.q.wholearm.chr4:50700000-191273063	0.37848687	-0.33268245	0.25122055	13.7909482	-1.32426449	0.20694416	0.65990328
chr7:65877239-79629882.BeroukhimS2.7q21.11.del.MAGI2	-0.32694231	0.23774281	0.18179073	14.1742035	1.30778297	0.21176124	0.65990328
1.p.wholearm.chr1:1-124300000	-0.10025359	0.13070851	0.10001797	14.1396621	1.30685025	0.21211935	0.65990328
chr12:85072329-85674601.BeroukhimS2.12q21.32.amp	-0.0995	0.41296667	0.31948027	17	1.29262026	0.21343586	0.65990328
chr7:54899301-55275419.BeroukhimS2.7p11.2.amp.EGFR	-0.3985	0.22377046	0.17209033	14.0431972	1.30030811	0.21443155	0.65990328
chr9:137859478-140273252.BeroukhimS2.9q34.3.amp	0.69203896	-0.38264848	0.29682168	17	-1.28915275	0.21461095	0.65990328
chr2:204533830-206266883.BeroukhimS2.2q33.2.del.PARD3I	0.195	-0.21743414	0.16743528	13.9978369	-1.29861605	0.21506259	0.65990328
chr11:110631916-112776199.LumB.11q23.1-159.del	-0.3635	0.12076902	0.09307569	13.9997573	1.29753564	0.21542085	0.65990328
chr5:45312870-49697231.BeroukhimS2.5p11.amp	0.3565	0.25436668	0.19626202	14.0168588	1.29605656	0.21589088	0.65990328
chr12:76852527-77064746.BeroukhimS2.12q21.2.amp	-0.0995	0.41016667	0.31920884	17	1.28494769	0.21604281	0.65990328
chr6:54352087-55392731.BeroukhimS5.amp	1.0215	0.38697119	0.2996273	14.0089844	1.29150845	0.21743058	0.65990328
chr5:66513872-67633403.Basal.5q13.1-32.del	-0.5155	-0.18520773	0.14342343	14.0040215	-1.29133524	0.21749621	0.65990328
chr5:68425638-71536822.LumB.5q13.2-134.amp	-0.5155	-0.18520773	0.14342343	14.0040215	-1.29133524	0.21749621	0.65990328
chr5:68425638-72913540.Basal.5q13.2-33.del	-0.5155	-0.18520773	0.14342343	14.0040215	-1.29133524	0.21749621	0.65990328
chr5:73958991-76396789.Basal.5q13.3-34.del	-0.5155	-0.18520773	0.14342343	14.0040215	-1.29133524	0.21749621	0.65990328
19.p.wholearm.chr19:1-28500000	0.18893313	-0.19048399	0.14799444	13.9931697	-1.28710233	0.21894236	0.66053798
chr8:128414451-128718816.BeroukhimS5.amp	2.35675	0.75965	0.60011642	17	1.26583772	0.22264448	0.66420198
chr8:128774432-128849112.BeroukhimS2.8q24.21.amp.MYC	2.35675	0.75965	0.60011642	17	1.26583772	0.22264448	0.66420198
chr17:44673157-45060263.BeroukhimS2.17q21.33.amp	0.59325	-0.30568889	0.24126228	14.0362864	-1.26703972	0.22576115	0.66605777

Segments_UP-DOWN_p.adj<0.05	Renamed for figure purposes and simplicity	Intercept	Coef	Std.err	df	T.value	P.value	P.adj
chr5:139008130-142795270.Basal.5q31.3-50.del		0.48868684	-0.80472307	0.10779302	14.0089281	-7.46544668	3.02E-06	0.00161397
chr5:143171919-147191453.Basal.5q32-51.del		0.25175	-0.57231963	0.09029249	14.0014212	-6.33850737	1.83E-05	0.00371006
chr5:147238467-151765017.Basal.5q33.1-52.del		0.28866228	-0.57914351	0.09251874	14.0116831	-6.25974307	2.08E-05	0.00371006
chr5:176660805-180009206.Basal.5q35.3-58.del		0.48575	-0.60599745	0.11842017	14.0581919	-5.11735007	1.55E-04	0.020638
chr5:135493104-138988200.Basal.5q31.2-49.del		0.425	-0.68635721	0.13741794	13.9881131	-4.99466971	1.97E-04	0.02103389
chr5:174477192-180857866.BeroukhimS2.5q35.3.amp	chr5:174477192-180857866_BeroukhimS2.5q35.3	0.45913043	-0.58729011	0.12818781	14.0722087	-4.58148158	4.22E-04	0.03754105
5.q.wholearm.chr5:47700000-180857866	5.q.wholearm.chr5:47700000-180857866	0.17090141	-0.46574901	0.10442789	14.0310889	-4.46000602	5.36E-04	0.04089957

chr17:45731662-46009941.BeroukhimS5.amp	0.59325	-0.30568889	0.24126228	14.0362864	-1.26703972	0.22576115	0.66605777
chr1:41717036-43943775.Basal.1p34.2-1.amp	0.06724219	0.18129112	0.14561867	14.1338733	1.24497169	0.23338941	0.67567143
chr8:38252951-38460772.BeroukhimS2.8p12.amp.FGFR1	0.198	0.52084115	0.42053663	14.0786835	1.23851555	0.23577882	0.67567143
chr5:18240864-21565169.BeroukhimS5.amp	0.533125	0.31905497	0.25785398	14.3215883	1.23734745	0.23586232	0.67567143
5.p.wholearm.chr5:1-47700000	0.30227241	0.32111844	0.25976815	14.1900671	1.23617325	0.23646623	0.67567143
chr10:28141103-31360860.Basal.10p11.23-75.amp	0.8343125	0.58047225	0.47018138	14.0108554	1.23457088	0.23729591	0.67567143
chr11:69588488-69842871.BeroukhimS5.amp	0.38883333	0.22443221	0.18192449	13.9601812	1.23365581	0.23769871	0.67567143
chr7:102988499-103837783.BeroukhimS5.amp	-0.21875	0.19332931	0.15685429	14.1195058	1.23254073	0.23787683	0.67567143
chr17:20027154-20122158.BeroukhimS5.amp	0.06	-0.24563231	0.20129291	13.9570012	-1.22027305	0.24257884	0.68136616
chr20:41621022-45719023.LumB.20q13.12-166.amp	0.29228289	0.25294307	0.20795456	14.0450713	1.21633815	0.24390318	0.68136616
chr4:112023722-118112513.BeroukhimS5.del	0.11275	-0.31774419	0.26264232	13.7262009	-1.20979812	0.24678025	0.68136616
chr4:114219937-114355024.Basal.4q26-18.del	0.11275	-0.32033105	0.26547239	13.7305302	-1.20664544	0.24794757	0.68136616
chr5:43612947-44998256.BeroukhimS5.amp	0.38625	0.23948148	0.19869138	13.9939012	1.20529377	0.24807648	0.68136616
chr17:62318152-63890591.BeroukhimS2.17q24.2.amp	0.17875	0.19696693	0.16377266	13.9801273	1.20268508	0.24907229	0.68136616
chr13:104916365-104941384.Basal.13q33.2-84.amp	-0.643	0.24609236	0.20531442	13.9281229	1.19861214	0.25067578	0.68136616
chr13:106620319-107317084.Basal.13q33.3-85.amp	-0.643	0.24609236	0.20531442	13.9281229	1.19861214	0.25067578	0.68136616
17.p.wholearm.chr17:1-22200000	0.05859394	-0.26791035	0.22448751	13.9378027	-1.19343101	0.25261953	0.68136616
chr14:65275722-67085224.BeroukhimS2.14q23.3.del	0.10385	-0.16250016	0.13618072	13.9662457	-1.19326843	0.25264138	0.68136616
chr5:49730236-50173926.Basal.5q11.1-27.del	0.177	0.17213438	0.14568984	13.9928227	1.1815126	0.25709081	0.68988188
chr2:241477619-242951149.BeroukhimS2.2q37.3.del	0.06205	-0.23120062	0.19666116	14.0216265	-1.17562928	0.25931952	0.69238313
chr22:15272143-16594738.BeroukhimS5.del	0.2185	-0.18520324	0.15797008	13.9858819	-1.17239445	0.26062221	0.69239931
chr17:10675416-12635879.BeroukhimS2.17p12.del.MAP2K4	-0.00175	-0.24185156	0.20956454	13.947818	-1.15406719	0.26786456	0.69755823
16.q.wholearm.chr16:38200000-88827254	0.17736035	-0.18821006	0.16334709	14.0074205	-1.15220945	0.26852096	0.69755823
chr14:69312342-69568836.Basal.14q24.2-97.del	-0.17625	-0.14730674	0.12831482	13.8363548	-1.14801031	0.27042617	0.69755823
chr14:77311255-79682157.BeroukhimS5.del	-0.17625	-0.14730674	0.12831482	13.8363548	-1.14801031	0.27042617	0.69755823
chr14:77945724-77992686.BeroukhimS5.amp	-0.17625	-0.14730674	0.12831482	13.8363548	-1.14801031	0.27042617	0.69755823
7.p.wholearm.chr7:1-59100000	-0.08856902	0.17196187	0.15004113	14.0166481	1.14609821	0.27094486	0.69755823
chr17:59852249-60135967.BeroukhimS5.amp	0.154	0.19511997	0.17123313	14.0067936	1.13949895	0.27360778	0.69755823
chr16:76685816-78205652.BeroukhimS2.16q23.1.del.WWOX	0.17975	-0.21746165	0.19139272	14.0159901	-1.13620648	0.27492481	0.69755823
chr16:79132355-80549399.LumA.16q23.2-127.del	0.17975	-0.21746165	0.19139272	14.0159901	-1.13620648	0.27492481	0.69755823
chr17:31895171-33758810.BeroukhimS5.del	0.40475	-0.31612784	0.27937417	13.924028	-1.13155715	0.27693323	0.69755823
chr17:33754231-34162198.BeroukhimS5.amp	0.40475	-0.31612784	0.27937417	13.924028	-1.13155715	0.27693323	0.69755823
chr17:7471230-7717938.BeroukhimS2.17p13.1.del.TP53	-0.00175	-0.26958883	0.23976263	13.918238	-1.1243989	0.27986236	0.70162677
chr1:110339388-119426489.BeroukhimS2.1p13.2.del	-0.24601744	0.15978619	0.14400587	14.0146947	1.10958109	0.28585651	0.7133055
chr22:18689393-20139185.BeroukhimS5.del	0.06929545	-0.14742758	0.13476439	13.9798171	-1.09396542	0.29246194	0.71883286
chr22:45488286-49691432.BeroukhimS2.22q13.33.del	-0.19375	-0.14618651	0.13378219	14.08827	-1.09272023	0.29285035	0.71883286
chr8:22125332-30139123.BeroukhimS2.8p21.2.del	-0.05658088	-0.24809259	0.22763205	13.9615535	-1.08988427	0.29421834	0.71883286
chr1:201678483-203358272.BeroukhimS2.1q32.1.amp.MDM	1.3855	0.50506901	0.46500143	14.1319107	1.08616656	0.29558582	0.71883286
chr17:28881201-29652449.BeroukhimS5.amp	0.40975	-0.27702792	0.25513826	13.958364	-1.08579532	0.29596609	0.71883286
chr1:3756302-6867390.BeroukhimS2.1p36.31.del	-0.22753704	0.21007641	0.19369691	14.2306425	1.08456251	0.29614837	0.71883286
chr6:110391-1254918.BeroukhimS5.del	0.87825	0.34302881	0.31904427	14.0780326	1.07517619	0.30037814	0.71975907
chr6:1543157-2570302.BeroukhimS2.6p25.3.del	0.87825	0.34302881	0.31904427	14.0780326	1.07517619	0.30037814	0.71975907
chr19:53372336-53572212.BeroukhimS5.del	-0.08375	-0.20347715	0.18969348	13.9311263	-1.07266282	0.30165287	0.71975907
chr17:63942109-65847254.BeroukhimS5.amp	0.154	0.16661715	0.15544713	13.9943243	1.07185733	0.30192141	0.71975907
chr22:19172385-19746441.BeroukhimS2.22q11.21.amp.CRKL	0.04871875	-0.14440456	0.13526378	13.9775017	-1.06757745	0.30380358	0.72102717
chr9:130999928-132303780.BeroukhimS5.del	0.60825	-0.30425	0.28983239	17	-1.04974465	0.30853052	0.72163988
chr16:56791457-72871981.BeroukhimS5.del	0.17975	-0.19012199	0.18005379	14.0148251	-1.05591776	0.30886893	0.72163988
chr16:73739922-78192112.LumA.16q23.1-126.del	0.17975	-0.19631413	0.18605608	14.0177169	-1.05513418	0.30921116	0.72163988
20.p.wholearm.chr20:1-27100000	0.12379545	-0.15959272	0.15262665	13.9754321	-1.04564127	0.31347607	0.72163988
chr15:28193434-29022600.Basal.15q13.2-101.del	-0.47675	-0.1166465	0.11171251	13.9940586	-1.04416681	0.31411088	0.72163988
chr12:8398512-8740196.BeroukhimS5.amp	1.10175	-0.33093515	0.31694241	13.994558	-1.04414915	0.31411815	0.72163988
chr20:14210829-15988895.BeroukhimS2.2p12.1.del.MACROD	-0.083	-0.16449187	0.15778807	13.9854175	-1.04248614	0.31487283	0.72163988
chr20:20585029-20740450.BeroukhimS5.amp	-0.083	-0.16449187	0.15778807	13.9854175	-1.04248614	0.31487283	0.72163988
chr15:29020705-31945591.Basal.15q13.3-102.del	-0.4760625	-0.10410978	0.10042536	13.997401	-1.0366881	0.31745969	0.7244593
chr8:101163387-103693879.BeroukhimS2.8q22.3.amp	2.50367187	0.62824063	0.61383279	17	1.02347191	0.32042913	0.7262878
chr11:26341787-27574592.BeroukhimS5.amp	-0.38425	-0.15586001	0.15142971	13.8647261	-1.0292565	0.32098113	0.7262878
chr12:67440273-67566002.BeroukhimS2.12q15.amp.MDM2	0.07875	0.25624472	0.25158991	13.6812625	1.01850161	0.3261134	0.73425562
chr11:112785528-114880322.LumB.11q23.2-160.del	-0.26907813	0.10188146	0.10036042	14.0023536	1.0151558	0.32725251	0.73425562
chr8:108043446-108683856.BeroukhimS5.amp	2.52475	0.61731667	0.61831218	17	0.99838996	0.33209017	0.74199227
chr7:3516940-6019790.BeroukhimS5.amp	-0.0501625	0.2701145	0.27107532	14.0350323	0.99645551	0.3358993	0.74262354
chr14:93528266-103946058.BeroukhimS5.amp	-0.04824719	-0.11297531	0.11354556	14.1824097	-0.99497773	0.33642083	0.74262354
chr1:114889793-115149999.BeroukhimS5.amp	-0.34425	0.13258593	0.13326392	14.102042	0.99491248	0.33654475	0.74262354
chr16:65206155-69392572.LumA.16q22.1-124.del	0.17975	-0.18303998	0.18586021	14.0183298	-0.98482603	0.341403	0.74716887
chr16:69398793-73198518.LumA.16q22.3-125.del	0.17975	-0.18303998	0.18586021	14.0183298	-0.98482603	0.341403	0.74716887
chr5:81303630-82685333.Basal.5q14.2-36.del	-0.02775	-0.20171121	0.20670364	13.9720896	-0.97584744	0.34573396	0.75355892
chr18:13905410-14018535.BeroukhimS5.amp	0.5165	-0.2694278	0.2781084	14.1817125	-0.96878696	0.34888447	0.75604539
chr19:1818912-2004994.BeroukhimS5.del	0.0315	-0.18736667	0.19483919	17	-0.96164772	0.34970639	0.75604539
chr5:1212750-1378766.BeroukhimS2.5p15.33.amp.TERT	0.191	0.38648651	0.40515281	13.9883126	0.95392773	0.35631507	0.76589894
chr19:15641747-15670750.BeroukhimS5.amp	0.1685	-0.19553569	0.20529521	13.9005005	-0.95246106	0.35713265	0.76589894
chr9:122980237-124410900.Basal.9q33.2-64.del	0.60825	-0.27571667	0.29413923	17	-0.93736788	0.36169653	0.77141205
chr19:1-526082.BeroukhimS2.19p13.3.del	0.02958929	-0.16867024	0.18132922	17	-0.93018784	0.3652955	0.77141205
chr2:159800558-163403274.HER2-enriched.2q24.2-116.amp	0.03745833	-0.07910846	0.08464636	14.4435707	-0.93457604	0.36536943	0.77141205
chr21:44316476-46902240.BeroukhimS5.amp	-0.04215909	0.13500232	0.14433482	13.9864609	0.93534132	0.36548174	0.77141205
chr1:39907605-40263248.BeroukhimS2.1p34.2.amp.MYCL1	0.27725	0.14243174	0.15370047	13.9709392	0.92668383	0.36982304	0.77508631
19.q.wholearm.chr19:28500000-63811651	0.54805668	0.24562009	0.26530723	14.1020252	0.92579491	0.37012548	0.77508631
chr9:110838572-127593510.BeroukhimS5.amp	0.60825	-0.24912538	0.27516695	14.8089256	-0.90536084	0.37976856	0.79217348
chr16:31854743-53525739.BeroukhimS2.16q11.2.del	0.11381548	-0.15201157	0.16911296	14.0101493	-0.89887595	0.38390763	0.79769133
chr18:14720419-16909449.BeroukhimS5.amp	0.3555	-0.21647548	0.24249614	14.1719884	-0.8926966	0.38692704	0.79786742
chr20:34179462-45265004.BeroukhimS5.del	0.28007851	0.14522003	0.1633005	13.9885951	0.88928097	0.38888921	0.79786742
chr16:5062786-7709383.BeroukhimS2.16p13.3.del.A2BP1	0.353	-0.18188589	0.20535441	14.1704021	-0.88571697	0.3905532	0.79786742
chr12:64149851-66323376.BeroukhimS5.del	0.16814286	0.22515896	0.25686386	13.9340543	0.87656922	0.39558182	0.79786742
chr8:29285985-30473918.BeroukhimS5.amp	-0.1715	-0.21294689	0.24335971	13.9297708	-0.87502933	0.39639516	0.79786742
12.q.wholearm.chr12:35400000-132349534	-0.07210962	0.11422271	0.13083065	14.0269753	0.87305772	0.39733158	0.79786742
chr8:140458177-146274826.BeroukhimS2.8q24.3.amp	2.13585859	0.53125746	0.60853198	13.8246478	0.87301486	0.39756556	0.79786742
chr16:80588753-82633263.LumA.16q23.3-128.del	0.21330556	-0.16904924	0.19394221	14.0177198	-0.87164748	0.3980843	0.79786742
chr2:15977811-16073001.BeroukhimS2.2p24.3.amp.MYCN	0.3885	0.20322254	0.23344854	13.9890551	0.87052395	0.39870659	0.79786742
chr11:1-1391954.BeroukhimS2.11p15.5.del	-0.52332292	-0.12717926	0.1464831	13.9504143	-0.86821799	0.39996482	0.79786742
chr8:128823745-146264218.BeroukhimS5.del	2.10077254	0.53223603	0.61370522	13.8172353	0.86725029	0.40061578	0.79786742
chr8:116186189-120600761.BeroukhimS2.8q24.11.amp	2.35675	0.53929615	0.62731608	17	0.85968807	0.40192198	0.79786742
chr2:135029078-135167772.BeroukhimS5.amp	0.0525	-0.11836437	0.13752951	14.0421325	-0.86064711	0.4038885	0.7988017
chr19:52031294-53331283.BeroukhimS2.19q13.32.del	-0.0152069	-0.14851246	0.1742753	14.4974817	-0.85217158	0.40797646	0.80117028
chr16:88436931-88827254.BeroukhimS2.16q24.3.del	0.2305	-0.16688094	0.19567678	14.0155049	-0.85283976	0.40808673	0.80117028
chr11:107086196-116175885.BeroukhimS2.11q23.1.del.ATM	-0.25721825	0.07683612	0.0905835	14.0014517	0.84823528	0.41057414	0.80200333
chr9:110661885-116923292.BeroukhimS5.del	0.60825	-0.21964108	0.25981365	14.6271718	-0.84537928	0.41151482	0.80200333
chr10:38685321-38707438.LumB.10p11.21-144.del	0.83575	0.51462967	0.61129632	14.0029409	0.84186614	0.41401005	0.80393225
chr8:133698327-133914560.BeroukhimS5.amp	1.93125	0.53965468	0.65321627	13.9652059	0.82615009	0.42260934	0.80854462
chr2:121187279-122198612.BeroukhimS5.amp	-0.05516667	-0.11518535	0.13990712	14.3062131	-0.82329868	0.42385044	0.80854462
chr21:46690655-46902240.BeroukhimS5.del	-0.24225	0.15454406	0.18824754	14.038487	0.82096192	0.42539001	0.80854462
chr1:46541957-46555032.Basal.1p33-3.amp	-0.2715	0.12463036	0.15218643	13.9974951	0.81893211	0.42654863	0.80854462
chr9:1-708871.BeroukhimS2.9p24.3.del	0.44514286	-0.18031168	0.22026207	14.0064347	-0.81862335	0.42671041	0.80854462
9.q.wholearm.chr9:51800000-140273252	0.62372299	-0.2047802	0.25221297	14.3888693	-0.81193365	0.43005779	0.80854462
chr2:138479322-143365272.BeroukhimS2.2q22.1.del.LRP1B	0.0525	-0.10349163	0.12817699	14.023558	-0.80741197	0.43291133	0.80854462
chr19:47121673-49919582.BeroukhimS5.del	0.18880282	-0.16076973	0.19972489	14.2373089	-0.80495589	0.43408259	0.80854462
chr1:26377344-27532551.BeroukhimS2.1p36.11.del	0.027	0.05556334	0.06981475	14.2500882	0.79586812	0.43916602	0.80854462
chr1:27351829-27460967.BeroukhimS5.amp	0.027	0.05556334	0.06981475	14.2500882	0.79586812	0.43916602	0.80854462
chr11:116803699-119605859.LumB.11q23.3-161.del	0.1095	0.12234634	0.15529656	13.9489489	0.78782387	0.44398193	0.80854462
chr16:80759878-82408573.BeroukhimS2.16q23.3.del.CDH13	0.17975	-0.15924566	0.20216582	14.0200357	-0.78769823	0.44398728	0.80854462
chr20:40134806-41603948.LumB.20q13.11-165.amp	0.269	0.17932624	0.22855166	14.011211	0.78462018	0.4457403	0.80854462
chr19:63402921-63811651.BeroukhimS2.19q13.43.del	0.99576136	-0.2369193	0.30331739	14.0058955	-0.7810937	0.44774958	0.80854462
chr9:235706-8362053.BeroukhimS5.amp	0.631	-0.24126261	0.31244189	14.0070057	-0.77218394	0.45283802	0.80854462
chr9:831690-1047552.Basal.9p24.3-61.amp	0.631	-0.24126261	0.31244189	14.0070057	-0.77218394	0.45283802	0.80854462
chr9:7161607-12713130.BeroukhimS2.9p24.1.del.PTPRD	0.631	-0.24126261	0.31244189	14.0070057	-0.77218394	0.45283802	0.80854462
chr9:12683435-13935586.Basal.9p23-62.amp	0.631	-0.24126261	0.31244189	14.0070057	-0.77218394	0.45283802	0.80854462
chr9:14071847-14900353.Basal.9p22.3-63.amp	0.631	-0.24126261	0.31244189	14.0070057	-0.77218394	0.45283802	0.80854462
8.q.wholearm.chr8:45200000-146274826	2.03492629	0.48801821	0.63199464	13.879411	0.77218725	0.45295222	0.80854462
chr5:63838208-66501179.Basal.5q12.3-31.del	-0.5155	-0.12612315	0.16344072	14.0042978	-0.77167516	0.45313218	0.80854462
chr2:154436672-155018734.HER2-enriched.2q23.3-114.amp	-0.13275	-0.06003832	0.0789864	13.9974472	-0.76010961	0.45980122	0.80854462
chr2:156889194-159797412.HER2-enriched.2q24.1-115.amp	-0.13275	-0.06003832	0.0789864	13.9974472	-0.76010961	0.45980122	0.80854462
chr7:3046420-4279470.BeroukhimS2.7p22.2.del.SDK1	0.19925	0.21831037	0.28723411	14.0054486	0.76004333	0.45983248	0.80854462
11.q.wholearm.chr11:52900000-134452384	0.01428905	0.07220363	0.09520158	14.0940715	0.75842893	0.46068957	0.80854462
chr6:1-23628840.BeroukhimS2.6p24.1.amp	0.8699925	0.23400475	0.30898082	14.0741333	0.75734396	0.4613361	0.80854462
chr2:164158152-168812365.HER2-enriched.2q24.3-117.amp	0.10918333	-0.06863148	0.09082188	13.9587881	-0.75567122	0.46240841	0.80854462
chr9:36365710-37139941.BeroukhimS2.9p13.2.del.PAX5	0.34175	-0.19844305	0.26270767	13.9852316	-0.75537592	0.46255652	0.80854462
chr9:36988416-36998984.BeroukhimS5.amp	0.34175	-0.19844305	0.26270767	13.9852316	-0.75537592	0.46255652	0.80854462
chr9:37861914-38370450.BeroukhimS5.del	0.34175	-0.19844305	0.26270767	13.9852316	-0.75537592	0.46255652	0.80854462
16.p.wholearm.chr16:1-38200000	0.40829931	-0.21803539	0.28928231	14.1176827	-0.75371144	0.46340846	0.80854462
chr17:70767943-71305641.BeroukhimS2.17q25.1.amp	0.15777778	0.09395347	0.12502391	13.9659218	0.75148408	0.46483745	0.80854462
chr16:45546775-51138307.LumA.16q12.1-120.del	0.02555435	-0.13490672	0.18026043	13.999261	-0.74839898	0.46660777	0.80867485
chr20:56249879-56298730.BeroukhimS5.amp	0.45475	0.15361221	0.20587512	13.9826479	0.74614266	0.46794107	0.80867485
chr17:58249995-58447640.BeroukhimS5.amp	0.2265	0.12766188	0.17236088	14.0162558	0.7406662	0.47112259	0.80873874
chr8:81242335-81979194.BeroukhimS2.8q21.13.amp	1.6545	0.48445584	0.65613182	13.972289	0.73835139	0.47252151	0.80873874
chr8:82099049-82634968.BeroukhimS5.amp	1.6545	0.48445584	0.65613182	13.972289	0.73835139	0.47252151	0.80873874
chr6:31910383-31915520.LumB.6p21.32-140.del	0.47575	0.20797099	0.28283415	13.9946868	0.73531075	0.47429378	0.80917852
18.p.wholearm.chr18:1-16100000	0.34055469	-0.17836955	0.24728165	14.0117023	-0.72132141	0.48257522	0.82068525
9.p.wholearm.chr9:1-51800000	0.47903721	-0.18899076	0.26460073	14.0031222	-0.71424883	0.48680962	0.82525821
chr22:20517661-21169423.BeroukhimS2.22q11.22.del	-0.0645	-0.10497971	0.15067552	13.9696037	-0.69672703	0.49740438	0.84049257
6.p.wholearm.chr6:1-60500000	0.6845779	0.21501224	0.3113315	14.0977412	0.69062156	0.50101676	0.84049257
chr22:22437934-22507511.BeroukhimS5.del	-0.0665	-0.10409579	0.15107747	13.9713702	-0.68902257	0.50209201	0.84049257
chr22:23139372-23249329.BeroukhimS5.amp	-0.0665	-0.10409579	0.15107747	13.9713702	-0.68902257	0.50209201	0.84049257
chr17:51748757-53292386.BeroukhimS5.amp	0.41356818	-0.17951796	0.26371154	13.9226555	-0.6807361	0.50720251	0.84607827
chr11:55503467-56066044.BeroukhimS5.amp	0.227	0.21796645	0.32191347	14.046061	0.67709639	0.50934348	0.84607827
chr2:32460827-55039898.BeroukhimS5.del	0.03585215	0.10822546	0.16016217	14.0545199	0.67572425	0.51018203	0.84607827
chr6:13729714-14088212.Basal.6p23-59.amp	0.705	0.20216994	0.30152617	14.015354	0.67048886	0.5134438	0.84885136
chr5:52119531-58183162.Basal.5q11.2-28.del	0.09671875	0.12151784	0.18220326	13.9918246	0.6669356	0.51566174	0.84988695
chr6:30420923-31893702.LumB.6p21.33-139.del	0.47343056	0.18764574	0.28844955	13.9963468	0.65053226	0.525884	0.86197572
chr14:37128947-38971371.Basal.14q21.1-92.del	-0.14775	-0.10998333	0.17064329	17	-0.64452187	0.52783906	0.86197572
chr14:50062686-50445987.BeroukhimS5.amp	-0.14775	-0.10998333	0.17064329	17	-0.64452187	0.52783906	0.86197572
chr19:21788507-34401877.BeroukhimS2.19p12.del	1.25382143	-0.28553597	0.44711575	13.9766375	-0.63861756	0.53339749	0.86570706
chr6:4058046-25852810.BeroukhimS5.del	0.83635	0.19256453	0.305056	14.0546685	0.63124323	0.53801287	0.86570706
chr9:34233377-35934224.BeroukhimS5.amp	0.30217157	-0.16767785	0.26657165	13.9858081	-0.62901605	0.53947733	0.86570706
chr17:24112056-24310787.BeroukhimS2.17q11.2.amp	0.40975	-0.15550975	0.24754508	13.9930415	-0.6282078	0.53998629	0.86570706
chr17:24936541-25468309.BeroukhimS5.amp	0.40975	-0.15550975	0.24754508	13.9930415	-0.6282078	0.53998629	0.86570706
chr16:87232502-87749670.LumA.16q24.3-131.del	0.29751667	-0.15383028	0.24591367	14.0046778	-0.62554584	0.54167323	0.86570706
chr7:16017926-18944036.BeroukhimS5.amp	-0.05706818	0.10425776	0.17021561	14.0339119	0.61250407	0.55000093	0.86570706
chr17:77653047-78605474.BeroukhimS5.amp	0.1765	-0.0871219	0.14240018	13.9597546	-0.61181032	0.55049825	0.86570706
chr17:78087533-78774742.BeroukhimS2.17q25.3.del	0.1765	-0.0871219	0.14240018	13.9597546	-0.61181032	0.55049825	0.86570706
22.q.wholearm.chr22:11800000-49691432	-0.02697079	0.07434802	0.12189042	14.0311505	0.60995782	0.55164092	0.86570706
chr1:58658784-60221344.BeroukhimS2.1p32.1.amp.JUN	-0.1045625	0.09454367	0.15534364	14.023453	0.60860984	0.55251447	0.86570706
chr12:68249634-68327233.BeroukhimS2.12q15.amp	0.26575	0.13302706	0.21892101	13.916927	0.60764866	0.55320738	0.86570706
chr8:11575198-11708771.BeroukhimS5.amp	-0.38375	-0.11835471	0.19750798	13.8879154	-0.59924015	0.55866221	0.86570706
chr17:55144989-55540417.BeroukhimS2.17q23.1.amp	0.39275	-0.157549	0.26383302	13.9286744	-0.59715423	0.55998711	0.86570706
chr10:75957348-77987139.LumB.10q22.2-146.del	-0.40175	-0.08569295	0.14417544	13.9740904	-0.59436582	0.56176763	0.86570706
chr19:8920808-8939190.BeroukhimS5.amp	-0.30075	-0.08689399	0.14820309	13.760809	-0.58631696	0.56715641	0.86570706
chr9:71280626-72167093.BeroukhimS5.amp	0.633	-0.15922153	0.27187659	14.1291957	-0.58563898	0.56735534	0.86570706
3.p.wholearm.chr3:1-91700000	0.29817996	0.11784084	0.20169156	13.3265815	0.58426259	0.56880271	0.86570706
chr1:118282114-118807006.BeroukhimS5.amp	0.492375	0.16940817	0.29450554	13.9932445	0.57522914	0.57427	0.86570706
chr8:42006632-42404492.BeroukhimS2.8p11.21.amp	2.00725	0.37202366	0.64857936	13.9254914	0.57359775	0.57538738	0.86570706
chr20:33386980-33969561.BeroukhimS5.amp	0.269	-0.07926249	0.1391043	13.983798	-0.56980619	0.57784909	0.86570706
chr14:54378476-55837783.Basal.14q22.3-95.del	-0.14775	0.15771971	0.27703891	14.1493233	0.56930526	0.57807544	0.86570706
chr13:108477140-110084607.BeroukhimS2.13q34.amp	-0.643	0.15429826	0.27185085	13.7197501	0.56758425	0.57948727	0.86570706
chr5:63292034-63701397.Basal.5q12.2-30.del	-0.5155	-0.10137819	0.17888158	14.0050596	-0.56673351	0.57986525	0.86570706
chr18:3240326-5937123.BeroukhimS5.del	0.419875	-0.14970042	0.265793	13.9967657	-0.56322184	0.58219469	0.86570706
chr11:74085123-74337880.LumB.11q13.4-153.del	0.14	0.10061181	0.17970594	13.9695388	0.55986914	0.58443519	0.86570706
chr11:74091491-74180816.BeroukhimS5.amp	0.14	0.10061181	0.17970594	13.9695388	0.55986914	0.58443519	0.86570706
chr3:86250885-95164178.BeroukhimS2.3p11.1.amp	0.62344444	-0.15421481	0.27994012	17	-0.550885	0.58887973	0.86570706
X.qwholearm.chrX:60000000-154913754	-0.30252649	-0.14191542	0.2594047	13.8622739	-0.54708112	0.59302161	0.86570706
chr11:68230745-68388280.BeroukhimS5.amp	0.599	-0.10951574	0.20026184	13.9452639	-0.54686273	0.59311657	0.86570706
chr3:36286137-40063081.BeroukhimS5.amp	0.53170946	0.11090731	0.2031282	13.9409783	0.54599662	0.59369859	0.86570706
chr19:59066340-59471027.BeroukhimS2.19q13.42.amp	0.87775	0.30910392	0.56871567	14.1458781	0.54351222	0.59523939	0.86570706
chr4:143168636-143603331.Basal.4q31.21-19.del	0.53775	-0.19004018	0.34995775	13.9447577	-0.54303751	0.59567801	0.86570706
chr18:17749667-22797232.BeroukhimS2.18q11.2.amp	0.07883696	-0.12638043	0.23456601	13.9299577	-0.53878408	0.59854131	0.86570706
chr6:38787570-41935171.BeroukhimS5.amp	0.87385156	0.21872369	0.40708152	14.1049681	0.53729702	0.59943698	0.86570706
chr11:76699529-78005085.BeroukhimS2.11q14.1.amp	0.14	0.09275881	0.17421624	13.9809381	0.53243496	0.60278454	0.86570706

chr10:123214869-123367187.BeroukhimS5.amp	0.1295	0.11886428	0.22663254	13.9905414	0.52448022	0.60815507	0.86570706
chr13:109973420-114110898.Basal.13q34-86.amp	-0.643	0.14622895	0.28017593	13.6955301	0.52191832	0.61006515	0.86570706
chr13:111767404-114142980.BeroukhimS2.13q34.del	-0.643	0.14622895	0.28017593	13.6955301	0.52191832	0.61006515	0.86570706
chr13:112030830-113770959.BeroukhimS5.amp	-0.643	0.14622895	0.28017593	13.6955301	0.52191832	0.61006515	0.86570706
chr4:1234177-1683843.Basal.4p16.3-4.del	0.0485	-0.11468988	0.22170905	14.1142642	-0.51729906	0.61295878	0.86570706
chrX:31041721-34564697.BeroukhimS2.Xp21.2.del.DMD	-0.1275	-0.13576088	0.26548277	13.9263279	-0.51137359	0.61710087	0.86570706
chr16:82644872-85172804.LumA.16q24.1-129.del	0.17689423	-0.10672198	0.20998988	14.0168536	-0.50822438	0.61920005	0.86570706
chr18:3200634-3858775.BeroukhimS5.amp	0.5669	-0.15542829	0.30653211	13.9922454	-0.50705385	0.6200139	0.86570706
chr10:118946990-119027085.LumB.10q25.3-147.del	-0.36475	0.11199235	0.2218146	13.9866217	0.5048917	0.62149629	0.86570706
chr10:119032598-121691235.LumB.10q26.11-148.del	-0.36475	0.11199235	0.2218146	13.9866217	0.5048917	0.62149629	0.86570706
chr10:122206456-122883255.LumB.10q26.12-149.del	-0.36475	0.11199235	0.2218146	13.9866217	0.5048917	0.62149629	0.86570706
chr1:43945805-44960161.Basal.1p34.1-2.amp	0.17957143	0.09659059	0.19149868	13.9958913	0.50439297	0.62183261	0.86570706
chr15:96891354-97698742.BeroukhimS2.15q26.3.amp.IGF1R	0.00475	0.07853745	0.15590552	14.5060916	0.50375026	0.62200048	0.86570706
chr13:27611014-27767472.LumB.13q12.2-162.del	-0.46075	0.09894143	0.19852944	13.9009238	0.4983716	0.62601449	0.86570706
chr15:1-24740084.BeroukhimS2.15q11.2.del	-0.26492647	-0.11762661	0.23777351	14.0060111	-0.49470024	0.62848117	0.86570706
8.p.wholearm.chr8:1-45200000	0.1408874	-0.08584001	0.17361192	13.9500358	-0.49443619	0.62869329	0.86570706
chr17:26185485-27216066.BeroukhimS2.17q11.2.del.NF1	0.44875	-0.11839158	0.24009649	13.98719	-0.49310003	0.62959313	0.86570706
chr9:21489625-22474701.BeroukhimS2.9p21.3.del.CDKN2A.I	0.44325	-0.13281036	0.26978288	14.0032705	-0.49228611	0.63014523	0.86570706
chr9:23819824-26891068.BeroukhimS5.del	0.44325	-0.13281036	0.26978288	14.0032705	-0.49228611	0.63014523	0.86570706
chr9:26573226-29470065.BeroukhimS5.amp	0.44325	-0.13281036	0.26978288	14.0032705	-0.49228611	0.63014523	0.86570706
chr20:45719063-49181611.LumB.20q13.13-167.amp	0.56866935	0.11377715	0.23206983	14.0112331	0.49027117	0.63153022	0.86570706
chr14:49113809-52311409.Basal.14q22.1-93.del	-0.14775	-0.08250714	0.17016374	17	-0.48486913	0.63395789	0.86570706
chr3:178149984-199501827.BeroukhimS2.3q28.amp.PIK3CA	0.67708615	0.11968556	0.24752075	13.9519051	0.48353748	0.63621484	0.86570706
chr12:1-1311104.BeroukhimS2.12p13.33.amp	0.889275	-0.14894873	0.30963794	13.9927708	-0.48104159	0.63792198	0.86570706
chr3:170024984-173604597.BeroukhimS2.3q26.2.amp.PRKCI	0.53725	0.09858388	0.20538126	14.2619259	0.48000427	0.63850284	0.86570706
chr8:42971602-72924037.BeroukhimS2.8q11.22.del.SNTG1	2.02626099	0.32715014	0.68562728	13.9485274	0.47715449	0.64064181	0.86570706
chr10:128584013-129240925.LumB.10q26.2-151.del	-0.33675	0.09839095	0.20676914	13.7148612	0.47584927	0.6416724	0.86570706
chr10:129812260-135374737.BeroukhimS2.1q26.3.del	-0.33675	0.09839095	0.20676914	13.7148612	0.47584927	0.6416724	0.86570706
chr15:35140533-43473382.BeroukhimS2.15q15.1.del	-0.4596078	-0.0700223	0.14792615	14.0102337	-0.47335984	0.64324746	0.86570706
chr15:49136093-51017946.Basal.15q21.2-105.del	-0.42825	-0.07144472	0.1510955	14.0096542	-0.47284476	0.64360619	0.86570706
chr15:51593230-55368004.Basal.15q21.3-106.del	-0.42825	-0.07144472	0.1510955	14.0096542	-0.47284476	0.64360619	0.86570706
chr15:56490060-57176541.Basal.15q22.1-107.del	-0.42825	-0.07144472	0.1510955	14.0096542	-0.47284476	0.64360619	0.86570706
chr15:57184612-61461128.Basal.15q22.2-108.del	-0.42825	-0.07144472	0.1510955	14.0096542	-0.47284476	0.64360619	0.86570706
chr15:61583863-61913200.Basal.15q22.31-109.del	-0.42825	-0.07144472	0.1510955	14.0096542	-0.47284476	0.64360619	0.86570706
chr16:85920445-87225756.LumA.16q24.2-130.del	0.1505	-0.10004397	0.21450852	14.0180541	-0.46638691	0.64810364	0.86666819
chr4:147085600-153449318.BeroukhimS5.del	0.53772059	-0.16504596	0.35635059	13.9426624	-0.46315614	0.65039916	0.86666819
17.q.wholearm.chr17:22200000-78774742	0.26742188	-0.07028866	0.15205326	13.9452121	-0.46226342	0.65102223	0.86666819
chr13:89500014-93206506.BeroukhimS2.13q31.3.amp	-0.448125	-0.09629054	0.20988916	13.9859906	-0.45876854	0.65344873	0.86666819
chr17:41015664-41353348.BeroukhimS5.amp	0.1995	0.06601354	0.14420955	13.9777801	0.45776122	0.65415889	0.86666819
chr14:52311662-54325595.Basal.14q22.2-94.del	-0.14775	0.11070357	0.24250143	14.2614084	0.45650686	0.65490077	0.86666819
chr10:73394126-73443318.LumB.10q22.1-145.del	-0.40175	0.07839761	0.17204686	13.8577075	0.4556759	0.65568156	0.86666819
chr20:59868877-61137250.BeroukhimS5.amp	1.1745	-0.19458779	0.43290505	14.0326365	-0.449493	0.6599407	0.87014403
chr10:54201467-57779853.BeroukhimS5.del	-0.39975	0.07784986	0.17471153	13.8566574	0.44559085	0.66277545	0.87172929
chr5:56146657-56594666.LumB.5q11.2-132.amp	-0.30275	0.13392088	0.30329086	13.9274716	0.44155925	0.66558636	0.87212996
chr8:41004735-41425303.BeroukhimS5.amp	2.00725	0.27586253	0.63015276	13.9298682	0.43777088	0.66826391	0.87212996
chr12:70849987-70966467.BeroukhimS2.12q21.1.amp	0.26575	0.09869115	0.22779285	13.9130229	0.43324957	0.67147495	0.87212996
chr12:95089777-95350380.BeroukhimS2.12q23.1.amp	-0.00575	0.07788333	0.18050173	17	0.43148248	0.67153976	0.87212996
chr8:1-392555.BeroukhimS2.8p23.3.del	-0.38375	-0.11167713	0.25844194	14.3399164	-0.43211691	0.67208355	0.87212996
chr12:50987234-51048711.BeroukhimS5.amp	0.025	-0.20149757	0.46729479	14.0198189	-0.43120013	0.6728793	0.87212996
chr2:79257340-80427237.BeroukhimS5.del	-0.10075	0.0620258	0.14546599	14.0733467	0.42639381	0.67627096	0.87297525
chr8:94587173-100685605.BeroukhimS5.amp	1.52673611	0.29051487	0.68241199	13.9659899	0.42571771	0.67680104	0.87297525
chr8:84799647-85622331.BeroukhimS5.amp	1.6545	0.29497732	0.70099146	13.9684745	0.42080016	0.68030338	0.87537833
chr12:94480961-96479233.BeroukhimS5.del	-0.00575	0.07532333	0.18093732	17	0.41629517	0.68240248	0.87596857
chr12:63329154-63614508.BeroukhimS5.amp	0.6475	0.18949447	0.46313514	14.0746696	0.40915588	0.68858354	0.87997762
chr6:117109043-117359993.LumB.6q22.2-142.del	-0.23475	0.05996593	0.14749038	13.9950386	0.40657521	0.69046933	0.87997762
chr6:119295855-120272161.BeroukhimS5.amp	-0.23475	0.05996593	0.14749038	13.9950386	0.40657521	0.69046933	0.87997762
chr12:64461446-64607139.BeroukhimS2.12q14.3.amp.HMG/	0.400375	0.13646034	0.34028849	14.1272105	0.40101368	0.6944097	0.88289233
chr4:170251982-170915637.Basal.4q33-24.del	0.339	-0.15429057	0.4039762	13.9640745	-0.38192985	0.70826263	0.89836638
chr6:42772314-43039595.Basal.6p21.1-60.amp	1.1695	0.16211978	0.43261115	14.1588641	0.37474711	0.71340187	0.90060662
chr6:43556800-44361368.BeroukhimS2.6p21.1.amp	1.1695	0.16211978	0.43261115	14.1588641	0.37474711	0.71340187	0.90060662
chr14:95740950-100105884.Basal.14q32.2-100.del	-0.0455	-0.04052406	0.1108836	14.1752003	-0.36546488	0.72016023	0.90417542
chr20:49438571-52269898.LumB.20q13.2-168.amp	0.6533	0.08327849	0.2297865	14.0087777	0.36241683	0.72244972	0.90417542
chr6:101000242-121511318.BeroukhimS2.6q22.1.del	-0.1369233	-0.03395489	0.0937581	13.9956295	-0.36215425	0.72264664	0.90417542
chr4:164612456-170167997.Basal.4q32.3-23.del	0.36369444	-0.14851658	0.41744691	13.9652243	-0.35577358	0.72732693	0.90417542
chr6:116369389-117096650.LumB.6q22.1-141.del	-0.23475	0.04633959	0.13087067	13.995776	0.35408692	0.72855151	0.90417542
chr6:149442557-149779188.BeroukhimS5.amp	0.09275	0.09631792	0.27482889	14.0828883	0.35046505	0.73117649	0.90417542
chr18:44506813-45038631.BeroukhimS5.amp	-0.17525	-0.12142305	0.34831452	13.9550277	-0.34860175	0.73259166	0.90417542
chr10:123223889-124739898.LumB.10q26.13-150.del	0.36377941	0.07042741	0.20321787	13.9947754	0.34656112	0.7340766	0.90417542
chr12:47446248-48387464.Basal.12q13.12-79.del	-0.447	0.06038486	0.17473725	14.0042192	0.34557517	0.73479811	0.90417542
chr6:157695838-157823719.BeroukhimS5.amp	0.09275	-0.04665702	0.13504661	14.0258917	-0.34548826	0.73485418	0.90417542
chr6:161612277-163134099.BeroukhimS2.6q26.del.PARK2	0.09275	-0.04665702	0.13504661	14.0258917	-0.34548826	0.73485418	0.90417542
chr19:32764651-33569283.BeroukhimS5.amp	0.6575	0.17996356	0.53298628	13.973223	0.33765139	0.74064471	0.90814687
chr14:57736586-58085299.Basal.14q23.1-96.del	-0.14775	-0.06010556	0.17924627	17	-0.33532388	0.74148322	0.90814687
chr17:75915141-77213225.BeroukhimS5.del	0.10713889	-0.04797176	0.1438473	13.9609558	-0.33349087	0.74371964	0.90880158
chr11:76710709-85146692.LumB.11q14.1-154.del	0.10647321	0.04891127	0.14913156	14.1246203	0.32797394	0.74774248	0.91055345
chr12:44599181-47397048.Basal.12q13.11-78.del	-0.42187931	0.05717999	0.17490528	14.0044405	0.32691976	0.7485636	0.91055345
chr12:55201993-55269875.Basal.12q13.3-82.del	0.5545	-0.13672075	0.4233865	14.0291048	-0.32292185	0.75151711	0.9120685
chr6:106604408-107782614.BeroukhimS5.amp	-0.04375	-0.03602528	0.11315002	13.9931845	-0.31838509	0.75489508	0.91409064
chr12:48421630-53099317.Basal.12q13.13-80.del	-0.3581875	0.04121364	0.13170067	14.0005891	0.31293419	0.75894359	0.91519793
chr13:92308911-94031607.BeroukhimS2.13q31.3.del.GPC6	-0.62583333	0.06055221	0.19404177	14.1565391	0.31205763	0.75954562	0.91519793
13.q.wholearm.chr13:16000000-114142980	-0.32128483	0.04609625	0.14858203	13.9903375	0.31024107	0.76095109	0.91519793
chr4:186684565-191273063.BeroukhimS2.4q35.2.del	0.33564286	-0.12227655	0.40557518	13.9640352	-0.30148925	0.76748447	0.91931325
chr5:58300629-62108926.Basal.5q12.1-29.del	-0.3699375	-0.05141893	0.17100205	13.9961877	-0.30069187	0.7680698	0.91931325
chr17:39354620-40542083.BeroukhimS5.del	0.0105	0.03874255	0.13197106	13.9728477	0.29356849	0.77340288	0.91931325
chr8:2053441-6259545.BeroukhimS2.8p23.2.del.CSMD1	-0.38375	0.06710437	0.23013924	14.0695376	0.29158163	0.77486137	0.91931325
chr17:37319013-37988602.BeroukhimS2.17q21.2.del	0.1825	-0.05443002	0.18687315	13.9083957	-0.29126721	0.77514559	0.91931325
chr6:76630464-105342994.BeroukhimS2.6q16.1.del	-0.19980793	-0.03263027	0.11322511	13.9949601	-0.2881893	0.7774259	0.91931325
chr3:175446835-178263192.BeroukhimS2.3q26.31.del.NAAL/	0.46975	0.06189444	0.21552899	17	0.28717456	0.77744966	0.91931325
chr19:49818193-50114786.BeroukhimS5.amp	0.06325	-0.0474828	0.16650224	14.0436268	-0.28517816	0.77967026	0.91931325
chrX:14236816-19468566.BeroukhimS5.amp	-0.1275	-0.07355511	0.25813947	13.9744994	-0.28494331	0.77986686	0.91931325
chr18:46172638-49935241.BeroukhimS2.18q21.2.del.SMAD4	-0.19261111	-0.09691274	0.34615913	13.9547159	-0.27996587	0.78361089	0.9216921
chrX:66436234-67090514.BeroukhimS2.Xq12.amp.AR	-0.467	0.09127914	0.33259209	13.6380258	0.27444772	0.78785395	0.92464618
chr9:97183463-97625342.BeroukhimS5.del	0.46075	0.0663672	0.24620964	14.2817944	0.26955564	0.79135769	0.92672151
chr8:62174237-62716885.BeroukhimS5.amp	2.1805	0.19424119	0.72854374	13.9516718	0.26661569	0.79366521	0.92738998
chr4:174326479-176135905.Basal.4q34.1-25.del	0.27175	-0.1066638	0.40514208	13.9633344	-0.26327504	0.79618377	0.9283016
chr3:43362938-44130419.BeroukhimS5.del	0.2295	0.04986025	0.19210171	13.8608018	0.25955132	0.79902522	0.92932426
chr14:56307190-56415610.BeroukhimS5.amp	-0.14775	0.07041667	0.27438575	17	0.25663383	0.8005415	0.92932426
chr13:1-23902184.BeroukhimS2.13q12.11.del	-0.38912097	0.04384254	0.17427658	13.9227589	0.25156874	0.80504966	0.93253041
chr3:66644057-68118027.BeroukhimS5.del	0.3355	0.04659176	0.18907619	13.9062108	0.24641788	0.80895897	0.9327888
chr3:70107050-74383584.BeroukhimS5.del	0.3355	0.04588367	0.18907753	13.9058356	0.24267121	0.81180284	0.9327888
chr13:46362859-48209064.BeroukhimS2.13q14.2.del.RB1	-0.19822222	0.05216891	0.22320352	14.0139204	0.23372798	0.81857597	0.9327888
15.q.wholearm.chr15:17000000-100338915	-0.30799829	-0.03048131	0.13062868	14.0195591	-0.23334316	0.81886757	0.9327888
chr4:151218876-154929678.Basal.4q31.3-20.del	0.3375375	-0.08541197	0.36902128	13.9601986	-0.2314554	0.82031914	0.9327888
20.q.wholearm.chr20:27100000-62435964	0.49011538	0.04134539	0.18104728	13.9949366	0.22836791	0.82266334	0.9327888
chr12:53136012-55168448.Basal.12q13.2-81.del	-0.175255	0.03367331	0.14785216	13.9783091	0.22774985	0.82313835	0.9327888
chr12:38788913-42596599.BeroukhimS2.12q12.amp	-0.27473077	0.04006055	0.17749185	13.9981993	0.22570361	0.82469401	0.9327888
chrX:43505580-46847125.BeroukhimS5.amp	-0.162	0.06206253	0.27868955	13.9138854	0.22269414	0.82700948	0.9327888
chr12:97551177-99047626.BeroukhimS2.12q23.1.del.ANKS1I	-0.00575	0.03395	0.15666691	17	0.2167018	0.83101996	0.9327888
chr12:131913408-132349534.BeroukhimS2.12q24.33.del	0.07075	0.05824742	0.26804271	14.2848753	0.2173065	0.83104272	0.9327888
chr21:16248572-25859193.BeroukhimS5.del	0.2058	0.05293771	0.24518898	13.9613921	0.21590574	0.83218377	0.9327888
chr4:175486008-184028292.BeroukhimS5.del	0.34938333	-0.08882849	0.41302699	13.9544937	-0.21506705	0.83282648	0.9327888
chr4:1-435793.BeroukhimS2.4p16.3.del	0.3145	-0.10770555	0.50239026	14.0136772	-0.21438622	0.83333411	0.9327888
chr3:58626894-61524607.BeroukhimS2.3p14.2.del.FHIT	0.1514375	0.04594997	0.21726606	14.0954981	0.21149172	0.83553094	0.9327888
chr18:75796373-76117153.BeroukhimS2.18q23.del	-0.279	-0.06134103	0.2940864	13.9140556	-0.20858167	0.83779736	0.9327888
chr4:162524501-164492534.Basal.4q32.2-22.del	0.28575	-0.0867715	0.42382541	13.9641827	-0.20473407	0.84073403	0.9327888
chrX:1-3243111.BeroukhimS2.Xp22.33.del	-0.1275	0.03924784	0.19322662	13.9810283	0.20311819	0.84196909	0.9327888
chrX:6695466-8345492.BeroukhimS5.del	-0.1275	0.03924784	0.19322662	13.9810283	0.20311819	0.84196909	0.9327888
chrX:8707411-9925292.BeroukhimS5.del	-0.1275	0.03924784	0.19322662	13.9810283	0.20311819	0.84196909	0.9327888
chr18:1-587750.BeroukhimS2.18p11.32.del	0.19125	-0.05551107	0.27676786	14.013473	-0.20056906	0.8439173	0.9327888
chr20:61329497-62435964.BeroukhimS2.2q13.33.amp	1.128	-0.08824018	0.44162125	14.0327216	-0.19980963	0.84449602	0.9327888
chr20:51603033-51989829.BeroukhimS2.2q13.2.amp	0.69025	0.04534969	0.23244181	14.0076939	0.19510125	0.84811525	0.9327888
chr15:88253886-88997242.BeroukhimS5.amp	-0.309	0.02971857	0.15232666	13.9873235	0.19509762	0.84812208	0.9327888
chr4:155375138-160500747.Basal.4q32.1-21.del	0.27426852	-0.08159546	0.42051894	13.9644735	-0.19403515	0.84894262	0.9327888
chr16:45393460-45522699.LumA.16q11.2-119.del	0.01575	-0.04737948	0.25059462	14.0172525	-0.18906823	0.85274957	0.93504778
chr10:74560456-82020637.BeroukhimS2.1q22.3.amp	-0.432135	0.02770296	0.15097158	13.9493543	0.18349785	0.85704792	0.93515533
chr11:92342437-95765250.LumB.11q21-157.del	-0.043	-0.02292617	0.12651132	14.0481961	-0.18121835	0.85878477	0.93515533
chr15:75011134-75564998.Basal.15q24.3-111.del	-0.142	-0.02893031	0.16159596	13.9942241	-0.17902867	0.8604813	0.93515533
chr5:57754754-59053198.BeroukhimS2.5q11.2.del.PDE4D	-0.30275	-0.02965064	0.16856609	13.9812678	-0.17589922	0.86289539	0.93515533
chr5:58300629-59320301.LumB.5q12.1-133.amp	-0.30275	-0.02965064	0.16856609	13.9812678	-0.17589922	0.86289539	0.93515533
chr10:89467202-90419015.BeroukhimS2.1q23.31.del.PTEN	-0.55875	0.02323246	0.13252743	13.9811385	0.17530305	0.86335502	0.93515533
chr11:101433436-102134907.BeroukhimS2.11q22.2.amp.BIR	-0.043	-0.02142091	0.12640255	14.047561	-0.16946582	0.86784644	0.93543176
chr11:130280899-134452384.BeroukhimS2.11q25.del.OPCM	-0.3995	0.02062715	0.12260474	13.9908544	0.16824101	0.86880155	0.93543176
chr13:72212826-74986423.BeroukhimS5.del	-0.30475	-0.02860576	0.17263848	14.0084577	-0.16569749	0.8707626	0.93543176
18.q.wholearm.chr18:16100000-76117153	-0.17581692	-0.04358998	0.26587231	13.9359388	-0.16395081	0.87212392	0.93543176
X.pwholearm.chrX:1-60000000	-0.14968894	0.04004942	0.24590228	13.9323263	0.16286723	0.87296167	0.93543176
chr13:1-40829685.BeroukhimS2.13q12.2.amp	-0.21745082	0.02183485	0.14156355	13.9928741	0.15424065	0.87962251	0.93543176
chr20:29526118-29834552.BeroukhimS2.2q11.21.amp.BCL2L	0.269	-0.02780018	0.18348188	13.9757435	-0.15151457	0.88173518	0.93543176
chr15:65899096-66370691.Basal.15q23-110.del	-0.35575	0.02146505	0.15421431	14.0056545	0.13918973	0.89128188	0.93543176
chr6:129217882-131730157.BeroukhimS5.amp	0.09275	0.02198351	0.1599154	14.0375715	0.13746964	0.89261192	0.93543176
chr6:135561194-135665525.BeroukhimS2.6q23.3.amp.MYB	0.09275	0.02198351	0.1599154	14.0375715	0.13746964	0.89261192	0.93543176
chr6:137619861-138589104.BeroukhimS5.amp	0.09275	0.02198351	0.1599154	14.0375715	0.13746964	0.89261192	0.93543176
chr6:139381116-139689363.BeroukhimS5.amp	0.09275	0.02198351	0.1599154	14.0375715	0.13746964	0.89261192	0.93543176
chr6:143157329-144194971.BeroukhimS5.amp	0.09275	0.02198351	0.1599154	14.0375715	0.13746964	0.89261192	0.93543176
chr13:27774389-30804409.LumB.13q12.3-163.del	-0.09961667	0.02716764	0.19773858	14.0017015	0.13739172	0.89267732	0.93543176
chr11:73357303-74035012.BeroukhimS5.amp	0.37727778	-0.03100005	0.23083929	14.007481	-0.13429279	0.89508153	0.93543176
10.q.wholearm.chr10:40300000-135374737	-0.28648423	0.01926898	0.14506676	13.7950981	0.13282835	0.8962472	0.93543176
chr1:71284749-74440273.BeroukhimS2.1p31.1.del.NEGR1	-0.17075	-0.01688388	0.12809311	13.9948929	-0.13180941	0.89701127	0.93543176
chr12:56419524-56488685.BeroukhimS2.12q14.1.amp.CDK4	0.6475	-0.05425816	0.41294285	14.0536734	-0.13139386	0.89732622	0.93543176
chr12:56621627-57600529.Basal.12q14.1-83.del	0.6475	-0.05425816	0.41294285	14.0536734	-0.13139386	0.89732622	0.93543176
chr18:32113759-32649538.BeroukhimS5.del	-0.17525	-0.03297344	0.2564964	13.9524632	-0.12855322	0.89954582	0.93543176
chrX:69545961-73353657.BeroukhimS5.amp	-0.41877027	0.04144165	0.32502215	13.6789349	0.12750408	0.90039686	0.93543176
chr11:104262627-109547776.LumB.11q22.3-158.del	-0.05730357	-0.01478383	0.12414721	14.0394873	-0.11908308	0.9068967	0.94035503
chr8:39008109-41238710.BeroukhimS2.8p11.22.del	1.63006818	0.06199331	0.60091705	13.9757031	0.1031645	0.91929814	0.95136668
6.q.wholearm.chr6:60500000-170899992	-0.01079325	-0.00902442	0.10996096	13.9983767	-0.08206932	0.93575353	0.96323314
chr4:176791083-177486490.Basal.4q34.2-26.del	0.27175	-0.0347141	0.42979427	13.9443693	-0.08076911	0.93677337	0.96323314
chr2:173927807-175001974.HER2-enriched.2q31.1-118.amp	-0.0445	0.01443333	0.18002015	17	0.08017621	0.93703341	0.96323314
chr17:36989609-37319012.BeroukhimS5.amp	0.09353846	0.01283129	0.16196079	13.9417927	0.07922468	0.93797984	0.96323314
chr5:39149695-41885012.BeroukhimS5.amp	0.807	-0.02219417	0.29889714	14.1542294	-0.07425354	0.94184786	0.96460307
chr10:52313829-53768264.BeroukhimS2.1q11.23.del.PRKG1	-0.39975	0.01147152	0.15926254	13.9374359	0.07202899	0.94360226	0.96460307
chr11:85633463-87710586.LumB.11q14.2-155.del	-0.043	0.00987902	0.13998367	14.0443145	0.07057264	0.94473297	0.96460307
chr10:61909390-66364553.BeroukhimS5.amp	-0.23413462	0.01085769	0.16018327	17	0.06778294	0.94674929	0.96481702
chr15:37880223-38115089.Basal.15q15.1-104.del	-0.599125	0.00994255	0.15610117	13.9852078	0.06369298	0.95011612	0.96640383
chr12:34188071-44391168.BeroukhimS5.del	0.10819792	0.01143821	0.18893802	13.9648141	0.06053949	0.95258383	0.9670718
chr17:35067383-35272328.BeroukhimS2.17q12.amp.ERBB2	0.0105	-0.00988943	0.17546942	13.9431568	-0.05635987	0.9558548	0.96855116
chr11:69098089-69278404.BeroukhimS2.11q13.2.amp.CCND	0.599	0.01008653	0.19917294	13.9605728	0.05064205	0.9603282	0.97124102
chr20:22719364-23648289.BeroukhimS5.amp	0.10289286	-0.00750225	0.17346548	13.9860388	-0.04324923	0.96611426	0.97524578
chr11:82612034-85091467.BeroukhimS2.11q14.1.del.DLG2	0.02265625	0.00345399	0.14251574	14.1208057	0.02423582	0.98100367	0.98804907
chr13:31211674-31275009.LumB.13q13.1-164.del	0.07625	-0.00678979	0.30404461	14.0184482	-0.02233156	0.98249824	0.98804907
chr17:22479313-22877776.BeroukhimS2.17q11.1.amp	0.40975	0.00597301	0.34866567	13.9523494	0.01713104	0.98657464	0.99011652
chr11:87881006-92269284.LumB.11q14.3-156.del	-0.043	0.00200787	0.13407692	14.043846	0.01497554	0.98826237	0.99011652
chr12:42034279-44588086.Basal.12q12-77.del	-0.06602083	0.00114129	0.18252293	13.9681226	0.00625285	0.99509941	0.99509941

Supplementary Data 6

Linear mixed model - DNA - Basal-like samples

Segment	Intercept	Coef	Std.err	df	T.value	P.value	P.adj
chr5:174477192-180857866.BeroukhimS2.5q35.3.amp	0.88803261	-0.83723188	0.17131044	6	-4.88722058	0.0027461	0.27591259
chr5:176660805-180009206.Basal.5q35.3-58.del	0.895	-0.819	0.16830792	6	-4.8660812	0.0028055	0.27591259
chr5:159922707-167623739.Basal.5q34-55.del	0.895	-0.85716667	0.17676014	6	-4.84932114	0.00285362	0.27591259
chr5:177541057-180857866.BeroukhimS2.5q35.3.del	0.88043182	-0.78114015	0.16247097	6	-4.80787511	0.00297669	0.27591259
chr5:152850499-154326721.Basal.5q33.2-53.del	0.895	-0.80666667	0.16917622	6	-4.7682037	0.00310014	0.27591259
chr5:155686345-159845035.Basal.5q33.3-54.del	0.895	-0.80666667	0.16917622	6	-4.7682037	0.00310014	0.27591259
chr5:167651670-171366482.Basal.5q35.1-56.del	0.895	-1.12666667	0.2592778	5	-4.34540352	0.00739115	0.49335894
chr5:172591744-174888201.Basal.5q35.2-57.del	0.895	-1.12666667	0.2592778	5	-4.34540352	0.00739115	0.49335894
5.q.wholearm.chr5:47700000-180857866	0.28622042	-0.58605469	0.14588152	5.00000003	-4.0173333	0.01014754	0.55369956
chr5:147238467-151765017.Basal.5q33.1-52.del	0.49992105	-0.68371637	0.17838158	5.00000009	-3.83288659	0.01221174	0.55369956
chr5:130522776-135427403.Basal.5q31.1-48.del	0.413	-0.68375287	0.18059697	5	-3.7860706	0.0128099	0.55369956
chr5:135493104-138988200.Basal.5q31.2-49.del	0.413	-0.65666667	0.17916448	5.00000001	-3.66516101	0.01451647	0.55369956
chr5:139008130-142795270.Basal.5q31.3-50.del	0.413	-0.65666667	0.17916448	5.00000001	-3.66516101	0.01451647	0.55369956
chr5:143171919-147191453.Basal.5q32-51.del	0.413	-0.65666667	0.17916448	5.00000001	-3.66516101	0.01451647	0.55369956
chr5:127447382-129549383.Basal.5q23.3-47.del	0.413	-0.752	0.23671699	5.00000008	-3.17678935	0.02462668	0.59134795
chr5:128458341-128477620.LumB.5q23.3-138.amp	0.413	-0.752	0.23671699	5.00000008	-3.17678935	0.02462668	0.59134795
chr1:201678483-203358272.BeroukhimS2.1q32.1.amp.MDM4	0.9035	1.012	0.33983329	5.00000007	2.97793075	0.03087346	0.59134795
chr5:98132900-102642260.Basal.5q21.1-39.del	-0.0737	-0.51738333	0.17703961	5	-2.92241562	0.03292126	0.59134795
chr5:85837489-133480433.BeroukhimS2.5q21.1.del.APC	0.04856908	-0.54071491	0.18638266	5	-2.90110096	0.03374741	0.59134795
chr2:53680282-53845245.BeroukhimS5.amp	0.7425	0.35366667	0.12495937	5.00000001	2.83025326	0.03666381	0.59134795
1.q.wholearm.chr1:124300000-247249719	1.53324592	0.46587958	0.16673442	5.00000082	2.79414156	0.03825727	0.59134795
chr8:39008109-41238710.BeroukhimS2.8p11.22.del	1.40963636	-0.74398485	0.27281015	5	-2.72711567	0.04142229	0.59134795
2.p.wholearm.chr2:1-93300000	0.27825054	0.22239237	0.08186709	5.00000001	2.71650497	0.04194938	0.59134795
4.p.wholearm.chr4:1-50700000	0.25684977	-0.59597261	0.23171547	6	-2.57200184	0.04222114	0.59134795
chr4:37121738-38804810.Basal.4p14-12.del	0.7865	-0.97860606	0.39927524	6	-2.45095602	0.04972616	0.59134795
chr5:121675719-127022221.Basal.5q23.2-46.del	0.28102778	-0.65833333	0.26779642	5	-2.45833505	0.05734581	0.59134795
chr7:83246850-83689170.BeroukhimS5.amp	-0.3695	0.32633333	0.13294074	5.00000055	2.45472774	0.0576006	0.59134795
chr7:86091574-88424037.BeroukhimS5.amp	-0.3695	0.32633333	0.13294074	5.00000055	2.45472774	0.0576006	0.59134795
chr7:87797038-89779004.BeroukhimS5.del	-0.3695	0.32633333	0.13294074	5.00000055	2.45472774	0.0576006	0.59134795
chr8:42971602-72924037.BeroukhimS2.8q11.22.del.SNTG1	2.14917582	-0.86298352	0.35329216	5	-2.44269086	0.05845975	0.59134795
chr11:82612034-85091467.BeroukhimS2.11q14.1.del.DLG2	0.0948125	-0.3713125	0.15946537	6	-2.32848354	0.05876256	0.59134795
chr10:74560456-82020637.BeroukhimS2.1q22.3.amp	-0.63896	0.17940333	0.07404502	5.00000012	2.4228954	0.05990308	0.59134795
chr10:52313829-53768264.BeroukhimS2.1q11.23.del.PRKG1	-0.65	0.21220833	0.08890724	5	2.38685095	0.06263134	0.59134795
chr22:37740208-38258806.Basal.22q13.1-113.amp	-0.3515	0.3825	0.16869977	6	2.26734161	0.06390054	0.59134795
chr10:73394126-73443318.LumB.10q22.1-145.del	-0.654	0.36383333	0.15493341	5	2.34832072	0.06569709	0.59134795
chr5:125787000-126200608.LumB.5q23.2-137.amp	0.413	-0.7051	0.30143927	5.00000166	-2.33911132	0.06645357	0.59134795
chr4:41057559-42353879.Basal.4p13-13.del	0.7865	-0.91816667	0.41037975	6	-2.23735862	0.0665885	0.59134795
chr5:63292034-63701397.Basal.5q12.2-30.del	-0.8595	-0.29866667	0.12795772	4.99999941	-2.33410426	0.06686883	0.59134795
chr5:63838208-66501179.Basal.5q12.3-31.del	-0.8595	-0.29866667	0.12795772	4.99999941	-2.33410426	0.06686883	0.59134795
chr5:66513872-67633403.Basal.5q13.1-32.del	-0.8595	-0.29866667	0.12795772	4.99999941	-2.33410426	0.06686883	0.59134795
chr5:68425638-71536822.LumB.5q13.2-134.amp	-0.8595	-0.29866667	0.12795772	4.99999941	-2.33410426	0.06686883	0.59134795
chr5:68425638-72913540.Basal.5q13.2-33.del	-0.8595	-0.29866667	0.12795772	4.99999941	-2.33410426	0.06686883	0.59134795
chr5:73958991-76396789.Basal.5q13.3-34.del	-0.8595	-0.29866667	0.12795772	4.99999941	-2.33410426	0.06686883	0.59134795
chr16:1-359092.BeroukhimS2.16p13.3.del	0.115	-0.60083333	0.26065807	5	-2.30506326	0.06933294	0.59134795
chr2:61834240-63151654.BeroukhimS5.amp	0.4820625	0.23502083	0.10313595	5	2.27874792	0.0716502	0.59134795
chr5:58300629-62108926.Basal.5q12.1-29.del	-0.568375	-0.19227778	0.08487615	5	-2.26539237	0.07285792	0.59134795
chr16:76685816-78205652.BeroukhimS2.16q23.1.del.WWOX	0.7255	-0.6055	0.26765061	5	-2.26227766	0.0731427	0.59134795
chr16:79132355-80549399.LumA.16q23.2-127.del	0.7255	-0.6055	0.26765061	5	-2.26227766	0.0731427	0.59134795
chr10:54201467-57779853.BeroukhimS5.del	-0.65	0.35066667	0.15585984	4.99998114	2.24988472	0.07428787	0.59134795
chr2:1-15244284.BeroukhimS2.2p25.3.del	0.72226415	0.4885283	0.21736798	5.00000001	2.24747132	0.07451286	0.59134795
chr11:76710709-85146692.LumB.11q14.1-154.del	0.26244643	-0.38025595	0.17067678	5	-2.22793017	0.07636334	0.59134795
chr11:73357303-74035012.BeroukhimS5.amp	0.3295	-0.38383333	0.17250457	5.00000001	-2.22506179	0.07663907	0.59134795
chr11:74085123-74337880.LumB.11q13.4-153.del	0.3295	-0.38383333	0.17250457	5.00000001	-2.22506179	0.07663907	0.59134795
chr11:74091491-74180816.BeroukhimS5.amp	0.3295	-0.38383333	0.17250457	5.00000001	-2.22506179	0.07663907	0.59134795
chr11:76699529-78005085.BeroukhimS2.11q14.1.amp	0.3295	-0.38383333	0.17250457	5.00000001	-2.22506179	0.07663907	0.59134795
chr4:91089383-93486891.BeroukhimS2.4q22.1.del	0.8085	-0.81555556	0.38386432	6	-2.12459326	0.0777882	0.59134795
chr11:85633463-87710586.LumB.11q14.2-155.del	-0.0365	-0.353	0.16167787	5	-2.18335386	0.08077061	0.59134795
chr11:87881006-92269284.LumB.11q14.3-156.del	-0.0365	-0.353	0.16167787	5	-2.18335386	0.08077061	0.59134795
chr11:92342437-95765250.LumB.11q21-157.del	-0.0365	-0.353	0.16167787	5	-2.18335386	0.08077061	0.59134795
chr11:101433436-102134907.BeroukhimS2.11q22.2.amp.BIRC	-0.0365	-0.353	0.16167787	5	-2.18335386	0.08077061	0.59134795
chr1:174611641-175371076.BeroukhimS5.amp	1.9095	0.3635	0.16657346	5	2.18222042	0.08088615	0.59134795
chr16:73739922-78192112.LumA.16q23.1-126.del	0.7255	-0.56146212	0.25809571	4.99999999	-2.17540276	0.08158481	0.59134795
chr4:5577784-10295484.Basal.4p16.1-6.del	0.01073529	-0.44468137	0.21411028	6	-2.07688009	0.08309267	0.59134795
chr11:104262627-109547776.LumB.11q22.3-158.del	-0.06510714	-0.338375	0.15713656	5.00000012	-2.15338171	0.08388564	0.59134795
chr16:51646446-54466783.LumA.16q12.2-121.del	0.7255	-0.5335	0.25873249	6	-2.06197531	0.08482423	0.59134795
chr16:54782803-56638303.LumA.16q13-122.del	0.7255	-0.5335	0.25873249	6	-2.06197531	0.08482423	0.59134795
chr16:56705000-65205296.LumA.16q21-123.del	0.7255	-0.5335	0.25873249	6	-2.06197531	0.08482423	0.59134795
chr16:56791457-72871981.BeroukhimS5.del	0.7255	-0.5335	0.25873249	6	-2.06197531	0.08482423	0.59134795

chr16:65206155-69392572.LumA.16q22.1-124.del	0.7255	-0.5335	0.25873249	6	-2.06197531	0.08482423	0.59134795
chr16:69398793-73198518.LumA.16q22.3-125.del	0.7255	-0.5335	0.25873249	6	-2.06197531	0.08482423	0.59134795
X.pwholearm.chrX:1-60000000	-0.45376403	0.19158966	0.09007117	5.00000114	2.12709182	0.08672292	0.59134795
chr5:76408288-81082828.Basal.5q14.1-35.del	-0.15870588	-0.36028922	0.16992447	5.00000117	-2.12029033	0.08747338	0.59134795
chr8:1-392555.BeroukhimS2.8p23.3.del	-0.143	-0.59216667	0.2826217	5	-2.09526253	0.09029454	0.59134795
chr1:58658784-60221344.BeroukhimS2.1p32.1.amp.JUN	-0.164125	-0.17333333	0.08288322	5	-2.0912959	0.0907504	0.59134795
chr5:57754754-59053198.BeroukhimS2.5q11.2.del.PDE4D	-0.434	-0.14316667	0.06869613	5.00000118	-2.0840571	0.09158857	0.59134795
chr5:58300629-59320301.LumB.5q12.1-133.amp	-0.434	-0.14316667	0.06869613	5.00000118	-2.0840571	0.09158857	0.59134795
chr5:49730236-50173926.Basal.5q11.1-27.del	0.1795	0.3445	0.16652821	5.00000053	2.06871857	0.09339169	0.59134795
chr9:137859478-140273252.BeroukhimS2.9q34.3.amp	1.15303896	-0.86722294	0.4373444	6	-1.98292913	0.09463894	0.59134795
chr8:81242335-81979194.BeroukhimS2.8q21.13.amp	1.366	-0.49316667	0.24161311	5	-2.04114201	0.09672794	0.59134795
chr8:82099049-82634968.BeroukhimS5.amp	1.366	-0.49316667	0.24161311	5	-2.04114201	0.09672794	0.59134795
chr5:101597589-102642260.LumB.5q21.1-135.amp	0.049	-0.57413889	0.28527739	5.00000001	-2.01256361	0.10031757	0.59134795
chr4:76623381-79684447.Basal.4q21.1-16.del	0.66651852	-0.72758025	0.37953386	6	-1.91703644	0.10369133	0.59134795
chr5:102912456-102926389.Basal.5q21.2-40.del	0.049	-0.57216667	0.28866883	5.00000001	-1.98208675	0.1042995	0.59134795
chr5:102912456-102926389.LumB.5q21.2-136.amp	0.049	-0.57216667	0.28866883	5.00000001	-1.98208675	0.1042995	0.59134795
chr5:106744250-109231328.Basal.5q21.3-41.del	0.049	-0.57216667	0.28866883	5.00000001	-1.98208675	0.1042995	0.59134795
chr5:109877429-111137815.Basal.5q22.1-42.del	0.049	-0.57216667	0.28866883	5.00000001	-1.98208675	0.1042995	0.59134795
chr5:111507433-112958878.Basal.5q22.2-43.del	0.049	-0.57216667	0.28866883	5.00000001	-1.98208675	0.1042995	0.59134795
chr5:113725565-115180304.Basal.5q22.3-44.del	0.049	-0.57216667	0.28866883	5.00000001	-1.98208675	0.1042995	0.59134795
chr5:115193714-121517156.Basal.5q23.1-45.del	0.049	-0.57216667	0.28866883	5.00000001	-1.98208675	0.1042995	0.59134795
chr4:13238819-37565122.BeroukhimS5.del	0.19470833	-0.53234375	0.26967768	5	-1.97400007	0.10538349	0.59134795
chr8:62174237-62716885.BeroukhimS5.amp	2.418	-1.14891667	0.58864568	5.00000014	-1.95179664	0.10842047	0.59134795
16.q.wholearm.chr16:38200000-88827254	0.71447411	-0.48048774	0.25796074	6	-1.86263903	0.11181398	0.59134795
chr4:1-435793.BeroukhimS2.4p16.3.del	-0.0475	-0.43316667	0.23424674	6	-1.8491897	0.11391799	0.59134795
chr4:1234177-1683843.Basal.4p16.3-4.del	-0.0475	-0.43316667	0.23424674	6	-1.8491897	0.11391799	0.59134795
chr4:3220565-5553626.Basal.4p16.2-5.del	-0.0475	-0.43316667	0.23424674	6	-1.8491897	0.11391799	0.59134795
chr4:4349500-4427489.BeroukhimS5.amp	-0.0475	-0.43316667	0.23424674	6	-1.8491897	0.11391799	0.59134795
8.p.wholearm.chr8:1-45200000	0.50733264	-0.52139187	0.27259007	5	-1.91273244	0.11398607	0.59134795
chr4:19864333-22430289.Basal.4p15.31-9.del	0.11333333	-0.512	0.27225635	5	-1.88058058	0.11878808	0.59134795
chr1:148661965-149063439.BeroukhimS2.1q21.2.amp.MCL1	1.868	0.27633333	0.14805352	5.00000001	1.86644219	0.12096521	0.59134795
chr1:151026302-152973244.BeroukhimS5.amp	1.868	0.27633333	0.14805352	5.00000001	1.86644219	0.12096521	0.59134795
chr8:38252951-38460772.BeroukhimS2.8p12.amp.FGFR1	1.0205	-0.57033333	0.30850074	5.00000015	-1.84872599	0.12375125	0.59134795
chr12:85072329-85674601.BeroukhimS2.12q21.32.amp	-0.1995	0.44616667	0.25008839	6	1.78403589	0.12467813	0.59134795
chr4:104889590-106814504.BeroukhimS5.del	0.4897	-0.59116667	0.33141834	6	-1.7837476	0.1247279	0.59134795
chr12:76852527-77064746.BeroukhimS2.12q21.2.amp	-0.1995	0.43916667	0.24671257	6	1.78007414	0.12536378	0.59134795
chr9:130999928-132303780.BeroukhimS5.del	1.026	-0.763	0.42992583	6	-1.77472471	0.12629547	0.59134795
chr4:10979549-15266111.Basal.4p15.33-7.del	0.3485	-0.513	0.28141393	5.00000001	-1.82293748	0.12792475	0.59134795
chr4:15313739-17632477.Basal.4p15.32-8.del	0.3485	-0.513	0.28141393	5.00000001	-1.82293748	0.12792475	0.59134795
chr9:110661885-116923292.BeroukhimS5.del	1.026	-0.57832143	0.3277792	6	-1.76436283	0.12811951	0.59134795
chr4:30331135-30753569.Basal.4p15.1-11.del	-0.0865	-0.49133333	0.27033185	5.00000015	-1.81751925	0.12881974	0.59134795
chr4:79691766-80079606.Basal.4q21.21-17.del	0.4095	-0.7455	0.41138066	5.00000001	-1.81219019	0.12970625	0.59134795
chr4:83634873-83961360.BeroukhimS5.del	0.4095	-0.7455	0.41138066	5.00000001	-1.81219019	0.12970625	0.59134795
chrX:43505580-46847125.BeroukhimS5.amp	-0.4565	0.32788889	0.18235354	5.00000028	1.79809446	0.13208121	0.59134795
chr8:42006632-42404492.BeroukhimS2.8p11.21.amp	2.164	-0.48210417	0.26898884	5	-1.79228316	0.1330732	0.59134795
chr4:71492582-75938920.Basal.4q13.3-15.del	0.672	-0.663	0.3818397	6	-1.73633072	0.1331843	0.59134795
chr4:73526461-75252649.BeroukhimS5.amp	0.672	-0.663	0.3818397	6	-1.73633072	0.1331843	0.59134795
chr4:23402742-26636101.Basal.4p15.2-10.del	-0.107	-0.48383333	0.27227527	5.00000016	-1.77700065	0.13571821	0.59134795
chr8:41004735-41425303.BeroukhimS5.amp	2.164	-0.47333333	0.26643192	5.00000028	-1.77656389	0.13579458	0.59134795
7.q.wholearm.chr7:59100000-158821424	-0.03334596	0.13479574	0.07596659	5.00000001	1.77440824	0.13617216	0.59134795
chr13:104916365-104941384.Basal.13q33.2-84.amp	-0.751	0.41183333	0.23298261	5	1.767657	0.13736158	0.59134795
chr13:106620319-107317084.Basal.13q33.3-85.amp	-0.751	0.41183333	0.23298261	5	1.767657	0.13736158	0.59134795
chr13:108477140-110084607.BeroukhimS2.13q34.amp	-0.751	0.41183333	0.23298261	5	1.767657	0.13736158	0.59134795
chr13:109973420-114110898.Basal.13q34-86.amp	-0.751	0.41183333	0.23298261	5	1.767657	0.13736158	0.59134795
chr13:111767404-114142980.BeroukhimS2.13q34.del	-0.751	0.41183333	0.23298261	5	1.767657	0.13736158	0.59134795
chr13:112030830-113770959.BeroukhimS5.amp	-0.751	0.41183333	0.23298261	5	1.767657	0.13736158	0.59134795
chr22:36048950-38681384.BeroukhimS5.amp	0.00126667	0.35877778	0.2036571	5	1.76167574	0.13842415	0.59134795
chr1:169549478-170484405.BeroukhimS2.1q24.3.amp	1.9095	0.28619444	0.1637272	5	1.74799574	0.14088576	0.59708726
chr9:110838572-127593510.BeroukhimS5.amp	1.026	-0.63278571	0.37738661	6	-1.67675719	0.1446037	0.60801871
chr1:3756302-6867390.BeroukhimS2.1p36.31.del	-0.32957407	0.31090741	0.18101124	4.99999994	1.71761383	0.14651201	0.61122977
chr8:22125332-30139123.BeroukhimS2.8p21.2.del	0.49804412	-0.57773284	0.34053515	5.00000283	-1.69654395	0.15054601	0.62319046
chr7:102988499-103837783.BeroukhimS5.amp	-0.075	0.1475	0.08734253	4.99999994	1.68875355	0.15206564	0.62463887
8.q.wholearm.chr8:45200000-146274826	2.20033538	-0.44127027	0.26806315	5.0000001	-1.64614299	0.16065252	0.65487362
chr5:92250850-96544700.Basal.5q15-38.del	-0.426	-0.264	0.16171394	5	-1.63251227	0.16349999	0.66143179
chr5:82803339-90714877.Basal.5q14.3-37.del	-0.34682353	-0.24243137	0.1490999	5	-1.62596598	0.16488526	0.66202051
chr1:144078052-144943150.BeroukhimS5.amp	1.55508333	0.31233333	0.19318541	5.00000044	1.61675429	0.16685425	0.66492664
chr12:47446248-48387464.Basal.12q13.12-79.del	-1.1445	0.27283333	0.17548847	6	1.55470801	0.17101275	0.67045831
chr12:50987234-51048711.BeroukhimS5.amp	-1.1445	0.27283333	0.17548847	6	1.55470801	0.17101275	0.67045831
chr9:122980237-124410900.Basal.9q33.2-64.del	1.026	-0.69166667	0.44911312	6	-1.54007229	0.17447141	0.67045831
12.q.wholearm.chr12:35400000-132349534	-0.31158204	0.15124917	0.09895482	6	1.52846702	0.17726065	0.67045831
chr16:80588753-82633263.LumA.16q23.3-128.del	0.7255	-0.45738889	0.30426787	6	-1.50324411	0.18346778	0.67045831
chr12:20728852-21227330.BeroukhimS5.del	2.166	-0.40816667	0.26531379	5.00000066	-1.53842987	0.1845587	0.67045831

chr12:25189655-25352305.BeroukhimS2.12p12.1.amp.KRAS	2.166	-0.40816667	0.26531379	5.00000066	-1.53842987	0.1845587	0.67045831
chr12:27342867-29425476.BeroukhimS5.amp	2.166	-0.40816667	0.26531379	5.00000066	-1.53842987	0.1845587	0.67045831
chr12:29425476-31105002.BeroukhimS5.del	2.166	-0.40816667	0.26531379	5.00000066	-1.53842987	0.1845587	0.67045831
chr1:158317017-159953843.BeroukhimS2.1q23.3.amp	1.8776	0.26758	0.17604418	5.00000081	1.51995934	0.18899428	0.67045831
chr1:26377344-27532551.BeroukhimS2.1p36.11.del	0.099	-0.05166667	0.0340183	5.00000002	-1.51879057	0.18927842	0.67045831
chr1:27351829-27460967.BeroukhimS5.amp	0.099	-0.05166667	0.0340183	5.00000002	-1.51879057	0.18927842	0.67045831
chr1:162199047-163197517.BeroukhimS5.amp	1.9095	0.27016667	0.17803881	5.00000084	1.51745942	0.18960252	0.67045831
chr1:163619952-164752515.BeroukhimS5.amp	1.9095	0.27016667	0.17803881	5.00000084	1.51745942	0.18960252	0.67045831
chr1:166729757-167532460.BeroukhimS5.amp	1.9095	0.27016667	0.17803881	5.00000084	1.51745942	0.18960252	0.67045831
chr1:168438419-168880930.BeroukhimS5.amp	1.9095	0.27016667	0.17803881	5.00000084	1.51745942	0.18960252	0.67045831
chr12:44599181-47397048.Basal.12q13.11-78.del	-1.09425862	0.26579885	0.17982529	6	1.4780949	0.18985857	0.67045831
chr14:57736586-58085299.Basal.14q23.1-96.del	-0.3785	0.33969444	0.23112013	6	1.46977437	0.19201815	0.67045831
chr16:80759878-82408573.BeroukhimS2.16q23.3.del.CDH13	0.7255	-0.45116667	0.30723784	6	-1.46846059	0.19236121	0.67045831
chr1:223876038-247249719.BeroukhimS2.1q43.del.RYR2	1.28551852	0.55179938	0.36732416	5.00000009	1.5022137	0.19335315	0.67045831
chr15:1-24740084.BeroukhimS2.15q11.2.del	-0.83380882	0.25023529	0.16830871	5.00000062	1.48676376	0.19722715	0.67947936
9.q.wholearm.chr9:51800000-140273252	1.04891484	-0.50606166	0.35057504	6	-1.44351878	0.19898288	0.68113372
chr8:84799647-85622331.BeroukhimS5.amp	1.366	-0.70633333	0.48162071	5.00000001	-1.46657591	0.20240203	0.68842473
chr7:92829306-132566935.BeroukhimS5.del	-0.02144964	0.14419305	0.10052075	4.99981738	1.43446044	0.21090567	0.71280778
chr19:53372336-53572212.BeroukhimS5.del	-0.0175	-0.35483333	0.24894249	5	-1.4253627	0.21337325	0.71661203
chr3:178149984-199501827.BeroukhimS2.3q28.amp.PIK3CA	1.0786723	-0.29410023	0.21022442	5	-1.3989822	0.22069055	0.73360896
chr5:9224578-10673735.BeroukhimS5.amp	-0.6185	0.40583333	0.29142951	5.00000001	1.39256086	0.22250726	0.73360896
chr12:48421630-53099317.Basal.12q13.13-80.del	-0.87479464	0.15692262	0.11304741	5.00000027	1.38811337	0.2237738	0.73360896
4.q.wholearm.chr4:50700000-191273063	0.75864397	-0.66679507	0.48240326	5.00000002	-1.38223584	0.225458	0.73360896
chr4:54471680-55980061.BeroukhimS2.4q12.amp.PDGFRA.KI	0.912	-0.58433333	0.42865166	5.00000001	-1.36318925	0.23099802	0.73360896
chr17:75915141-77213225.BeroukhimS5.del	0.35	-0.28916667	0.21240046	5.00000027	-1.36142202	0.23151847	0.73360896
chr17:77653047-78605474.BeroukhimS5.amp	0.35	-0.28916667	0.21240046	5.00000027	-1.36142202	0.23151847	0.73360896
chr17:78087533-78774742.BeroukhimS2.17q25.3.del	0.35	-0.28916667	0.21240046	5.00000027	-1.36142202	0.23151847	0.73360896
chr5:36750649-37111693.BeroukhimS5.amp	1.4395	-0.60366667	0.45425022	6	-1.32892984	0.23217213	0.73360896
chr5:39149695-41885012.BeroukhimS5.amp	1.4395	-0.60366667	0.45425022	6	-1.32892984	0.23217213	0.73360896
chr20:59868877-61137250.BeroukhimS5.amp	2.216	-0.84296914	0.63821358	6	-1.32082606	0.23469879	0.73723033
chr6:42772314-43039595.Basal.6p21.1-60.amp	1.9425	-0.56466667	0.43111861	6	-1.30977102	0.23818527	0.73948219
chr6:43556800-44361368.BeroukhimS2.6p21.1.amp	1.9425	-0.56466667	0.43111861	6	-1.30977102	0.23818527	0.73948219
chr20:61329497-62435964.BeroukhimS2.2q13.33.amp	2.216	-0.837	0.64642298	6	-1.29481783	0.24297456	0.74999083
chr6:38787570-41935171.BeroukhimS5.amp	1.35120312	-0.46135937	0.35093469	5.00000071	-1.31465878	0.24569403	0.75402649
chr18:13905410-14018535.BeroukhimS5.amp	0.5485	-0.40866667	0.31840757	5	-1.28347034	0.25559047	0.77991606
chr3:86250885-95164178.BeroukhimS2.3p11.1.amp	1.08288889	-0.67581481	0.54657944	6	-1.2364439	0.26249605	0.7938872
chr7:89924533-98997268.BeroukhimS2.7q21.3.amp.CDK6	-0.03428462	0.25427949	0.20182722	5	1.25988697	0.263315	0.7938872
chr19:1-526082.BeroukhimS2.19p13.3.del	-0.34589286	0.20994048	0.17064189	6	1.23029857	0.26462907	0.7938872
chr4:164612456-170167997.Basal.4q32.3-23.del	1.24138889	-0.86167593	0.69630017	4.99997519	-1.23750641	0.27084173	0.80798593
chr8:29285985-30473918.BeroukhimS5.amp	0.2815	-0.498	0.40532522	5	-1.22864302	0.27387562	0.81249768
chr10:75957348-77987139.LumB.10q22.2-146.del	-0.654	0.02187037	0.0179145	5.00000179	1.22081943	0.27657924	0.81598515
chr8:2053441-6259545.BeroukhimS2.8p23.2.del.CSMD1	-0.143	-0.30716667	0.25382793	5.0000005	-1.2101374	0.28030943	0.81601144
chr8:11575198-11708771.BeroukhimS5.amp	-0.143	-0.30716667	0.25382793	5.0000005	-1.2101374	0.28030943	0.81601144
chr1:241364021-247249719.BeroukhimS2.1q44.amp	1.55432456	0.49247368	0.40778355	5.00000125	1.20768404	0.28117248	0.81601144
chr10:61909390-66364553.BeroukhimS5.amp	-0.32884615	0.24444872	0.2086852	6	1.17137542	0.28585512	0.82495142
chr1:46541957-46555032.Basal.1p33-3.amp	-0.498	-0.10483333	0.08807021	5	-1.19033824	0.28734263	0.82495142
chr19:1818912-2004994.BeroukhimS5.del	-0.371	0.2175	0.18809912	6	1.15630527	0.29151274	0.83244815
12.p.wholearm.chr12:1-35400000	1.62452518	-0.15241787	0.13139878	5.0000028	-1.15996412	0.29843759	0.83937724
chr4:170251982-170915637.Basal.4q33-24.del	1.192	-0.81691667	0.70467921	5.00000058	-1.15927455	0.29869383	0.83937724
chr16:88436931-88827254.BeroukhimS2.16q24.3.del	0.6895	-0.37316667	0.33063808	6	-1.12862581	0.30215178	0.83937724
chr16:82644872-85172804.LumA.16q24.1-129.del	0.7255	-0.388	0.34451286	6	-1.12622791	0.30308867	0.83937724
chr16:85920445-87225756.LumA.16q24.2-130.del	0.7255	-0.388	0.34451286	6	-1.12622791	0.30308867	0.83937724
chrX:139424793-139908341.BeroukhimS5.del	-0.4525	-0.2655	0.23236132	5.00000001	-1.14261702	0.30494229	0.83937724
chrX:148267454-148753845.BeroukhimS5.amp	-0.4525	-0.2655	0.23236132	5.00000001	-1.14261702	0.30494229	0.83937724
18.p.wholearm.chr18:1-16100000	0.81623437	-0.34603125	0.31068414	6	-1.11377185	0.30799472	0.83982374
chr3:1-2121282.BeroukhimS2.3p26.3.del	0.632	0.46066667	0.40773691	5.00000085	1.1298135	0.30982262	0.83982374
chr3:41866-18694914.BeroukhimS5.amp	0.632	0.46066667	0.40773691	5.00000085	1.1298135	0.30982262	0.83982374
16.p.wholearm.chr16:1-38200000	0.35805619	-0.32101453	0.28545687	5.00000001	-1.12456402	0.31184319	0.84103163
chr20:45719063-49181611.LumB.20q13.13-167.amp	1.20683871	-0.37951075	0.33990803	5	-1.11651011	0.31496548	0.8427834
chr19:52031294-53331283.BeroukhimS2.19q13.32.del	0.11958621	-0.2112069	0.19294205	6	-1.09466495	0.31564921	0.8427834
chr3:70107050-74383584.BeroukhimS5.del	0.507	-0.17651852	0.1606214	5.00000002	-1.09897259	0.32185818	0.85173736
chr8:133698327-133914560.BeroukhimS5.amp	2.0395	-0.45766667	0.41676929	5.00000002	-1.09812953	0.32219278	0.85173736
chr3:66644057-68118027.BeroukhimS5.del	0.507	-0.175	0.16103892	5.00000001	-1.08669384	0.32676112	0.85333575
chrX:152729030-154913754.BeroukhimS2.Xq28.amp	-0.0865	-0.21216667	0.19625074	5.00000059	-1.08109997	0.32901593	0.85333575
chr16:87232502-87749670.LumA.16q24.3-131.del	0.97796667	-0.48735556	0.45973036	6	-1.06009001	0.32990112	0.85333575
chr4:175486008-184028292.BeroukhimS5.del	1.21276667	-0.7683	0.71560614	5	-1.07363529	0.33204557	0.85333575
chr16:11352227-11658331.BeroukhimS5.amp	0.388	-0.3105	0.29090373	5.00000011	-1.06736341	0.33460947	0.85333575
chr18:3240326-5937123.BeroukhimS5.del	1.20975	-0.3775625	0.35534706	5.00000114	-1.06251758	0.33660192	0.85333575
chr8:108043446-108683856.BeroukhimS5.amp	3.2265	-0.24533333	0.23282531	5.00000092	-1.05372278	0.34024382	0.85333575
chr6:63255006-65243766.BeroukhimS2.6q12.amp	-0.276	0.574	0.54590225	4.99999999	1.05147029	0.34118192	0.85333575
1.p.wholearm.chr1:1-124300000	-0.06821463	0.04795076	0.0458022	5.00000001	1.04690972	0.34308797	0.85333575
chr4:186684565-191273063.BeroukhimS2.4q35.2.del	1.18528571	-0.75352381	0.72174203	5.00000001	-1.04403481	0.34429412	0.85333575

chr4:162524501-164492534.Basal.4q32.2-22.del	1.0855	-0.751	0.71954467	5.00000002	-1.04371561	0.34442826	0.85333575
chr4:155375138-160500747.Basal.4q32.1-21.del	1.06253704	-0.7404321	0.7101086	5.00000002	-1.04270262	0.34485424	0.85333575
chr7:137042370-158819393.BeroukhimS5.amp	0.17378981	0.08062951	0.077363	5.00000082	1.04222318	0.34505601	0.85333575
chr4:147085600-153449318.BeroukhimS5.del	0.9595	-0.57983333	0.55648673	5.00000069	-1.04195357	0.34516952	0.85333575
chr4:151218876-154929678.Basal.4q31.3-20.del	0.956025	-0.61025833	0.59027307	5.00000067	-1.03385766	0.34859261	0.8565015
chr8:128823745-146264218.BeroukhimS5.del	2.37854508	-0.26447951	0.25809608	5.000001	-1.02473277	0.35248488	0.8565015
chr20:1-325978.BeroukhimS2.2p13.del	0.3875	-0.24216667	0.24183485	5	-1.00137208	0.36261504	0.8565015
22.q.wholearm.chr22:11800000-49691432	0.01456742	0.13568464	0.13562863	5.00000002	1.00041299	0.36303605	0.8565015
chr4:174326479-176135905.Basal.4q34.1-25.del	1.0575	-0.71883333	0.72608895	5.00000001	-0.99000727	0.36762983	0.8565015
chr4:176791083-177486490.Basal.4q34.2-26.del	1.0575	-0.71883333	0.72608895	5.00000001	-0.99000727	0.36762983	0.8565015
chr5:52119531-58183162.Basal.5q11.2-28.del	0.35204688	0.19309896	0.19666889	5.00000114	0.98184803	0.37126523	0.8565015
chr4:143168636-143603331.Basal.4q31.21-19.del	0.9175	-0.54666667	0.55707269	5	-0.98132018	0.37150144	0.8565015
chr6:129217882-131730157.BeroukhimS5.amp	5.00E-04	-0.15083333	0.15705195	6	-0.96040408	0.37393982	0.8565015
chr6:135561194-135665525.BeroukhimS2.6q23.3.amp.MYB	5.00E-04	-0.15083333	0.15705195	6	-0.96040408	0.37393982	0.8565015
chr6:137619861-138589104.BeroukhimS5.amp	5.00E-04	-0.15083333	0.15705195	6	-0.96040408	0.37393982	0.8565015
chr6:139381116-139689363.BeroukhimS5.amp	5.00E-04	-0.15083333	0.15705195	6	-0.96040408	0.37393982	0.8565015
chr6:143157329-144194971.BeroukhimS5.amp	5.00E-04	-0.15083333	0.15705195	6	-0.96040408	0.37393982	0.8565015
chr6:149442557-149779188.BeroukhimS5.amp	5.00E-04	-0.15083333	0.15705195	6	-0.96040408	0.37393982	0.8565015
chr6:157695838-157823719.BeroukhimS5.amp	5.00E-04	-0.15083333	0.15705195	6	-0.96040408	0.37393982	0.8565015
chr6:161612277-163134099.BeroukhimS2.6q26.del.PARK2	5.00E-04	-0.15083333	0.15705195	6	-0.96040408	0.37393982	0.8565015
chr2:159800558-163403274.HER2-enriched.2q24.2-116.amp	0.01491667	-0.08644444	0.09126398	6	-0.94719127	0.38011055	0.8565015
chr9:71280626-72167093.BeroukhimS5.amp	1.0775	-0.2935	0.30574866	4.9996554	-0.9599388	0.38117566	0.8565015
chr7:65877239-79629882.BeroukhimS2.7q21.11.del.MAGI2	-0.35274359	0.17714957	0.18491878	5.00000001	0.95798584	0.38206627	0.8565015
chr8:140458177-146274826.BeroukhimS2.8q24.3.amp	2.44871717	-0.22477778	0.2366428	5.00000162	-0.94986104	0.38580161	0.8565015
20.q.wholearm.chr20:27100000-62435964	0.99530912	-0.28066049	0.29774708	5.00000255	-0.94261376	0.38915834	0.8565015
chr12:55201993-55269875.Basal.12q13.3-82.del	-0.036	-0.24983333	0.26549038	5.0000007	-0.94102593	0.38989692	0.8565015
13.q.wholearm.chr13:16000000-114142980	-0.60106656	0.14097214	0.14982532	5.00000001	0.94090993	0.38995092	0.8565015
7.p.wholearm.chr7:1-59100000	-0.3279798	0.06434624	0.06845611	5.00000103	0.93996339	0.39039178	0.8565015
chr11:107086196-116175885.BeroukhimS2.11q23.1.del.ATM	-0.46493651	-0.1058254	0.11388805	5	-0.92920545	0.39543055	0.8565015
chr12:1-1311104.BeroukhimS2.12p13.33.amp	1.512	-0.0765	0.08240579	5.00000072	-0.92833279	0.39584155	0.8565015
chr14:37128947-38971371.Basal.14q21.1-92.del	-0.3785	0.2285	0.25258013	6	0.9046634	0.40050855	0.8565015
chr14:49113809-52311409.Basal.14q22.1-93.del	-0.3785	0.2285	0.25258013	6	0.9046634	0.40050855	0.8565015
chr14:50062686-50445987.BeroukhimS5.amp	-0.3785	0.2285	0.25258013	6	0.9046634	0.40050855	0.8565015
chr14:52311662-54325595.Basal.14q22.2-94.del	-0.3785	0.2285	0.25258013	6	0.9046634	0.40050855	0.8565015
chr14:54378476-55837783.Basal.14q22.3-95.del	-0.3785	0.2285	0.25258013	6	0.9046634	0.40050855	0.8565015
chr14:56307190-56415610.BeroukhimS5.amp	-0.3785	0.2285	0.25258013	6	0.9046634	0.40050855	0.8565015
chr2:173927807-175001974.HER2-enriched.2q31.1-118.amp	-0.339	0.27833333	0.30796128	6	0.90379328	0.40093446	0.8565015
chr12:38788913-42596599.BeroukhimS2.12q12.amp	-0.79996154	0.20451282	0.22308718	5.00000271	0.91673947	0.4013341	0.8565015
11.q.wholearm.chr11:52900000-134452384	-0.00526702	-0.10379194	0.11354413	4.99999727	-0.91411107	0.40258778	0.8565015
chr20:49438571-52269898.LumB.20q13.2-168.amp	1.2475	-0.30416667	0.34571722	5.00000249	-0.87981347	0.41923092	0.86862549
chr20:51603033-51989829.BeroukhimS2.2q13.2.amp	1.2475	-0.30416667	0.34571722	5.00000249	-0.87981347	0.41923092	0.86862549
chr8:116186189-120600761.BeroukhimS2.8q24.11.amp	2.8905	-0.47891026	0.55277995	6	-0.86636691	0.41958002	0.86862549
chr8:94587173-100685605.BeroukhimS5.amp	1.16693056	-0.51886111	0.59532304	5	-0.87156229	0.42331394	0.86862549
chr2:138479322-143365272.BeroukhimS2.2q22.1.del.LRP1B	0.174	-0.19383333	0.22488507	5.00000001	-0.86192176	0.42812333	0.86862549
chr2:135029078-135167772.BeroukhimS5.amp	0.174	-0.1975	0.23017511	5.00000015	-0.85804239	0.43007046	0.86862549
chr12:30999223-32594050.BeroukhimS2.12p11.21.amp	1.99054545	-0.23271212	0.27217683	5.00000002	-0.85500343	0.43160053	0.86862549
chr7:129281946-131188950.BeroukhimS5.amp	-0.02	0.02966667	0.0352252	6	0.84220006	0.43195849	0.86862549
chr10:89467202-90419015.BeroukhimS2.1q23.31.del.PTEN	-0.654	0.01583333	0.01860003	5	0.85125311	0.43349449	0.86862549
chr22:31596415-33790650.BeroukhimS5.del	-0.043	0.21475	0.25268731	5.00000016	0.8498646	0.43419732	0.86862549
chr12:10732932-10778896.BeroukhimS5.amp	1.463	-0.16933333	0.20036461	5.00000002	-0.84512595	0.43660246	0.86862549
chr12:11410696-12118386.BeroukhimS2.12p13.2.del.ETV6	1.463	-0.16933333	0.20036461	5.00000002	-0.84512595	0.43660246	0.86862549
6.q.wholearm.chr6:60500000-170899992	-0.20611256	-0.11215916	0.13395942	5.00000182	-0.83726224	0.44061609	0.86862549
chr17:18837023-19933105.BeroukhimS2.17p11.2.amp	0.77403333	-0.26162222	0.31757357	6	-0.82381611	0.44155284	0.86862549
chr6:106604408-107782614.BeroukhimS5.amp	-0.2725	-0.08833333	0.10683154	5.00000001	-0.82684696	0.44597503	0.86862549
chr2:154436672-155018734.HER2-enriched.2q23.3-114.amp	-0.3255	-0.04	0.04846647	5.00000112	-0.8253128	0.44676853	0.86862549
chr2:156889194-159797412.HER2-enriched.2q24.1-115.amp	-0.3255	-0.04	0.04846647	5.00000112	-0.8253128	0.44676853	0.86862549
chr21:35022364-36008600.BeroukhimS5.del	-0.287	0.10116667	0.12394766	5.00000077	0.81620471	0.45150129	0.86862549
chr21:38584860-42033506.BeroukhimS2.21q22.2.del.DSCAM.	-0.287	0.10116667	0.12394766	5.00000077	0.81620471	0.45150129	0.86862549
chr3:43362938-44130419.BeroukhimS5.del	0.295	-0.173	0.21339456	5.00000175	-0.81070483	0.45437725	0.86862549
chr19:63402921-63811651.BeroukhimS2.19q13.43.del	1.401	0.42516667	0.53108949	5.00000174	0.8005556	0.45972023	0.86862549
chr3:116900556-120107320.BeroukhimS2.3q13.31.del	0.1265	0.1705	0.21338334	5.00000002	0.79903145	0.46052662	0.86862549
10.q.wholearm.chr10:40300000-135374737	-0.30166667	0.12640901	0.16004093	5.00000181	0.78985424	0.46540409	0.86862549
chr16:31854743-53525739.BeroukhimS2.16q11.2.del	0.5102381	-0.24343254	0.3097753	4.99999552	-0.78583587	0.46755173	0.86862549
6.p.wholearm.chr6:1-60500000	0.91646655	-0.15740816	0.20273886	5.00000101	-0.77640843	0.4726186	0.86862549
chr15:28193434-29022600.Basal.15q13.2-101.del	-0.8085	0.03883333	0.05054464	5.00000079	0.7682978	0.47700969	0.86862549
chr18:3200634-3858775.BeroukhimS5.amp	1.5038	-0.38933333	0.51129209	5.00000177	-0.7614695	0.48072937	0.86862549
chr17:37319013-37988602.BeroukhimS2.17q21.2.del	0.4675	-0.27476812	0.36313711	5.00000183	-0.75665116	0.48336668	0.86862549
chr15:29020705-31945591.Basal.15q13.3-102.del	-0.807125	0.03797222	0.05039474	5.00000072	0.75349577	0.48509942	0.86862549
chr19:7321723-7480918.BeroukhimS5.del	-0.159	0.058	0.07955408	4.99996717	0.72906377	0.49866619	0.86862549
chr6:76630464-105342994.BeroukhimS2.6q16.1.del	-0.58461585	-0.07838211	0.10758782	5.00000001	-0.72854075	0.49895931	0.86862549
chr14:31868274-34078694.Basal.14q13.1-89.del	0.0015	-0.1515	0.21125301	6	-0.71714955	0.50023453	0.86862549
chr14:34100051-35410919.Basal.14q13.2-90.del	0.0015	-0.1515	0.21125301	6	-0.71714955	0.50023453	0.86862549

chr14:35708407-36097605.BeroukhimS2.14q13.3.amp.NKX2-1	0.0015	-0.1515	0.21125301	6	-0.71714955	0.50023453	0.86862549
chr6:101000242-121511318.BeroukhimS2.6q22.1.del	-0.45884659	-0.07738447	0.10696149	5.00000002	-0.72347975	0.5018039	0.86862549
chr17:58249995-58447640.BeroukhimS5.amp	0.4145	0.24066667	0.3414979	6	0.70473835	0.50739265	0.86862549
chr17:59852249-60135967.BeroukhimS5.amp	0.4145	0.24066667	0.3414979	6	0.70473835	0.50739265	0.86862549
chr6:110391-1254918.BeroukhimS5.del	1.2575	0.27066667	0.38409827	6	0.70468077	0.50742602	0.86862549
chr6:1543157-2570302.BeroukhimS2.6p25.3.del	1.2575	0.27066667	0.38409827	6	0.70468077	0.50742602	0.86862549
chr16:45546775-51138307.LumA.16q12.1-120.del	0.4171087	-0.21903623	0.30776974	4.99999999	-0.71168865	0.50847517	0.86862549
chr2:79257340-80427237.BeroukhimS5.del	-0.0185	0.15366667	0.22213093	6	0.69178421	0.5149362	0.86862549
11.p.wholearm.chr11:1-52900000	-0.18596214	-0.10616014	0.15255789	5.00000001	-0.69586789	0.51752258	0.86862549
chr2:121187279-122198612.BeroukhimS5.amp	-0.04133333	-0.14333333	0.20907055	6	-0.68557401	0.51857862	0.86862549
chr8:101163387-103693879.BeroukhimS2.8q22.3.amp	3.10240625	-0.18373958	0.27112876	5	-0.67768387	0.52805685	0.86862549
chr15:35140533-43473382.BeroukhimS2.15q15.1.del	-0.838	0.04683333	0.06924035	5.00000067	0.67638784	0.52881315	0.86862549
chr15:37880223-38115089.Basal.15q15.1-104.del	-0.838	0.04683333	0.06924035	5.00000067	0.67638784	0.52881315	0.86862549
chr15:49136093-51017946.Basal.15q21.2-105.del	-0.838	0.04683333	0.06924035	5.00000067	0.67638784	0.52881315	0.86862549
chr15:51593230-55368004.Basal.15q21.3-106.del	-0.838	0.04683333	0.06924035	5.00000067	0.67638784	0.52881315	0.86862549
chr15:56490060-57176541.Basal.15q22.1-107.del	-0.838	0.04683333	0.06924035	5.00000067	0.67638784	0.52881315	0.86862549
chr15:57184612-61461128.Basal.15q22.2-108.del	-0.838	0.04683333	0.06924035	5.00000067	0.67638784	0.52881315	0.86862549
chr15:61583863-61913200.Basal.15q22.31-109.del	-0.838	0.04683333	0.06924035	5.00000067	0.67638784	0.52881315	0.86862549
chr7:3516940-6019790.BeroukhimS5.amp	-0.3855	0.16334167	0.24168977	4.9999973	0.67583193	0.5291378	0.86862549
chr10:3811260-5139878.LumB.10p15.1-143.del	1.489	-0.282	0.41936771	5.00000013	-0.6724409	0.53112091	0.86862549
chr20:14210829-15988895.BeroukhimS2.2p12.1.del.MACROD	-0.069	-0.16633333	0.2473898	5	-0.67235325	0.53117223	0.86862549
chr20:20585029-20740450.BeroukhimS5.amp	-0.069	-0.16633333	0.2473898	5	-0.67235325	0.53117223	0.86862549
chr4:112023722-118112513.BeroukhimS5.del	0.0675	-0.32933333	0.49300932	5.00000192	-0.6680063	0.53372184	0.86862549
chr4:114219937-114355024.Basal.4q26-18.del	0.0675	-0.32933333	0.49300932	5.00000192	-0.6680063	0.53372184	0.86862549
chr17:62318152-63890591.BeroukhimS2.17q24.2.amp	0.42188462	0.21402564	0.3246616	5.00000001	0.65922685	0.53889614	0.86862549
chr13:27611014-27767472.LumB.13q12.2-162.del	-0.42	0.09033333	0.13731777	5.00000103	0.65784154	0.53971562	0.86862549
chr13:27774389-30804409.LumB.13q12.3-163.del	-0.42	0.09033333	0.13731777	5.00000103	0.65784154	0.53971562	0.86862549
chr13:31211674-31275009.LumB.13q13.1-164.del	-0.42	0.09033333	0.13731777	5.00000103	0.65784154	0.53971562	0.86862549
chr12:95089777-95350380.BeroukhimS2.12q23.1.amp	-0.012	0.1505	0.23167782	6	0.64960902	0.54000186	0.86862549
chr12:8398512-8740196.BeroukhimS5.amp	1.4635	-0.05807143	0.08935124	5.00000001	-0.64992301	0.54441569	0.86862549
chr20:22719364-23648289.BeroukhimS5.amp	0.59710714	-0.16288095	0.25165091	5.00000165	-0.64724961	0.54600855	0.86862549
chr9:1-708871.BeroukhimS2.9p24.3.del	1.141	-0.27357143	0.428631	6	-0.63824462	0.54688676	0.86862549
chr21:44316476-46902240.BeroukhimS5.amp	-0.27023636	0.07710909	0.12067474	5	0.63898287	0.55095337	0.86862549
chr20:29526118-29834552.BeroukhimS2.2q11.21.amp.BCL2L1	0.6075	-0.15783333	0.25010142	5.00000167	-0.63107731	0.55570932	0.86862549
chr20:33386980-33969561.BeroukhimS5.amp	0.6075	-0.15783333	0.25010142	5.00000167	-0.63107731	0.55570932	0.86862549
chr20:40134806-41603948.LumB.20q13.11-165.amp	0.6075	-0.15783333	0.25010142	5.00000167	-0.63107731	0.55570932	0.86862549
chr2:164158152-168812365.HER2-enriched.2q24.3-117.amp	0.186	-0.08183333	0.13042527	5.0000017	-0.62743466	0.55790963	0.86862549
chr12:42034279-44588086.Basal.12q12-77.del	-0.38254167	0.15541667	0.24811485	5.00000001	0.62639001	0.55854168	0.86862549
chr6:54352087-55392731.BeroukhimS5.amp	1.6115	0.283	0.45278369	5.00000001	0.62502252	0.55936975	0.86862549
chr1:43945805-44960161.Basal.1p34.1-2.amp	0.40414286	-0.17513095	0.28113607	5.00000002	-0.62294017	0.56063221	0.86862549
chr12:94480961-96479233.BeroukhimS5.del	-0.012	0.1441	0.23486949	6	0.61353222	0.56204673	0.86862549
chr22:45488286-49691432.BeroukhimS2.22q13.33.del	-0.3515	0.095	0.15409512	5.00000306	0.61650231	0.56454679	0.86862549
chr11:42387077-47453403.BeroukhimS5.amp	-0.323	-0.07083333	0.11504707	5.00000001	-0.61569004	0.56504195	0.86862549
20.p.wholearm.chr20:1-27100000	0.32476738	-0.15468895	0.25214357	5	-0.61349552	0.56638106	0.86862549
chr15:96891354-97698742.BeroukhimS2.15q26.3.amp.IGF1R	-0.0195	0.11166667	0.18261174	4.99999886	0.61149776	0.56760187	0.86862549
chr13:1-40829685.BeroukhimS2.13q12.2.amp	-0.44895082	0.03793579	0.06213915	5.00000002	0.61049741	0.56821379	0.86862549
chr17:44673157-45060263.BeroukhimS2.17q21.33.amp	0.5015	-0.12966667	0.21245584	5.00000172	-0.61032292	0.56832056	0.86862549
chr17:45731662-46009941.BeroukhimS5.amp	0.5015	-0.12966667	0.21245584	5.00000172	-0.61032292	0.56832056	0.86862549
15.q.wholearm.chr15:17000000-100338915	-0.61534274	0.03687493	0.06134412	6	0.60111595	0.5697599	0.86862549
chr21:46690655-46902240.BeroukhimS5.del	-0.287	0.0765	0.12607214	5	0.60679546	0.57048194	0.86862549
chr14:92773649-95250286.Basal.14q32.13-99.del	-0.5953	-0.08674286	0.14299615	5	-0.60660973	0.57059589	0.86862549
chr1:119996566-120303234.BeroukhimS2.1p12.amp	1.0245	0.37083333	0.61560958	5	0.60238395	0.57319228	0.86862549
chr5:1473801-6790134.BeroukhimS5.amp	-0.328175	0.23266667	0.38707205	5	0.60109395	0.57398635	0.86862549
chr17:63942109-65847254.BeroukhimS5.amp	0.4145	0.17742424	0.29616899	5.00000001	0.59906421	0.57523718	0.86862549
chr9:235706-8362053.BeroukhimS5.amp	1.605	-0.36716667	0.6205817	6	-0.5916492	0.57568356	0.86862549
chr9:831690-1047552.Basal.9p24.3-61.amp	1.605	-0.36716667	0.6205817	6	-0.5916492	0.57568356	0.86862549
chr9:7161607-12713130.BeroukhimS2.9p24.1.del.PTPRD	1.605	-0.36716667	0.6205817	6	-0.5916492	0.57568356	0.86862549
chr9:12683435-13935586.Basal.9p23-62.amp	1.605	-0.36716667	0.6205817	6	-0.5916492	0.57568356	0.86862549
chr9:14071847-14900353.Basal.9p22.3-63.amp	1.605	-0.36716667	0.6205817	6	-0.5916492	0.57568356	0.86862549
chr21:16248572-25859193.BeroukhimS5.del	0.4501	-0.30175	0.50523217	5.00000168	-0.59725018	0.57635651	0.86862549
chr11:112785528-114880322.LumB.11q23.2-160.del	-0.48865625	-0.06861458	0.11519262	5	-0.59565087	0.57734449	0.86862549
chr7:3046420-4279470.BeroukhimS2.7p22.2.del.SDK1	-0.3855	0.10383333	0.17432325	5.00000302	0.59563675	0.5773532	0.86862549
chr6:116369389-117096650.LumB.6q22.1-141.del	-0.6545	-0.06466667	0.10911696	5	-0.59263625	0.57920963	0.86862549
chr6:117109043-117359993.LumB.6q22.2-142.del	-0.6545	-0.06466667	0.10911696	5	-0.59263625	0.57920963	0.86862549
chr6:119295855-120272161.BeroukhimS5.amp	-0.6545	-0.06466667	0.10911696	5	-0.59263625	0.57920963	0.86862549
19.p.wholearm.chr19:1-28500000	0.0219965	0.09288199	0.15722833	5	0.59074592	0.58038109	0.86862549
chr7:141592807-142264966.BeroukhimS2.7q34.del.TRB	0.21866667	0.04144444	0.07019627	5	0.59040809	0.5805906	0.86862549
chr1:39907605-40263248.BeroukhimS2.1p34.2.amp.MYCL1	0.5995	-0.06754167	0.11508793	5.00000002	-0.58687013	0.58278754	0.86862549
chr6:31910383-31915520.LumB.6p21.32-140.del	0.5105	-0.10566667	0.18077143	5.00000002	-0.58453189	0.58424232	0.86862549
chr11:26341787-27574592.BeroukhimS5.amp	-0.3335	-0.06633333	0.11483631	5.00000001	-0.5776338	0.58854709	0.86862549
chr6:30420923-31893702.LumB.6p21.33-139.del	0.47428472	-0.1072338	0.18779757	5	-0.57100737	0.59270055	0.86862549
chr12:63329154-63614508.BeroukhimS5.amp	0.15	0.285	0.50032363	5.00000001	0.5696313	0.5935653	0.86862549

chr12:64149851-66323376.BeroukhimS5.del	0.15	0.285	0.50032363	5.00000001	0.5696313	0.5935653	0.86862549
chr12:64461446-64607139.BeroukhimS2.12q14.3.amp.HMGA	0.15	0.285	0.50032363	5.00000001	0.5696313	0.5935653	0.86862549
chr13:72212826-74986423.BeroukhimS5.del	-0.751	0.08816667	0.15719098	5.00000254	0.56088883	0.59907705	0.86862549
chr13:89500014-93206506.BeroukhimS2.13q31.3.amp	-0.751	0.08816667	0.15719098	5.00000254	0.56088883	0.59907705	0.86862549
chr13:92308911-94031607.BeroukhimS2.13q31.3.del.GPC6	-0.751	0.08816667	0.15719098	5.00000254	0.56088883	0.59907705	0.86862549
chr17:20027154-20122158.BeroukhimS5.amp	0.2215	-0.191	0.34103763	5	-0.5600555	0.59960404	0.86862549
21.p.wholearm.chr21:1-12300000	-0.0665	0.21783333	0.38936935	5.00000003	0.55945167	0.59998606	0.86862549
chr12:67440273-67566002.BeroukhimS2.12q15.amp.MDM2	0.157	0.27866667	0.49907205	5.00000001	0.55836961	0.60067101	0.86862549
chr3:58626894-61524607.BeroukhimS2.3p14.2.del.FHIT	0.138875	-0.110875	0.20218062	5.00000004	-0.54839579	0.60700643	0.86862549
9.p.wholearm.chr9:1-51800000	1.27101628	-0.30252946	0.55477939	5.00000001	-0.54531488	0.60897141	0.86862549
chr17:26185485-27216066.BeroukhimS2.17q11.2.del.NF1	0.15833333	-0.164375	0.30605224	5	-0.53708152	0.61424092	0.86862549
chr3:36286137-40063081.BeroukhimS5.amp	0.95144595	-0.12472523	0.23280933	5.00000003	-0.53573981	0.61510216	0.86862549
chr17:22479313-22877776.BeroukhimS2.17q11.1.amp	0.137	-0.1825	0.34101892	4.99997303	-0.53516093	0.61547408	0.86862549
chr17:24112056-24310787.BeroukhimS2.17q11.2.amp	0.137	-0.1825	0.34101892	4.99997303	-0.53516093	0.61547408	0.86862549
chr17:24936541-25468309.BeroukhimS5.amp	0.137	-0.1825	0.34101892	4.99997303	-0.53516093	0.61547408	0.86862549
chr17:28881201-29652449.BeroukhimS5.amp	0.137	-0.1825	0.34101892	4.99997303	-0.53516093	0.61547408	0.86862549
chr17:31895171-33758810.BeroukhimS5.del	0.137	-0.1825	0.34101892	4.99997303	-0.53516093	0.61547408	0.86862549
chr17:33754231-34162198.BeroukhimS5.amp	0.137	-0.1825	0.34101892	4.99997303	-0.53516093	0.61547408	0.86862549
chr17:35067383-35272328.BeroukhimS2.17q12.amp.ERBB2	0.137	-0.1825	0.34101892	4.99997303	-0.53516093	0.61547408	0.86862549
chr14:35837522-37090214.Basal.14q13.3-91.del	-0.04235714	-0.10764286	0.2087202	6	-0.51572802	0.62448991	0.86862549
chrX:1-3243111.BeroukhimS2.Xp22.33.del	-0.4565	-0.021	0.04072591	5.00000002	-0.51564222	0.62808627	0.86862549
chrX:6695466-8345492.BeroukhimS5.del	-0.4565	-0.021	0.04072591	5.00000002	-0.51564222	0.62808627	0.86862549
chrX:8707411-9925292.BeroukhimS5.del	-0.4565	-0.021	0.04072591	5.00000002	-0.51564222	0.62808627	0.86862549
chrX:14236816-19468566.BeroukhimS5.amp	-0.4565	-0.021	0.04072591	5.00000002	-0.51564222	0.62808627	0.86862549
chrX:31041721-34564697.BeroukhimS2.Xp21.2.del.DMD	-0.4565	-0.021	0.04072591	5.00000002	-0.51564222	0.62808627	0.86862549
chr12:53136012-55168448.Basal.12q13.2-81.del	-0.346	-0.07033333	0.13699773	5.00000002	-0.51339051	0.62955066	0.86862549
17.p.wholearm.chr17:1-22200000	0.20069255	-0.2102588	0.41009486	5.00000249	-0.51270772	0.62999509	0.86862549
chr7:144118814-148066271.BeroukhimS2.7q35.del.CNTNAP2	0.338	0.04733333	0.09257129	5.00000002	0.51131762	0.63090047	0.86862549
chr22:20517661-21169423.BeroukhimS2.22q11.22.del	-0.043	0.08	0.15702717	5	0.50946596	0.6321076	0.86862549
chr22:22437934-22507511.BeroukhimS5.del	-0.043	0.08	0.15702717	5	0.50946596	0.6321076	0.86862549
chr22:23139372-23249329.BeroukhimS5.amp	-0.043	0.08	0.15702717	5	0.50946596	0.6321076	0.86862549
chr2:204533830-206266883.BeroukhimS2.2q33.2.del.PARD3B	0.3145	0.14083333	0.27960513	5	0.50368652	0.63588367	0.86862549
chr19:32764651-33569283.BeroukhimS5.amp	1.744	-0.12233333	0.24472606	5.00000002	-0.49987866	0.63837846	0.86862549
chr19:34975531-35098303.BeroukhimS2.19q12.amp.CCNE1	1.744	-0.12233333	0.24472606	5.00000002	-0.49987866	0.63837846	0.86862549
chr19:37265855-38702029.BeroukhimS5.amp	1.744	-0.12233333	0.24472606	5.00000002	-0.49987866	0.63837846	0.86862549
chr19:39664720-39962225.Basal.19q13.11-112.amp	1.744	-0.12233333	0.24472606	5.00000002	-0.49987866	0.63837846	0.86862549
chr15:31945721-37862331.Basal.15q14-103.del	-0.79133333	0.02504444	0.05044254	5	0.49649453	0.64060019	0.86862549
chr18:75796373-76117153.BeroukhimS2.18q23.del	0.095	-0.17366667	0.350103	5.00000002	-0.4960445	0.64089596	0.86862549
chr14:23609961-31698685.Basal.14q12-88.del	0.0015	-0.07159434	0.14724873	5.00000002	-0.48621365	0.64737588	0.87038866
chr12:56419524-56488685.BeroukhimS2.12q14.1.amp.CDK4	0.15	-0.07383333	0.15419642	6	-0.4788265	0.64901225	0.87038866
chr12:56621627-57600529.Basal.12q14.1-83.del	0.15	-0.07383333	0.15419642	6	-0.4788265	0.64901225	0.87038866
chr14:28763785-29367842.BeroukhimS5.amp	0.0015	-0.07066667	0.14696341	5.00000001	-0.48084531	0.65092943	0.87038866
chr17:55144989-55540417.BeroukhimS2.17q23.1.amp	0.1005	0.15033333	0.31267264	5.00000002	0.48080106	0.65095876	0.87038866
chr17:7471230-7717938.BeroukhimS2.17p13.1.del.TP53	0.098	-0.212	0.445187	5.00000277	-0.47620438	0.65400995	0.87038866
chr10:28141103-31360860.Basal.10p11.23-75.amp	1.351125	-0.16234722	0.34210409	5.00000002	-0.47455504	0.65510664	0.87038866
chr22:15272143-16594738.BeroukhimS5.del	0.523	-0.103	0.21713467	5	-0.47435998	0.6552364	0.87038866
chr2:15977811-16073001.BeroukhimS2.2p24.3.amp.MYCN	0.96	0.20866667	0.45149099	5	0.46217239	0.66337144	0.87283621
chr18:14720419-16909449.BeroukhimS5.amp	0.31616667	-0.17433333	0.37820093	5	-0.46095427	0.66418744	0.87283621
chr17:10675416-12635879.BeroukhimS2.17p12.del.MAP2K4	0.098	-0.15666667	0.34326799	5.00000001	-0.45639755	0.6672446	0.87283621
chr17:36989609-37319012.BeroukhimS5.amp	0.28957692	-0.13807692	0.30272829	5.00000003	-0.45610843	0.66743882	0.87283621
chr9:34233377-35934224.BeroukhimS5.amp	0.9395	-0.253	0.55990666	5.00000001	-0.45186103	0.67029547	0.87283621
chr9:36365710-37139941.BeroukhimS2.9p13.2.del.PAX5	0.9395	-0.253	0.55990666	5.00000001	-0.45186103	0.67029547	0.87283621
chr9:36988416-36998984.BeroukhimS5.amp	0.9395	-0.253	0.55990666	5.00000001	-0.45186103	0.67029547	0.87283621
chr9:37861914-38370450.BeroukhimS5.del	0.9395	-0.253	0.55990666	5.00000001	-0.45186103	0.67029547	0.87283621
chr13:46362859-48209064.BeroukhimS2.13q14.2.del.RB1	-0.71355556	0.07525926	0.16737553	5.0000026	0.44964313	0.67178967	0.87283621
chr18:1-587750.BeroukhimS2.18p11.32.del	0.7525	-0.1745	0.40301179	6	-0.43298982	0.68015403	0.87951813
X.qwholearm.chrX:60000000-154913754	-0.52471413	-0.10420052	0.23835389	5.00000216	-0.43716725	0.68022657	0.87951813
chr1:110339388-119426489.BeroukhimS2.1p13.2.del	-0.06553488	0.09240891	0.21759674	5.00000333	0.42467968	0.6887248	0.88406033
chr11:130280899-134452384.BeroukhimS2.11q25.del.OPCML	-0.6775	-0.04883333	0.11547681	5	-0.42288432	0.68995095	0.88406033
chr19:43177306-45393020.BeroukhimS2.19q13.2.amp	1.60466667	0.03704722	0.08827809	5.00000035	0.41966497	0.69215229	0.88406033
chr11:110631916-112776199.LumB.11q23.1-159.del	-0.6775	-0.04816667	0.11553972	5.00000002	-0.41688404	0.69405663	0.88406033
chr17:39354620-40542083.BeroukhimS5.del	0.1235	-0.08633333	0.2077798	5.00000002	-0.41550398	0.69500263	0.88406033
chr19:8920808-8939190.BeroukhimS5.amp	-0.4365	0.0315	0.0762945	5	0.41287382	0.69680728	0.88406033
chr19:10895846-11164554.BeroukhimS5.del	-0.4365	0.0315	0.0762945	5	0.41287382	0.69680728	0.88406033
chr17:51748757-53292386.BeroukhimS5.amp	0.14213636	0.12125758	0.29407938	5	0.41232941	0.69718111	0.88406033
chr20:34179462-45265004.BeroukhimS5.del	0.62965702	-0.11394077	0.28147199	5.00000024	-0.40480323	0.70235897	0.88406033
chr14:92049878-92765306.Basal.14q32.12-98.del	-0.684	-0.05816667	0.14466039	5.00000002	-0.40209119	0.70422932	0.88406033
2.q.wholearm.chr2:93300000-242951149	0.11205782	-0.04840204	0.12119949	4.99997314	-0.39935846	0.70611641	0.88406033
chr12:97551177-99047626.BeroukhimS2.12q23.1.del.ANKS1B	-0.012	0.04066667	0.10311231	6	0.39439197	0.70692821	0.88406033
chr11:1-1391954.BeroukhimS2.11p15.5.del	-0.65142708	-0.046125	0.11709732	5.00000002	-0.39390313	0.70989052	0.88406033
chr10:128584013-129240925.LumB.10q26.2-151.del	-0.255	0.1335	0.34272213	4.99999872	0.38952839	0.71292395	0.88406033
chr10:129812260-135374737.BeroukhimS2.1q26.3.del	-0.255	0.1335	0.34272213	4.99999872	0.38952839	0.71292395	0.88406033

chr5:81303630-82685333.Basal.5q14.2-36.del	-0.0415	-0.15916667	0.41185381	5	-0.38646399	0.71505238	0.88406033
chr10:17311250-19896508.Basal.10p12.33-70.amp	1.521	-0.153	0.39665266	5	-0.38572791	0.71556408	0.88406033
chr12:33156845-33480710.BeroukhimS5.amp	1.8675	-0.10966667	0.28984992	5.00000003	-0.37835672	0.72069756	0.88406033
5.p.wholearm.chr5:1-47700000	0.41464483	-0.10933793	0.29291283	5.00000002	-0.37327806	0.72424422	0.88406033
chr11:68230745-68388280.BeroukhimS5.amp	0.544	0.0555	0.15309142	4.99999999	0.36252848	0.73177695	0.88406033
chr11:69098089-69278404.BeroukhimS2.11q13.2.amp.CCND1	0.544	0.0555	0.15309142	4.99999999	0.36252848	0.73177695	0.88406033
chr11:69588488-69842871.BeroukhimS5.amp	0.544	0.0555	0.15309142	4.99999999	0.36252848	0.73177695	0.88406033
21.q.wholearm.chr21:12300000-46944323	-0.12476889	0.05243259	0.14523648	5	0.36101531	0.73284008	0.88406033
chr10:118946990-119027085.LumB.10q25.3-147.del	-0.226	0.11966667	0.33547998	6	0.35670286	0.73352333	0.88406033
chr10:119032598-121691235.LumB.10q26.11-148.del	-0.226	0.11966667	0.33547998	6	0.35670286	0.73352333	0.88406033
chr10:122206456-122883255.LumB.10q26.12-149.del	-0.226	0.11966667	0.33547998	6	0.35670286	0.73352333	0.88406033
chr5:43612947-44998256.BeroukhimS5.amp	0.598	-0.074	0.20650582	4.99997168	-0.35834342	0.73471905	0.88406033
chr16:5062786-7709383.BeroukhimS2.16p13.3.del.A2BP1	0.388	-0.12022222	0.3406157	6	-0.35295561	0.73619063	0.88406033
chr2:241477619-242951149.BeroukhimS2.2q37.3.del	0.2795	-0.139	0.39033617	5.00000012	-0.3561033	0.73629585	0.88406033
chr7:54899301-55275419.BeroukhimS2.7p11.2.amp.EGFR	-0.344	-0.05316667	0.15034702	5	-0.35362633	0.73804118	0.88406033
chr10:1-1042949.BeroukhimS2.1p15.3.del	1.1185	-0.20883333	0.5982123	5	-0.34909568	0.7412382	0.88406033
chr10:170643-1769670.Basal.10p15.3-65.amp	1.1185	-0.20863333	0.59804201	5	-0.34886067	0.7414042	0.88406033
17.q.wholearm.chr17:22200000-78774742	0.28559813	-0.085805	0.2461252	5.00000002	-0.34862338	0.74157181	0.88406033
chr12:34188071-44391168.BeroukhimS5.del	-0.07135417	0.08191667	0.23504031	5	0.34852178	0.74164359	0.88406033
chr22:18689393-20139185.BeroukhimS5.del	0.22477273	-0.02238636	0.06457166	5.00000003	-0.34669024	0.74293799	0.88406033
chr18:17749667-22797232.BeroukhimS2.18q11.2.amp	0.29286957	-0.12986957	0.37521224	5	-0.34612294	0.74333912	0.88406033
chr12:131913408-132349534.BeroukhimS2.12q24.33.del	0.141	0.15371429	0.45167263	4.99999996	0.34032233	0.74744585	0.88696907
chr5:56146657-56594666.LumB.5q11.2-132.amp	-0.434	0.2025	0.61499756	5.00000002	0.3292696	0.75529724	0.89230513
chr7:16017926-18944036.BeroukhimS5.amp	-0.3255	0.02172727	0.06614943	5	0.32845746	0.75587548	0.89230513
chr10:123214869-123367187.BeroukhimS5.amp	0.7625	0.11616667	0.35531352	5	0.32694131	0.75695547	0.89230513
chr15:75011134-75564998.Basal.15q24.3-111.del	-0.313	-0.04833333	0.15359303	5.00000311	-0.31468443	0.76570923	0.89797834
chr7:156893473-158821424.BeroukhimS2.7q36.3.del.PTPRN2	-0.0295	-0.03547619	0.11392265	4.99999995	-0.31140594	0.76805751	0.89797834
chr20:56249879-56298730.BeroukhimS5.amp	0.7765	-0.08633333	0.27762423	5.00000546	-0.31097189	0.7683686	0.89797834
chr19:44889764-44995371.BeroukhimS5.del	1.744	0.03083333	0.09924495	5.00000002	0.3106791	0.7685785	0.89797834
chr10:12431738-17283687.Basal.10p13-69.amp	1.50513793	-0.11993678	0.39524916	5	-0.30344601	0.77377064	0.89797834
chr9:21489625-22474701.BeroukhimS2.9p21.3.del.CDKN2A.B	1.2295	-0.17166667	0.56950965	5	-0.3014289	0.775221	0.89797834
chr9:23819824-26891068.BeroukhimS5.del	1.2295	-0.17166667	0.56950965	5	-0.3014289	0.775221	0.89797834
chr9:26573226-29470065.BeroukhimS5.amp	1.2295	-0.17166667	0.56950965	5	-0.3014289	0.775221	0.89797834
chr20:41621022-45719023.LumB.20q13.12-166.amp	0.65406579	-0.09147588	0.31893476	5	-0.2868169	0.78575808	0.90730463
chr14:80741860-106368585.BeroukhimS2.14q32.12.del	-0.22761145	-0.04575703	0.16109108	5	-0.28404445	0.78776333	0.90730463
chr3:175446835-178263192.BeroukhimS2.3q26.31.del.NAALA	0.664	-0.10272222	0.36595235	6	-0.28069835	0.78836957	0.90730463
chr8:128414451-128718816.BeroukhimS5.amp	2.8905	0.09066667	0.33129974	5.00000315	0.2736696	0.79528365	0.91026612
chr8:128774432-128849112.BeroukhimS2.8q24.21.amp.MYC	2.8905	0.09066667	0.33129974	5.00000315	0.2736696	0.79528365	0.91026612
chr19:15641747-15670750.BeroukhimS5.amp	0.024	0.08916667	0.32709094	5.00000024	0.27260512	0.7960567	0.91026612
chr1:71284749-74440273.BeroukhimS2.1p31.1.del.NEGR1	-0.2965	-0.04866667	0.18070285	4.99997229	-0.26931876	0.79844505	0.91104628
chr7:115981465-116676953.BeroukhimS2.7q31.2.amp.MET	1.0185	0.01616667	0.06342837	5.00000203	0.2548807	0.80896679	0.9199113
chr10:3811260-6662244.Basal.10p15.1-67.amp	1.489	-0.08910667	0.35090753	5	-0.25393205	0.80965976	0.9199113
10.p.wholearm.chr10:1-40300000	1.37622289	-0.08639759	0.34884618	5	-0.24766673	0.81424137	0.92315264
chr10:101955-12088176.BeroukhimS5.amp	1.40247059	-0.08834967	0.36964618	5	-0.23901146	0.8205845	0.92837314
chrX:66436234-67090514.BeroukhimS2.Xq12.amp.AR	-0.4565	-0.11166667	0.48248977	5.00000001	-0.23143841	0.82614732	0.92977202
chrX:69545961-73353657.BeroukhimS5.amp	-0.4565	-0.11166667	0.48248977	5.00000001	-0.23143841	0.82614732	0.92977202
chr22:19172385-19746441.BeroukhimS2.22q11.21.amp.CRKL	0.1836875	-0.01503125	0.06767809	5	-0.2220992	0.8330234	0.92977202
chr13:1-23902184.BeroukhimS2.13q12.11.del	-0.35174194	0.01141935	0.05240094	5.00000209	0.21792271	0.8361039	0.92977202
chr14:69312342-69568836.Basal.14q24.2-97.del	-0.3455	-0.03566667	0.16509445	5.00000002	-0.21603795	0.83749517	0.92977202
chr14:77311255-79682157.BeroukhimS5.del	-0.3455	-0.03566667	0.16509445	5.00000002	-0.21603795	0.83749517	0.92977202
chr14:77945724-77992686.BeroukhimS5.amp	-0.3455	-0.03566667	0.16509445	5.00000002	-0.21603795	0.83749517	0.92977202
chr5:18240864-21565169.BeroukhimS5.amp	0.76325	-0.05925	0.27851514	6	-0.2127353	0.83857699	0.92977202
3.p.wholearm.chr3:1-91700000	0.42863804	-0.05043388	0.23866313	5.00000002	-0.21131827	0.84098201	0.92977202
chr10:123223889-124739898.LumB.10q26.13-150.del	1.12955882	0.07083333	0.3371214	5	0.21011224	0.84187368	0.92977202
14.q.wholearm.chr14:15600000-106368585	-0.19584044	0.03020848	0.14520303	6	0.20804301	0.84207755	0.92977202
chr11:55503467-56066044.BeroukhimS5.amp	-0.031	-0.02533333	0.12446517	5.00000001	-0.20353754	0.84673932	0.92977202
chr11:57344931-58658339.BeroukhimS5.del	-0.031	-0.02533333	0.12446517	5.00000001	-0.20353754	0.84673932	0.92977202
chr11:57861150-58074632.BeroukhimS5.amp	-0.031	-0.02533333	0.12446517	5.00000001	-0.20353754	0.84673932	0.92977202
chr10:3099740-3205003.Basal.10p15.2-66.amp	1.135	-0.10758333	0.53280383	5.0000054	-0.20191922	0.84793815	0.92977202
chr12:18393150-19784941.BeroukhimS5.amp	1.463	-0.05436667	0.29096431	5.00000001	-0.18684995	0.85912333	0.93699664
chr6:4058046-25852810.BeroukhimS5.del	1.1737	-0.05422963	0.29026679	5.00000288	-0.18682685	0.8591405	0.93699664
chr1:118282114-118807006.BeroukhimS5.amp	1.41125	0.1225	0.65877157	5	0.18595217	0.85979092	0.93699664
chr1:114889793-115149999.BeroukhimS5.amp	-0.262	0.03083333	0.16816568	5.00000002	0.18335093	0.86172596	0.93719279
3.q.wholearm.chr3:91700000-199501827	0.58360893	-0.0222128	0.13173147	6	-0.1686218	0.8716349	0.94604275
chr19:59066340-59471027.BeroukhimS2.19q13.42.amp	0.393	0.04378333	0.26162893	5	0.16734897	0.87365331	0.94631008
chr18:32113759-32649538.BeroukhimS5.del	0.095	-0.0655	0.4113583	5	-0.15922859	0.87972081	0.94673183
chr10:19022250-42889659.BeroukhimS5.del	1.21063333	-0.05391778	0.33935704	5	-0.15888215	0.87997988	0.94673183
chr10:31648148-32903498.Basal.10p11.22-76.amp	1.354	-0.0594	0.38505308	5	-0.15426444	0.88343464	0.94673183
chr14:1-29140968.BeroukhimS2.14q11.2.del	0.0539375	-0.02402474	0.15574681	5.00000002	-0.1542551	0.88344163	0.94673183
chr10:35175415-37212810.BeroukhimS5.amp	1.354	-0.05883333	0.38553784	5	-0.15260067	0.88468012	0.94673183
chr10:38685321-38707438.LumB.10p11.21-144.del	1.354	-0.05883333	0.38553784	5	-0.15260067	0.88468012	0.94673183
chr6:13729714-14088212.Basal.6p23-59.amp	0.911	-0.03883333	0.26917174	5	-0.14426972	0.89092218	0.95150489

18.q.wholearm.chr18:16100000-76117153	0.12008838	-0.05635017	0.40427617	5.00000292	-0.13938533	0.89458607	0.9535109
chr19:39677306-41155075.BeroukhimS5.amp	1.744	-0.01990881	0.15746714	5	-0.12643149	0.90431722	0.96005805
chr10:20145174-20609292.Basal.10p12.32-71.amp	1.216	0.05133333	0.40604742	5	0.12642201	0.90432435	0.96005805
chr5:1212750-1378766.BeroukhimS2.5p15.33.amp.TERT	0.2075	-0.08683333	0.72912813	5.00000002	-0.11909201	0.90983936	0.96399646
chr11:116803699-119605859.LumB.11q23.3-161.del	0.2685	0.0345	0.30402442	5.00000001	0.11347773	0.91406739	0.96626782
chr6:1-23628840.BeroukhimS2.6p24.1.amp	1.240985	0.03642833	0.32688474	5.00000003	0.11144091	0.91560209	0.96626782
chr19:21788507-34401877.BeroukhimS2.19p12.del	1.38928571	-0.02634524	0.25816026	5	-0.10204994	0.92268329	0.97051166
chr2:32460827-55039898.BeroukhimS5.del	0.23868817	0.01878136	0.18542557	5.00000001	0.10128788	0.92325829	0.97051166
chr19:49818193-50114786.BeroukhimS5.amp	0.2765	-0.01283333	0.15157823	5	-0.08466475	0.93581338	0.979391
chr14:95740950-100105884.Basal.14q32.2-100.del	-0.0305	-0.01233333	0.15971524	5.0000027	-0.07722077	0.94144278	0.979391
chr5:45312870-49697231.BeroukhimS2.5p11.amp	0.5385	-0.0145	0.19039924	5.00000336	-0.07615577	0.94224849	0.979391
chr10:21110098-22746531.Basal.10p12.31-72.amp	1.216	-0.0275	0.36401564	5	-0.07554621	0.94270969	0.979391
chr10:22863772-24876778.Basal.10p12.2-73.amp	1.216	-0.0275	0.36401564	5	-0.07554621	0.94270969	0.979391
chr10:24604620-28074753.Basal.10p12.1-74.amp	1.216	-0.0275	0.36401564	5	-0.07554621	0.94270969	0.979391
chr17:41015664-41353348.BeroukhimS5.amp	0.5015	-0.018125	0.25665937	5.00000022	-0.07061889	0.9464386	0.98135576
chr14:65275722-67085224.BeroukhimS2.14q23.3.del	0.0802	-0.01638333	0.25637225	5.00000001	-0.06390447	0.95152246	0.9847151
chr15:65899096-66370691.Basal.15q23-110.del	-0.693	0.00436667	0.07170327	5.00000001	0.06089913	0.95379883	0.98516165
chr11:32027116-37799354.BeroukhimS2.11p13.amp	-0.20595714	-0.00671905	0.12224697	5	-0.05496289	0.95829663	0.98624459
chr19:47121673-49919582.BeroukhimS5.del	0.14226056	0.00676291	0.12377144	5.00000002	0.05464032	0.95854109	0.98624459
chr15:88253886-88997242.BeroukhimS5.amp	-0.647	-0.00795833	0.15478853	5	-0.05141423	0.96098625	0.98648038
chr16:45393460-45522699.LumA.16q11.2-119.del	0.3975	-0.02183333	0.48067982	6	-0.04542178	0.96524513	0.98648038
19.q.wholearm.chr19:28500000-63811651	0.64319594	-0.00542529	0.11886247	5.00000544	-0.04564346	0.9653613	0.98648038
chr1:41717036-43943775.Basal.1p34.2-1.amp	0.17948437	-0.0030625	0.06868029	5.00000003	-0.04459067	0.96615962	0.98648038
chr9:97183463-97625342.BeroukhimS5.del	0.733	0.0145	0.35241729	6	0.04114441	0.96851574	0.98699886
chr14:1-23145193.BeroukhimS2.14q11.2.amp	0.0854	0.00460625	0.16320035	5	0.02822451	0.97857499	0.99350369
chr14:21961377-23608756.Basal.14q11.2-87.del	-0.1123125	0.00434167	0.1554187	6	0.02793529	0.97861974	0.99350369
chr12:68249634-68327233.BeroukhimS2.12q15.amp	0.531	0.00883333	0.39487461	5.00000002	0.02236997	0.98301812	0.99418878
chr12:70849987-70966467.BeroukhimS2.12q21.1.amp	0.531	0.00883333	0.39487461	5.00000002	0.02236997	0.98301812	0.99418878
chr18:44506813-45038631.BeroukhimS5.amp	0.095	0.00783333	0.47533297	5	0.01647968	0.98748909	0.99430481
chr18:46172638-49935241.BeroukhimS2.18q21.2.del.SMAD4.	0.095	0.00783333	0.47533297	5	0.01647968	0.98748909	0.99430481
chr3:170024984-173604597.BeroukhimS2.3q26.2.amp.PRKCI	0.799	-0.00419444	0.28459528	6	-0.01473828	0.98871883	0.99430481
chr14:93528266-103946058.BeroukhimS5.amp	-0.20121348	0.00154307	0.16791519	6	0.00918959	0.9929658	0.99647331
chr17:70767943-71305641.BeroukhimS2.17q25.1.amp	0.41747222	-0.00119444	0.19947752	5.00000338	-0.00598787	0.99545397	0.99647331
chr10:7244255-12332593.Basal.10p14-68.amp	1.489	0.00166667	0.35879294	5.00000001	0.0046452	0.99647331	0.99647331
